# Photoredox-catalyzed branch-selective pyridylation of alkenes for the expedient synthesis of Triprolidine

**DOI:** 10.1038/s41467-019-08669-1

**Published:** 2019-02-14

**Authors:** Shengqing Zhu, Jian Qin, Fang Wang, Huan Li, Lingling Chu

**Affiliations:** State Key Laboratory for Modification of Chemical Fibers and Polymer Materials, Center for Advanced Low-Dimension Materials, College of Chemistry, Chemical Engineering and Biotechnology, Donghua University, 201620 Shanghai, China

## Abstract

Alkenylpyridines are important pharmaceutical cores as well as versatile building blocks in organic synthesis. Heck reaction represents one of the most powerful platform for the construction of aryl-substituted alkenes, nevertheless, examples for Heck type coupling of alkenes with pyridines, particularly with branched selectivity, remain elusive. Here we report a catalytic, branch-selective pyridylation of alkenes via a sulfinate assisted photoredox catalysis. This reaction proceeds through a sequential radical addition/coupling/elimination, by utilizing readily available sodium sulfinates as reusable radical precursors as well as traceless elimination groups. This versatile protocol allows for the installation of important vinylpyridines with complete branched selectivity under mild conditions. Furthermore, this catalytic manifold is successfully applied to the expedient synthesis of Triprolidine.

## Abstract (web summary)

Alkenylpyridines are versatile building blocks for the synthesis of drugs and other complex molecular structures. Here, the authors show a branch-selective pyridylation of alkenes via sulfinate-assisted photoredox catalysis and showcase its utility in an expedient synthesis of Triprolidine.

## Introduction

**Fig. 1:**
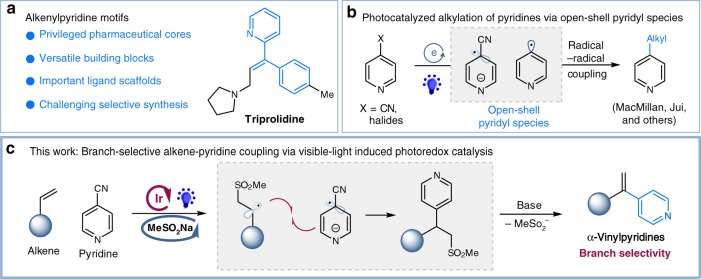
Design of catalytic and branch-selective alkene–pyridine coupling via photoredox catalysis. **a** Importance of alkenylpyridines. **b** Photocatalyzed alkylations of pyridines. **c** Design of branch-selective alkenylation of pyridines via photoredox catalysis

Pyridine is recognized as one of the most important heterocycles in pharmaceuticals, agrochemicals, and bioactive natural products^1,2^. Alkenylpyridines, an important subunit of pyridines, also serve as versatile synthetic building blocks for complex pyridines^3,4^, as well as important ligand scaffolds in the area of catalysis (Fig. 1a)^5^. As a result, the efficient and selective assembly of alkenylpyridines from readily available starting materials has drawn intensive attentions of chemists^6–10^.

Alkenes are one of the most ubiquitous material in organic synthesis. The cross-coupling of alkenes and aryl halides in the presence of palladium catalyst, the Heck reaction^11–13^, is a powerful protocol for the construction of aryl-substituted alkenes. Nevertheless, there are few examples of Heck coupling of alkenes with pyridines, probably due to the potential coordination between the nitrogen atom and metal catalyst^14–19^. Alternatively, several examples of Pd-catalyzed cross-coupling of alkenes with pyridine *N*-oxides have been reported, while only delivering vinylpyridine derivatives with linear selectivity^20–23^. To the best of our knowledge, catalytic alkene–pyridine couplings with branch selectivity, the control of which remains a big challenge in Heck couplings^24–26^, is unknown.

Recently, radical-based chemistry provides an alternative platform to address this challenge. Particularly, the groups of MacMillan, Jui, and others^27–46^ have successfully demonstrated that visible light photocatalyzed cross-couplings of pyridines to access to alkylated pyridines under mild conditions, by taking advantage of unique reactivity of open-shell pyridyl radical species (Fig. 1b). While significant advances, no examples of vinylation of pyridines via pyridyl radical species has been reported. Herein, we demonstrate an alternative protocol, through a sequential radical addition/radical coupling/elimination pathway, to access vinylpyridines from readily available alkenes with complete branch selectivity (Fig. 1c). This reaction takes advantage of a synergistic combination of visible light-induced photoredox catalysis and a catalytic radical precursor, providing an effective and selective strategy for the synthesis of α-vinylpyridines under mild conditions.

## Results

### Design plan

**Fig. 2:**
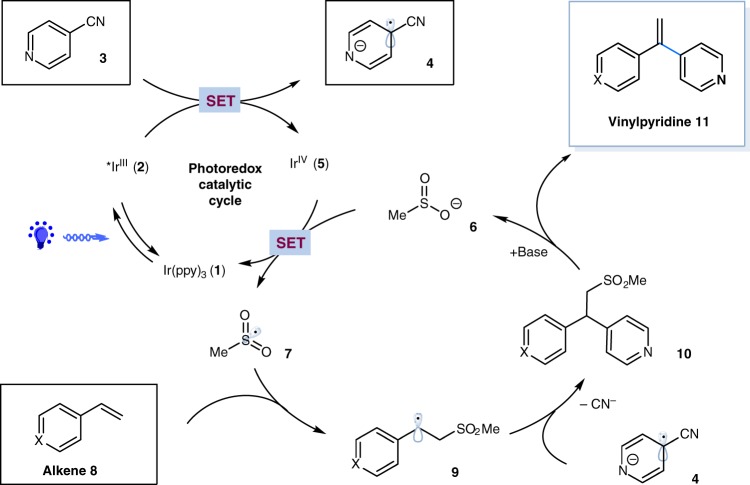
Proposed mechanism. Possible reaction pathway utilizing sulfinate as a promoter

We hypothesized that sodium sulfinates would be the ideal reusable radical precursors, due to the unique properties of which: (i) sufinates have been recently employed as efficient sulfonyl coupling partners in photoredox catalysis^47–62^; (ii) it is well known that the alkyl sulfones would be prone to undergo desulfonylation under basic conditions^63–65^. As depicted in Fig. 2, we envisioned that a single-electron reduction between photoexcited *Ir(ppy)_3_ **2** {*E*_1/2_^red^ [*Ir^III^/Ir^IV^] = –1.73 V vs. SCE }^66^ and cyanopyridine **3** (*E*_1/2_^red^ = –1.75 V vs. SCE in CH_3_CN)^67^ could be feasible under specific conditions, generating pyridyl radical anion species **3** and the oxidizing Ir^IV^ **5**. We hypothesized that Ir^IV^ **5** {*E*_1/2_^red^[Ir^IV^/Ir^III^] = +0.77 V vs. SCE}^66,68^ could affect the oxidation of sodium methanesulfinate **6** (*E*_1/2_^red^ = +0.50 V vs. SCE) (see Supplementary Fig. 2) to form sulfonyl radical **7** and regenerate the ground-state Ir^III^ catalyst **1**. The electrophilic sulfonyl radical **7** would subsequently undergo facile radical addition to alkene to generate the nucleophilic benzylic radical **9**. At this stage, we envisioned that radical–radical coupling between the transient benzylic radical **9** and the persistent pyridyl radical anion **4** would forge β-sulfonyl pyridine **10**^32–46^. Due to the acidity of the benzylic proton and the good leaving ability of sulfone, alkyl sulfone **10** would be expected to undergo E1 elimination with the assistant of base, furnishing the final branched alkenylpyridine product **11**, as well as sulfinate **6** that could be recycled.

### Optimization study

Our investigation into this photoredox cascade protocol began with exposure of 1-(*tert*-butyl)-4-vinylbenzene **12** and 4-cyanopyridine **3** to a 90 W blue light-emitting diode (LED) in the presence of catalytic amounts of Ir(ppy)_3_ (5 mol%) and readily available MeSO_2_Na (30 mol%) (Table 1). In the presence of a stoichiometric amount of 1,8-diazabicyclo[5.4.0]undec-7-ene (DBU) as base; pleasingly, we found that the branched vinylpyridine product could be obtained in 69% yield (entry 1). The nature of sodium sulfinates was found to have an important effect to the reaction efficiency. Generally, electron-poor aryl sulfinates, which are better leaving groups, afforded higher efficiency than electron-rich ones (entries 2–6). And, comparable yields were obtained when *para*-chlorophenyl sulfinate was employed as the co-catalyst (entry 4). Gratifyingly, increasing the photocatalyst loading to 5 mol% provided the optimal yield of product (entry 7). Control experiments indicated that photocatalyst, sulfinate, and light were all essential to this transformation, as no desired products were observed in the absence of either photocatalyst, sulfinate, or light (entries 8–10). While conducting the reaction in the absence of DBU afforded the expected product in 18% yield, probably due to the basic condition in the presence of MeSO_2_Na (entry 11). Interestingly, a trace amount of C2-substituted product **13′**, which is assumed to be formed by the S_N_Ar reaction of cyanopyridine with alkyl radical, was observed under the reaction conditions^69^.

**Table 1:** Optimization of reaction conditions^a^


Entry	Ir(ppy)_3_	RSO_2_Na (30 mol%)	Yield^b^
1	1 mol%	MeSO_2_Na	69%
2	1 mol%	PhSO_2_Na	52%
3	1 mol%	4-F-C_6_H_4_SO_2_Na	50%
4	1 mol%	4-Cl-C_6_H_4_SO_2_Na	61%
5	1 mol%	4-CF_3_-C_6_H_4_SO_2_Na	45%
6	1 mol%	4-OMe-C_6_H_4_SO_2_Na	28%
7	5 mol%	MeSO_2_Na	86%
8	–	MeSO_2_Na	0%
9	5 mol%	–	0%
10^c^	5 mol%	MeSO_2_Na	0%
11^d^	5 mol%	MeSO_2_Na	18%

*DBU* 1,8-diazabicyclo[5.4.0]undec-7-ene, *LED* light-emitting diode, *GC* gas chromatography

^a^Reaction conditions: Ir(ppy)_3_ (5 mol%), RSO_2_Na (30 mol%), styrene (0.1 mmol), 4-cyanopyridine (2.0 equiv.), DBU (3 equiv.), MeCN/EtOH, 90 W blue LED, 40 °C, 24 h

^b^Yields were determined by GC using an internal standard

^c^Performed in the dark

^d^Performed in the absence of DBU

### Substrate scope

**Fig. 3:**
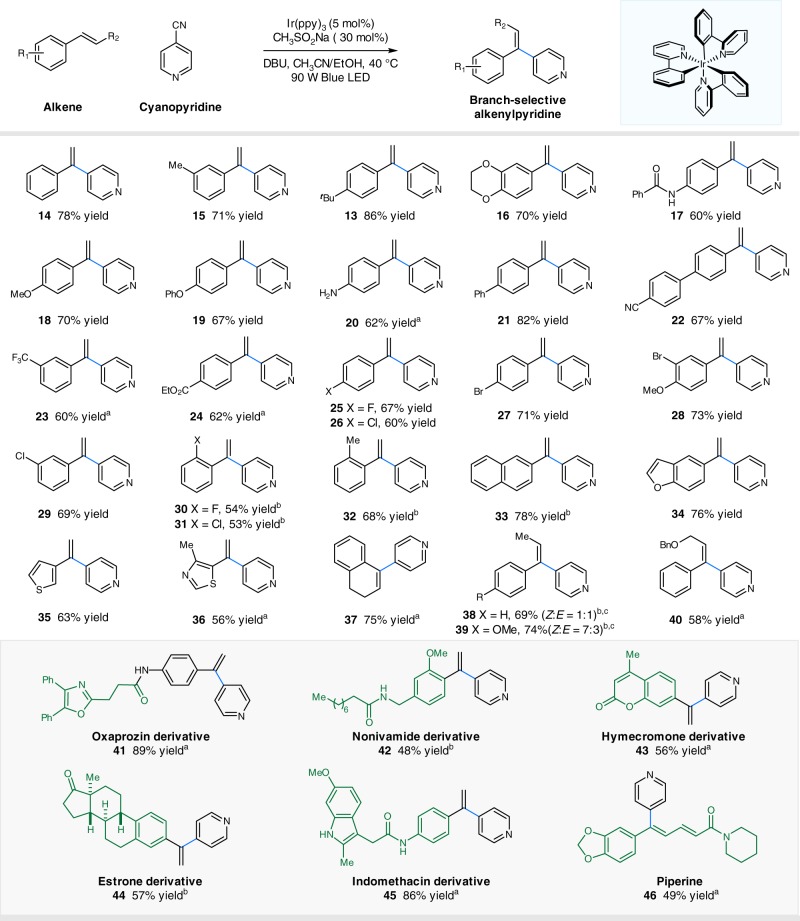
Substrate scope of olefins. Reaction conditions: Ir(ppy)_3_ (5 mol%), MeSO_2_Na (30 mol%), alkene (0. mmol), 4-cyanopyridine (2.0 equiv.), DBU (3 equiv.), MeCN/EtOH (1:1), 90 W blue light-emitting diode (LED), 40 °C, 24 h. All cited yields are isolated yields. ^a^Employed with 4-Cl-C_6_H_4_SO_2_Na (1.0 equiv). ^b^Employed with 4-Cl-C_6_H_4_SO_2_Na (0.5 equiv.). ^c^The ratio was determined by proton nuclear magnetic resonance (^1^H NMR) analysis

With the optimal conditions in hand, we next explored the generality of this transformation with respect to alkene component using 4-cyanopyridine as the coupling partner. As shown in Fig. 3, styrenes incorporating electron-donating and electron-withdrawing substituents readily underwent the desired radical addition/coupling/elimination cascade reactions, furnishing branched vinylpyridines in good to high yields (products **13**–**32**, 53–86% yields). The mild conditions tolerate a wide range of functional groups, including ethers, amides, esters, nitriles, chlorides, bromides, ketones, and amines (products **16**–**20**, **22**–**31**, 53–73% yields). Moreover, *ortho* substituents (Me, F, Cl) on the aryl ring were compatible in this manifold, albeit with slightly diminished yields (products **30**–**32**, 53–68% yields). Furthermore, this catalytic protocol was applicable to other aryl and heteroaryl alkenes, in the form of naphthalene, benzofuran, thiophene, and thiazole, furnishing the corresponding products in moderate yields (products **33**–**36**, 56–78% yields). It should be noted that both cyclic (e.g., 1,2-dihydronaphthalene) and acyclic (e.g., *trans*-β-methylstyrenes and benzyl ether of cinnamyl alcohol) internal alkenes were found to be viable substrates, delivering the corresponding alkenylpyridines with exclusive selectivity at the benzylic positions, respectively (products **37**–**40**, 69–75% yields). Finally, styrenes derived from drugs or natural products, including oxaprozin, nonivamide, hymecromone, estrone, and indomethacin, could be successfully employed to furnish the desired products in moderate to good yields (products **41**–**45**, 48–89% yields), further highlighting the potential synthetic utility of this new protocol. Notably, piperine, a naturally occurring alkaloid, underwent selective pyridylation with synthetic useful yields (product **46**, 49% yield). Nevertheless, un-activated alkenes were ineffective under the reaction conditions, and most of alkene materials remained, probably due to their weaker electrophilic nature.

**Fig. 4:**
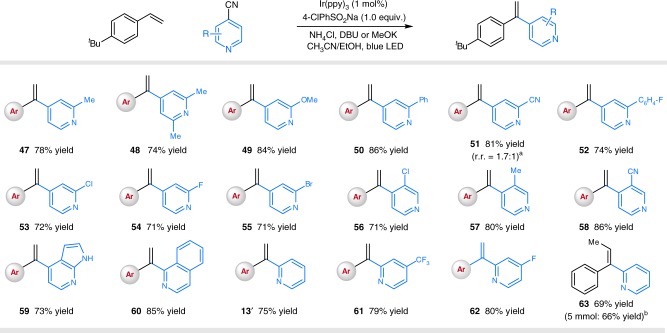
Substrate scope of pyridines. Reaction conditions: Ir(ppy)_3_ (1 mol%), 4-Cl-PhSO_2_Na (1.0 equiv.), styrene (0.2 mmol), cyanopyridine (2.0 equiv.), NH_4_Cl (2 equiv.), base (6 equiv.), MeCN/EtOH, 90 W blue light-emitting diode (LED, 40 °C, 2 h. All cited yields are isolated yields. ^a^The ratio was determined by ^1^H NMR analysis. ^b^Reaction performed on a 5 mmol scale. see Supplementary Fig. 1 for details. Ar = 4-*tert*-butylphenyl

We next sought to explore the scope regarding cyanopyridines (Fig. 4). Nevertheless, we found that only 4-cyanopyridines with alkyl and/or aryl substituents at the 2-positions underwent the desired couplings with moderate efficiency under the standard conditions (products **47**–**48**, and **50**, with yields around 50%). Pleasingly, employing a stoichiometric amount of 4-chlorophenyl sulfinate as the radical precursor and NH_4_Cl as the additive could improve the reaction efficiency. Under the modified conditions, a wide range of cyanopyridines could be successfully employed, furnishing the desired α-vinylpyridines in moderate to good yields (products **47**–**58**, 71–86% yields). Notably, halogen atoms remained intact under the photocatalytic conditions, offering useful handles for further synthetic manipulations (products **53**–**56**, 71–72% yields). Interestingly, 3,4-dicyanopyridine underwent selective coupling at C4 (product **58**, 86% yield), while 2,4-dicyanopyridine afforded a mixture of regioisomeric products (product **51**, 81% yield, r.r. = 1.7:1). *Ortho* substituents on the pyridines have no deleterious effect to the coupling/elimination efficiency (products **56**–**58**, 71–86% yields). Moreover, unprotected azaindole nitrile also was a viable substrate (product **58**, 73% yield). Notably, 1-cyanoisoquinoline and 2-cyanopyridines also underwent the selective alkenylation smoothly, delivering the corresponding vinylquinoline and vinylpyridines with moderate efficiency (products **13′**, **60**–**63**, 69–85% yields). Pleasingly, performing the photocatalytic reaction on a 5 mmol scale afforded product **63** with comparable efficiency (see Supplementary Fig. 1 for details).

**Fig. 5:**
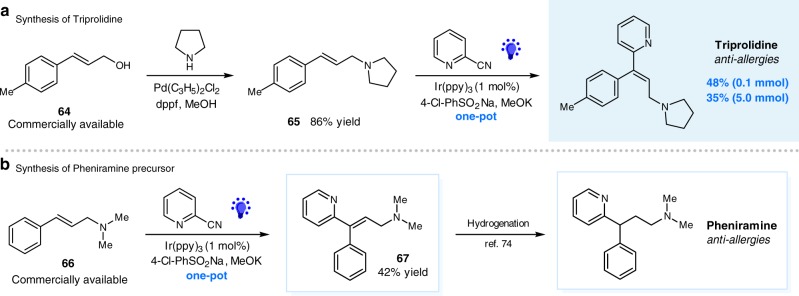
Synthesis of Triprolidine. **a** One-pot synthesis of Triprolidine via photoredox catalysis. **b** Synthesis of pheniramine precursor

To demonstrate the synthetic application of this sequential photoredox protocol, we have accomplished the expedient synthesis of Triprolidine, a top-selling antihistamine that used for allergies^70,71^. As shown in Fig. 5a, Pd-catalyzed amination of commercially available allylic 4-methylcinnamic alcohol **64** gave the allylic amine precursor **65** in 86% yield^72^. Reaction of **65** with simple 2-cyanopyridine under our photocatalytic conditions directly delivered Triprolidine in a simple and one-pot operation with a synthetic useful yield (48% yield). Notably, a 5 mmol scale synthesis of Triprolidine was performed, albeit with slightly decreased efficiency (35% yield). Furthermore, subjection of allylic amine **65** to the photocatalytic condition, yielding the desired pyridyl product **66**, a precursor for drug pheniramine^73^ through one-step hydrogenation^74^, with 42% yield (Fig. 5b).

### Mechanistic studies

**Fig. 6:**
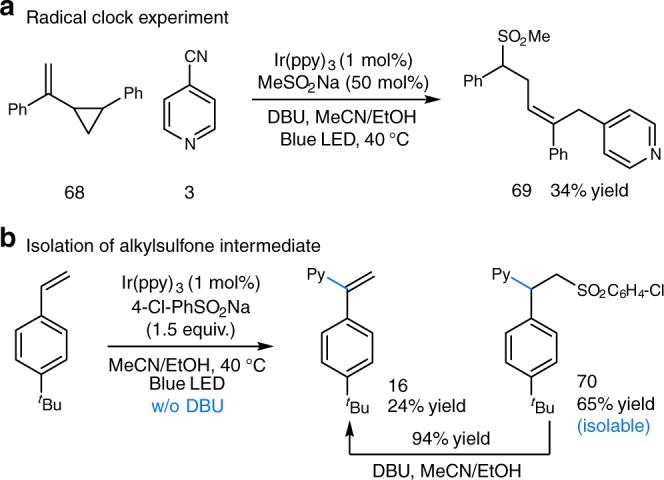
Mechanistic studies. **a** Radical clock experiment. **b** Isolation of alkyl sulfone intermediate

To further probe the mechanism proposal of this photocatalytic reaction, we performed Stern–Volmer fluorescence quenching studies. As shown in Supplementary Fig. 3, only cyanopyridine was found to quench the excited state of *Ir(ppy)_3_, while no significant quenching was observed in the presence of sulfinate or DBU, lending support for our proposed oxidative quenching pathway (Fig. 2). Next, investigation of the intermediacy of a sulfonyl radical was conducted (Fig. 6). Reaction of vinylcyclopropane **68** with MeSO_2_Na and 4-cyanopyridine in the presence of Ir(ppy)_3_ and DBU afforded product **69**, which could be formed through a sequential sulfonyl addition/ring opening/radical coupling protocol (Fig. 6a). Furthermore, the reaction of styrene and 4-chlorophenyl sulfinate, in the absence of DBU, gave 24% of the desired vinylpyridine product **16** as well as 65% of isolable β-sulfonyl pyridine **70**. As expected, treatment of **70** with DBU gave vinylpyridine **16** in 94% yield (Fig. 6b). These results further supported the intermediacy of a sulfonyl radical species in the transformation. Regarding the coupling step for forging the C–pyridine bond, nevertheless, we cannot rule out alternative pathway that proceeds via S_N_Ar reaction of pyridine with alkyl radical **9**, particularly with the observation of a trace amount of C2-substituted product (see Supplementary Fig. 4 for this alternative pathway).

## Discussion

In conclusion, we have developed an efficient strategy for branch-selective, formal alkene–pyridines cross-coupling via sulfinate-assisted photoredox catalysis. This versatile protocol utilizes a sequential radical addition, radical coupling, and β-elimination protocol, allowing for the construction of branched alkenylpyridines from simple starting material under mild conditions. Notably, an expedient and an operationally simple, one-pot synthesis of Troprilidine has been successfully achieved through this photocatalytic manifold.

## Methods

### General procedure for the branch-selective alkenylpyridylation reaction

To a flame-dried 20 mL reaction vial was charged with Ir(ppy)_3_ (1.0 mol%), MeSO_2_Na (30 mol%), 4-cyanopyridine (0.4 mmol, 2.0 equiv.), and a magnetic stir bar. MeCN/EtOH (1:1 v/v) [0.02 M] was added, and the vial was capped. The reaction mixture was degassed by nitrogen sparging for 15 min, followed by the addition of alkenes (0.2 mmol, 1.0 equiv.) and DBU (0.6 mmol, 3.0 equiv.). The reaction mixture was then irradiated with a 90 W blue LED for 24 h at 40 °C. The reaction mixture was concentrated in vacuo and then quenched with water, extracted with ethyl acetate. The combined organic layers were dried with MgSO_4_, filtered, and concentrated in vacuo. The crude material was purified by flash chromatography to afford the products. See Supplementary Methods for further experimental details.

## Supplementary information

Supplementary Information

## Data Availability

The authors declare that all the data supporting the findings of this work are available within the article and its Supplementary Information files, or from the corresponding author upon request.
