# Supplementary material for: Photoredox-catalyzed branch-selective pyridylation of alkenes for the expedient synthesis of Triprolidine
Source: Nat Commun. 2019 Feb 14;10:749. doi: 10.1038/s41467-019-08669-1 (PMC6375965; doi:10.1038/s41467-019-08669-1)
Supplement: Supplementary file 1 — Supplementary Information [file 41467_2019_8669_MOESM1_ESM.pdf]

Supplementary Information for

Photoredox-catalyzed branch-selective pyridylation of alkenes for the expedient synthesis of Triprolidine

Chu *et al*

correspondence to：lingling.chu1@dhu.edu.cn

This PDF Files includes:

Supplementary Methods

Supplementary Discussion

Supplementary Figure 1-130

Supplementary References

Supplementary Methods

General Considerations: Commercial reagents were purchased from Aldrich, TCI, Energy Chemical and J&K chemical, and were used as received. All reactions were carried out in oven-dried glassware under an atmosphere of nitrogen unless otherwise noted. Chromatographic purification of products was accomplished by flash chromatography using silica gel. Thin-layer chromatography (TLC) was performed on Silicycle 250 mm silica gel F-254 plates. ^{1}H, ^{19}F NMR, and ^{13}C NMR spectra were recorded on Bruker 400 (400, 376, and 100 MHz) and Bruker 600 (600, 564, and 150 MHz), and are internally referenced to residual solvent signals (for $CDCl_3$, δ 7.26 and 77.0 ppm). Data for ^{1}H NMR and ^{19}F NMR are reported as follows: chemical shift (δ ppm), multiplicity (s = singlet, d = doublet, t = triplet, q = quartet, m = multiplet, br = broad), integration, coupling constant (Hz). ^{13}C spectra were reported as chemical shifts in ppm and multiplicity where appropriate. High resolution mass spectra were obtained at Shanghai Institute of Organic Chemistry mass spectrometry facilities. All alkenes were used from commercial suppliers or prepared using standard literature procedures or the preparation procedures descried in this supporting information.

Preparation of substrates

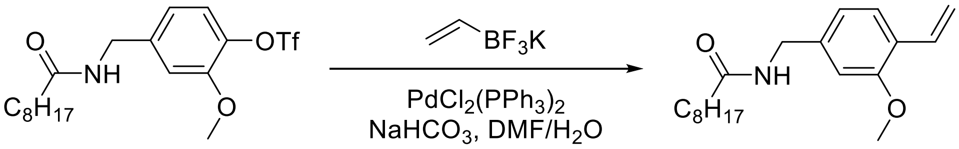

To a solution of 2-methoxy-4-(nonanamidomethyl)phenyl trifluoromethanesulfonate (400.0 mg, 0.94 mmol) and potassium vinyltrifluoroborate (251.8 mg, 1.88 mmol), $NaHCO_3$ (317 mg, 3.76 mmol) in DMF (7.2 mL) and water (0.72 mL) was added $PdCl_2(PPh_3)_2$ (32 mg, 5 mol%). The reaction mixture was degassed by N_2 sparging for 15 min, and then stirred at 70 °C for 20 h under N_2. The reaction was cooled down to room temperature and diluted with ethyl acetate. The organic layer was washed with water and brine. The organic layer was dried over anhydrous magnesium sulfate, and concentrated in vacuo. The crude material was purified by flash chromatography to afford the product (231 mg, 81%).

N-(3-Methoxy-4-vinylbenzyl)nonanamide (S1): ^{1}H NMR (400 MHz, $CDCl_3$) δ 7.42 (d, J = 7.8 Hz, 1H), 7.00 (dd, J = 17.8, 11.2 Hz, 1H), 6.83 (d, J = 7.8 Hz, 1H), 6.79 (s, 1H), 5.72 (dd, J = 17.8, 1.5 Hz, 1H), 5.69 – 5.59 (m, 1H), 5.26 (dd, J = 11.2, 1.5 Hz, 1H), 4.42 (d, J = 5.7 Hz, 2H), 3.84 (s, 3H), 2.26 – 2.13 (m, 2H), 1.70 – 1.61 (m, 2H), 1.36 – 1.18 (m, 10H), 0.87 (t, J = 6.9 Hz, 3H). ^{13}C NMR (150 MHz, $CDCl_3$) δ 172.99, 156.91, 139.38, 131.25, 126.72, 126.06, 119.90, 114.56, 110.44, 55.52, 43.59, 36.87, 31.82, 29.34, 29.34, 29.17, 25.82, 22.66, 14.11. HRMS (ESI): calcd for $C_{19}H_{30}NO_2^+$ (M+H) 304.2262, found: 304.2277.

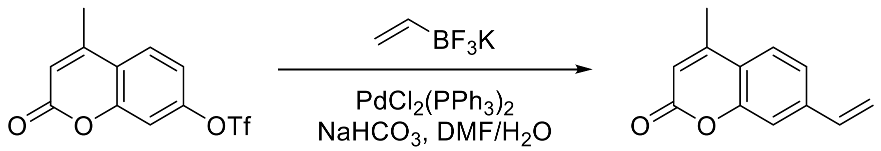

To a solution of 4-methyl-2-oxo-2H-chromen-7-yl trifluoromethanesulfonate (290.0 mg, 0.94 mmol) and potassium vinyltrifluoroborate (251.8 mg, 1.88 mmol), $NaHCO_3$ (317 mg, 3.76 mmol) in DMF (7.2 mL) and water (0.72 mL) was added $PdCl_2(PPh_3)_2$

(32 mg, 5 mol%). The reaction mixture was degassed by N_2 sparging for 15 min, and then stirred at 70 °C for 20 h under N_2. The reaction was cooled down to room temperature and diluted with ethyl acetate. The organic layer was washed with water and brine. The organic layer was dried over anhydrous magnesium sulfate, and concentrated in vacuo. The crude material was purified by flash chromatography to afford the product (122.5 mg, 69%).

4-methyl-7-vinyl-2H-chromen-2-one (S2): ^{1}H NMR (600 MHz, $CDCl_3$) δ 7.54 (d, *J* = 8.2 Hz, 1H), 7.33 (d, *J* = 8.2 Hz, 1H), 7.32 (s, 1H), 6.74 (dd, *J* = 17.6, 10.9 Hz, 1H), 6.25 (d, *J* = 0.8 Hz, 1H), 5.88 (d, *J* = 17.6 Hz, 1H), 5.42 (d, *J* = 10.9 Hz, 1H), 2.42 (d, *J* = 1.0 Hz, 3H). ^{13}C NMR (150 MHz, $CDCl_3$) δ 160.93, 153.84, 152.13, 141.32, 135.37, 124.69, 122.13, 119.38, 117.15, 114.70, 114.32, 18.64. HRMS (ESI): calcd for $C_{12}H_{11}O_2^+$ (M+H) 187.0754, found: 187.0749.

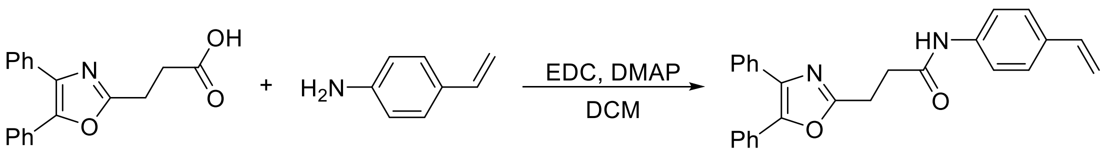

According to the previous reference,[1] a mixture of 3-(4,5-diphenyloxazol-2-yl)propanoic acid (293.3 mg, 1.0 mmol), 4-vinylaniline (121.5 mg, 1.02 mmol), 1-(3-dimethylaminopropyl)-3-ethylcarbodiimide hydrochloride (287.6 mg, 1.5 mmol), and DMAP (2.5 mg, 0.02 mmol) in CH_2Cl_2 (20.0 mL) was stirred at room temperature for 2.5 h. The reaction mixture was added to H_2O and extracted with CH_2Cl_2. The organic layers were washed with brine, dried over $MgSO_4$, filtered, concentrated in vacuo, and flash chromatographed (EtOAc/hexane = 1:5) to afford the product (340 mg, 86%).[1]

3-(4,5-diphenyloxazol-2-yl)-N-(4-vinylphenyl)propenamide (S3): ^{1}H NMR (600 MHz, $CDCl_3$) δ 8.59 (s, 1H), 7.64 (d, *J* = 7.0 Hz, 2H), 7.56 (d, *J* = 8.1 Hz, 2H), 7.46 (d, *J* = 8.4 Hz, 2H), 7.41 – 7.32 (m, 8H), 6.65 (dd, *J* = 17.6, 10.9 Hz, 1H), 5.66 (d, *J* = 17.6 Hz, 1H), 5.18 (d, *J* = 10.9 Hz, 1H), 3.12 (dt, *J* = 196.9, 6.7 Hz, 4H). ^{13}C NMR (150 MHz, $CDCl_3$) δ 169.76, 162.50, 145.73, 137.67, 136.15, 134.76, 133.55, 132.19,

128.70, 128.67, 128.66, 128.66, 128.29, 127.88, 126.81, 126.49, 119.61, 112.90, 34.16, 24.06. HRMS (ESI): calcd for $C_{26}H_{23}N_2O_2^+$ (M+H) 395.1754, found: 395.1750.

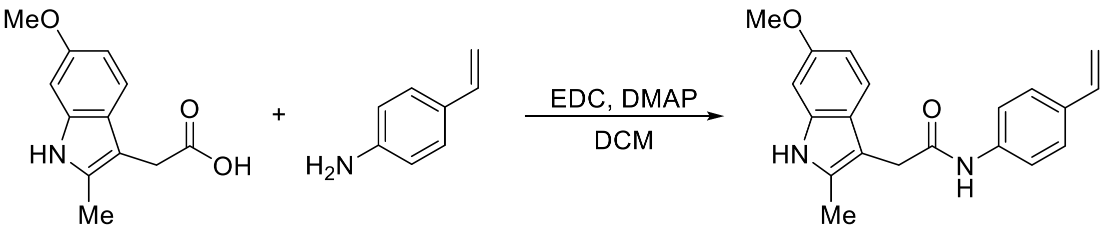

A mixture of 2-(6-methoxy-2-methyl-1H-indol-3-yl)acetic acid (219.2 mg, 1.0 mmol), 4-vinylaniline (121.5 mg, 1.02 mmol), 1-(3-dimethylaminopropyl)-3-ethylcarbodiimide hydrochloride (287.6 mg, 1.5 mmol), and DMAP (2.5 mg, 0.02 mmol) in CH_2Cl_2 (20.0 mL) was stirred at room temperature for 2.5 h. The reaction mixture was added to H_2O and extracted with CH_2Cl_2. The organic layers were washed with brine, dried over $MgSO_4$, filtered, concentrated in vacuo, and flash chromatographed (acetone/hexane = 1:2) to afford the product (215 mg, 67%).

2-(6-methoxy-2-methyl-1H-indol-3-yl)-N-(4-vinylphenyl)acetamide (S4): ^{1}H NMR (600 MHz, $CDCl_3$) δ 8.16 (s, 1H), 7.46 (s, 1H), 7.30 (q, *J* = 8.8 Hz, 4H), 7.23 (d, *J* = 8.7 Hz, 1H), 6.93 (d, *J* = 2.1 Hz, 1H), 6.84 (dd, *J* = 8.7, 2.3 Hz, 1H), 6.62 (dd, *J* = 17.6, 10.9 Hz, 1H), 5.64 (d, *J* = 17.6 Hz, 1H), 5.16 (d, *J* = 10.9 Hz, 1H), 3.81 (s, 3H), 3.79 (s, 2H), 2.41 (s, 3H). ^{13}C NMR (150 MHz, $CDCl_3$) δ 169.77, 154.66, 137.16, 136.08, 134.41, 133.79, 130.41, 128.53, 126.69, 119.92, 113.04, 111.88, 111.49, 104.12, 99.66, 55.92, 33.43, 11.84. HRMS (ESI): calcd for $C_{20}H_{21}N_2O_2^+$ (M+H) 321.1598, found: 321.1590.

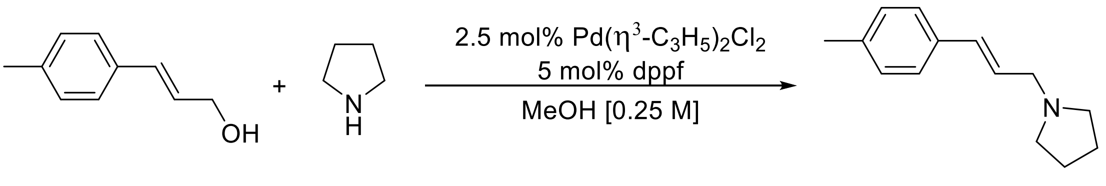

According to the previous reference,[2] a mixture of dppf (140.0 mg, 0.25 mmol) and $[Pd(\eta^3\text{-}C_3H_5)Cl]_2$ (46.0 mg, 0.125mmol) in dry MeOH (20 mL) was stirred at room temperature under a N_2 atmosphere for 60 min. (*E*)-3-(p-tolyl)prop-2-en-1-ol (5.0 mmol, 1.0 equiv) was added and the mixture was stirred for another 10 min, followed by the addition of pyrrolidine (7.5 mmol, 1.5 equiv). The reaction was monitored by GC-MS or TLC. The crude reaction mixture was concentrated by rotary evaporation and the residue was then purified by silica column chromatography (PE/EA/TEA = 10:1/0.4) to give the desired product (864 mg, 86%).

(*E*)-1-(3-(p-Tolyl)allyl)pyrrolidine (S5): ^1H NMR (600 MHz, $CDCl_3$) δ 7.27 (d, *J* = 8.1 Hz, 2H), 7.11 (d, *J* = 8.0 Hz, 2H), 6.50 (d, *J* = 15.8 Hz, 1H), 6.28 (dt, *J* = 15.8, 6.7 Hz, 1H), 3.25 (dd, *J* = 6.7, 1.1 Hz, 2H), 2.60 – 2.50 (m, 4H), 2.33 (s, 3H), 1.84 – 1.76 (m, 4H).

General procedure A (GPA) for the branch-selective pyridylation of alkenes: To a flame-dried 20 mL reaction vial was charged with $Ir(ppy)_3$ (0.002 mmol, 1.0 mol%), $MeSO_2Na$ (0.06 mmol, 30 mol%) [or sodium 4-chlorobenzylsulfinite (0.1 mmol, 50 mol%.)], 4-cyanopyridine (0.4 mmol, 2.0 equiv.), and a magnetic stir bar. A 1:1 mixture of MeCN/EtOH [0.02 M] was added. The reaction mixture was degassed by nitrogen sparging for 15 min, followed by the addition of alkenes (0.2 mmol, 1.0 equiv.) and 1,8-diazabicyclo[5.4.0]undec-7-ene (DBU) (0.6 mmol, 3.0 equiv.). The reaction mixture was then irradiated with a 90 W blue LED for 24 h at 40 ℃. The reaction mixture was concentrated in vacuo and then quenched with water, extracted with ethyl acetate. The combined organic layers were dried with $MgSO_4$, filtered and concentrated in vacuo. The crude material was purified by flash chromatography to afford the products.

General procedure B (GPB) for the branch-selective pyridylation of alkenes: To a flame-dried 20 mL reaction vial was charged with $Ir(ppy)_3$ (0.002 mmol, 1.0 mol%),

sodium 4-chlorobenzylsulfinite (0.2 mmol, 1.0 equiv.), 4-cyanopyridine (0.4 mmol, 2.0 equiv.), NH_4Cl (0.4 mmol, 2.0 equiv.) and a magnetic stir bar. A 1:1 mixture of MeCN/EtOH [0.02 M] was added. The reaction mixture was degassed by nitrogen sparging for 15 min, followed by the addition of alkenes (0.2 mmol, 1.0 equiv.). The reaction mixture was irritated with a 90 W blue LED for 2 h at 25 °C. Then MeOK (1.2 mmol, 6.0 equiv.) [or DBU (1.2 mmol, 6.0 equiv.)] was added. After stirred for another hour, the reaction mixture was concentrated in vacuo and then quenched with water, extracted with ethyl acetate. The combined organic layers were dried with $MgSO_4$, filtered and concentrated in vacuo. The crude material was purified by flash chromatography to afford the products.

Analytical Data of Compounds

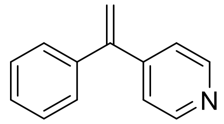

4-(1-Phenylvinyl)pyridine (14): According to the **GPA**, styrene (23.0 µL, 0.20 mmol, 1.0 equiv.), 4-cyanopyridine (41.6 mg, 0.40 mmol, 2.0 equiv.), sodium methanesulfinate (6.2 mg, 0.06 mmol, 30 mol%), $Ir(ppy)_3$ (6.6 mg, 0.01 mmol, 5 mol%), and 1,8-diazabicyclo[5.4.0]undec-7-ene (89.6 µL, 0.6 mmol, 3.0 equiv.) in MeCN-EtOH (1:1, 10 mL) were used. After 24 h, the result mixture was concentrated in vacuo and the product was isolated by flash chromatography (PE: EA= 10:1) as a pale-yellow oil (28.2 mg, 78%). ^1H NMR (400 MHz, $CDCl_3$) δ 8.57 (d, *J* = 5.5 Hz, 2H), 7.32 (dt, *J* = 7.4, 4.0 Hz, 5H), 7.24 (d, *J* = 5.5 Hz, 2H), 5.60 (d, *J* = 2.8 Hz, 2H). ^{13}C NMR (150 MHz, $CDCl_3$) δ 149.90, 148.79, 147.95, 139.78, 128.44, 128.24, 128.15, 122.78, 116.93. HRMS (ESI): calcd for $C_{13}H_{12}N^+$ (M+H) 182.0964, found 182.0963.

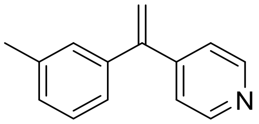

4-(1-(m-Tolyl)vinyl)pyridine (15): According to the **GPA**, 3-methylstyrene (26.2 μL, 0.20 mmol, 1.0 equiv.), 4-cyanopyridine (41.6 mg, 0.40 mmol, 2.0 equiv.), sodium methanesulfinate (6.2 mg, 0.06 mmol, 30 mol%), Ir(ppy)$_3$ (6.6 mg, 0.01 mmol, 5 mol%), and 1,8-diazabicyclo[5.4.0]undec-7-ene (89.6 μL, 0.6 mmol, 3.0 equiv.) in MeCN-EtOH (10 mL) were used. After 24 h, the result mixture was concentrated in vacuo and the product was isolated by flash chromatography (PE: EA= 10:1) as a pale-yellow oil (27.7 mg, 71%). ^{1}H NMR (600 MHz, CDCl$_3$) δ 8.60 (dd, *J* = 4.6, 1.5 Hz, 2H), 7.28 – 7.25 (m, 3H), 7.19 (d, *J* = 7.6 Hz, 1H), 7.14 – 7.09 (m, 2H), 5.60 (d, *J* = 1.2 Hz, 2H), 2.37 (s, 3H). ^{13}C NMR (150 MHz, CDCl$_3$) δ 149.39, 148.43, 147.57, 139.28, 137.58, 128.50, 128.34, 127.84, 124.81, 122.31, 116.24, 20.92. HRMS (ESI): calcd for $C_{14}H_{14}N^+$ (M+H) 196.1121, found 196.1120.

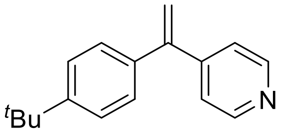

4-(1-(4-(*tert*-Butyl)phenyl)vinyl)pyridine (13): According to the **GPA**, 4-*tert*-butylstyrene (36.6 μL, 0.20 mmol, 1.0 equiv.), 4-cyanopyridine (41.6 mg, 0.40 mmol, 2.0 equiv.), sodium methanesulfinate (6.2 mg, 0.06 mmol, 30 mol%), Ir(ppy)$_3$ (6.6 mg, 0.01 mmol, 5 mol%), and 1,8-diazabicyclo[5.4.0]undec-7-ene (89.6 μL, 0.6 mmol, 3.0 equiv.) in MeCN-EtOH (10 mL) were used. After 24 h, the result mixture was concentrated in vacuo and the product was isolated by flash chromatography (PE: EA= 10:1) as a pale-yellow oil (40.8 mg, 86%). ^{1}H NMR (400 MHz, CDCl$_3$) δ 8.58 (dd, *J* = 4.7, 1.4 Hz, 2H), 7.38 (d, *J* = 8.4 Hz, 2H), 7.28 – 7.21 (m, 4H), 5.57 (d, *J* = 16.4 Hz, 2H), 1.34 (s, 9H). ^{13}C NMR (150 MHz, CDCl$_3$) δ 151.30, 149.78, 149.03, 147.69, 136.72, 127.80, 125.35, 122.89, 116.32, 34.60, 31.33. HRMS (ESI): calcd for $C_{17}H_{20}N^+$ (M+H) 238.1590, found 238.1589.

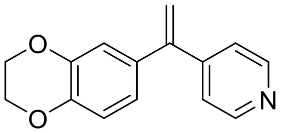

4-(1-(2,3-Dihydrobenzo[b][1,4]dioxin-6-yl)vinyl)pyridine (16): According to the **GPA**, 4-(1-(2,3-dihydrobenzo[b][1,4]dioxin-6-yl)vinyl)pyridine (32.4 mg, 0.20 mmol, 1.0 equiv.), 4-cyanopyridine (41.6 mg, 0.40 mmol, 2.0 equiv.), sodium 4-chlorobenzenesulfinate (19.8 mg, 0.10 mmol, 50 mol%), Ir(ppy)$_3$ (1.3 mg, 0.002 mmol, 1 mol%), and 1,8-diazabicyclo[5.4.0]undec-7-ene (89.6 µL, 0.6 mmol, 3.0 equiv.) in MeCN-EtOH (10 mL) were used. After 24 h, the result mixture was concentrated in vacuo and the product was isolated by flash chromatography (PE: acetone= 5:1) as a pale-yellow oil (36.8 mg, 77%). ^{1}H NMR (600 MHz, CDCl$_3$) δ 8.56 (d, *J* = 5.9 Hz, 2H), 7.23 (d, *J* = 6.0 Hz, 2H), 6.82 (dd, *J* = 14.0, 5.2 Hz, 2H), 6.77 (dd, *J* = 8.3, 2.0 Hz, 1H), 5.49 (d, *J* = 26.1 Hz, 2H), 4.26 (q, *J* = 5.5 Hz, 4H). ^{13}C NMR (150 MHz, CDCl$_3$) δ 149.83, 148.98, 147.26, 143.75, 143.29, 133.22, 122.90, 121.33, 117.16, 117.01, 115.81, 64.45, 64.35. HRMS (ESI): calcd for $C_{15}H_{14}NO_2^+$ (M+H) 240.1025, found 240.1018.

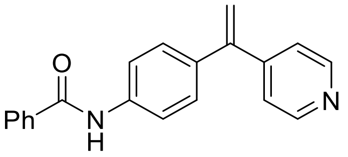

N-(4-(1-(pyridin-4-yl)vinyl)phenyl)benzamide (17): According to the **GPA**, N-(4-vinylphenyl)benzamide (44.7 mg, 0.20 mmol, 1.0 equiv.), 4-cyanopyridine (41.6 mg, 0.40 mmol, 2.0 equiv.), sodium 4-chlorobenzenesulfinate (19.8 mg, 0.10 mmol, 50 mol%), Ir(ppy)$_3$ (1.3 mg, 0.002 mmol, 1 mol%), and 1,8-diazabicyclo[5.4.0]undec-7-ene (89.6 µL, 0.6 mmol, 3.0 equiv.) in MeCN-EtOH (10 mL) were used. After 24 h, the result mixture was concentrated in vacuo and the product was isolated by flash chromatography (DCM: MeOH= 100:1) as a pale-yellow solid (36.0 mg, 60%). ^{1}H NMR (600 MHz, CDCl$_3$) δ 8.58 (d, *J* = 5.8 Hz, 2H), 8.17 (s, 1H), 7.90 (d, *J* = 7.4 Hz, 2H), 7.67 (d, *J* = 8.5 Hz, 2H), 7.57 (t, *J* = 7.4 Hz, 1H), 7.49 (t, *J* = 7.6 Hz, 2H), 7.31 (d, *J* = 8.5 Hz, 2H), 7.28 – 7.26 (m, 2H), 5.60 (d, *J* = 17.8 Hz, 2H). ^{13}C NMR (150 MHz, CDCl$_3$) δ 165.85, 149.89, 148.84, 147.26, 138.06, 135.89, 134.81, 131.99, 128.86, 128.84, 127.08, 122.88, 120.12, 116.56. HRMS (ESI): calcd for $C_{20}H_{17}N_2O^+$ (M+H) 301.1335, found 301.1353.

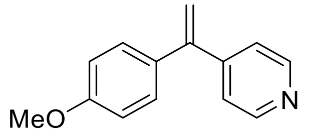

4-(1-Phenylvinyl)pyridine (18): According to the **GPA**, 4-methoxystyrene (26.6 μL, 0.20 mmol, 1.0 equiv.), 4-cyanopyridine (41.6 mg, 0.40 mmol, 2.0 equiv.), sodium methanesulfinate (6.2 mg, 0.06 mmol, 30 mol%), Ir(ppy)$_3$ (6.6 mg, 0.01 mmol, 5 mol%), and 1,8-diazabicyclo[5.4.0]undec-7-ene (89.6 μL, 0.6 mmol, 3.0 equiv.) in MeCN-EtOH (10 mL) were used. After 24 h, the result mixture was concentrated in vacuo and the product was isolated by flash chromatography (PE: EA= 10:1) as a pale-yellow oil (30.0 mg, 70%). ^{1}H NMR (400 MHz, CDCl$_3$) δ 8.57 (dd, *J* = 4.5, 1.6 Hz, 2H), 7.26 – 7.20 (m, 4H), 6.88 (d, *J* = 8.8 Hz, 2H), 5.51 (d, *J* = 14.5 Hz, 2H), 3.83 (s, 3H). ^{13}C NMR (150 MHz, CDCl$_3$) δ 159.67, 149.81, 149.24, 147.40, 132.21, 129.32, 122.90, 115.56, 113.79, 55.33. HRMS (ESI): calcd for $C_{14}H_{14}NO^+$ (M+H) 212.1062, found 212.1069.

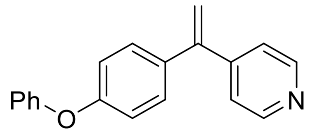

4-(1-(4-Phenoxyphenyl)vinyl)pyridine (19): According to the **GPA**, styrene (39.3 mg, 0.20 mmol, 1.0 equiv.), 4-cyanopyridine (41.6 mg, 0.40 mmol, 2.0 equiv.), sodium methanesulfinate (6.2 mg, 0.06 mmol, 30 mol%), Ir(ppy)$_3$ (6.6 mg, 0.01 mmol, 5 mol%), and 1,8-diazabicyclo[5.4.0]undec-7-ene (89.6 μL, 0.6 mmol, 3.0 equiv.) in MeCN-EtOH (10 mL) were used. After 24 h, the result mixture was concentrated in vacuo and the product was isolated by flash chromatography (PE: EA= 10:1) as a pale-yellow oil (36.6 mg, 67%). ^{1}H NMR (400 MHz, CDCl$_3$) δ 8.60 (dd, *J* = 4.6, 1.5 Hz, 2H), 7.37 (t, *J* = 7.9 Hz, 2H), 7.30 – 7.26 (m, 4H), 7.15 (t, *J* = 7.4 Hz, 1H), 7.07 (d, *J* = 7.9 Hz, 2H), 7.01 – 6.97 (m, 2H), 5.57 (d, *J* = 7.5 Hz, 2H). ^{13}C NMR (100 MHz, CDCl$_3$) δ 157.61, 156.72, 149.96, 148.90, 147.26, 134.58, 129.88, 129.57, 123.70, 122.85, 119.29, 118.39, 116.35. HRMS (ESI): calcd for $C_{19}H_{16}NO^+$ (M+H) 274.1226, found 274.1225.

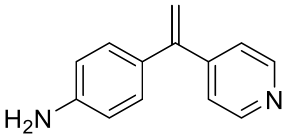

4-(1-(pyridin-4-yl)vinyl)aniline (20): According to the **GPB**, 4-vinylaniline (23.8 mg, 0.20 mmol, 1.0 equiv.), 4-cyanopyridine (41.6 mg, 0.40 mmol, 2.0 equiv.), sodium 4-chlorobenzenesulfinate (39.6 mg, 0.20 mmol, 1.0 equiv.), Ir(ppy)$_3$ (1.3 mg, 0.002 mmol, 1 mol%), NH_4Cl (21.4 mg, 0.4 mmol, 2.0 equiv.) and MeOK (70.0 mg, 1.0 mmol, 5.0 equiv.) in MeCN-EtOH (10 mL) were used. The result mixture was concentrated in vacuo and the product was isolated by flash chromatography (PE: EA= 5:1) as a brown oil (24.3 mg, 62%). ^{1}H NMR (600 MHz, $CDCl_3$) δ 8.57 (d, *J* = 5.8 Hz, 2H), 7.26 (d, *J* = 5.9 Hz, 2H), 7.11 (d, *J* = 8.4 Hz, 2H), 6.66 (d, *J* = 8.4 Hz, 2H), 5.46 (d, *J* = 57.0 Hz, 2H), 3.80 (s, 2H). ^{13}C NMR (150 MHz, $CDCl_3$) δ 149.72, 149.65, 147.67, 146.64, 129.86, 129.18, 123.07, 114.73, 114.36. HRMS (ESI): calcd for $C_{13}H_{13}N_2^+$ (M+H) 197.1073, found 197.1071.

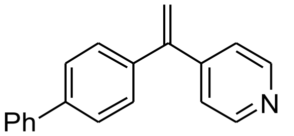

4-(1-([1,1'-Biphenyl]-4-yl)vinyl)pyridine (21): According to the **GPA**, 4-phenylstyrene (36.0 mg, 0.20 mmol, 1.0 equiv.), 4-cyanopyridine (41.6 mg, 0.40 mmol, 2.0 equiv.), sodium 4-chlorobenzenesulfinate (19.8 mg, 0.10 mmol, 50 mol%), Ir(ppy)$_3$ (1.3 mg, 0.002 mmol, 1 mol%), and 1,8-diazabicyclo[5.4.0]undec-7-ene (89.6 μL, 0.6 mmol, 3.0 equiv.) in MeCN-EtOH (10 mL) were used. After 24 h, the result mixture was concentrated in vacuo and the product was isolated by flash chromatography (PE: EA= 10:1) as a pale-yellow solid (42.1 mg, 82%). ^{1}H NMR (400 MHz, $CDCl_3$) δ 8.61 (d, *J* = 6.1 Hz, 2H), 7.61 (t, *J* = 8.5 Hz, 4H), 7.46 (t, *J* = 7.6 Hz, 2H), 7.41 – 7.34 (m, 3H), 7.29 (dd, *J* = 4.5, 1.6 Hz, 2H), 5.65 (d, *J* = 17.6 Hz, 2H). ^{13}C NMR (100 MHz, $CDCl_3$) δ 149.98, 148.82, 147.59, 141.14, 140.49, 138.68, 128.89, 128.58, 127.57,

127.17, 127.07, 122.91, 116.91. HRMS (ESI): calcd for $C_{19}H_{16}N^+$ (M+H) 258.1277, found 258.1277.

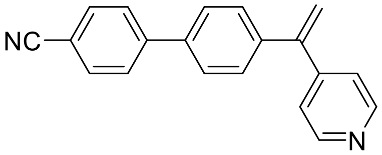

4'-(1-(Pyridin-4-yl)vinyl)-[1,1'-biphenyl]-4-carbonitrile (22): According to the **GPA**, 4-vinylphenyl 4-methylbenzenesulfonate (41.1 mg, 0.20 mmol, 1.0 equiv.), 4-cyanopyridine (41.6 mg, 0.40 mmol, 2.0 equiv.), sodium 4-chlorobenzenesulfinate (19.8 mg, 0.10 mmol, 50 mol%), Ir(ppy)$_3$ (1.3 mg, 0.002 mmol, 1 mol%), and 1,8-diazabicyclo[5.4.0]undec-7-ene (89.6 µL, 0.6 mmol, 3.0 equiv.) in MeCN-EtOH (10 mL) were used. After 24 h, the result mixture was concentrated in vacuo and the product was isolated by flash chromatography (PE: EA= 5:1) as a colorless crystal (37.8 mg, 67%). ^{1}H NMR (600 MHz, $CDCl_3$) δ 8.63 (d, J = 5.7 Hz, 2H), 7.75 (dd, J = 20.7, 8.3 Hz, 4H), 7.62 (d, J = 8.1 Hz, 2H), 7.44 (d, J = 8.1 Hz, 2H), 7.29 (d, J = 6.1 Hz, 2H), 5.70 (d, J = 11.6 Hz, 2H). ^{13}C NMR (150 MHz, $CDCl_3$) δ 150.02, 148.47, 147.23, 144.90, 140.14, 138.98, 132.69, 128.88, 127.61, 127.30, 122.79, 118.85, 117.56, 111.17. HRMS (ESI): calcd for $C_{20}H_{15}N_2^+$ (M+H) 283.1235, found 283.1228.

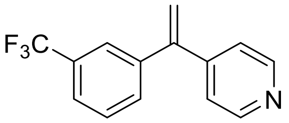

4-(1-(3-(Trifluoromethyl)phenyl)vinyl)pyridine (23): According to the **GPB**, 3-trifluoromethylstyrene (34.4 mg, 0.20 mmol, 1.0 equiv.), 4-cyanopyridine (41.6 mg, 0.40 mmol, 2.0 equiv.), sodium 4-chlorobenzenesulfinate (39.8 mg, 0.20 mmol, 1.0 equiv.), Ir(ppy)$_3$ (1.3 mg, 0.002 mmol, 1 mol%), and 1,8-diazabicyclo[5.4.0]undec-7-ene (89.6 µL, 0.6 mmol, 3.0 equiv.) in MeCN-EtOH (10 mL) were used. After 24 h, the result mixture was concentrated in vacuo and the product was isolated by flash chromatography (PE: EA= 10:1) as a pale-yellow oil (29.9 mg, 60%). ^{1}H NMR (400 MHz, $CDCl_3$) δ 8.61 (dd, J = 4.5, 1.5 Hz, 2H), 7.62 (d, J = 7.3 Hz, 1H), 7.57 (s, 1H), 7.52 – 7.44 (m, 2H), 7.22 (dd, J = 4.5, 1.5 Hz, 2H), 5.69 (d, J = 22.1 Hz, 2H). ^{13}C NMR

(100 MHz, $CDCl_3$) δ 150.13, 147.92, 146.80, 140.61, 131.47, 131.05 (q, *J* = 32.3 Hz), 129.01, 123.95 (q, *J* = 276.7 Hz), 125.01 (q, *J* = 3.8 Hz), 124.85 (q, *J* = 3.8 Hz), 122.58, 118.40. HRMS (ESI): calcd for $C_{14}H_{11}F_3N^+$ (M+H) 250.0838, found 250.0837.

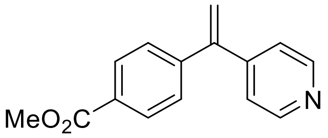

Methyl 4-(1-(pyridin-4-yl)vinyl)benzoate (24): According to the **GPB**, methyl 4-vinylbenzoate (44.0 mg, 0.20 mmol, 1.0 equiv.), 4-cyanopyridine (41.6 mg, 0.40 mmol, 2.0 equiv.), sodium 4-chlorobenzenesulfinate (39.8 mg, 0.20 mmol, 1.0 equiv.), $Ir(ppy)_3$ (1.3 mg, 0.002 mmol, 1 mol%), and 1,8-diazabicyclo[5.4.0]undec-7-ene (89.6 μL, 0.6 mmol, 3.0 equiv.) in MeCN-MeOH (10 mL) were used. After 24 h, the result mixture was concentrated in vacuo and the product was isolated by flash chromatography (PE: EA= 5:1) as a pale-yellow oil (29.6 mg, 62%). ^{1}H NMR (400 MHz, $CDCl_3$) δ 8.62 (d, *J* = 6.0 Hz, 2H), 8.03 (d, *J* = 8.3 Hz, 2H), 7.34 (dd, *J* = 12.9, 7.2 Hz, 4H), 5.75 (d, *J* = 7.8 Hz, 2H), 3.93 (s, 3H). ^{13}C NMR (100 MHz, $CDCl_3$) δ 166.72, 150.05, 148.12, 147.20, 144.24, 129.91, 129.79, 128.14, 122.69, 118.46, 52.21. HRMS (ESI): calcd for $C_{15}H_{14}NO_2^+$ (M+H) 240.1025, found 240.1030.

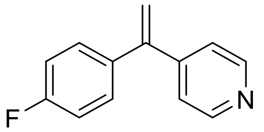

4-(1-(4-Fluorophenyl)vinyl)pyridine (25): According to the **GPA**, 4-fluorostyrene (23.8 μL, 0.20 mmol, 1.0 equiv.), 4-cyanopyridine (41.6 mg, 0.40 mmol, 2.0 equiv.), sodium methanesulfinate (6.2 mg, 0.06 mmol, 30 mol%), $Ir(ppy)_3$ (6.6 mg, 0.01 mmol, 5 mol%), and 1,8-diazabicyclo[5.4.0]undec-7-ene (89.6 μL, 0.6 mmol, 3.0 equiv.) in MeCN-EtOH (10 mL) were used. After 24 h, the result mixture was concentrated in vacuo and the product was isolated by flash chromatography (PE: EA= 10:1) as a pale-yellow oil (26.7 mg, 67%). ^{1}H NMR (400 MHz, $CDCl_3$) δ 8.58 (d, *J* = 6.1 Hz, 2H), 7.26 (dd, *J* = 8.7, 5.5 Hz, 2H), 7.21 (dd, *J* = 4.5, 1.6 Hz, 2H), 7.04 (t, *J* = 8.7 Hz, 2H), 5.57 (d, *J* = 13.1 Hz, 2H). ^{13}C NMR (150 MHz, $CDCl_3$) δ 162.74 (d, *J* = 247.8 Hz),

149.95, 148.61, 146.92, 135.84 (d, J = 3.3 Hz), 129.85 (d, J = 8.2 Hz), 122.69, 116.95, 115.40 (d, J = 21.5 Hz). HRMS (ESI): calcd for $C_{13}H_{11}FN^+$ (M+H) 200.0876, found 200.0891.

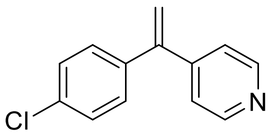

4-(1-(4-Chlorophenyl)vinyl)pyridine (26): According to the **GPA**, 4-chlorostyrene (24.0 μL, 0.20 mmol, 1.0 equiv.), 4-cyanopyridine (41.6 mg, 0.40 mmol, 2.0 equiv.), sodium methanesulfinate (6.2 mg, 0.06 mmol, 30 mol%), Ir(ppy)$_3$ (6.6 mg, 0.01 mmol, 5 mol%), and 1,8-diazabicyclo[5.4.0]undec-7-ene (89.6 μL, 0.6 mmol, 3.0 equiv.) in MeCN-EtOH (10 mL) were used. After 24 h, the result mixture was concentrated in vacuo and the product was isolated by flash chromatography (PE: EA= 10:1) as a pale-yellow oil (25.9 mg, 60%). ^{1}H NMR (400 MHz, $CDCl_3$) δ 8.58 (dd, J = 4.5, 1.5 Hz, 2H), 7.32 (d, J = 8.5 Hz, 2H), 7.22 (dt, J = 5.8, 1.9 Hz, 4H), 5.60 (d, J = 10.4 Hz, 2H). ^{13}C NMR (100 MHz, $CDCl_3$) δ 150.02, 148.33, 146.89, 138.23, 134.25, 129.46, 128.68, 122.68, 117.38. HRMS (ESI): calcd for $C_{13}H_{11}ClN^+$ (M+H) 216.0575, found 216.0573.

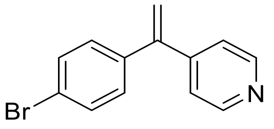

4-(1-(4-Bromophenyl)vinyl)pyridine (27): According to the **GPA**, 4-bromostyrene (27.2 μL, 0.20 mmol, 1.0 equiv.), 4-cyanopyridine (41.6 mg, 0.40 mmol, 2.0 equiv.), sodium methanesulfinate (6.2 mg, 0.06 mmol, 30 mol%), Ir(ppy)$_3$ (6.6 mg, 0.01 mmol, 5 mol%), and 1,8-diazabicyclo[5.4.0]undec-7-ene (89.6 μL, 0.6 mmol, 3.0 equiv.) in MeCN-EtOH (10 mL) were used. After 24 h, the result mixture was concentrated in vacuo and the product was isolated by flash chromatography (PE: EA= 10:1) as a pale-yellow oil (36.9 mg, 71%). ^{1}H NMR (400 MHz, $CDCl_3$) δ 8.57 (dd, J = 4.5, 1.6 Hz, 2H), 7.47 (d, J = 8.5 Hz, 2H), 7.20 (dd, J = 4.5, 1.6 Hz, 2H), 7.18 – 7.14 (m, 2H), 5.60 (d, J = 10.5 Hz, 2H). ^{13}C NMR (100 MHz, $CDCl_3$) δ 150.00, 148.25, 146.93, 138.69,

131.65, 129.78, 122.69, 122.43, 117.45. HRMS (ESI): calcd for $C_{13}H_{11}BrN^+$ (M+H) 260.0075, found 260.0093.

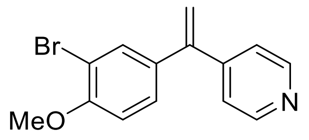

4-(1-(3-Bromo-4-methoxyphenyl)vinyl)pyridine (28): According to the **GPA**, 3-bromo-4-methoxystyrene (42.6 mg, 0.20 mmol, 1.0 equiv.), 4-cyanopyridine (41.6 mg, 0.40 mmol, 2.0 equiv.), sodium methanesulfinate (6.2 mg, 0.06 mmol, 30 mol%), Ir(ppy)$_3$ (6.6 mg, 0.01 mmol, 5 mol%), and 1,8-diazabicyclo[5.4.0]undec-7-ene (89.6 μL, 0.6 mmol, 3.0 equiv.) in MeCN-EtOH (10 mL) were used. After 24 h, the result mixture was concentrated in vacuo and the product was isolated by flash chromatography (PE: EA= 10:1) as a colorless crystal (42.3 mg, 73%). ^{1}H NMR (400 MHz, $CDCl_3$) δ 8.57 (d, J = 5.8 Hz, 2H), 7.50 (d, J = 2.2 Hz, 1H), 7.21 (d, J = 6.0 Hz, 2H), 7.17 (dd, J = 8.5, 2.2 Hz, 1H), 6.86 (d, J = 8.5 Hz, 1H), 5.53 (d, J = 1.8 Hz, 2H), 3.91 (s, 3H). ^{13}C NMR (100 MHz, $CDCl_3$) δ 155.89, 149.97, 148.56, 146.27, 133.63, 132.89, 128.33, 122.76, 116.60, 111.73, 111.60, 56.34. HRMS (ESI): calcd for $C_{14}H_{13}BrNO^+$ (M+H) 290.0175, found 290.0196.

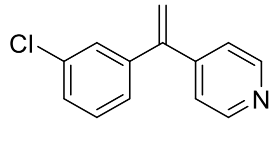

4-(1-(3-Chlorophenyl)vinyl)pyridine (29): According to the **GPA**, 3-chlorostyrene (54.9 mg, 0.20 mmol, 1.0 equiv.), 4-cyanopyridine (25.4 μL, 0.40 mmol, 2.0 equiv.), sodium methanesulfinate (6.2 mg, 0.06 mmol, 30 mol%), Ir(ppy)$_3$ (6.6 mg, 0.01 mmol, 5 mol%), and 1,8-diazabicyclo[5.4.0]undec-7-ene (89.6 μL, 0.6 mmol, 3.0 equiv.) in MeCN-EtOH (10 mL) were used. After 24 h, the result mixture was concentrated in vacuo and the product was isolated by flash chromatography (PE: EA= 10:1) as a pale-yellow oil (29.8 mg, 69%). ^{1}H NMR (600 MHz, $CDCl_3$) δ 8.59 (dd, J = 4.5, 1.6 Hz, 2H), 7.34 – 7.31 (m, 1H), 7.29 (dd, J = 4.7, 2.8 Hz, 2H), 7.21 (dd, J = 4.5, 1.6 Hz, 2H),

7.17 – 7.14 (m, 1H), 5.63 (d, *J* = 20.9 Hz, 2H). ^{13}C NMR (150 MHz, $CDCl_3$) δ 150.05, 148.06, 146.79, 141.62, 134.45, 129.72, 128.33, 128.22, 126.34, 122.63, 117.93. HRMS (ESI): calcd for $C_{13}H_{11}ClN^+$ (M+H) 216.0575, found 216.0574.

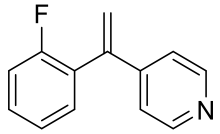

4-(1-(2-Fluorophenyl)vinyl)pyridine (30): According to the **GPA**, 4-vinylphenyl 2-fluorostyrene (23.8 μL, 0.20 mmol, 1.0 equiv.), 4-cyanopyridine (41.6 mg, 0.40 mmol, 2.0 equiv.), sodium 4-chlorobenzenesulfinate (19.8 mg, 0.10 mmol, 50 mol%), $Ir(ppy)_3$ (1.3 mg, 0.002 mmol, 1 mol%), and 1,8-diazabicyclo[5.4.0]undec-7-ene (89.6 μL, 0.6 mmol, 3.0 equiv.) in MeCN-EtOH (10 mL) were used. After 24 h, the result mixture was concentrated in vacuo and the product was isolated by flash chromatography (PE: EA= 10:1) as a pale-yellow oil (21.5 mg, 54%). ^{1}H NMR (400 MHz, $CDCl_3$) δ 8.56 (d, *J* = 4.9 Hz, 2H), 7.36 (dd, *J* = 13.1, 6.6 Hz, 1H), 7.28 (t, *J* = 6.9 Hz, 1H), 7.18 (dd, *J* = 13.8, 6.2 Hz, 3H), 7.09 (t, *J* = 9.2 Hz, 1H), 5.75 (d, *J* = 126.0 Hz, 2H). ^{13}C NMR (150 MHz, $CDCl_3$) δ 159.98 (d, *J* = 248.9 Hz), 149.98, 147.70, 142.35, 131.33 (d, *J* = 3.4 Hz), 130.06 (d, *J* = 8.2 Hz), 127.61 (d, *J* = 14.4 Hz), 124.27 (d, *J* = 3.6 Hz), 121.27, 120.08 (d, *J* = 1.7 Hz), 115.95 (d, *J* = 22.0 Hz). HRMS (ESI): calcd for $C_{13}H_{11}FN^+$ (M+H) 200.0870, found 200.0892.

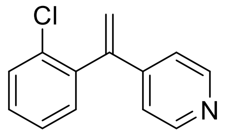

4-(1-(2-Chlrophenyl)vinyl)pyridine (31): According to the **GPA**, 4-vinylphenyl 2-chlorostyrene (23.8 μL, 0.20 mmol, 1.0 equiv.), 4-cyanopyridine (41.6 mg, 0.40 mmol, 2.0 equiv.), sodium 4-chlorobenzenesulfinate (19.8 mg, 0.10 mmol, 50 mol%), $Ir(ppy)_3$ (1.3 mg, 0.002 mmol, 1 mol%), and 1,8-diazabicyclo[5.4.0]undec-7-ene (89.6 μL, 0.6 mmol, 3.0 equiv.) in MeCN-EtOH (10 mL) were used. After 24 h, the result mixture was concentrated in vacuo and the product was isolated by flash chromatography (PE:

EA= 10:1) as a pale-yellow oil (23.0 mg, 53%). ^{1}H NMR (400 MHz, $CDCl_3$) δ 8.53 (dd, *J* = 4.6, 1.6 Hz, 1H), 7.44 – 7.36 (m, 1H), 7.36 – 7.28 (m, 1H), 7.14 (dd, *J* = 4.6, 1.6 Hz, 1H), 5.74 (d, *J* = 209.8 Hz, 1H). ^{13}C NMR (100 MHz, $CDCl_3$) δ 149.99, 146.99, 145.61, 138.99, 133.23, 131.57, 129.88, 129.50, 126.98, 120.90, 119.72. HRMS (ESI): calcd for $C_{13}H_{11}ClN^+$ (M+H) 216.0575, found 216.0572.

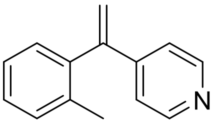

4-(1-(o-Tolyl)vinyl)pyridine (32): According to the **GPA**, 2-methylstyrene (23.0 μL, 0.20 mmol, 1.0 equiv.), 4-cyanopyridine (41.6 mg, 0.40 mmol, 2.0 equiv.), sodium 4-chlorobenzenesulfinate (19.8 mg, 0.10 mmol, 50 mol%), Ir(ppy)$_3$ (1.3 mg, 0.002 mmol, 1 mol%), and 1,8-diazabicyclo[5.4.0]undec-7-ene (89.6 μL, 0.6 mmol, 3.0 equiv.) in MeCN-EtOH (10 mL) were used. After 24 h, the result mixture was concentrated in vacuo and the product was isolated by flash chromatography (PE: EA= 20:1) as a pale-yellow oil (26.5 mg, 68%). ^{1}H NMR (600 MHz, $CDCl_3$) δ 8.52 (dd, *J* = 4.6, 1.5 Hz, 2H), 7.28 (td, *J* = 7.4, 1.3 Hz, 1H), 7.23 (t, *J* = 7.3 Hz, 1H), 7.22 – 7.18 (m, 2H), 7.14 (dd, *J* = 4.6, 1.6 Hz, 2H), 5.68 (dd, *J* = 341.0, 0.7 Hz, 2H), 2.03 (s, 3H). ^{13}C NMR (100 MHz, $CDCl_3$) δ 150.06, 147.68, 147.42, 139.82, 135.96, 130.27, 129.99, 128.10, 125.95, 120.90, 118.31, 20.05. HRMS (ESI): calcd for $C_{14}H_{13}N^+$ (M+H) 196.1121, found 196.1120.

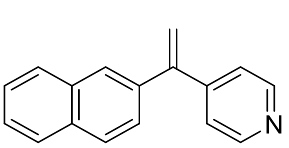

4-(1-(Naphthalen-2-yl)vinyl)pyridine (33): According to the **GPB**, 2-vinylnaphthalene (30.8 mg, 0.20 mmol, 1.0 equiv.), 4-cyanopyridine (41.6 mg, 0.40 mmol, 2.0 equiv.), sodium 4-chlorobenzenesulfinate (19.8 mg, 0.10 mmol, 50 mol%), Ir(ppy)$_3$ (1.3 mg, 0.002 mmol, 1 mol%), and 1,8-diazabicyclo[5.4.0]undec-7-ene (89.6 μL, 0.6 mmol, 3.0 equiv.) in MeCN-EtOH (10 mL) were used. After 24 h, the result

mixture was concentrated in vacuo and the product was isolated by flash chromatography (PE: EA= 10:1) as a pale-yellow oil (36.0 mg, 78%). ^{1}H NMR (400 MHz, $CDCl_3$) δ 8.61 (dd, J = 4.5, 1.6 Hz, 2H), 7.83 (tt, J = 9.5, 3.5 Hz, 3H), 7.74 (s, 1H), 7.53 – 7.47 (m, 2H), 7.44 (dd, J = 8.5, 1.8 Hz, 1H), 7.29 (dd, J = 4.5, 1.6 Hz, 2H), 5.71 (d, J = 17.7 Hz, 2H). ^{13}C NMR (100 MHz, $CDCl_3$) δ 149.99, 148.89, 147.96, 137.15, 133.25, 133.12, 128.21, 128.13, 127.68, 127.42, 126.47, 126.43, 125.94, 122.95, 117.39. HRMS (ESI): calcd for $C_{17}H_{14}N^+$ (M+H) 232.1121, found 232.1120.

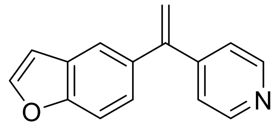

4-(1-(Benzofuran-5-yl)vinyl)pyridine (34): According to the **GPA**, 5-vinylbenzofuran (28.8 mg, 0.20 mmol, 1.0 equiv.), 4-cyanopyridine (41.6 mg, 0.40 mmol, 2.0 equiv.), sodium methanesulfinate (6.2 mg, 0.06 mmol, 30 mol%), Ir(ppy)$_3$ (6.6 mg, 0.01 mmol, 5 mol%), and 1,8-diazabicyclo[5.4.0]undec-7-ene (89.6 μL, 0.6 mmol, 3.0 equiv.) in MeCN-EtOH (10 mL) were used. After 24 h, the result mixture was concentrated in vacuo and the product was isolated by flash chromatography (PE: EA= 10:1) as a pale-yellow oil (50.0 mg, 76%). ^{1}H NMR (400 MHz, $CDCl_3$) δ 8.58 (d, J = 5.7 Hz, 2H), 7.64 (d, J = 1.0 Hz, 1H), 7.49 (dd, J = 14.2, 4.8 Hz, 2H), 7.24 (t, J = 7.8 Hz, 3H), 6.74 (s, 1H), 5.60 (d, J = 5.6 Hz, 2H). ^{13}C NMR (100 MHz, $CDCl_3$) δ 154.85, 149.88, 149.28, 148.12, 145.76, 134.89, 127.60, 124.75, 122.84, 121.04, 116.66, 111.28, 106.72. HRMS (ESI): calcd for $C_{15}H_{12}NO^+$ (M+H) 222.0905, found 222.0912.

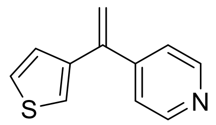

4-(1-(Thiophen-3-yl)vinyl)pyridine (35): According to the **GPA**, 3-vinylthiophene (22.0 mg, 0.20 mmol, 1.0 equiv.), 4-cyanopyridine (41.6 mg, 0.40 mmol, 2.0 equiv.), sodium methanesulfinate (6.2 mg, 0.06 mmol, 30 mol%), Ir(ppy)$_3$ (6.6 mg, 0.01 mmol,

5 mol%), and 1,8-diazabicyclo[5.4.0]undec-7-ene (89.6 μL, 0.6 mmol, 3.0 equiv.) in MeCN-EtOH (10 mL) were used. After 24 h, the result mixture was concentrated in vacuo and the product was isolated by flash chromatography (PE: EA= 10:1) as a pale-yellow oil (23.6 mg, 63%). ^{1}H NMR (400 MHz, $CDCl_3$) δ 8.60 (d, J = 6.0 Hz, 2H), 7.33 (dd, J = 5.0, 3.0 Hz, 1H), 7.30 (dd, J = 4.5, 1.5 Hz, 2H), 7.16 – 7.10 (m, 2H), 5.55 (d, J = 73.2 Hz, 2H). ^{13}C NMR (100 MHz, $CDCl_3$) δ 149.90, 148.95, 142.53, 140.75, 126.89, 126.05, 123.65, 122.76, 115.80. HRMS (ESI): calcd for $C_{11}H_{10}NS^+$ (M+H) 188.0528, found 188.0550.

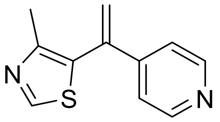

4-methyl-5-(1-(pyridin-4-yl)vinyl)thiazole (36): According to the **GPB**, 4-methyl-5-vinylthiazole (25.0 mg, 0.20 mmol, 1.0 equiv.), 4-cyanopyridine (41.6 mg, 0.40 mmol, 2.0 equiv.), sodium 4-chlorobenzenesulfinate (39.6 mg, 0.20 mmol, 1.0 equiv.), $Ir(ppy)_3$ (1.3 mg, 0.002 mmol, 1 mol%), NH_4Cl (21.4 mg, 0.4 mmol, 2.0 equiv.) and MeOK (70.0 mg, 1.0 mmol, 5.0 equiv.) in MeCN-EtOH (10 mL) were used. The result mixture was concentrated in vacuo and the product was isolated by flash chromatography (PE: EA= 5:1) as a yellow oil (22.6 mg, 56%). ^{1}H NMR (600 MHz, $CDCl_3$) δ 8.70 (s, 1H), 8.58 (d, J = 6.0 Hz, 2H), 7.21 (dd, J = 4.6, 1.5 Hz, 2H), 5.77 (d, J = 200.0 Hz, 2H), 2.25 (s, 3H). ^{13}C NMR (150 MHz, $CDCl_3$) δ 151.26, 151.03, 150.23, 147.27, 137.91, 129.46, 121.89, 121.41, 16.28. HRMS (ESI): calcd for $C_{11}H_{11}N_2S^+$ (M+H) 203.0637, found 203.0634.

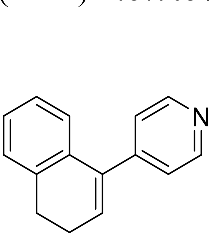

4-(3,4-Dihydronaphthalen-1-yl)pyridine (37): According to the **GPB**, 1,2-dihydronaphthalene (26.0 mg, 0.20 mmol, 1.0 equiv.), 4-cyanopyridine (41.6 mg, 0.40 mmol, 2.0 equiv.), sodium 4-chlorobenzenesulfinate (39.8 mg, 0.20 mmol, 1.0 equiv.),

Ir(ppy)$_3$ (1.3 mg, 0.002 mmol, 1 mol%), and MeOK (70.0 mg, 1.0 mmol, 5.0 equiv.) in MeCN-EtOH (10 mL) were used. After 24 h, the result mixture was concentrated in vacuo and the product was isolated by flash chromatography (PE: EA= 10:1) as a pale-yellow oil (31.1 mg, 75%). ^{1}H NMR (400 MHz, CDCl$_3$) δ 8.61 (d, *J* = 4.4 Hz, 2H), 7.28 (d, *J* = 5.7 Hz, 2H), 7.23 – 7.17 (m, 2H), 7.16 – 7.10 (m, 1H), 6.96 (d, *J* = 7.5 Hz, 1H), 6.20 (t, *J* = 4.7 Hz, 1H), 2.86 (t, *J* = 7.9 Hz, 2H), 2.43 (td, *J* = 7.9, 4.8 Hz, 2H). ^{13}C NMR (100 MHz, CDCl$_3$) δ 149.81, 148.58, 137.98, 136.74, 133.64, 129.82, 127.86, 127.58, 126.43, 125.00, 123.59, 28.03, 23.51. HRMS (ESI): calcd for $C_{15}H_{14}N^+$ (M+H) 208.1126, found 208.1130.

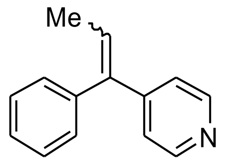

4-(1-Phenylprop-1-en-1-yl)pyridine (38): According to the **GPB**, β-methylstyrene (23.6 mg, 0.20 mmol, 1.0 equiv.), 4-cyanopyridine (41.6 mg, 0.40 mmol, 2.0 equiv.), sodium 4-chlorobenzenesulfinate (19.8 mg, 0.10 mmol, 50 mol%), Ir(ppy)$_3$ (1.3 mg, 0.002 mmol, 1 mol%), and 1,8-diazabicyclo[5.4.0]undec-7-ene (89.6 μL, 0.6 mmol, 3.0 equiv.) in MeCN-EtOH (10 mL) were used. After 24 h, the result mixture was concentrated in vacuo and the product was isolated by flash chromatography (PE: EA= 20:1) as a pale-yellow oil (26.9 mg, 69%, 1:1 mixture of two isomers). ^{1}H NMR (400 MHz, CDCl$_3$) δ 8.62 (dd, *J* = 4.4, 1.6 Hz, 2H), 7.26 (dq, *J* = 6.4, 4.7 Hz, 3H), 7.19 – 7.15 (m, 2H), 7.12 (dd, *J* = 4.4, 1.6 Hz, 2H), 6.25 (q, *J* = 7.1 Hz, 1H), 1.78 (d, *J* = 7.1 Hz, 3H). ^{13}C NMR (100 MHz, CDCl$_3$) δ 149.86, 148.19, 141.41, 140.28, 128.32, 127.27, 127.16, 125.93, 125.10, 15.67. ^{1}H NMR (400 MHz, CDCl$_3$) δ 8.46 (dd, *J* = 4.6, 1.6 Hz, 2H), 7.43 – 7.32 (m, 3H), 7.17 – 7.12 (m, 2H), 7.09 (dd, *J* = 4.6, 1.6 Hz, 2H), 6.41 (q, *J* = 7.0 Hz, 1H), 1.78 (d, *J* = 7.1 Hz, 3H). ^{13}C NMR (100 MHz, CDCl$_3$) δ 149.75, 149.70, 140.46, 138.26, 129.92, 128.48, 127.86, 127.42, 121.53, 15.82. HRMS (ESI): calcd for $C_{14}H_{14}N^+$ (M+H) 196.1121, found 196.1120.

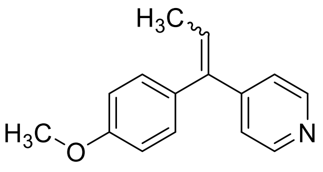

4-(1-(4-methoxyphenyl)prop-1-en-1-yl)pyridine (39): According to the **GPB**, (E)-1-methoxy-4-(prop-1-en-1-yl)benzene (29.6 mg, 0.20 mmol, 1.0 equiv.), 4-cyanopyridine (41.6 mg, 0.40 mmol, 2.0 equiv.), sodium 4-chlorobenzenesulfinate (39.6 mg, 0.20 mmol, 1.0 equiv.), Ir(ppy)$_3$ (1.3 mg, 0.002 mmol, 1 mol%), NH_4Cl (21.4 mg, 0.4 mmol, 2.0 equiv.) and MeOK (70.0 mg, 1.0 mmol, 5.0 equiv.) in MeCN-EtOH (10 mL) were used. The result mixture was concentrated in vacuo and the product was isolated by flash chromatography (PE: EA= 20:1) as a pale-yellow oil (33.3 mg, 74%, 1:1 mixture of two isomers). ^{1}H NMR (600 MHz, $CDCl_3$) δ 8.46 (d, J = 5.4 Hz, 1.4H), 7.13 (dd, J = 4.3, 1.8 Hz, 1.4H), 7.07 – 7.05 (m, 1.4H), 6.95 – 6.92 (m, 1.4H), 6.38 (q, J = 7.0 Hz, 0.7H), 3.84 (s, 2.1H), 1.80 (d, J = 7.0 Hz, 2.1H). ^{13}C NMR (150 MHz, $CDCl_3$) δ 158.89, 150.86, 149.04, 139.90, 131.08, 130.22, 128.27, 121.81, 113.90, 55.27, 15.92. ^{1}H NMR (600 MHz, $CDCl_3$) δ 8.61 (d, J = 5.6 Hz, 0.6H), 7.13 (dd, J = 4.3, 1.8 Hz, 0.6H), 7.08 (d, J = 8.8 Hz, 0.6H), 6.81 (d, J = 8.8 Hz, 0.6H), 6.15 (q, J = 7.1 Hz, 0.3H), 3.79 (s, 0.9H), 1.74 (d, J = 7.1 Hz, 0.9H). ^{13}C NMR (150 MHz, $CDCl_3$) δ 158.96, 149.62, 148.72, 139.63, 134.06, 128.22, 125.17, 124.32, 113.69, 55.29, 15.58. HRMS (ESI): calcd for $C_{15}H_{16}NO^+$ (M+H) 226.1226, found 226.1221.

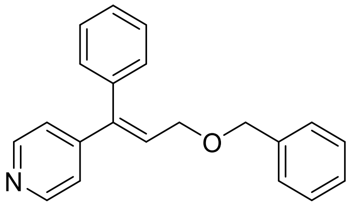

(E)-4-(3-(benzyloxy)-1-phenylprop-1-en-1-yl)pyridine (40): According to the **GPB**, (E)-(3-(benzyloxy)prop-1-en-1-yl)benzene (44.8 mg, 0.20 mmol, 1.0 equiv.), 4-cyanopyridine (41.6 mg, 0.40 mmol, 2.0 equiv.), sodium 4-chlorobenzenesulfinate (39.6 mg, 0.20 mmol, 1.0 equiv.), Ir(ppy)$_3$ (1.3 mg, 0.002 mmol, 1 mol%), NH_4Cl (21.4 mg, 0.4 mmol, 2.0 equiv.) and MeOK (70.0 mg, 1.0 mmol, 5.0 equiv.) in MeCN-EtOH (10 mL) were used. The result mixture was concentrated in vacuo and the product was

isolated by flash chromatography (PE: acetone= 10:1) as a pale-yellow oil (34.9 mg, 58%). ^{1}H NMR (400 MHz, $CDCl_3$) δ 8.51 (d, *J* = 6.0 Hz, 2H), 7.46 – 7.28 (m, 8H), 7.13 (m, 4H), 6.45 (t, *J* = 6.6 Hz, 1H), 4.48 (s, 2H), 4.12 (d, *J* = 6.6 Hz, 2H). ^{13}C NMR (100 MHz, $CDCl_3$) δ 149.85, 148.85, 142.24, 138.02, 137.50, 129.61, 129.01, 128.51, 128.46, 128.04, 127.86, 127.78, 121.93, 72.76, 67.81. HRMS (ESI): calcd for $C_{21}H_{20}NO^+$ (M+H) 302.1539, found 302.1533.

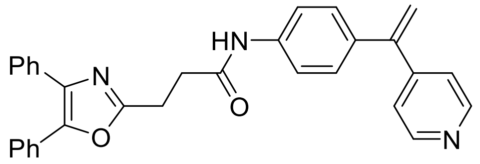

3-(4,5-diphenyloxazol-2-yl)-N-(4-(1-(pyridin-4-yl)vinyl)phenyl)propenamide (41): According to the **GPB**, 3-(4,5-diphenyloxazol-2-yl)-N-(4-vinylphenyl)propanamide (78.8 mg, 0.20 mmol, 1.0 equiv.), 4-cyanopyridine (41.6 mg, 0.40 mmol, 2.0 equiv.), sodium 4-chlorobenzenesulfinate (39.6 mg, 0.20 mmol, 1.0 equiv.), $Ir(ppy)_3$ (1.3 mg, 0.002 mmol, 1 mol%), NH_4Cl (21.4 mg, 0.4 mmol, 2.0 equiv.) and MeOK (70.0 mg, 1.0 mmol, 5.0 equiv.) in MeCN-EtOH (10 mL) were used. The result mixture was concentrated in vacuo and the product was isolated by flash chromatography (PE: EA= 2:1) as a pale-yellow solid (83.8 mg, 89%). ^{1}H NMR (600 MHz, $CDCl_3$) δ 9.08 (s, 1H), 8.56 (d, *J* = 4.8 Hz, 2H), 7.63 (d, *J* = 7.4 Hz, 2H), 7.55 (d, *J* = 7.4 Hz, 2H), 7.51 (d, *J* = 8.2 Hz, 2H), 7.39 – 7.28 (m, 6H), 7.20 (t, *J* = 6.9 Hz, 4H), 5.53 (d, *J* = 10.8 Hz, 2H), 3.13 (dt, *J* = 184.8, 6.8 Hz, 4H). ^{13}C NMR (150 MHz, $CDCl_3$) δ 170.08, 162.49, 149.78, 149.00, 147.27, 145.73, 138.35, 135.36, 134.79, 132.22, 128.75, 128.72, 128.72, 128.67, 128.67, 128.30, 127.87, 126.49, 122.94, 119.61, 116.46, 34.06, 24.02. HRMS (ESI): calcd for $C_{31}H_{26}N_3O_2^+$ (M+H) 472.2020, found 472.2010.

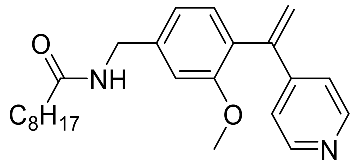

N-(3-Methoxy-4-(1-(pyridin-4-yl)vinyl)benzyl)nonanamide (42): According to the **GPA**, N-(3-methoxy-4-vinylbenzyl)nonanamide (60.0 mg, 0.20 mmol, 1.0 equiv.), 4-cyanopyridine (41.6 mg, 0.40 mmol, 2.0 equiv.), sodium 4-chlorobenzenesulfinate

(19.8 mg, 0.10 mmol, 50 mol%), Ir(ppy)$_3$ (1.3 mg, 0.002 mmol, 1 mol%), and 1,8-diazabicyclo[5.4.0]undec-7-ene (89.6 µL, 0.6 mmol, 3.0 equiv.) in MeCN-EtOH (10 mL) were used. After 24 h, the result mixture was concentrated in vacuo and the product was isolated by flash chromatography (PE: acetone= 3:1) as a pale-yellow solid (36.5 mg, 48%). ^{1}H NMR (400 MHz, CDCl$_3$) δ 8.45 (m, 2H), 7.19 (d, *J* = 7.6 Hz, 1H), 7.14 (dd, *J* = 4.6, 1.6 Hz, 2H), 6.90 (dd, *J* = 7.6, 1.3 Hz, 1H), 6.82 (s, 1H), 6.06 (s, 1H), 5.65 (d, *J* = 150.3 Hz, 2H), 4.47 (d, *J* = 5.8 Hz, 2H), 3.58 (s, 3H), 2.26 – 2.20 (m, 2H), 1.71 – 1.63 (m, 2H), 1.27 (dd, *J* = 13.8, 7.5 Hz, 10H), 0.86 (t, *J* = 6.8 Hz, 3H). ^{13}C NMR (100 MHz, CDCl$_3$) δ 173.16, 157.14, 149.63, 148.47, 144.93, 140.49, 131.35, 128.48, 120.96, 119.93, 118.74, 110.63, 55.43, 43.52, 36.86, 31.82, 29.36, 29.33, 29.17, 25.83, 22.64, 14.09. HRMS (ESI): calcd for $C_{24}H_{33}N_2O_2^+$ (M+H) 381.2537, found 381.2559.

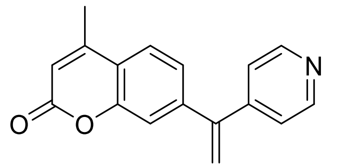

4-methyl-7-(1-(pyridin-4-yl)vinyl)-2H-chromen-2-one (43): According to the **GPB**, 4-methyl-7-vinyl-2H-chromen-2-one (37.2 mg, 0.20 mmol, 1.0 equiv.), 4-cyanopyridine (41.6 mg, 0.40 mmol, 2.0 equiv.), sodium 4-chlorobenzenesulfinate (39.6 mg, 0.20 mmol, 1.0 equiv.), Ir(ppy)$_3$ (1.3 mg, 0.002 mmol, 1 mol%), NH_4Cl (21.4 mg, 0.4 mmol, 2.0 equiv.) and MeOK (70.0 mg, 1.0 mmol, 5.0 equiv.) in MeCN-EtOH (10 mL) were used. The result mixture was concentrated in vacuo and the product was isolated by flash chromatography (PE: EA= 3:1) as a pale-yellow solid (29.5mg, 56%). ^{1}H NMR (600 MHz, CDCl$_3$) δ 8.65 (d, *J* = 4.8 Hz, 2H), 7.62 (d, *J* = 8.1 Hz, 1H), 7.31 (d, *J* = 12.7 Hz, 2H), 7.26 (d, *J* = 14.2 Hz, 2H), 6.35 (s, 1H), 5.77 (s, 2H), 2.49 (s, 3H). ^{13}C NMR (150 MHz, CDCl$_3$) δ 160.63, 153.59, 151.96, 150.13, 147.74, 146.47, 143.39, 124.68, 124.02, 122.73, 119.78, 119.02, 116.49, 115.31, 18.68. HRMS (ESI): calcd for $C_{17}H_{14}NO_2^+$ (M+H) 264.1019, found 264.1007.

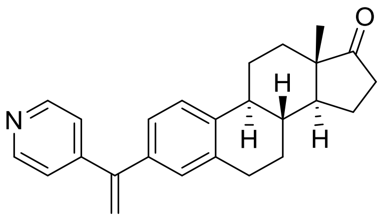

(8R,9S,13S,14S)-13-Methyl-3-(1-(pyridin-4-yl)vinyl)-6,7,8,9,11,12,13,14,15,16-decahydro-17H-cyclopenta[a]phenanthren-17-one (44): According to the **GPA**, (8R,9S,13S,14S)-13-methyl-3-vinyl-6,7,8,9,11,12,13,14,15,16-decahydro-17H-cyclopenta[a]phenanthren-17-one (56.1 mg, 0.20 mmol, 1.0 equiv.), 4-cyanopyridine (41.6 mg, 0.40 mmol, 2.0 equiv.), sodium 4-chlorobenzenesulfinate (19.8 mg, 0.10 mmol, 50 mol%), $Ir(ppy)_3$ (1.3 mg, 0.002 mmol, 1 mol%), and 1,8-diazabicyclo[5.4.0]undec-7-ene (89.6 μL, 0.6 mmol, 3.0 equiv.) in MeCN-EtOH (10 mL) were used. After 24 h, the result mixture was concentrated in vacuo and the product was isolated by flash chromatography (PE: EA= 5:1) as a pale-yellow solid (40.8 mg, 57%). ^1H NMR (400 MHz, $CDCl_3$) δ 8.56 (d, J = 6.0 Hz, 2H), 7.28 (d, J = 8.2 Hz, 1H), 7.25 (dd, J = 4.6, 1.5 Hz, 2H), 7.08 (dd, J = 8.1, 1.6 Hz, 1H), 7.02 (s, 1H), 5.56 (d, J = 10.3 Hz, 2H), 2.89 (dd, J = 8.7, 3.9 Hz, 2H), 2.51 (dd, J = 18.8, 8.7 Hz, 1H), 2.43 (dd, J = 11.3, 4.8 Hz, 1H), 2.36 – 2.30 (m, 1H), 2.16 (dd, J = 18.4, 9.3 Hz, 1H), 2.09 – 1.96 (m, 3H), 1.65 – 1.45 (m, 6H), 0.92 (s, 3H). ^{13}C NMR (150 MHz, $CDCl_3$) δ 220.74, 149.73, 149.10, 147.70, 140.00, 137.24, 136.60, 128.64, 125.55, 125.45, 122.90, 116.44, 50.52, 47.98, 44.44, 38.12, 35.86, 31.60, 29.41, 26.47, 25.70, 21.60, 13.86. HRMS (ESI): calcd for $C_{25}H_{28}NO^+$ (M+H) 358.2157, found 358.2164.

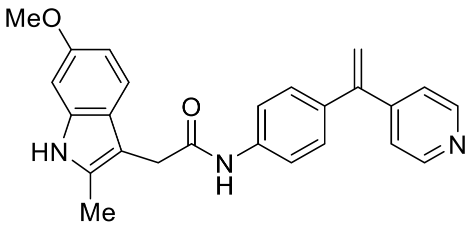

2-(6-methoxy-2-methyl-1H-indol-3-yl)-N-(4-(1-(pyridin-4-yl)vinyl)phenyl) acetamide (45): According to the **GPB**, 2-(6-methoxy-2-methyl-1H-indol-3-yl)-N-(4-vinylphenyl)acetamide (64.0 mg, 0.20 mmol, 1.0 equiv.), 4-cyanopyridine (41.6 mg, 0.40 mmol, 2.0 equiv.), sodium 4-chlorobenzenesulfinate (39.6 mg, 0.20 mmol, 1.0

equiv.), Ir(ppy)$_3$ (1.3 mg, 0.002 mmol, 1 mol%), NH_4Cl (21.4 mg, 0.4 mmol, 2.0 equiv.) and MeOK (70.0 mg, 1.0 mmol, 5.0 equiv.) in MeCN-EtOH (10 mL) were used. The result mixture was concentrated in vacuo and the product was isolated by flash chromatography (PE: EA= 2:1) as a pale-yellow solid (68.38 mg, 86%). ^{1}H NMR (600 MHz, $CDCl_3$) δ 8.55 (d, J = 5.4 Hz, 2H), 8.17 (s, 1H), 7.48 (s, 1H), 7.34 (d, J = 8.5 Hz, 2H), 7.24 (d, J = 8.7 Hz, 1H), 7.19 (dd, J = 10.3, 7.2 Hz, 4H), 6.94 (d, J = 1.7 Hz, 1H), 6.84 (dd, J = 8.7, 2.1 Hz, 1H), 5.53 (s, 2H), 3.82 (s, 3H), 3.80 (s, 2H), 2.44 (s, 3H). ^{13}C NMR (150 MHz, $CDCl_3$) δ 169.95, 154.64, 149.74, 148.93, 147.18, 137.69, 135.65, 134.48, 130.49, 128.66, 128.53, 122.87, 119.86, 116.53, 111.79, 111.51, 104.00, 99.70, 55.94, 33.43, 11.85. HRMS (ESI): calcd for $C_{25}H_{24}N_3O_2^+$ (M+H) 398.1863, found 398.1859.

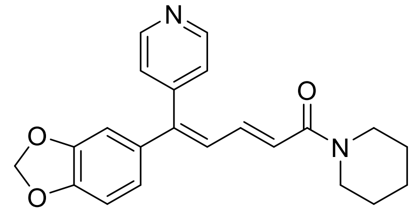

(2E,4E)-5-(benzo[d][1,3]dioxol-5-yl)-1-(piperidin-1-yl)-5-(pyridin-4-yl)penta-2,4-dien-1-one (46): According to the **GPB**, (2E,4E)-5-(benzo[d][1,3]dioxol-5-yl)-1-(piperidin-1-yl)penta-2,4-dien-1-one (57.0 mg, 0.20 mmol, 1.0 equiv.), 4-cyanopyridine (41.6 mg, 0.40 mmol, 2.0 equiv.), sodium 4-chlorobenzenesulfinate (39.6 mg, 0.20 mmol, 1.0 equiv.), Ir(ppy)$_3$ (1.3 mg, 0.002 mmol, 1 mol%), NH_4Cl (21.4 mg, 0.4 mmol, 2.0 equiv.) and MeOK (70.0 mg, 1.0 mmol, 5.0 equiv.) in MeCN-EtOH (10 mL) were used. The result mixture was concentrated in vacuo and the product was isolated by flash chromatography (PE: EA= 2:1) as a pale-yellow solid (35.5 mg, 49%). ^{1}H NMR (600 MHz, $CDCl_3$) δ 8.53 (d, J = 5.1 Hz, 2H), 7.37 (dd, J = 14.7, 11.5 Hz, 1H), 7.18 (d, J = 5.9 Hz, 2H), 6.86 (dd, J = 17.1, 9.7 Hz, 2H), 6.66 (dd, J = 7.9, 1.3 Hz, 1H), 6.61 (s, 1H), 6.59 (s, 1H), 6.00 (s, 2H), 3.55 (d, J = 50.7 Hz, 4H), 1.70 – 1.50 (m, 6H). ^{13}C NMR (150 MHz, $CDCl_3$) δ 165.01, 149.91, 148.85, 147.85, 147.85, 145.43, 138.80, 130.87, 128.70, 124.32, 124.29, 122.05, 110.49, 108.51, 101.32, 47.01, 43.21,

26.75, 25.57, 24.62. HRMS (ESI): calcd for $C_{22}H_{23}N_2O_3^+$ (M+H) 363.1703, found 363.1697.

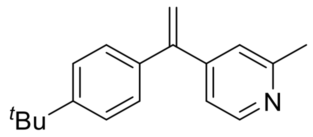

4-(1-(4-(*tert*-Butyl)phenyl)vinyl)-2-methylpyridine (47): According to the **GPB**, 4-*tert*-butylstyrene (36.6 μL, 0.20 mmol, 1.0 equiv.), 2-methylisonicotinonitrile (47.2 mg, 0.40 mmol, 2.0 equiv.), sodium 4-chlorobenzenesulfinate (39.6 mg, 0.20 mmol, 1.0 equiv.), $Ir(ppy)_3$ (1.3 mg, 0.002 mmol, 1 mol%), NH_4Cl (21.4 mg, 0.4 mmol, 2.0 equiv.) and MeOK (70.0 mg, 1.0 mmol, 5.0 equiv.) in MeCN-EtOH (10 mL) were used. The result mixture was concentrated in vacuo and the product was isolated by flash chromatography (PE: EA= 20:1) as a pale-yellow oil (39.1 mg, 78%). ^{1}H NMR (400 MHz, $CDCl_3$) δ 8.45 (d, J = 5.2 Hz, 1H), 7.37 (d, J = 8.5 Hz, 2H), 7.23 (d, J = 8.5 Hz, 2H), 7.12 (s, 1H), 7.06 (dd, J = 5.2, 1.2 Hz, 1H), 5.54 (dd, J = 20.6, 0.8 Hz, 2H), 2.56 (s, 3H), 1.34 (s, 9H). ^{13}C NMR (150 MHz, $CDCl_3$) δ 158.43, 151.28, 149.46, 149.10, 147.92, 136.89, 127.73, 125.31, 122.41, 120.18, 115.99, 34.63, 31.32, 24.47. HRMS (ESI): calcd for $C_{18}H_{22}N^+$ (M+H) 252.1752, found 252.1748.

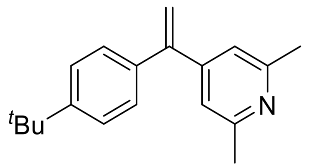

4-(1-(4-(*tert*-Butyl)phenyl)vinyl)-2,6-dimethylpyridine (48): According to the **GPB**, 4-*tert*-butylstyrene (36.6 μL, 0.20 mmol, 1.0 equiv.), 2,6-dimethylisonicotinonitrile (52.8 mg, 0.40 mmol, 2.0 equiv.), sodium 4-chlorobenzenesulfinate (39.6 mg, 0.20 mmol, 1.0 equiv.), $Ir(ppy)_3$ (1.3 mg, 0.002 mmol, 1 mol%), NH_4Cl (21.4 mg, 0.4 mmol, 2.0 equiv.) and MeOK (70.0 mg, 1.0 mmol, 5.0 equiv.) in MeCN-EtOH (10 mL) were used. The result mixture was concentrated in vacuo and the product was isolated by flash chromatography (PE: EA= 10:1) as a pale-yellow oil (32.9 mg, 62%). ^{1}H NMR (600 MHz, $CDCl_3$) δ 7.37 (d, J = 8.3 Hz, 2H), 7.23 (d, J = 8.3 Hz, 2H), 6.92 (s, 2H),

5.51 (d, J = 34.3 Hz, 2H), 2.52 (s, 6H), 1.34 (s, 9H). ^{13}C NMR (150 MHz, $CDCl_3$) δ 157.71, 151.16, 149.78, 148.11, 137.04, 127.70, 125.26, 119.60, 115.67, 34.62, 31.32, 24.50. HRMS (ESI): calcd for $C_{19}H_{24}N^+$ (M+H) 266.1909, found 266.1902.

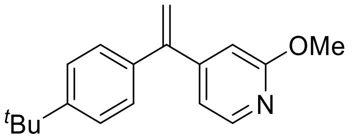

4-(1-(4-(tert-butyl)phenyl)vinyl)-2-methoxypyridine (49): According to the **GPB**, 4-*tert*-butylstyrene (36.6 μL, 0.20 mmol, 1.0 equiv.), 2-methoxyisonicotinonitrile (53.6 mg, 0.40 mmol, 2.0 equiv.), sodium 4-chlorobenzenesulfinate (39.6 mg, 0.20 mmol, 1.0 equiv.), Ir(ppy)$_3$ (1.3 mg, 0.002 mmol, 1 mol%), NH_4Cl (21.4 mg, 0.4 mmol, 2.0 equiv.) and MeOK (70.0 mg, 1.0 mmol, 5.0 equiv.) in MeCN-EtOH (10 mL) were used. The result mixture was concentrated in vacuo and the product was isolated by flash chromatography (PE: EA= 10:1) as a pale-yellow oil (44.9 mg, 84%). ^{1}H NMR (600 MHz, $CDCl_3$) δ 8.12 (d, J = 5.3 Hz, 1H), 7.36 (d, J = 8.4 Hz, 2H), 7.24 (d, J = 8.4 Hz, 2H), 6.86 (dd, J = 5.3, 1.4 Hz, 1H), 6.71 (s, 1H), 5.54 (d, J = 13.2 Hz, 2H), 3.95 (s, 3H), 1.34 (s, 9H). ^{13}C NMR (150 MHz, $CDCl_3$) δ 164.57, 151.94, 151.20, 147.69, 146.59, 136.83, 127.75, 125.26, 116.54, 115.93, 110.00, 53.47, 34.61, 31.31. HRMS (ESI): calcd for $C_{18}H_{22}NO^+$ (M+H) 268.1696, found 268.1690.

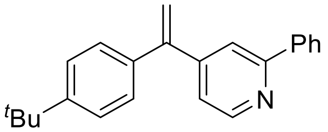

4-(1-(4-(*tert*-Butyl)phenyl)vinyl)-2-phenylpyridine (50): According to the **GPB**, 4-*tert*-butylstyrene (36.6 μL, 0.20 mmol, 1.0 equiv.), 2-phenylisonicotinonitrile (72.0 mg, 0.40 mmol, 2.0 equiv.), sodium 4-chlorobenzenesulfinate (39.6 mg, 0.20 mmol, 1.0 equiv.), Ir(ppy)$_3$ (1.3 mg, 0.002 mmol, 1 mol%), NH_4Cl (21.4 mg, 0.4 mmol, 2.0 equiv.) and MeOK (70.0 mg, 1.0 mmol, 5.0 equiv.) in MeCN-EtOH (10 mL) were used. The result mixture was concentrated in vacuo and the product was isolated by flash chromatography (PE: EA= 50:1) as a pale-yellow oil (53.8 mg, 86%). ^{1}H NMR (600

MHz, $CDCl_3$) δ 8.66 (d, *J* = 5.0 Hz, 1H), 7.98 (d, *J* = 7.9 Hz, 2H), 7.69 (s, 1H), 7.47 (t, *J* = 7.6 Hz, 2H), 7.42 (d, *J* = 7.4 Hz, 1H), 7.39 (d, *J* = 8.4 Hz, 2H), 7.29 (d, *J* = 8.4 Hz, 2H), 7.21 (dd, *J* = 5.0, 1.6 Hz, 1H), 5.62 (d, *J* = 29.8 Hz, 2H), 1.35 (s, 9H). ^{13}C NMR (100 MHz, $CDCl_3$) δ 157.71, 151.42, 150.09, 149.71, 148.03, 139.49, 136.77, 128.97, 128.73, 127.74, 127.06, 125.42, 121.64, 119.96, 116.16, 34.65, 31.32. HRMS (ESI): calcd for $C_{23}H_{24}N^+$ (M+H) 314.1892, found 314.1909.

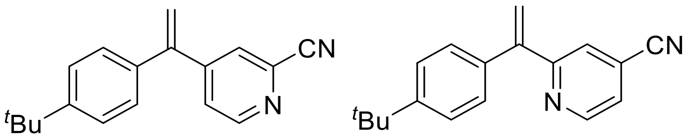

2-(1-(4-(*tert*-Butyl)phenyl)vinyl)isonicotinonitrile, 4-(1-(4-(*tert*-butyl)phenyl)vinyl)picolinonitrile (51): According to the **GPB**, 4-*tert*-butylstyrene (36.6 μL, 0.20 mmol, 1.0 equiv.), pyridine-2,4-dicarbonitrile (51.6 mg, 0.40 mmol, 2.0 equiv.), sodium 4-chlorobenzenesulfinate (39.6 mg, 0.20 mmol, 1.0 equiv.), $Ir(ppy)_3$ (1.3 mg, 0.002 mmol, 1 mol%), NH_4Cl (21.4 mg, 0.4 mmol, 2.0 equiv.) and MeOK (70.0 mg, 1.0 mmol, 5.0 equiv.) in MeCN-EtOH (10 mL) were used. The result mixture was concentrated in vacuo and the product was isolated by flash chromatography (PE: EA= 10:1) as a pale-yellow oil (42.2 mg, 81%, mixture of two isomer 1.7: 1). ^{1}H NMR (400 MHz, $CDCl_3$) δ 8.67 (d, *J* = 5.0 Hz, 1H), 7.65 (d, *J* = 1.0 Hz, 1H), 7.49 (dd, *J* = 5.1, 1.7 Hz, 1H), 7.43 – 7.38 (m, 2H), 7.21 – 7.16 (m, 2H), 5.66 (d, *J* = 20.9 Hz, 2H), 1.35 (s, 9H). ^{13}C NMR (100 MHz, $CDCl_3$) δ 152.05, 151.18, 150.57, 146.18, 135.65, 134.13, 127.75, 127.68, 125.79, 125.72, 118.22, 117.34, 34.72, 31.28. HRMS (ESI): calcd for $C_{18}H_{19}N_2^+$ (M+H) 263.1543, found 263.1533. ^{1}H NMR (400 MHz, $CDCl_3$) δ 8.81 (dd, *J* = 4.9, 0.7 Hz, 1H), 7.50 (s, 1H), 7.44 – 7.40 (m, 3H), 7.26 – 7.24 (m, 2H), 5.88 (dd, *J* = 159.0, 1.1 Hz, 2H), 1.36 (s, 9H). ^{13}C NMR (100 MHz, $CDCl_3$) δ 159.98, 151.49, 150.32, 147.44, 136.11, 128.03, 125.61, 124.28, 123.57, 120.75, 119.46, 116.66, 34.68, 31.33. HRMS (ESI): calcd for $C_{18}H_{19}N_2^+$ (M+H) 263.1543, found 263.1567.

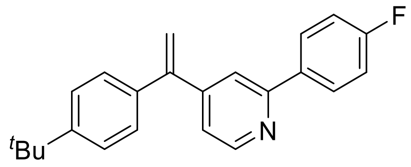

4-(1-(4-(tert-butyl)phenyl)vinyl)-2-(4-fluorophenyl)pyridine (52): According to the **GPB**, 4-*tert*-butylstyrene (36.6 μL, 0.20 mmol, 1.0 equiv.), 2-(4-fluorophenyl)isonicotinonitrile (79.2 mg, 0.40 mmol, 2.0 equiv.), sodium 4-chlorobenzenesulfinate (39.6 mg, 0.20 mmol, 1.0 equiv.), $Ir(ppy)_3$ (1.3 mg, 0.002 mmol, 1 mol%), NH_4Cl (21.4 mg, 0.4 mmol, 2.0 equiv.) and MeOK (70.0 mg, 1.0 mmol, 5.0 equiv.) in MeCN-EtOH (10 mL) were used. The result mixture was concentrated in vacuo and the product was isolated by flash chromatography (PE: EA= 100:1) as a pale-yellow oil (49.0 mg, 74%). ^1H NMR (400 MHz, $CDCl_3$) δ 8.64 (d, *J* = 5.1 Hz, 1H), 7.97 (dd, *J* = 8.7, 5.5 Hz, 2H), 7.65 (s, 1H), 7.39 (d, *J* = 8.3 Hz, 2H), 7.28 (d, *J* = 8.3 Hz, 2H), 7.20 (dd, *J* = 5.0, 1.2 Hz, 1H), 7.14 (t, *J* = 8.7 Hz, 2H), 5.61 (d, *J* = 23.8 Hz, 2H), 1.35 (s, 9H). ^{13}C NMR (100 MHz, $CDCl_3$) δ 163.54 (d, *J* = 248.4 Hz), 156.67, 151.48, 150.21, 149.70, 147.95, 136.70, 135.61 (d, *J* = 3.1 Hz), 128.86 (d, *J* = 8.3 Hz), 127.72, 125.44, 121.60, 119.58, 116.24, 115.63 (d, *J* = 21.6 Hz), 34.66, 31.32. HRMS (ESI): calcd for $C_{23}H_{23}FN^+$ (M+H) 332.1809, found 332.1812.

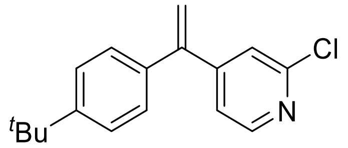

4-(1-(4-(*tert*-Butyl)phenyl)vinyl)-2-chloropyridine (53): According to the **GPB**, 4-*tert*-butylstyrene (36.6 μL, 0.20 mmol, 1.0 equiv.), 2-chloroisonicotinonitrile (55.4 mg, 0.40 mmol, 2.0 equiv.), sodium 4-chlorobenzenesulfinate (39.6 mg, 0.20 mmol, 1.0 equiv.), $Ir(ppy)_3$ (1.3 mg, 0.002 mmol, 1 mol%), NH_4Cl (21.4 mg, 0.4 mmol, 2.0 equiv.) and 1,8-diazabicyclo[5.4.0]undec-7-ene (149.3 μL, 1.0 mmol, 5.0 equiv.) in MeCN-EtOH (10 mL) were used. The result mixture was concentrated in vacuo and the product was isolated by flash chromatography (PE: EA= 10:1) as a pale-yellow oil (39.2 mg, 72%). ^1H NMR (400 MHz, $CDCl_3$) δ 8.34 (dd, *J* = 5.2, 0.4 Hz, 1H), 7.40 – 7.37 (m, 2H), 7.30 (d, *J* = 0.9 Hz, 1H), 7.23 – 7.21 (m, 1H), 7.20 (t, *J* = 2.1 Hz, 1H), 7.18 (dd,

J = 5.2, 1.5 Hz, 1H), 5.59 (d, J = 22.8 Hz, 2H), 1.34 (s, 9H). ^{13}C NMR (100 MHz, $CDCl_3$) δ 152.37, 151.83, 151.70, 149.58, 146.71, 136.16, 127.72, 125.53, 123.31, 121.78, 117.35, 34.68, 31.30. HRMS (ESI): calcd for $C_{17}H_{19}ClN^+$ (M+H) 272.1201, found 272.1207.

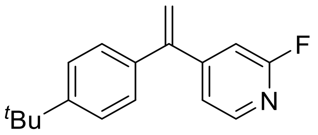

4-(1-(4-(*tert*-Butyl)phenyl)vinyl)-2-fluoropyridine (54): According to the **GPB**, 4-*tert*-butylstyrene (36.6 μL, 0.20 mmol, 1.0 equiv.), 2-fluoroisonicotinonitrile (48.8 mg, 0.40 mmol, 2.0 equiv.), sodium 4-chlorobenzenesulfinate (39.6 mg, 0.20 mmol, 1.0 equiv.), $Ir(ppy)_3$ (1.3 mg, 0.002 mmol, 1 mol%), NH_4Cl (21.4 mg, 0.4 mmol, 2.0 equiv.) and 1,8-diazabicyclo[5.4.0]undec-7-ene (149.3 μL, 1.0 mmol, 5.0 equiv.) in MeCN-EtOH (10 mL) were used. The result mixture was concentrated in vacuo and the product was isolated by flash chromatography (PE: EA= 20:1) as a pale-yellow oil (36.2 mg, 71%). ^{1}H NMR (400 MHz, $CDCl_3$) δ 8.17 (d, J = 5.2 Hz, 1H), 7.41 – 7.37 (m, 2H), 7.24 – 7.20 (m, 2H), 7.17 – 7.14 (m, 1H), 6.89 (s, 1H), 5.61 (d, J = 15.5 Hz, 2H), 1.34 (s, 9H). ^{13}C NMR (100 MHz, $CDCl_3$) δ 164.20 (d, J = 238.2 Hz), 154.84 (d, J = 8.0 Hz), 151.66, 147.45 (d, J = 15.3 Hz), 146.89 (d, J = 3.2 Hz), 136.26, 127.75, 125.49, 120.75 (d, J = 4.0 Hz), 117.21, 108.54 (d, J = 37.8 Hz), 34.66, 31.30. HRMS (ESI): calcd for $C_{17}H_{19}FN^+$ (M+H) 256.1496, found 256.1518.

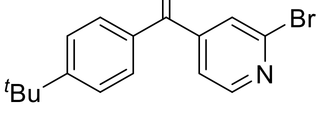

2-Bromo-4-(1-(4-(*tert*-butyl)phenyl)vinyl)pyridine (55): According to the **GPB**, 4-*tert*-butylstyrene (36.6 μL, 0.20 mmol, 1.0 equiv.), 2-bromoisonicotinonitrile (73.2 mg, 0.40 mmol, 2.0 equiv.), sodium 4-chlorobenzenesulfinate (39.6 mg, 0.20 mmol, 1.0 equiv.), $Ir(ppy)_3$ (1.3 mg, 0.002 mmol, 1 mol%), NH_4Cl (21.4 mg, 0.4 mmol, 2.0 equiv.)

and 1,8-diazabicyclo[5.4.0]undec-7-ene (149.3 μL, 1.0 mmol, 5.0 equiv.) in MeCN-EtOH (10 mL) were used. The result mixture was concentrated in vacuo and the product was isolated by flash chromatography (PE: EA= 20:1) as a pale-yellow oil (44.9 mg, 71%). ^{1}H NMR (400 MHz, $CDCl_3$) δ 8.32 (d, J = 5.1 Hz, 1H), 7.46 (d, J = 1.0 Hz, 1H), 7.40 – 7.37 (m, 2H), 7.21 (dd, J = 6.1, 2.1 Hz, 3H), 5.59 (d, J = 26.1 Hz, 2H), 1.34 (s, 9H). ^{13}C NMR (100 MHz, $CDCl_3$) δ 152.12, 151.71, 149.99, 146.59, 142.54, 136.12, 127.71, 127.04, 125.54, 122.16, 117.41, 34.68, 31.30. HRMS (ESI): calcd for $C_{17}H_{19}BrN^+$ (M+H) 316.0695, found 316.0710.

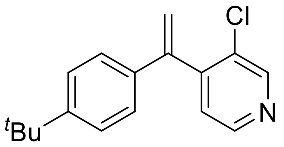

4-(1-(4-(*tert*-Butyl)phenyl)vinyl)-3-chloropyridine (56): According to the **GPB**, 4-*tert*-butylstyrene (36.6 μL, 0.20 mmol, 1.0 equiv.), 3-chloroisonicotinonitrile (55.4 mg, 0.40 mmol, 2.0 equiv.), sodium 4-chlorobenzenesulfinate (39.6 mg, 0.20 mmol, 1.0 equiv.), $Ir(ppy)_3$ (1.3 mg, 0.002 mmol, 1 mol%), NH_4Cl (21.4 mg, 0.4 mmol, 2.0 equiv.) and MeOK (70.0 mg, 1.0 mmol, 5.0 equiv.) in MeCN-EtOH (10 mL) were used. The result mixture was concentrated in vacuo and the product was isolated by flash chromatography (PE: EA= 10:1) as a pale-yellow oil (38.4 mg, 71%). ^{1}H NMR (400 MHz, $CDCl_3$) δ 8.62 (s, 1H), 8.50 (d, J = 4.9 Hz, 1H), 7.37 – 7.32 (m, 2H), 7.23 (d, J = 4.7 Hz, 1H), 7.19 – 7.15 (m, 2H), 5.59 (d, J = 239.5 Hz, 2H), 1.32 (s, 9H). ^{13}C NMR (100 MHz, $CDCl_3$) δ 151.47, 149.88, 148.50, 147.75, 144.83, 135.25, 131.17, 126.06, 125.68, 125.51, 116.59, 34.62, 31.28. HRMS (ESI): calcd for $C_{17}H_{19}ClN^+$ (M+H) 272.1201, found 272.1235.

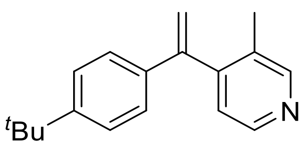

4-(1-(4-(*tert*-Butyl)phenyl)vinyl)-3-methylpyridine (57): According to the **GPB**, 4-*tert*-butylstyrene (36.6 μL, 0.20 mmol, 1.0 equiv.), 3-methylisonicotinonitrile (47.2 mg,

0.40 mmol, 2.0 equiv.), sodium 4-chlorobenzenesulfinate (39.6 mg, 0.20 mmol, 1.0 equiv.), Ir(ppy)$_3$ (1.3 mg, 0.002 mmol, 1 mol%), NH_4Cl (21.4 mg, 0.4 mmol, 2.0 equiv.) and MeOK (70.0 mg, 1.0 mmol, 5.0 equiv.) in MeCN-EtOH (10 mL) were used. The result mixture was concentrated in vacuo and the product was isolated by flash chromatography (PE: EA= 20:1) as a pale-yellow oil (40.0 mg, 80%). ^{1}H NMR (400 MHz, $CDCl_3$) δ 8.46 – 8.42 (m, 2H), 7.34 – 7.30 (m, 2H), 7.18 – 7.14 (m, 2H), 7.12 (d, *J* = 4.9 Hz, 1H), 5.49 (dd, *J* = 258.4, 0.8 Hz, 2H), 2.06 (s, 3H), 1.31 (s, 9H). ^{13}C NMR (100 MHz, $CDCl_3$) δ 151.29, 150.97, 149.58, 147.41, 146.82, 135.94, 131.55, 126.04, 125.48, 124.31, 114.93, 34.58, 31.27, 16.90. HRMS (ESI): calcd for $C_{18}H_{22}N^+$ (M+H) 252.1747, found 252.1768.

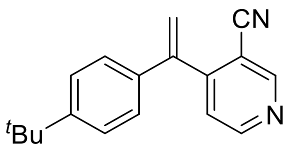

4-(1-(4-(*tert*-Butyl)phenyl)vinyl)nicotinonitrile (58): According to the **GPB**, 4-*tert*-butylstyrene (36.6 μL, 0.20 mmol, 1.0 equiv.), pyridine-3,4-dicarbonitrile (51.6 mg, 0.40 mmol, 2.0 equiv.), sodium 4-chlorobenzenesulfinate (39.6 mg, 0.20 mmol, 1.0 equiv.), Ir(ppy)$_3$ (1.3 mg, 0.002 mmol, 1 mol%), NH_4Cl (21.4 mg, 0.4 mmol, 2.0 equiv.) and 1,8-diazabicyclo[5.4.0]undec-7-ene (149.3 μL, 1.0 mmol, 5.0 equiv.) in MeCN-EtOH (10 mL) were used. The result mixture was concentrated in vacuo and the product was isolated by flash chromatography (PE: EA= 10:1) as a pale-yellow oil (45.1 mg, 86%). ^{1}H NMR (400 MHz, $CDCl_3$) δ 8.92 (d, *J* = 0.5 Hz, 1H), 8.74 (d, *J* = 5.2 Hz, 1H), 7.39 – 7.36 (m, 2H), 7.30 (dd, *J* = 5.2, 0.6 Hz, 1H), 7.19 – 7.15 (m, 2H), 5.76 (d, *J* = 151.7 Hz, 2H), 1.32 (s, 9H). ^{13}C NMR (100 MHz, $CDCl_3$) δ 153.77, 153.22, 152.49, 152.11, 144.37, 135.37, 127.04, 125.69, 124.30, 119.13, 116.02, 109.73, 34.69, 31.25. HRMS (ESI): calcd for $C_{18}H_{19}N_2^+$ (M+H) 263.1548, found 263.1564.

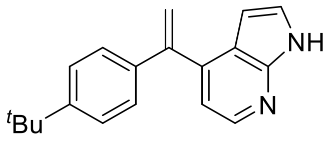

4-(1-(4-(*tert*-Butyl)phenyl)vinyl)-1H-pyrrolo[2,3-b]pyridine (59): According to the **GPB**, 4-*tert*-butylstyrene (36.6 μL, 0.20 mmol, 1.0 equiv.), *tert*-butyl 4-cyano-1H-pyrrolo[2,3-b]pyridine-1-carboxylate (57.3 mg, 0.40 mmol, 2.0 equiv.), sodium 4-chlorobenzenesulfinate (39.6 mg, 0.20 mmol, 1.0 equiv.), Ir(ppy)$_3$ (1.3 mg, 0.002 mmol, 1 mol%), NH_4Cl (21.4 mg, 0.4 mmol, 2.0 equiv.) and MeOK (70.0 mg, 1.0 mmol, 5.0 equiv.) in MeCN-EtOH (10 mL) were used. The result mixture was concentrated in vacuo and the product was isolated by flash chromatography (DCM: MeOH= 100:1) as a pale-yellow solid (40.0 mg, 73%). ^{1}H NMR (400 MHz, $CDCl_3$) δ 10.61 (s, 1H), 8.32 (d, J = 4.9 Hz, 1H), 7.36 (d, J = 8.4 Hz, 2H), 7.30 (d, J = 8.2 Hz, 3H), 7.04 (d, J = 5.0 Hz, 1H), 6.25 (d, J = 3.4 Hz, 1H), 5.72 (d, J = 71.2 Hz, 2H), 1.34 (s, 9H). ^{13}C NMR (100 MHz, $CDCl_3$) δ 151.11, 149.18, 147.13, 142.65, 142.59, 137.50, 127.43, 125.22, 124.75, 119.32, 116.65, 116.00, 101.03, 34.61, 31.35. HRMS (ESI): calcd for $C_{19}H_{21}N_2^+$ (M+H) 277.1699, found 277.1706.

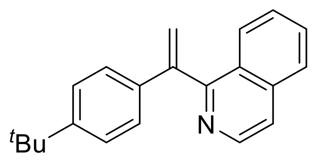

1-(1-(4-(tert-butyl)phenyl)vinyl)isoquinoline (60): According to the **GPB**, 4-*tert*-butylstyrene (36.6 μL, 0.20 mmol, 1.0 equiv.), isoquinoline-1-carbonitrile (61.6 mg, 0.40 mmol, 2.0 equiv.), sodium 4-chlorobenzenesulfinate (39.6 mg, 0.20 mmol, 1.0 equiv.), Ir(ppy)$_3$ (1.3 mg, 0.002 mmol, 1 mol%), NH_4Cl (21.4 mg, 0.4 mmol, 2.0 equiv.) and MeOK (70.0 mg, 1.0 mmol, 5.0 equiv.) in MeCN-EtOH (10 mL) were used (the MeOK was added after dilution of the concentrated result mixture with 4 mL THF). The result mixture was concentrated in vacuo and the product was isolated by flash chromatography (PE: EA= 20:1) as a pale-yellow oil (48.9 mg, 85%). ^{1}H NMR (600 MHz, $CDCl_3$) δ 8.59 (d, J = 5.7 Hz, 1H), 8.07 (d, J = 8.4 Hz, 1H), 7.85 (d, J = 8.2 Hz, 1H), 7.65 (dd, J = 10.4, 6.5 Hz, 2H), 7.48 (t, J = 7.6 Hz, 1H), 7.28 (dd, J = 32.8, 8.3 Hz, 4H), 5.79 (d, J = 396.9 Hz, 2H), 1.29 (s, 9H). ^{13}C NMR (150 MHz, $CDCl_3$) δ 161.51, 150.95, 146.86, 142.21, 137.08, 136.59, 130.11, 127.57, 127.44, 127.12,

126.84, 126.37, 125.43, 120.11, 116.91, 34.55, 31.27. HRMS (ESI): calcd for $C_{21}H_{22}N^+$ (M+H) 288.1747, found 288.1740.

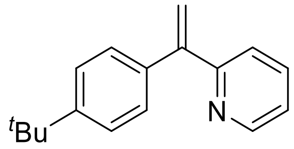

2-(1-(4-(*tert*-Butyl)phenyl)vinyl)pyridine (13): According to the **GPB**, 4-*tert*-butylstyrene (36.6 μL, 0.20 mmol, 1.0 equiv.), 2-cyanopyridine (41.6 mg, 0.40 mmol, 2.0 equiv.), sodium 4-chlorobenzenesulfinate (39.6 mg, 0.20 mmol, 1.0 equiv.), $Ir(ppy)_3$ (1.3 mg, 0.002 mmol, 1 mol%), NH_4Cl (21.4 mg, 0.4 mmol, 2.0 equiv.) and MeOK (70.0 mg, 1.0 mmol, 5.0 equiv.) in MeCN-EtOH (10 mL) were used. The result mixture was concentrated in vacuo and the product was isolated by flash chromatography (PE: EA= 20:1) as a pale-yellow oil (35.6 mg, 75%). ^1H NMR (400 MHz, $CDCl_3$) δ 8.65 (dd, J = 4.8, 0.9 Hz, 1H), 7.64 (td, J = 7.7, 1.8 Hz, 1H), 7.41 – 7.36 (m, 2H), 7.33 – 7.28 (m, 3H), 7.21 (ddd, J = 7.5, 4.8, 1.0 Hz, 1H), 5.77 (dd, J = 128.1, 1.4 Hz, 2H), 1.34 (s, 9H). ^{13}C NMR (100 MHz, $CDCl_3$) δ 158.84, 150.81, 149.37, 149.02, 137.35, 136.26, 128.06, 125.23, 122.96, 122.38, 117.16, 34.59, 31.36. HRMS (ESI): calcd for $C_{17}H_{20}N^+$ (M+H) 238.1590, found 238.1598.

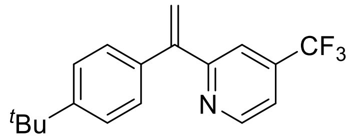

2-(1-(4-(tert-butyl)phenyl)vinyl)-4-(trifluoromethyl)pyridine (61): According to the **GPB**, 4-*tert*-butylstyrene (36.6 μL, 0.20 mmol, 1.0 equiv.), 4-(trifluoromethyl)picolinonitrile (68.8 mg, 0.40 mmol, 2.0 equiv.), sodium 4-chlorobenzenesulfinate (39.6 mg, 0.20 mmol, 1.0 equiv.), $Ir(ppy)_3$ (1.3 mg, 0.002 mmol, 1 mol%), NH_4Cl (21.4 mg, 0.4 mmol, 2.0 equiv.) and MeOK (70.0 mg, 1.0 mmol, 5.0 equiv.) in MeCN-EtOH (10 mL) were used (the MeOK was added after dilution of the

concentrated result mixture with 4 mL THF). The result mixture was concentrated in vacuo and the product was isolated by flash chromatography (PE: EA= 20:1) as a pale-yellow oil (48.2 mg, 79%). ^{1}H NMR (600 MHz, $CDCl_3$) δ 8.90 (s, 1H), 7.87 (dd, J = 8.1, 1.6 Hz, 1H), 7.43 (d, J = 8.3 Hz, 1H), 7.40 (d, J = 8.2 Hz, 2H), 7.27 (d, J = 8.3 Hz, 2H), 5.88 (d, J = 209.0 Hz, 2H), 1.35 (s, 9H). ^{13}C NMR (150 MHz, $CDCl_3$) δ 162.04, 151.26, 148.00, 146.27 (q, J = 3.9 Hz), 136.52, 133.48 (q, J = 3.4 Hz), 128.01, 125.42, 125.10 (q, J = 33.1 Hz), 123.67 (q, J = 272.1 Hz), 122.40, 119.30, 34.64, 31.32. HRMS (ESI): calcd for $C_{18}H_{19}F_3N^+$ (M+H) 306.1464, found 306.1458.

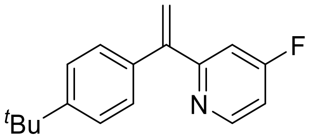

2-(1-(4-(tert-butyl)phenyl)vinyl)-4-fluoropyridine (62): According to the **GPB**, 4-*tert*-butylstyrene (36.6 μL, 0.20 mmol, 1.0 equiv.), 4-fluoropicolinonitrile (48.8 mg, 0.40 mmol, 2.0 equiv.), sodium 4-chlorobenzenesulfinate (39.6 mg, 0.20 mmol, 1.0 equiv.), $Ir(ppy)_3$ (1.3 mg, 0.002 mmol, 1 mol%), NH_4Cl (21.4 mg, 0.4 mmol, 2.0 equiv.) and MeOK (70.0 mg, 1.0 mmol, 5.0 equiv.) in MeCN-EtOH (10 mL) were used (the MeOK was added after dilution of the concentrated result mixture with 4 mL THF). The result mixture was concentrated in vacuo and the product was isolated by flash chromatography (PE: EA= 20:1) as a pale-yellow oil (40.8 mg, 80%). ^{1}H NMR (600 MHz, $CDCl_3$) δ 8.50 (d, J = 2.5 Hz, 1H), 7.39 (d, J = 8.2 Hz, 2H), 7.37 – 7.30 (m, 2H), 7.28 (d, J = 8.3 Hz, 2H), 5.73 (d, J = 174.0 Hz, 2H), 1.35 (s, 9H). ^{13}C NMR (150 MHz, $CDCl_3$) δ 158.75 (d, J = 256.6 Hz), 155.07 (d, J = 4.1 Hz), 150.98, 147.95, 137.53 (d, J = 23.6 Hz), 137.19, 127.96, 125.30, 123.78 (d, J = 4.1 Hz), 122.87 (d, J = 18.3 Hz), 117.13, 34.60, 31.34. HRMS (ESI): calcd for $C_{17}H_{19}FN^+$ (M+H) 256.1496, found 256.1490.

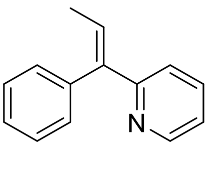

(E)-2-(1-phenylprop-1-en-1-yl)pyridine (63): According to the **GPB**, (E)-prop-1-en-1-ylbenzene (23.6 mg, 0.20 mmol, 1.0 equiv.), 2-cyanopyridine (41.6 mg, 0.40 mmol, 2.0 equiv.), sodium 4-chlorobenzenesulfinate (39.6 mg, 0.20 mmol, 1.0 equiv.), $Ir(ppy)_3$ (1.3 mg, 0.002 mmol, 1 mol%), NH_4Cl (21.4 mg, 0.4 mmol, 2.0 equiv.) and MeOK (70.0 mg, 1.0 mmol, 5.0 equiv.) in MeCN-EtOH (10 mL) were used (the MeOK was added after dilution of the concentrated result mixture with 4 mL THF). The result mixture was concentrated in vacuo and the product was isolated by flash chromatography (PE: EA= 20:1) as a pale-yellow oil (27.0 mg, 69%). ^{1}H NMR (600 MHz, $CDCl_3$) δ 8.58 (d, J = 4.0 Hz, 1H), 7.50 (td, J = 7.8, 1.8 Hz, 1H), 7.41 (t, J = 7.5 Hz, 2H), 7.34 (t, J = 7.4 Hz, 1H), 7.24 – 7.18 (m, 2H), 7.09 (dd, J = 7.0, 5.2 Hz, 1H), 6.96 (q, J = 7.2 Hz, 1H), 6.88 (d, J = 8.0 Hz, 1H), 1.77 (d, J = 7.2 Hz, 3H). ^{13}C NMR (150 MHz, $CDCl_3$) δ 158.78, 149.11, 141.57, 138.75, 136.20, 130.07, 128.42, 128.27, 127.11, 121.95, 121.50, 15.52. HRMS (ESI): calcd for $C_{14}H_{14}N^+$ (M+H) 196.1121, found 196.1115.

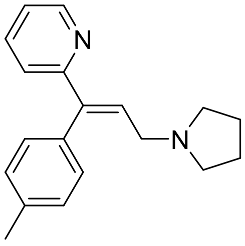

(E)-2-(3-(pyrrolidin-1-yl)-1-(p-tolyl)prop-1-en-1-yl)pyridine: According to the **GPB**, (E)-1-(3-(p-tolyl)allyl)pyrrolidine (20.1 mg, 0.10 mmol, 1.0 equiv.), 2-cyanopyridine (20.8 mg, 0.20 mmol, 2.0 equiv.), sodium 4-chlorobenzenesulfinate (29.7 mg, 0.15 mmol, 1.0 equiv.), $Ir(ppy)_3$ (0.7 mg, 0.001 mmol, 1 mol%) and MeOK (35.0 mg, 0.50 mmol, 5.0 equiv.) in MeCN-EtOH (6 mL) were used. The result mixture was concentrated in vacuo and purified by basic aluminum oxide column chromatography (PE/DCM = 2:1) to give the desired product as a pale-yellow oil (13.4 mg, 48%). ^{1}H NMR (400 MHz, $CDCl_3$) δ 8.57 (dd, J = 4.7, 0.9 Hz, 1H), 7.52 (td, J = 7.8, 1.8 Hz, 1H), 7.21 (d, J = 7.8 Hz, 2H), 7.10 (m, 3H), 7.00 (d, J = 8.0 Hz, 1H), 6.93 (t, J = 6.9 Hz, 1H), 3.22 (d, J = 6.9 Hz, 2H), 2.55 (m, 4H), 2.39 (s, 3H), 1.77 (m, 4H). ^{13}C NMR (100 MHz, $CDCl_3$) δ 158.67, 149.22, 142.34, 137.01, 136.16, 135.46, 129.74,

129.13, 122.18, 121.81, 54.57, 53.99, 23.52, 21.30. HRMS (ESI): calcd for $C_{19}H_{23}N_2^+$ (M+H) 279.1861, found 279.1861.

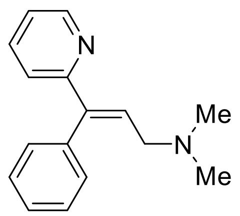

(E)-N,N-dimethyl-3-phenyl-3-(pyridin-2-yl)prop-2-en-1-amine (67): According to the **GPB**, (E)-N,N-dimethyl-3-phenylprop-2-en-1-amine (16.1 mg, 0.10 mmol, 1.0 equiv.), 2-cyanopyridine (20.8 mg, 0.20 mmol, 2.0 equiv.), sodium 4-chlorobenzenesulfinate (29.7 mg, 0.15 mmol, 1.0 equiv.), Ir(ppy)$_3$ (0.7 mg, 0.001 mmol, 1 mol%) and MeOK (35.0 mg, 0.50 mmol, 5.0 equiv.) in MeCN-EtOH (6 mL) were used. The result mixture was concentrated in vacuo and purified by basic aluminum oxide column chromatography (PE/DCM = 2:1) to give the desired product as a pale-yellow oil (10.0 mg, 42%). ^{1}H NMR (400 MHz, $CDCl_3$) δ 8.59 (d, *J* = 4.7 Hz, 1H), 7.53 (td, *J* = 7.7, 1.7 Hz, 1H), 7.41 (t, *J* = 7.2 Hz, 2H), 7.35 (t, *J* = 7.3 Hz, 1H), 7.22 – 7.18 (m, 2H), 7.12 (dd, *J* = 7.4, 4.8 Hz, 1H), 6.97 (d, *J* = 8.0 Hz, 1H), 6.93 (t, *J* = 6.9 Hz, 1H), 3.01 (d, *J* = 6.9 Hz, 2H), 2.25 (s, 6H). ^{13}C NMR (100 MHz, $CDCl_3$) δ 158.37, 149.25, 142.69, 138.55, 136.22, 130.34, 129.81, 128.40, 127.33, 122.11, 121.87, 58.25, 45.47. HRMS (ESI): calcd for $C_{16}H_{19}N_2^+$ (M+H) 239.1543, found 239.1535.

Large scale reaction

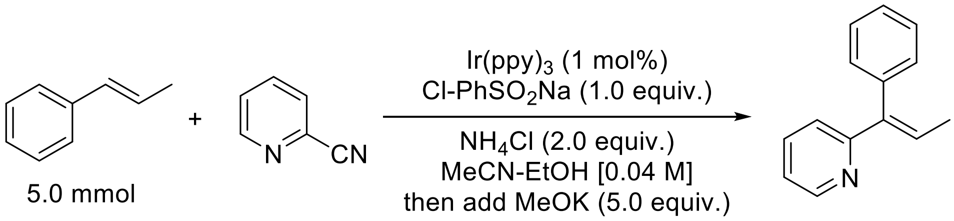

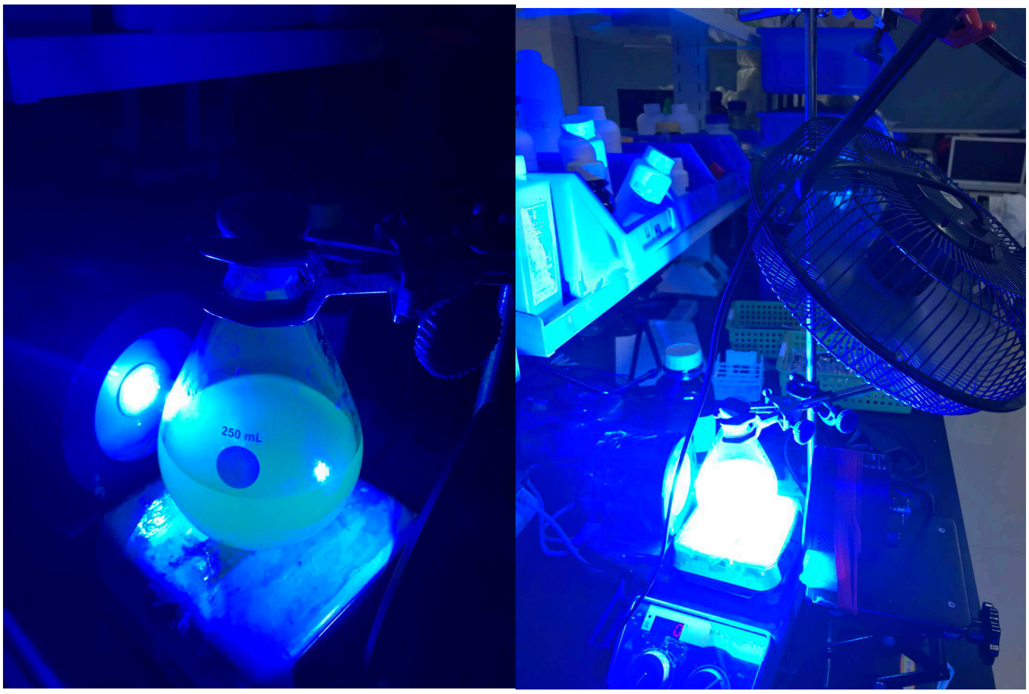

Supplementary Figure 1. Large scale reaction

A 250 mL round bottom flask containing a stirring bar was charged with Ir(ppy)$_3$ (32.7 mg, 0.05 mmol, 1 mol%), 2-cyanopyridine (1.04 g, 10 mmol, 2.0 equiv), 4-chlorobenzenesulfinate (0.99 g, 5 mmol, 1.0 equiv.) and NH_4Cl (0.53 g, 10 mmol, 2.0 equiv.). The flask was capped. After evacuated and backfilled nitrogen three times, MeCN-EtOH (1:1, 125 mL) was added via a syringe, followed by the addition of (E)-prop-1-en-1-ylbenzene (591 mg, 5 mmol, 1.0 equiv.). The reaction mixture was then irradiated with two 90 W blue LED lamps (at approximately 3 cm away from the light source) with cooling from one fan for 24 h. The MeOK (1.75g, 25 mmol, 5 equiv.) was added to the concentrated result mixture which diluted with 100 mL THF. After stirred for another 4 hours, the reaction mixture was concentrated and then quenched with water, extracted with ethyl acetate. The combined organic layers were dried with Mg_2SO_4, filtered, and concentrated in vacuo. The crude material was purified by flash chromatography (PE: EA= 20:1) to afford the product as a glassy yellow oil (644 mg, 66%).

Supplementary Discussion

Electrochemistry.

Cyclic voltammetry experiments were performed on a CHI600E Workstation. Scan Rate: 0.5 V/s. Glassy carbon electrode working electrode, Ag/AgCl reference electrode, Pt wire counter electrode. The irreversible peak at 0.477 V correspond to the oxidation of sodium methanesulfinate. $E_{1/2}$(Fc/Fc+) = 0.380 V, E_{OX}= 0.497 V versus SCE in [0.1 M] $TBAPF_6$ in CH_3CN.

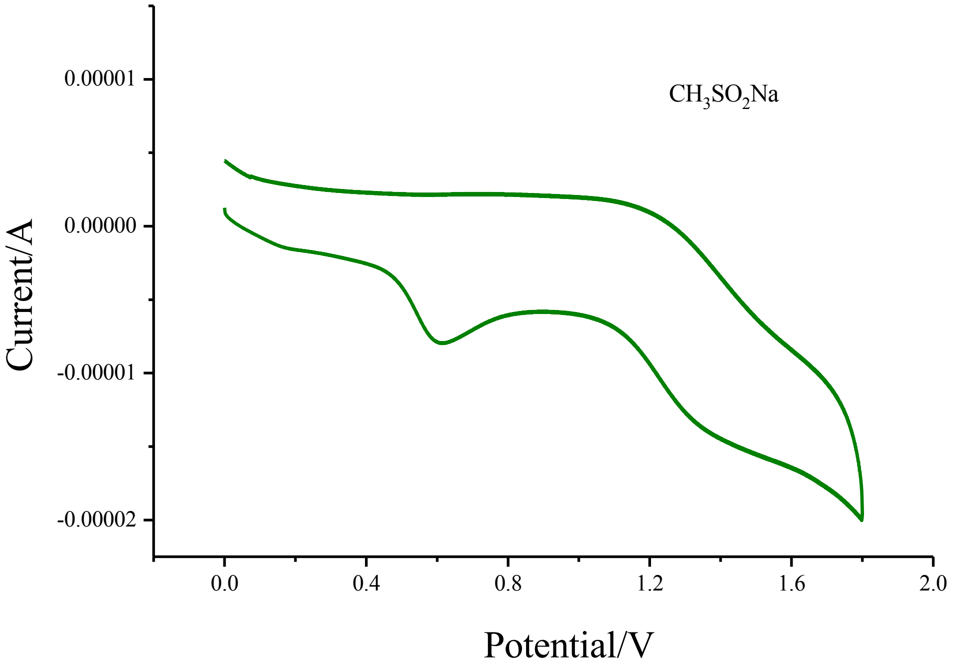

Supplementary Figure 2. Cyclic voltammogram of CH_3SO_2Na.

Luminescence quenching experiments.

Emission intensities were recorded using a Perkin Elmer LS50 Luminescence spectrometer. All $Ir(ppy)_3$ solutions were excited at 385 nm and the emission intensity at 517 nm was observed. In a typical experiment, a 1.0×10^{-4} M solution of $Ir(ppy)_3$ in acetonitrile-ethanol (1:1) was added to the appropriate amount of quencher in a screw-top 1.0 cm quartz cuvette. After degassing with a stream of nitrogen for 10 minutes, the emission spectrum of the sample was collected.

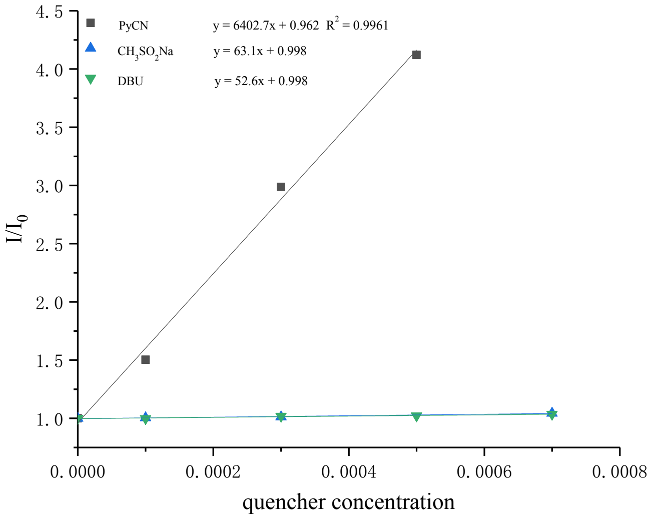

Supplementary Figure 3. Stern-Volmer quenching study.

Radical clock experiments.

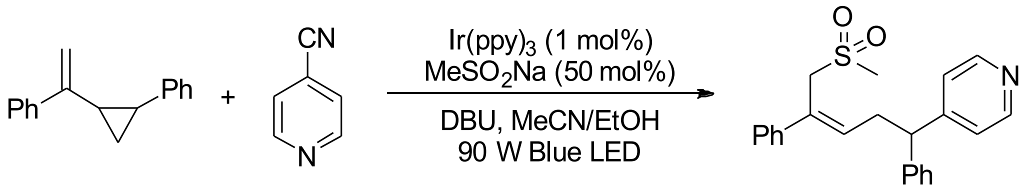

According to the **GPA**, (1-(2-phenylcyclopropyl)vinyl)benzene (22.0 mg, 0.10 mmol, 1.0 equiv.), 4-cyanopyridine (20.8 mg, 0.20 mmol, 2.0 equiv.), sodium methanesulfinate (5.1 mg, 0.05 mmol, 50 mol%), Ir(ppy)$_3$ (0.7 mg, 0.001 mmol, 1 mol%), and 1,8-diazabicyclo[5.4.0]undec-7-ene (44.8 μL, 0.3 mmol, 3.0 equiv.) in MeCN-EtOH (1:1, 5 mL) were used. After 24 h, the result mixture was concentrated in vacuo and the product was isolated by flash chromatography (DCM: MeOH= 100:1) as a pale-yellow oil (12.8 mg, 34%).

(Z)-4-(5-(methylsulfonyl)-1,4-diphenylpent-3-en-1-yl)pyridine (S5): ^{1}H NMR (400 MHz, $CDCl_3$) δ 8.45 (d, J = 5.6 Hz, 2H), 7.48 – 7.17 (m, 6H), 7.10 – 7.06 (m, 2H), 7.01 (d, J = 5.9 Hz, 2H), 5.85 (t, J = 7.2 Hz, 1H), 4.02 (t, J = 7.8 Hz, 1H), 3.91 (s, 2H), 2.85 (t, J = 7.5 Hz, 2H), 2.46 (s, 3H). ^{13}C NMR (150 MHz, $CDCl_3$) δ 152.67, 149.78, 141.85, 137.71, 135.48, 130.64, 128.79, 128.76, 128.52, 128.09, 127.96, 127.04, 123.28, 64.09,

50.44, 40.62, 34.70. HRMS (ESI): calcd for $C_{23}H_{24}NO_2S^+$ (M+H) 378.1512, found 378.1522.

Radical inhibition experiments.

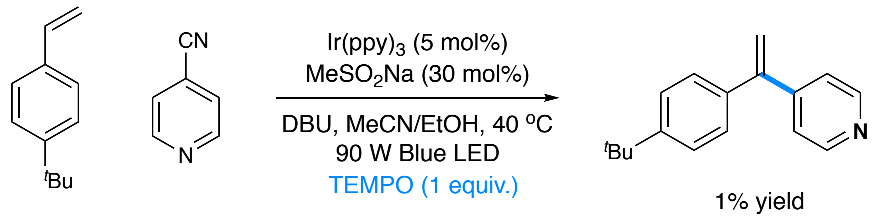

According to the **GPA**, 4-*tert*-butylstyrene (18.0 μL, 0.10 mmol, 1.0 equiv.), 4-cyanopyridine (20.8 mg, 0.20 mmol, 2.0 equiv.), sodium methanesulfinate (5.1 mg, 0.05 mmol, 50 mol%), $Ir(ppy)_3$ (0.7 mg, 0.001 mmol, 1 mol%), and 1,8-diazabicyclo[5.4.0]undec-7-ene (44.8 μL, 0.3 mmol, 3.0 equiv.), TEMPO (15.6 mg, 0.1 mmol, 1.0 equiv.) in MeCN-EtOH (1:1, 5 mL) were used. After 24 h, the result mixture was analyzed by GC with dodecane as internal standard.

Isolation of alkyl sulfone intermediate.

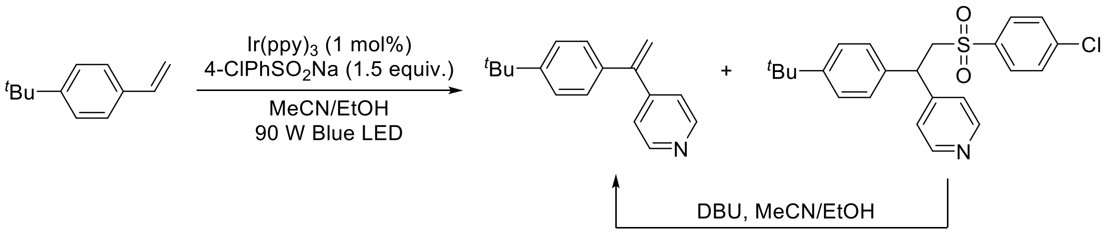

According to the general procedure, 4-*tert*-butylstyrene (90.0 μL, 0.5 mmol, 1.0 equiv.), 4-cyanopyridine (104.2 mg, 1.0 mmol, 2.0 equiv.), 4-cholrobenzenesulfinate (149.0 mg, 0.75 mmol, 1.5 equiv.), $Ir(ppy)_3$ (3.3 mg, 0.005 mmol, 1 mol%) in MeCN-EtOH (1:1, 5 mL) were used. After 24 h, the result mixture was concentrated under vacuum and the residue was separated by flash chromatography (PE:EA = 5:1) to give the alkene as a colorless oil (28.4 mg, 24%) and the sulfone as a white powder (134.6 mg, 65%).

4-(1-(4-(tert-butyl)phenyl)-2-((4-chlorophenyl)sulfonyl)ethyl)pyridine (mg, 0.1 mmol, 1.0 equiv.) and DBU (mg, 0.3 mmol, 3.0 equiv.) were dissolved in MeCN-EtOH (1:1, 5 mL). After stirred at 40 °C for 24 h, the result mixture was concentrated under vacuum

and the product was isolated by flash chromatography (PE:EA = 10:1) as a colorless oil (22.3 mg, 94 %).

4-(1-(4-(tert-butyl)phenyl)-2-((4-chlorophenyl)sulfonyl)ethyl)pyridine (S6): ^{1}H NMR (600 MHz, $CDCl_3$) δ 8.45 (d, *J* = 5.6 Hz, 2H), 7.52 (d, *J* = 8.5 Hz, 2H), 7.26 (d, *J* = 8.5 Hz, 2H), 7.17 (d, *J* = 8.3 Hz, 2H), 7.08 (d, *J* = 5.9 Hz, 2H), 6.95 (d, *J* = 8.3 Hz, 2H), 4.56 (t, *J* = 7.1 Hz, 1H), 3.89 (ddd, *J* = 21.2, 14.8, 7.2 Hz, 2H), 1.25 (s, 9H). ^{13}C NMR (150 MHz, $CDCl_3$) δ 150.77, 150.35, 150.11, 140.18, 137.76, 135.86, 129.41, 129.18, 127.24, 125.92, 122.68, 60.73, 45.32, 34.44, 31.27. HRMS (ESI): calcd for $C_{23}H_{25}ClNO_2S^+$ (M+H) 414.1295, found 414.1301.

Alternative mechanism.

Due to the fact that a trace amount of C2-substituted product **13'** is observed under our photocatalytic conditions, alternative pathway involving the S_NAr reaction of cyanopyridine with benzylic radical is provided below:

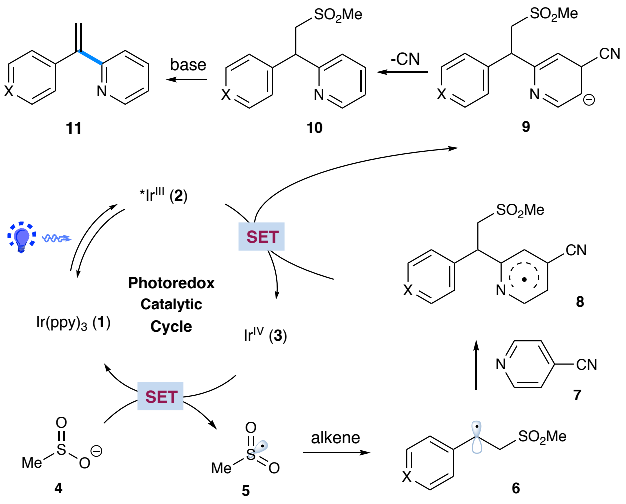

Supplementary Figure 4. Alternative mechanism.

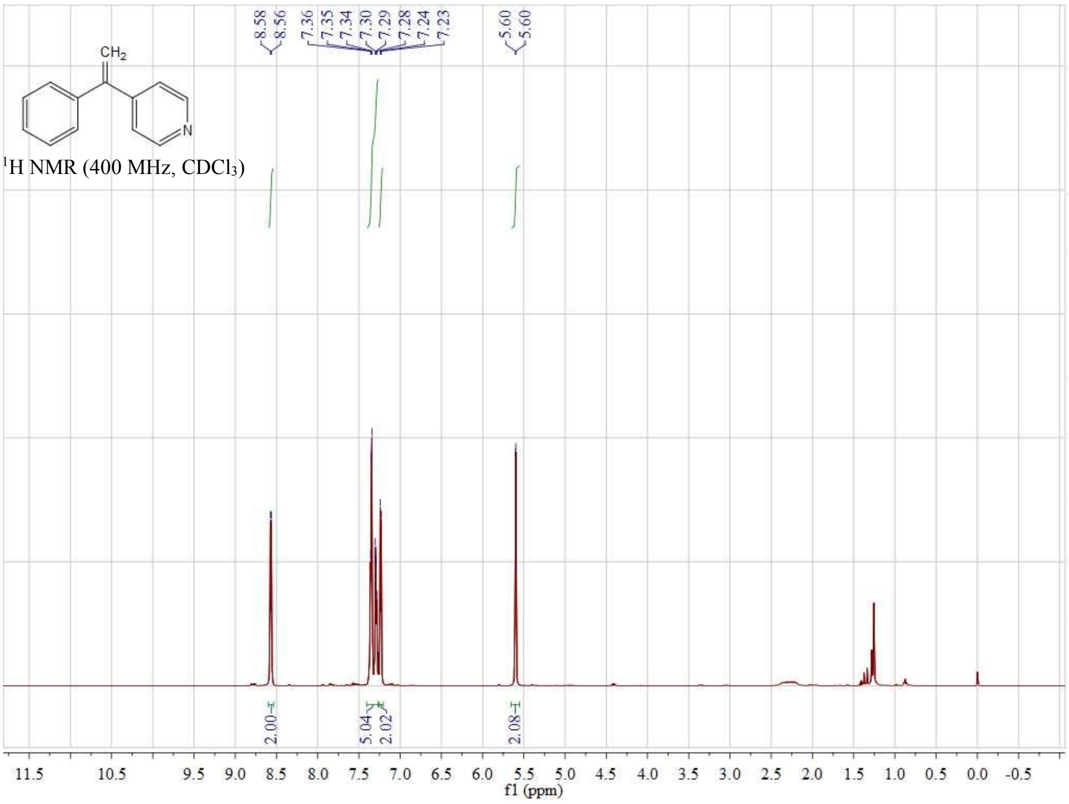

Supplementary Figure 5: ^{1}H NMR Spectra of 4-(1-Phenylvinyl)pyridine (14)

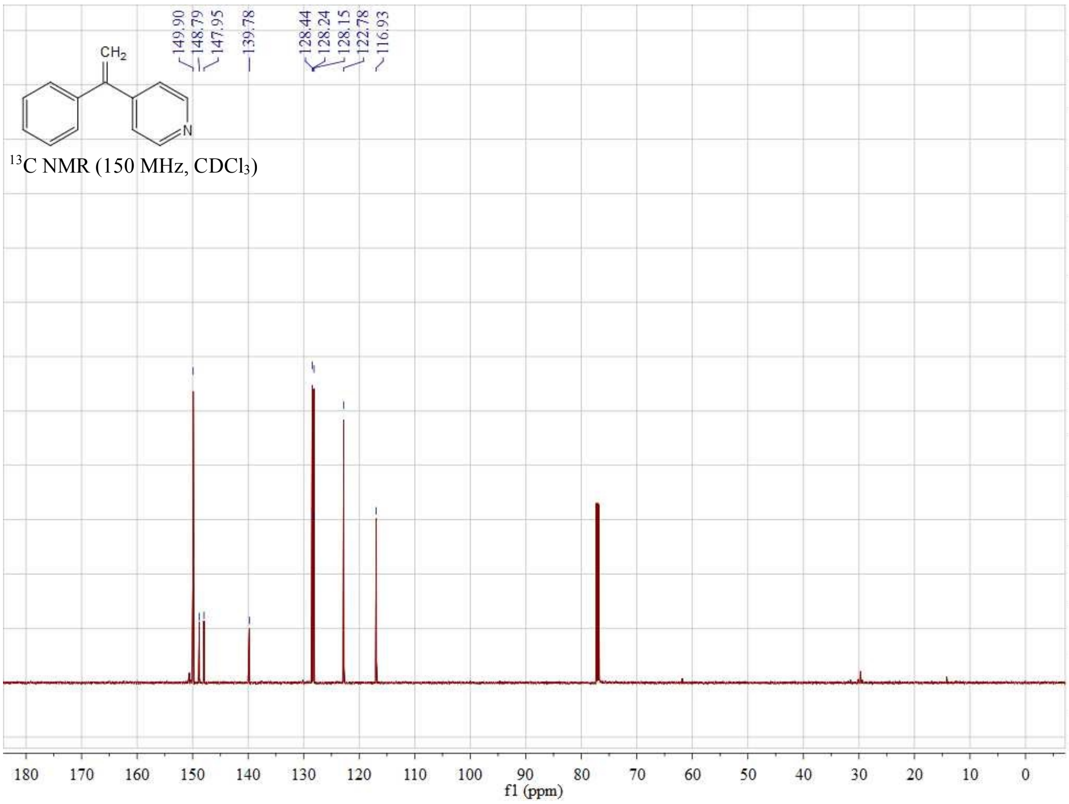

Supplementary Figure 6: ^{13}C NMR Spectra of 4-(1-Phenylvinyl)pyridine (14)

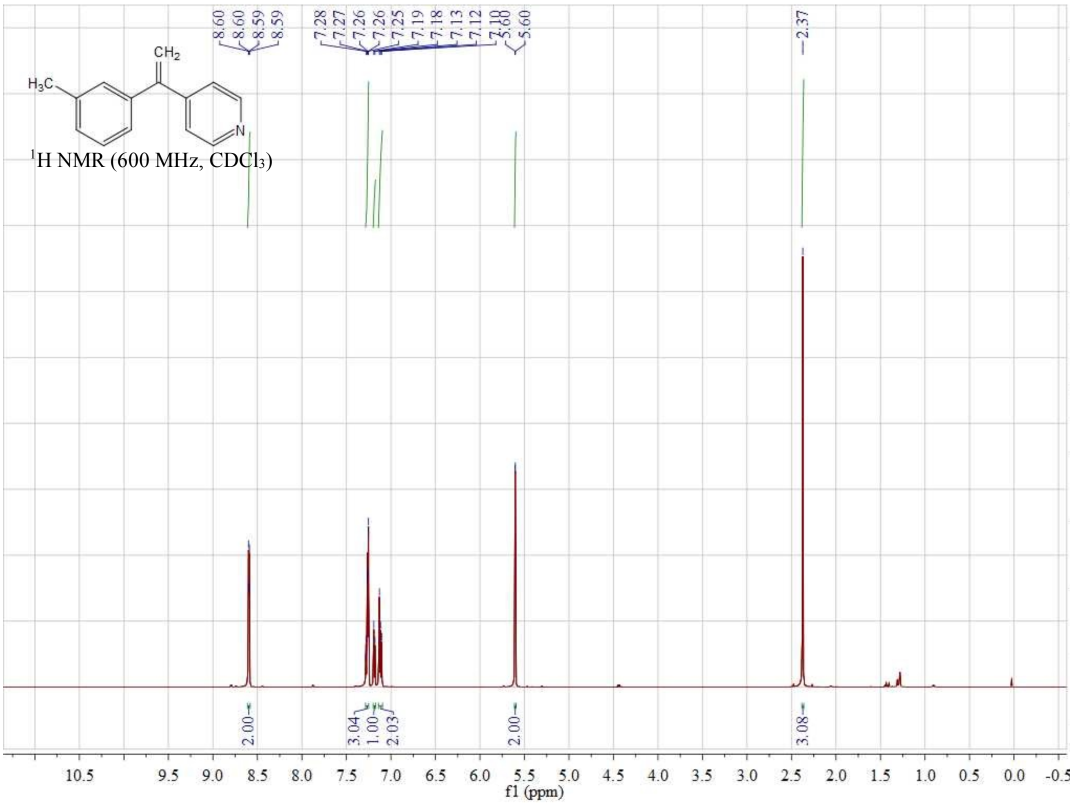

Supplementary Figure 7: ^{1}H NMR Spectra of 4-(1-(m-Tolyl)vinyl)pyridine (15)

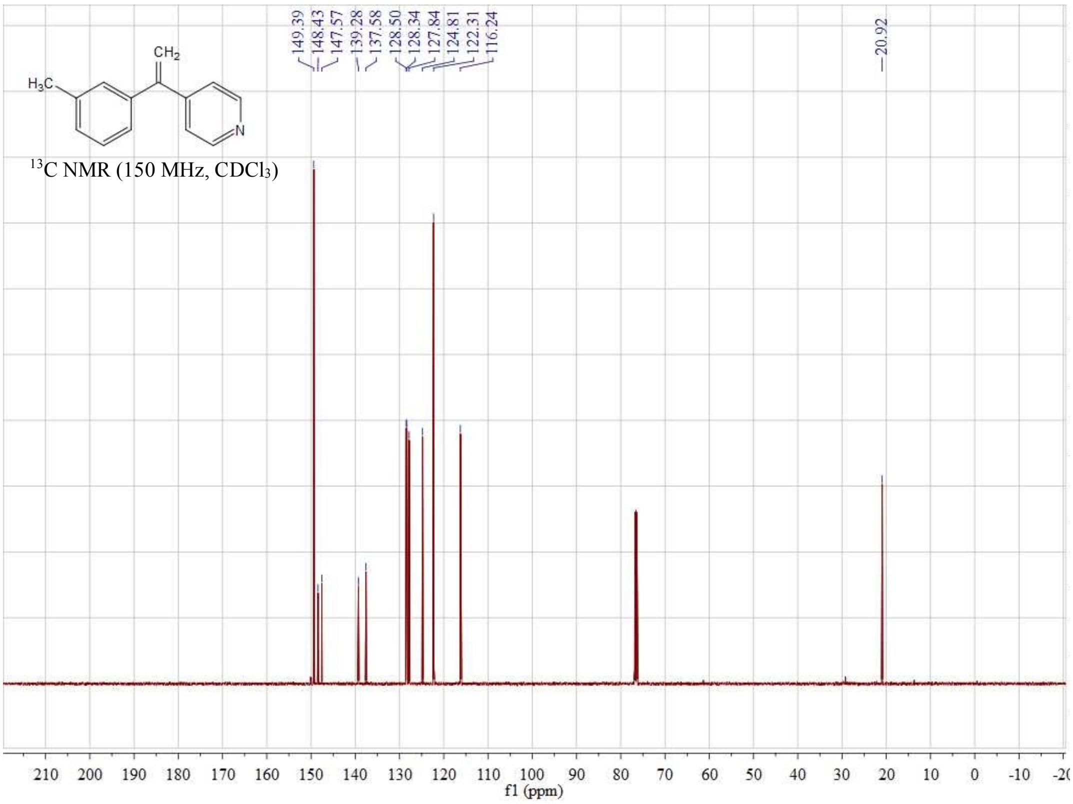

Supplementary Figure 8: ^{13}C NMR Spectra of 4-(1-(m-Tolyl)vinyl)pyridine (15)

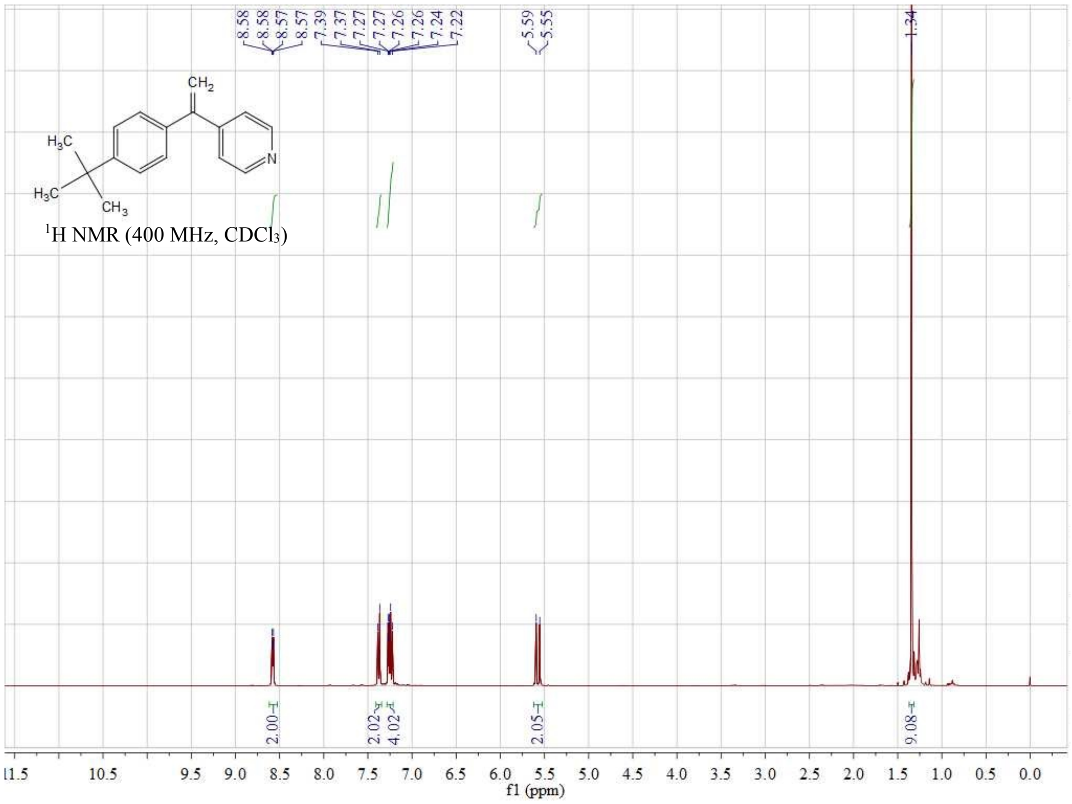

Supplementary Figure 9: ^{1}H NMR Spectra of 4-(1-(4-(*tert*-Butyl)phenyl)vinyl) pyridine (13)

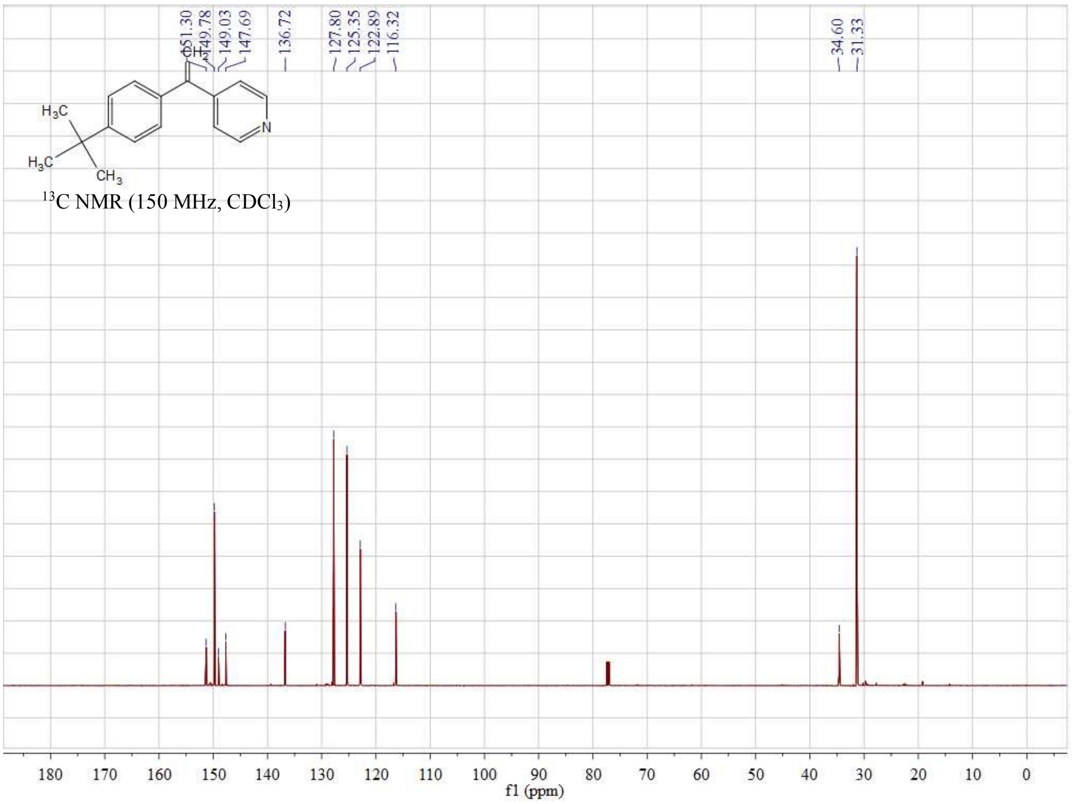

Supplementary Figure 10: ^{13}C NMR Spectra of 4-(1-(4-(*tert*-Butyl)phenyl)vinyl) pyridine (13)

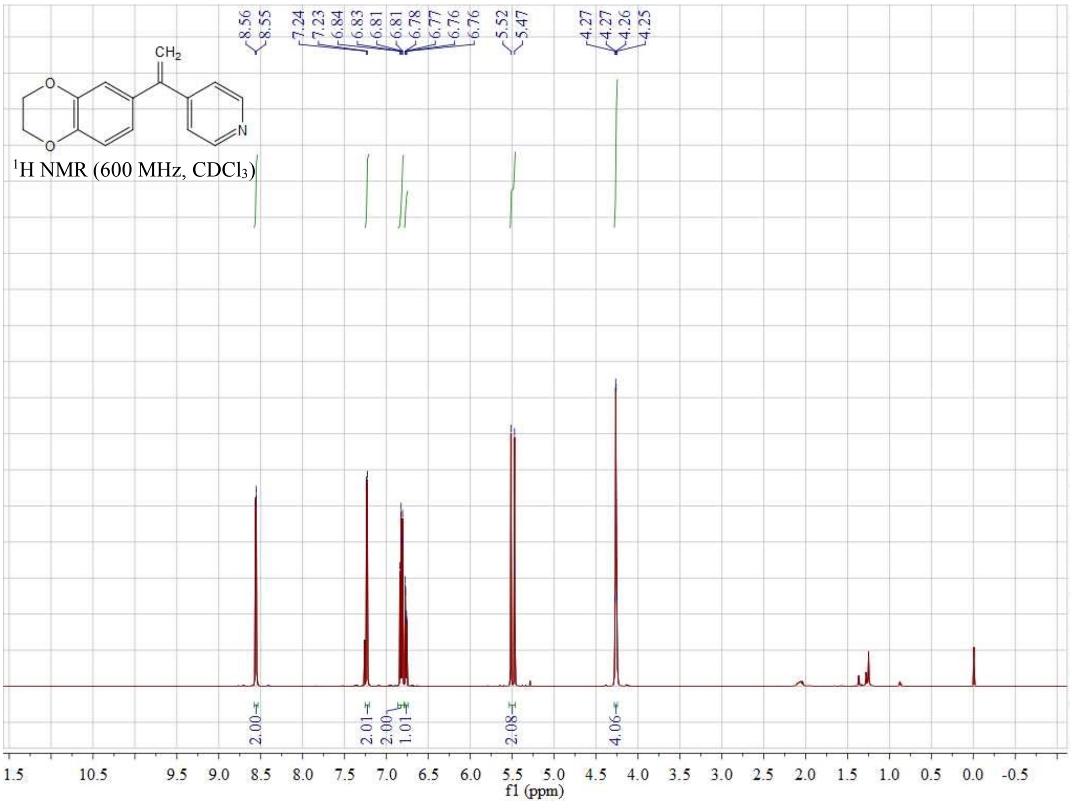

Supplementary Figure 11: ^{1}H NMR Spectra of 4-(1-(2,3-Dihydrobenzo[b][1,4] dioxin-6-yl)vinyl)pyridine (16)

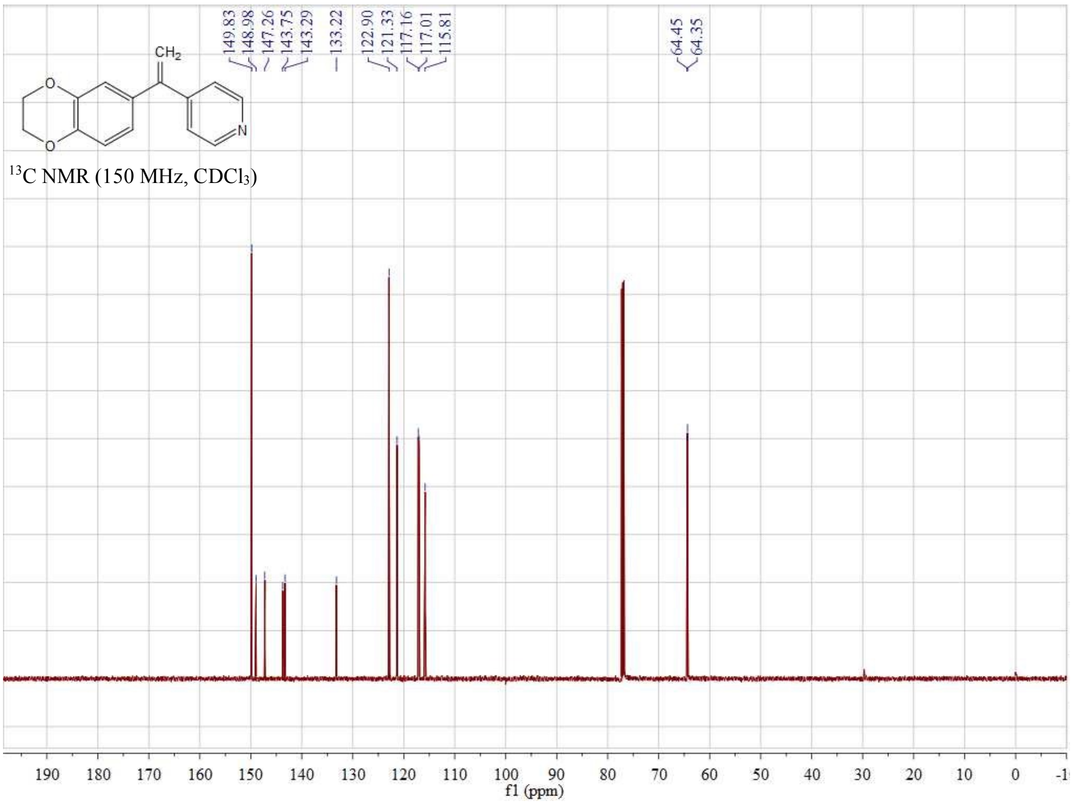

Supplementary Figure 12: ^{13}C NMR Spectra of 4-(1-(2,3-Dihydrobenzo[b][1,4] dioxin-6-yl)vinyl)pyridine (16)

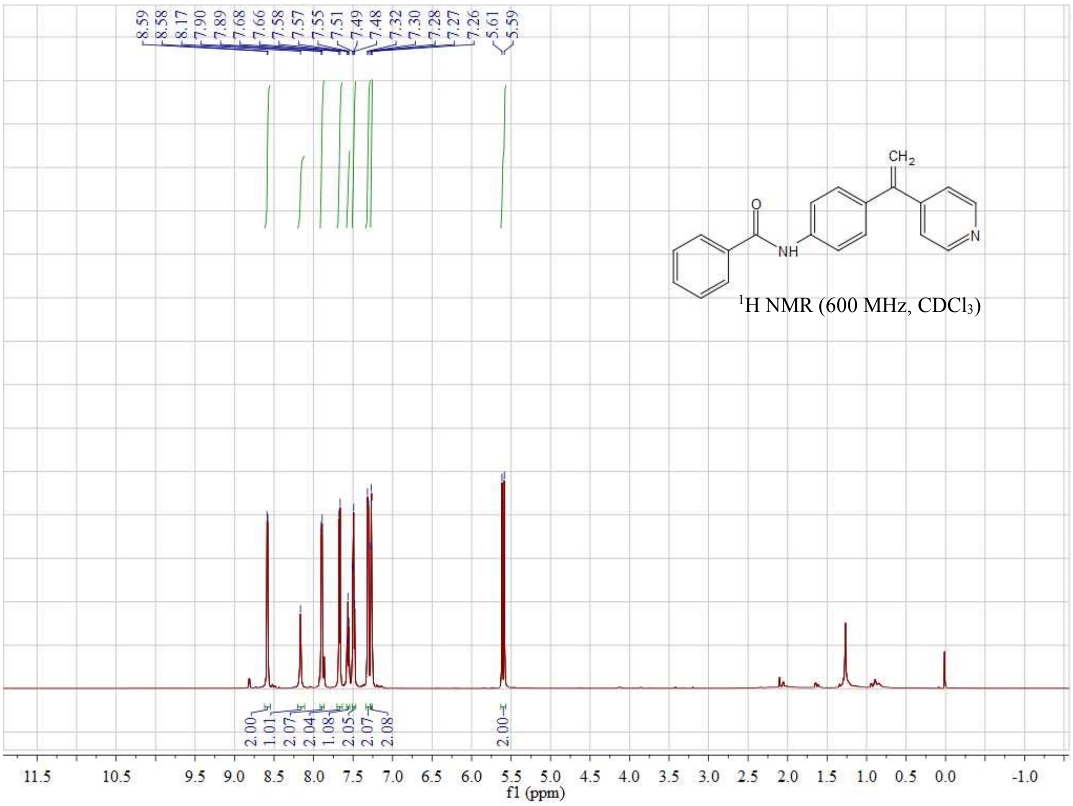

Supplementary Figure 13: ^{1}H NMR Spectra of N-(4-(1-(pyridin-4-yl)vinyl)phenyl) benzamide (17)

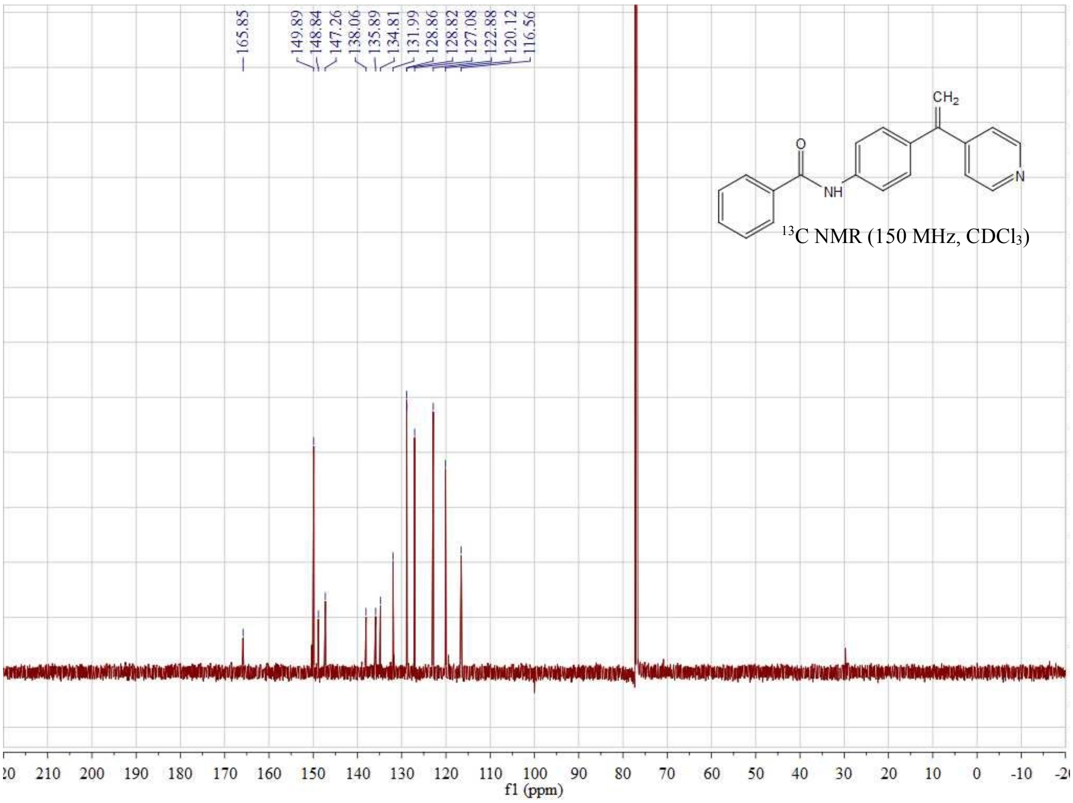

Supplementary Figure 14: ^{13}C NMR Spectra of N-(4-(1-(pyridin-4-yl)vinyl) phenyl)benzamide (17)

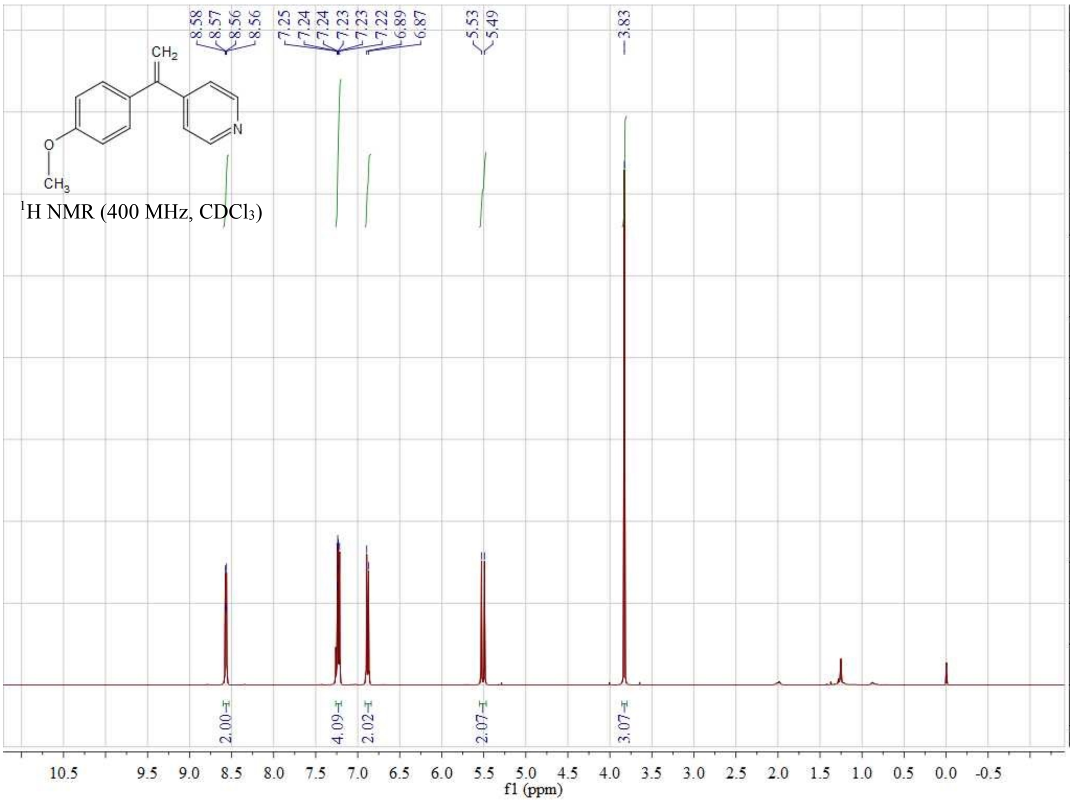

Supplementary Figure 15: ^{1}H NMR Spectra of 4-(1-Phenylvinyl)pyridine (18)

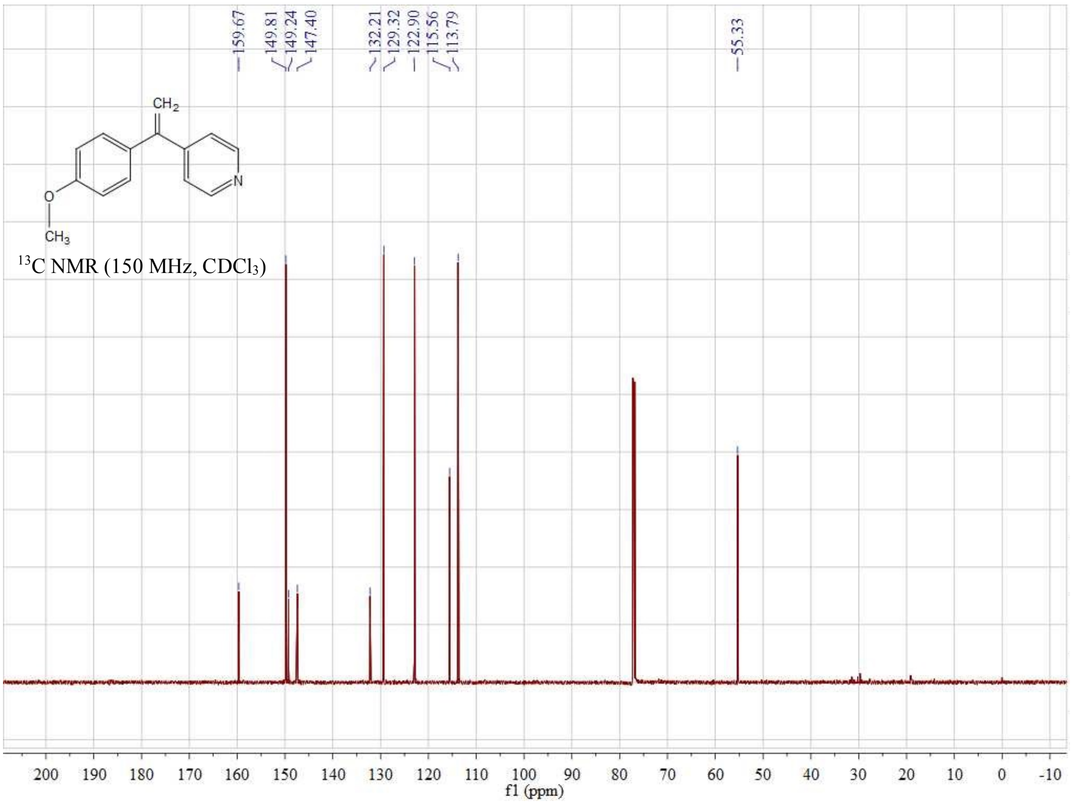

Supplementary Figure 16: ^{13}C NMR Spectra of 4-(1-Phenylvinyl)pyridine (18)

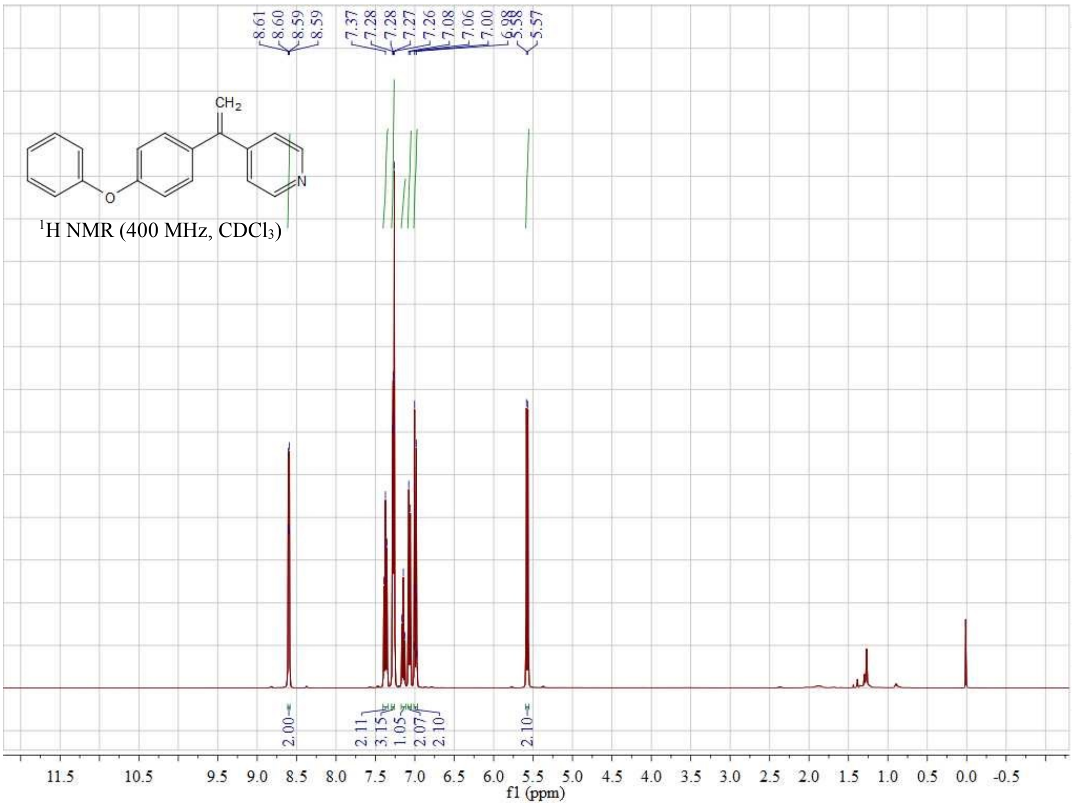

Supplementary Figure 17: ^{1}H NMR Spectra of 4-(1-(4-Phenoxyphenyl)vinyl) pyridine (19)

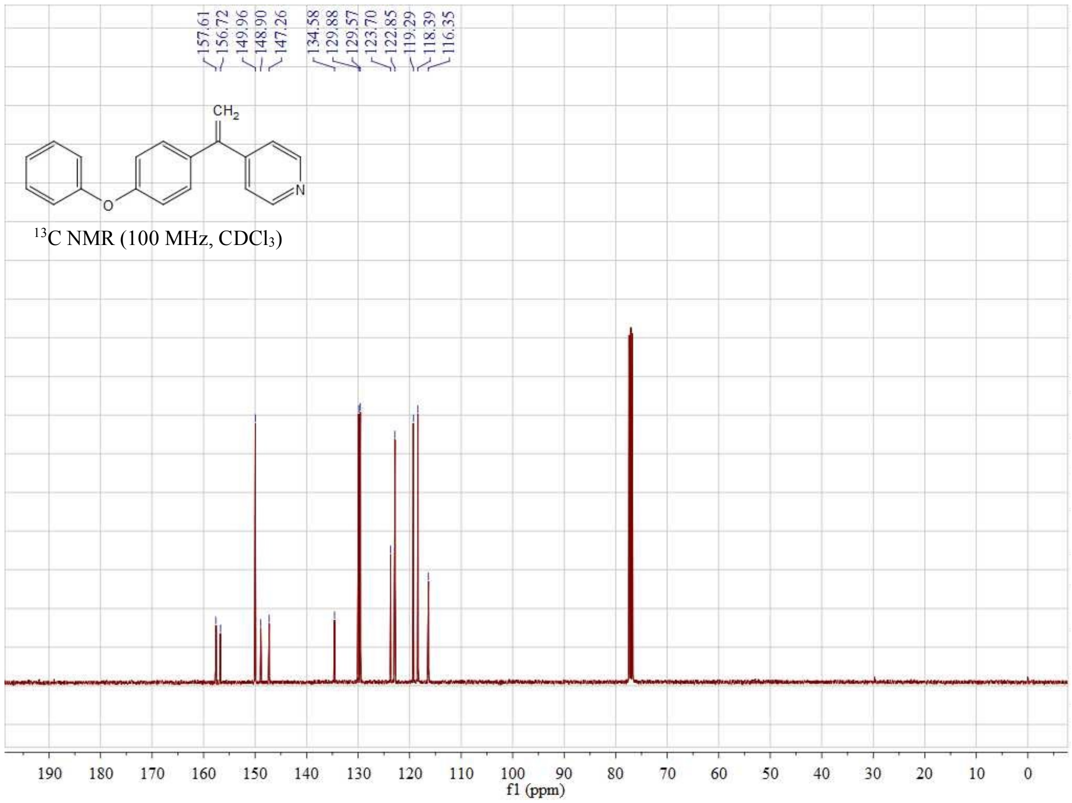

Supplementary Figure 18: ^{13}C NMR Spectra of 4-(1-(4-Phenoxyphenyl)vinyl) pyridine (19)

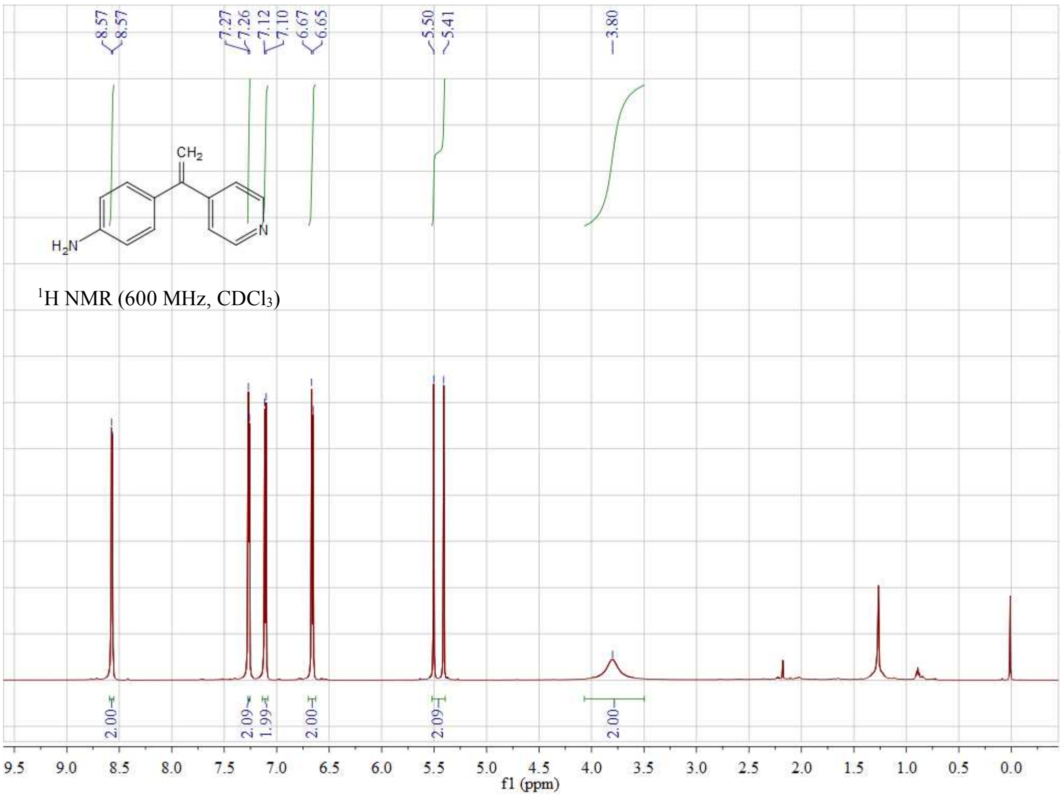

Supplementary Figure 19: ^{1}H NMR Spectra of 4-(1-(pyridin-4-yl)vinyl)aniline (20)

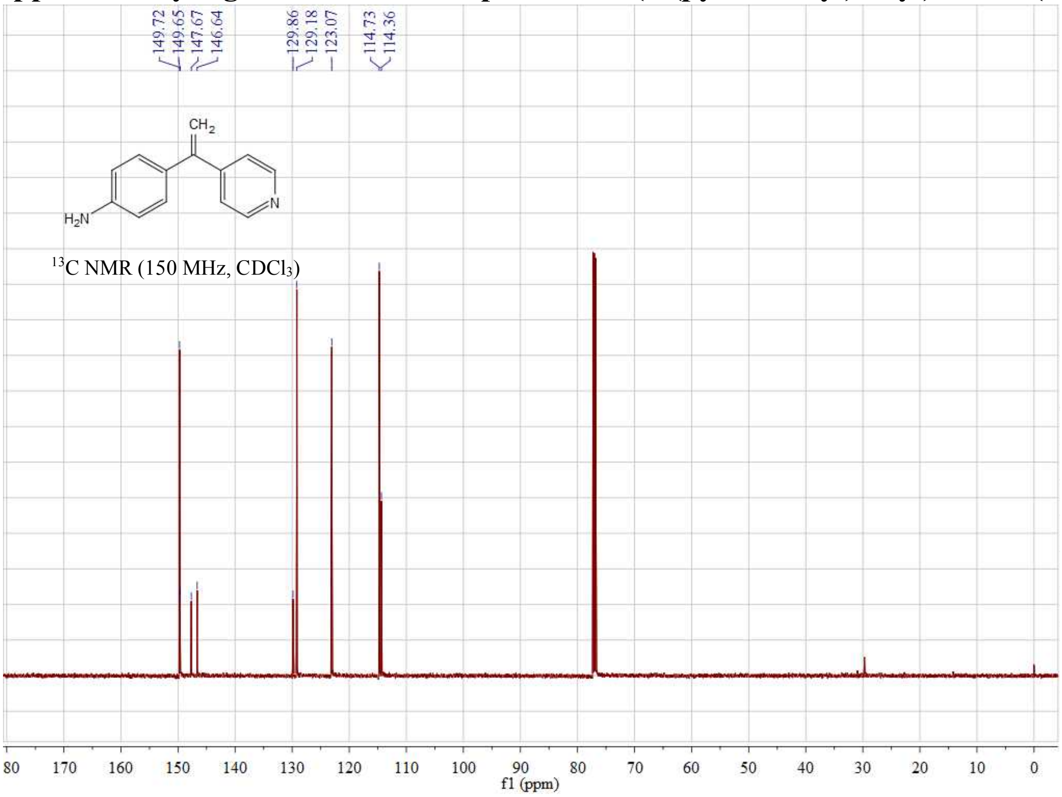

Supplementary Figure 20: ^{13}C NMR Spectra of 4-(1-(pyridin-4-yl)vinyl)aniline (20)

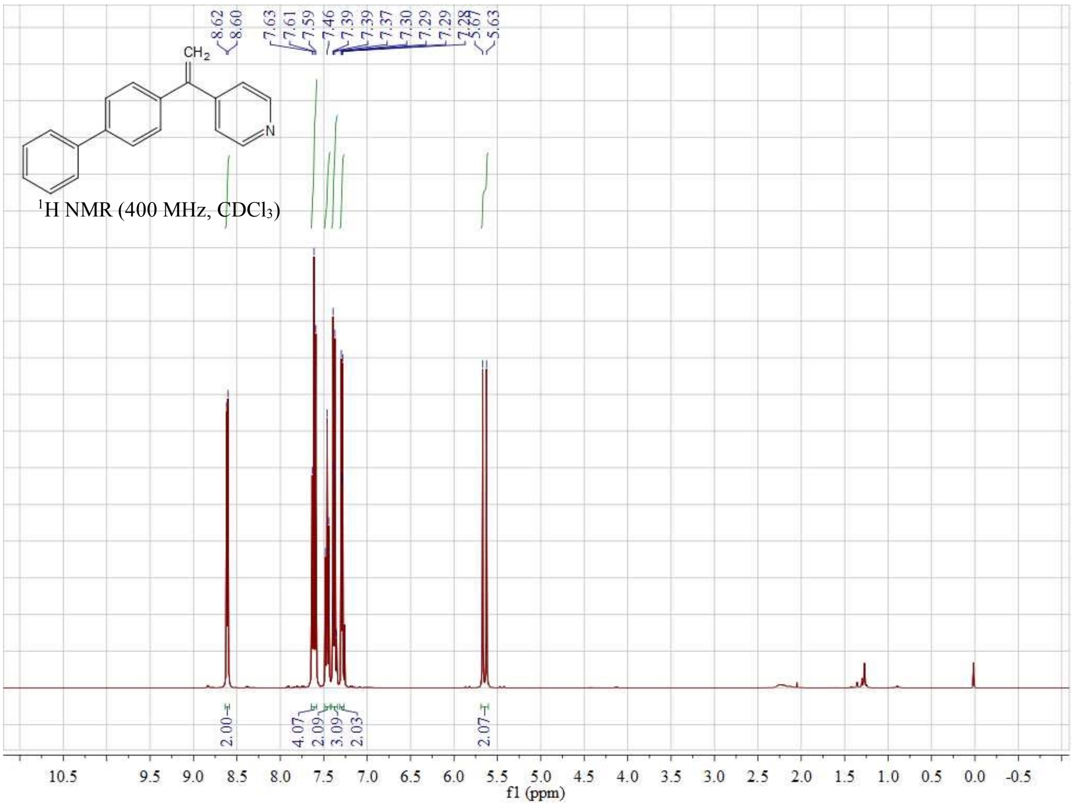

Supplementary Figure 21: ^{1}H NMR Spectra of 4-(1-([1,1'-Biphenyl]-4-yl)vinyl)pyridine (21)

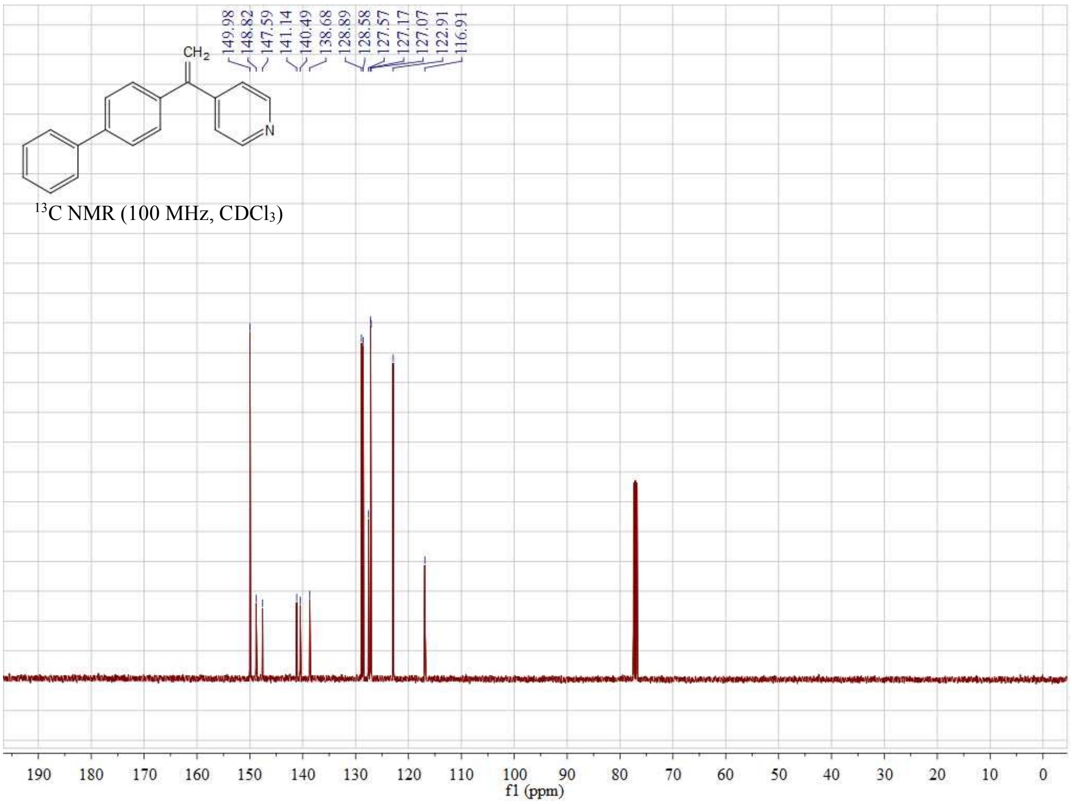

Supplementary Figure 22: ^{13}C NMR Spectra of 4-(1-([1,1'-Biphenyl]-4-yl)vinyl)pyridine (21)

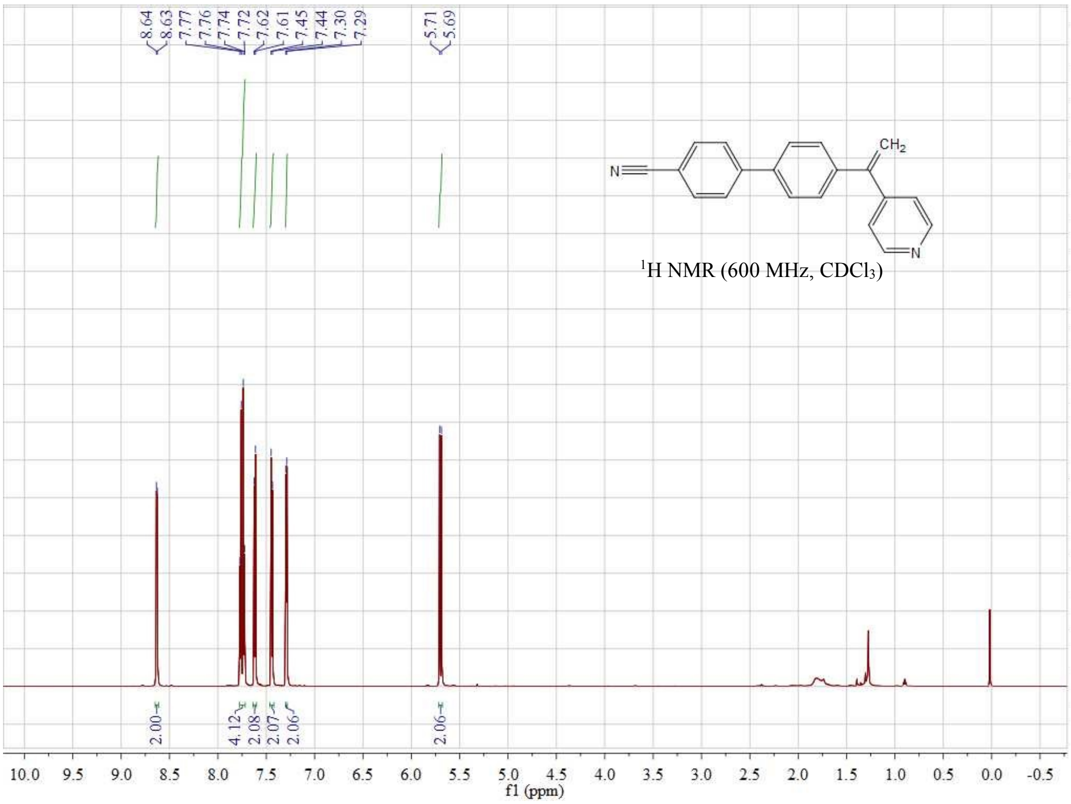

Supplementary Figure 23: ^{1}H NMR Spectra of 4'-(1-(Pyridin-4-yl)vinyl)-[1,1'-biphenyl]-4-carbonitrile (22)

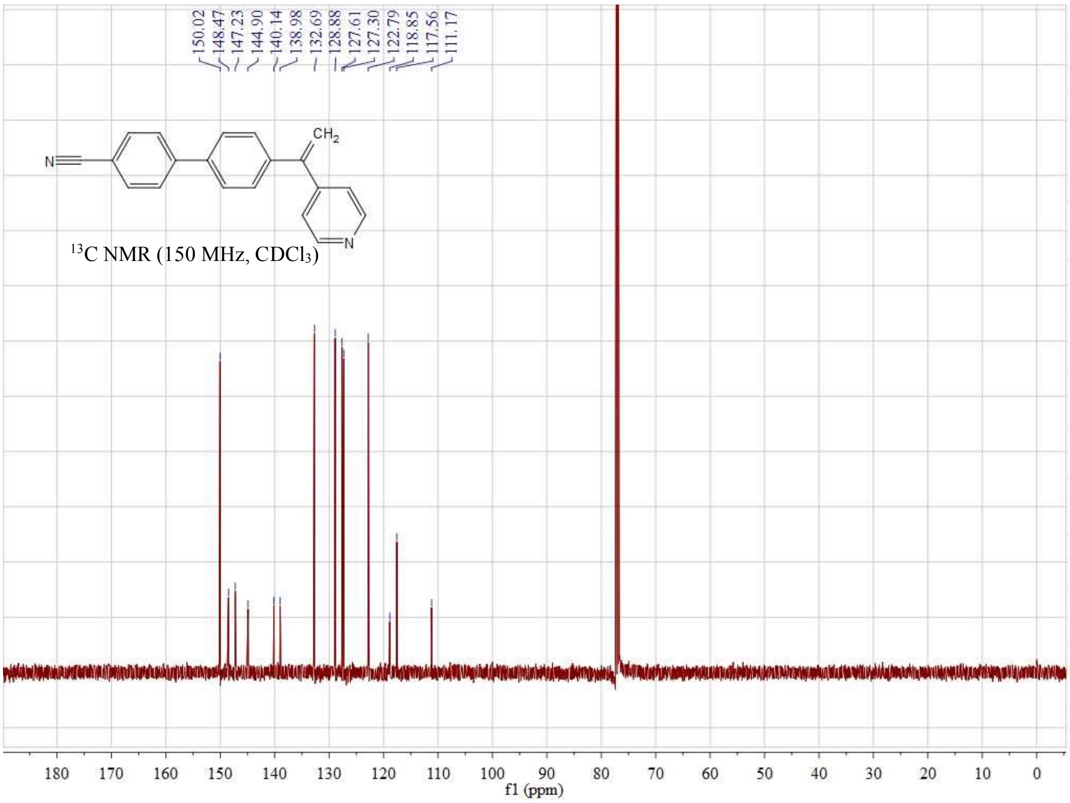

Supplementary Figure 24: ^{13}C NMR Spectra of 4'-(1-(Pyridin-4-yl)vinyl)-[1,1'-biphenyl]-4-carbonitrile (22)

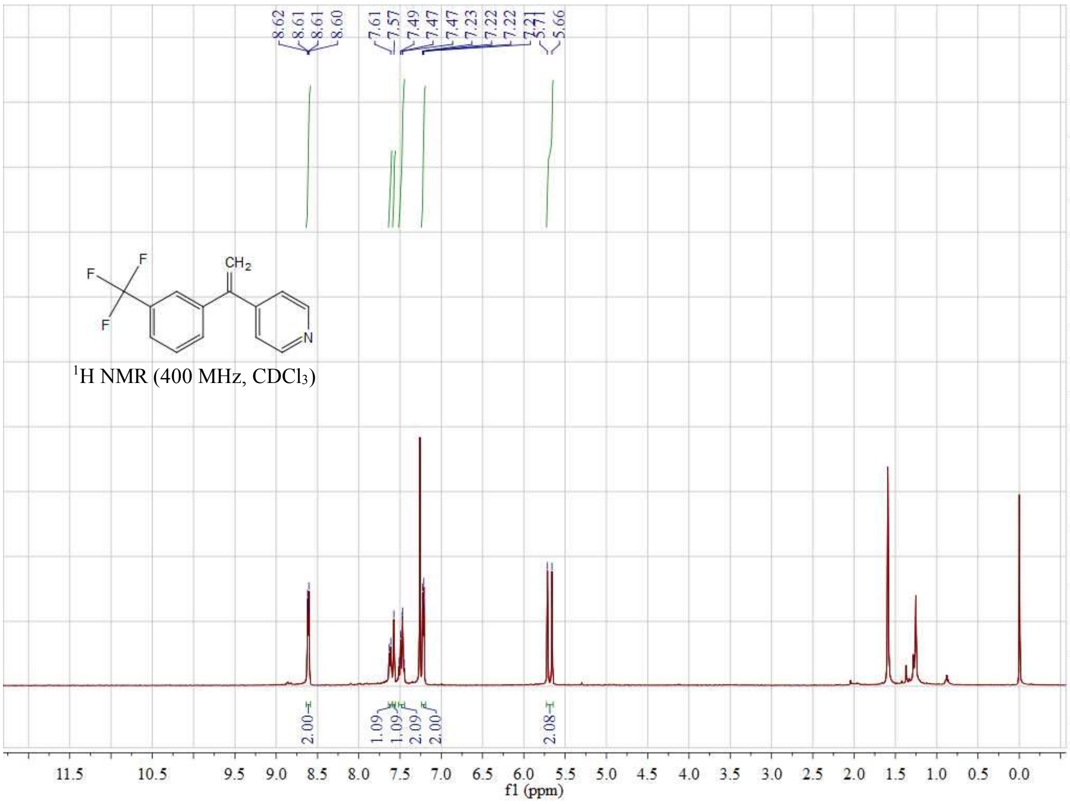

Supplementary Figure 25: ^{1}H NMR Spectra of 4-(1-(3-(Trifluoromethyl)phenyl) vinyl)pyridine (23)

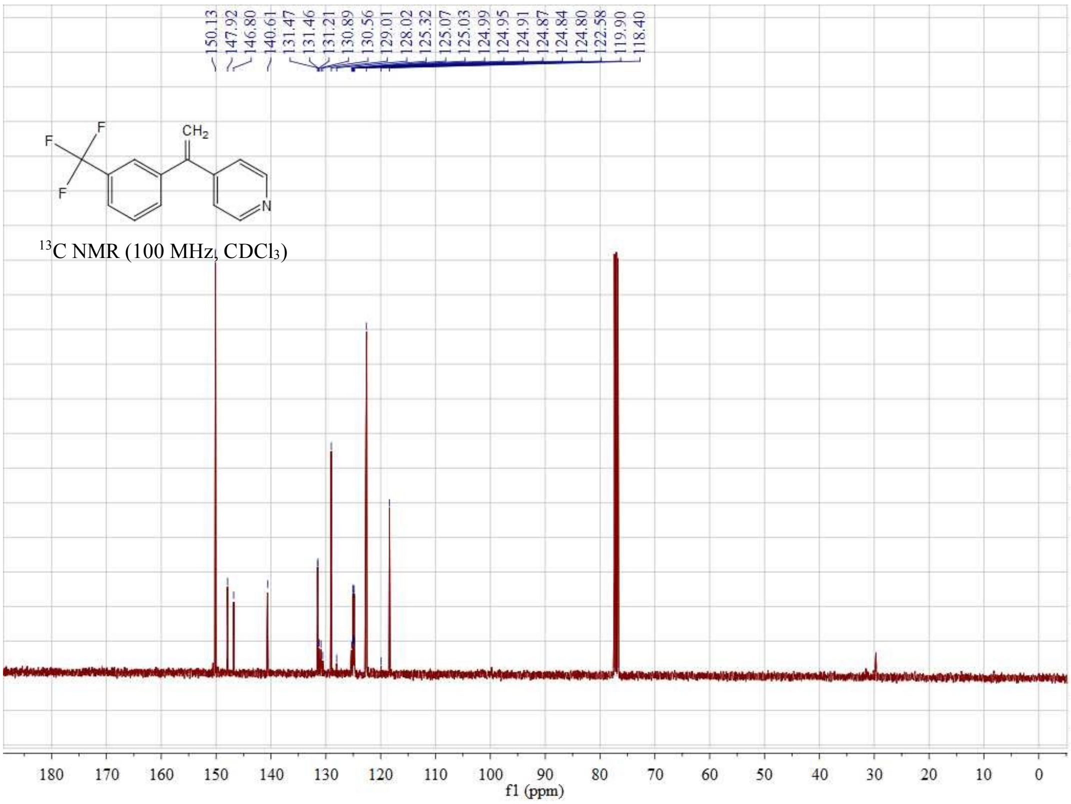

Supplementary Figure 26: ^{13}C NMR Spectra of 4-(1-(3-(Trifluoromethyl)phenyl) vinyl)pyridine (23)

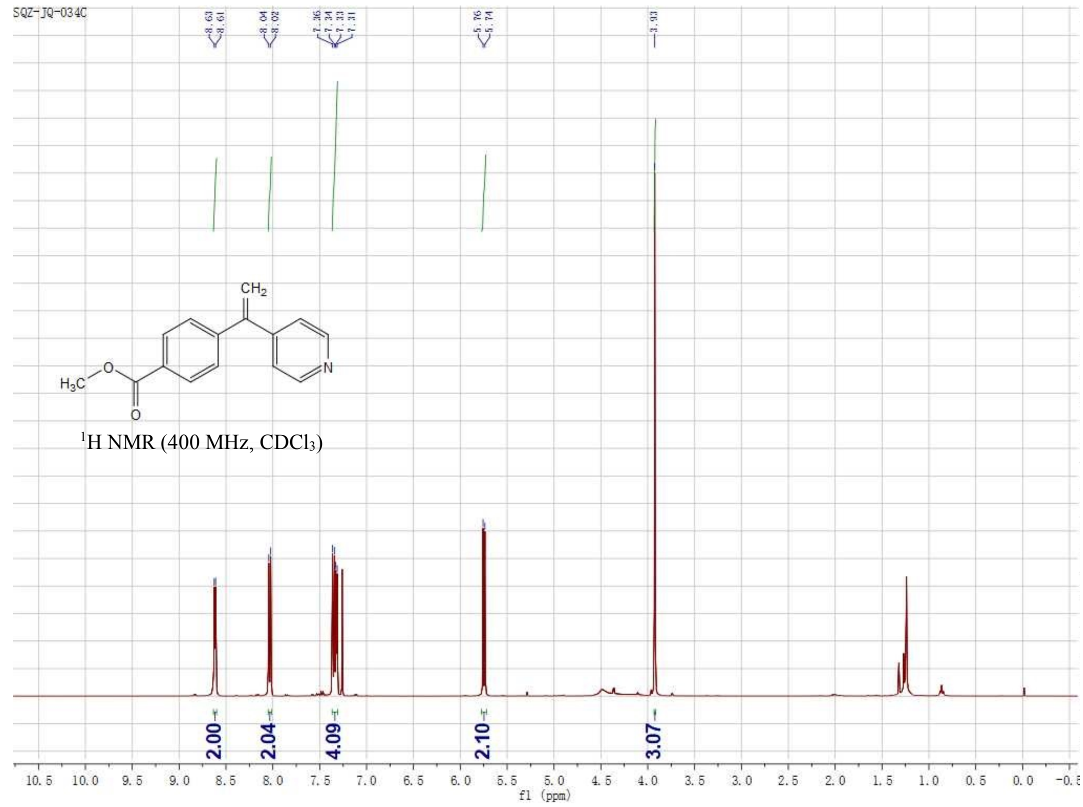

Supplementary Figure 27: ^{1}H NMR Spectra of Methyl 4-(1-(pyridin-4-yl)vinyl)benzoate (24)

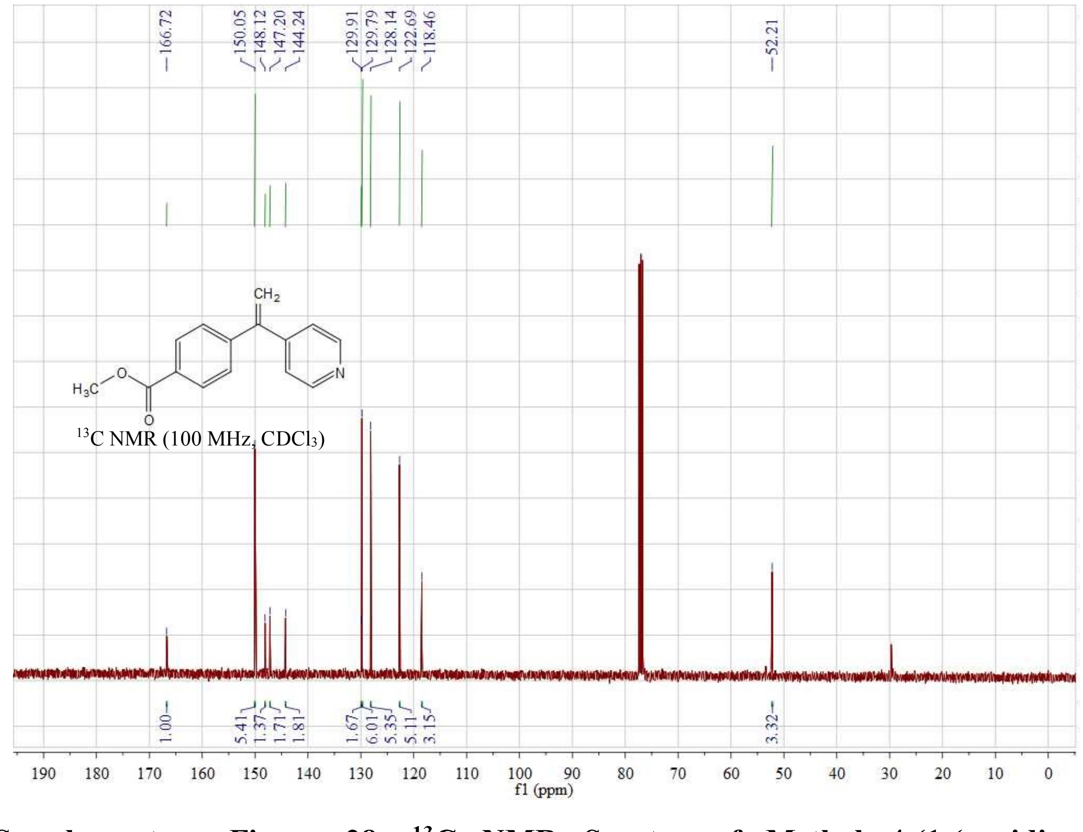

Supplementary Figure 28: ^{13}C NMR Spectra of Methyl 4-(1-(pyridin-4-yl)vinyl)benzoate (24)

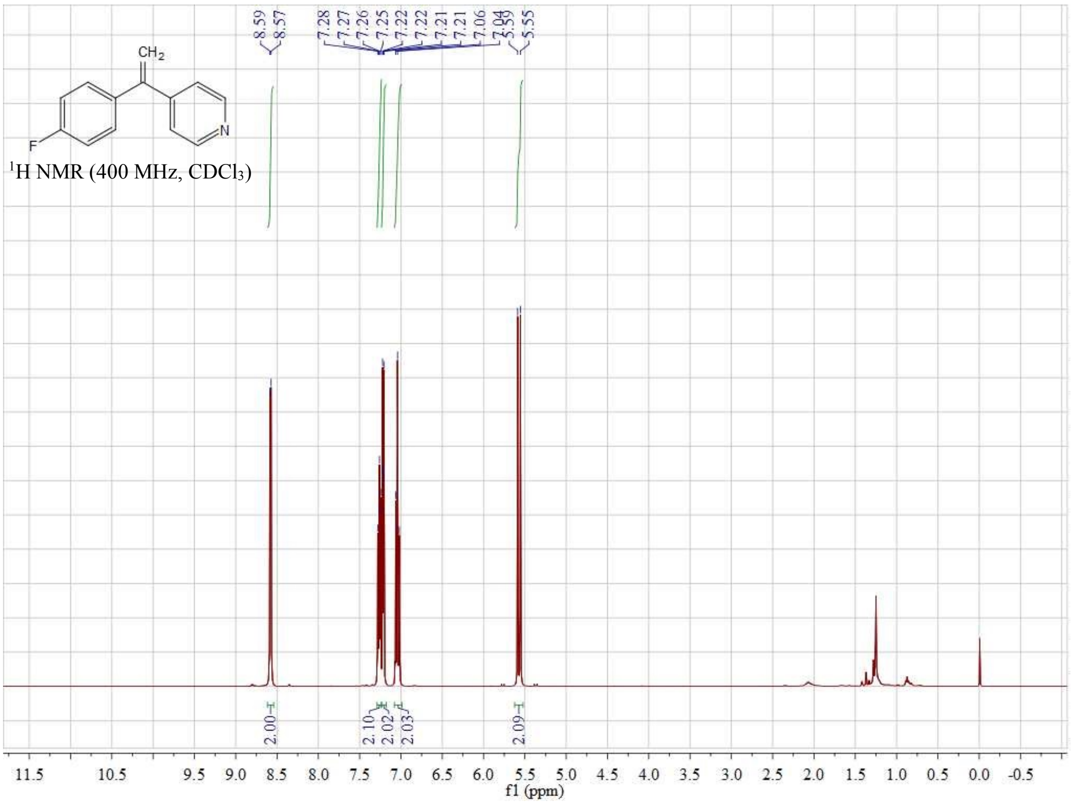

Supplementary Figure 29: ^{1}H NMR Spectra of 4-(1-(4-Fluorophenyl) vinyl)pyridine (25)

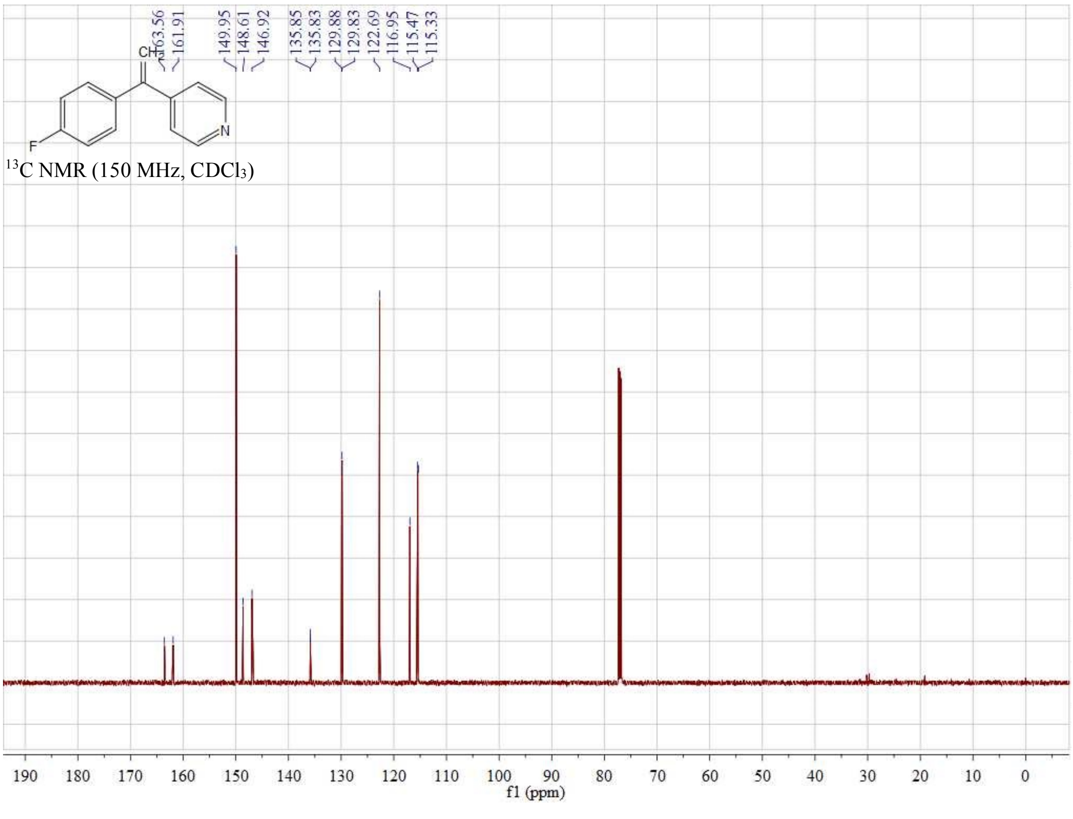

Supplementary Figure 30: ^{13}C NMR Spectra of 4-(1-(4-Fluorophenyl) vinyl)pyridine (25)

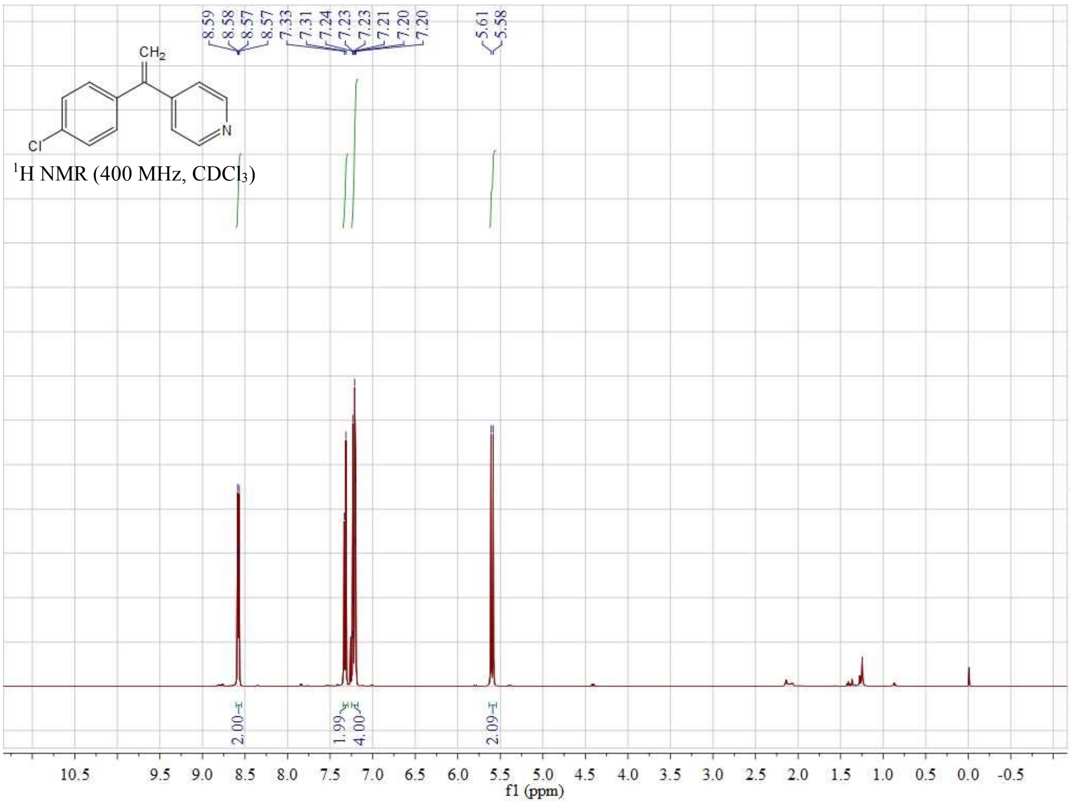

Supplementary Figure 31: ^{1}H NMR Spectra of 4-(1-(4-Chlorophenyl)vinyl) pyridine (26)

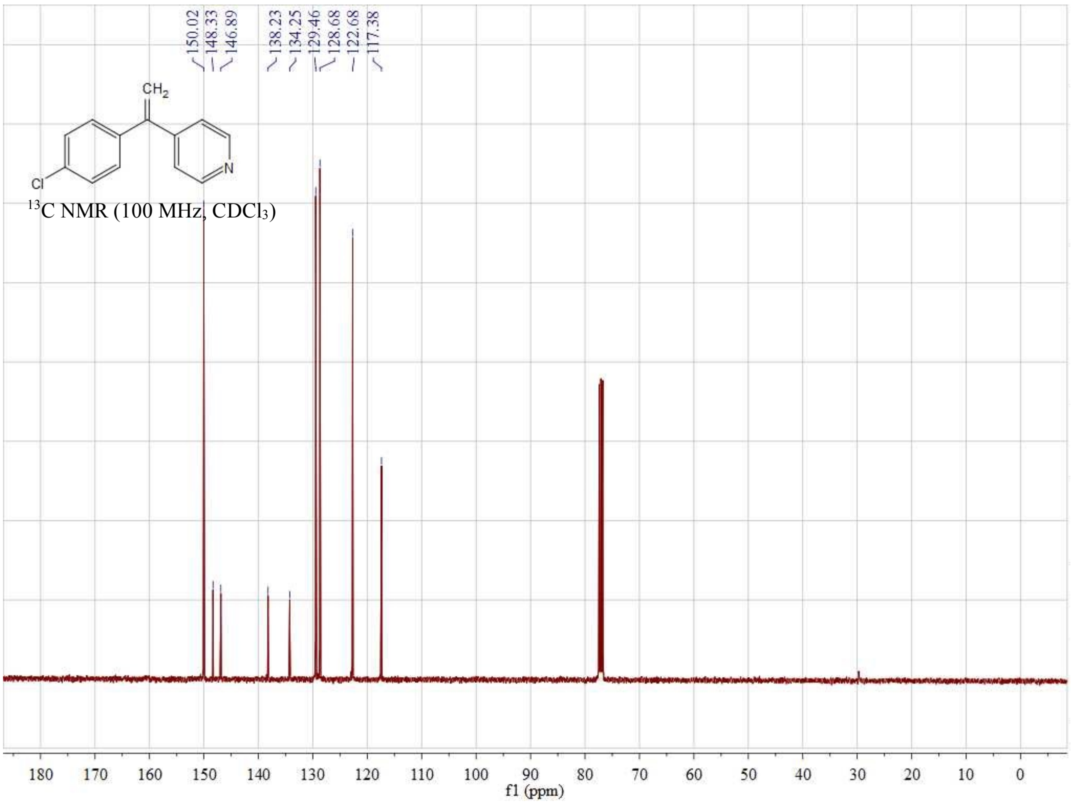

Supplementary Figure 32: ^{13}C NMR Spectra of 4-(1-(4-Chlorophenyl)vinyl) pyridine (26)

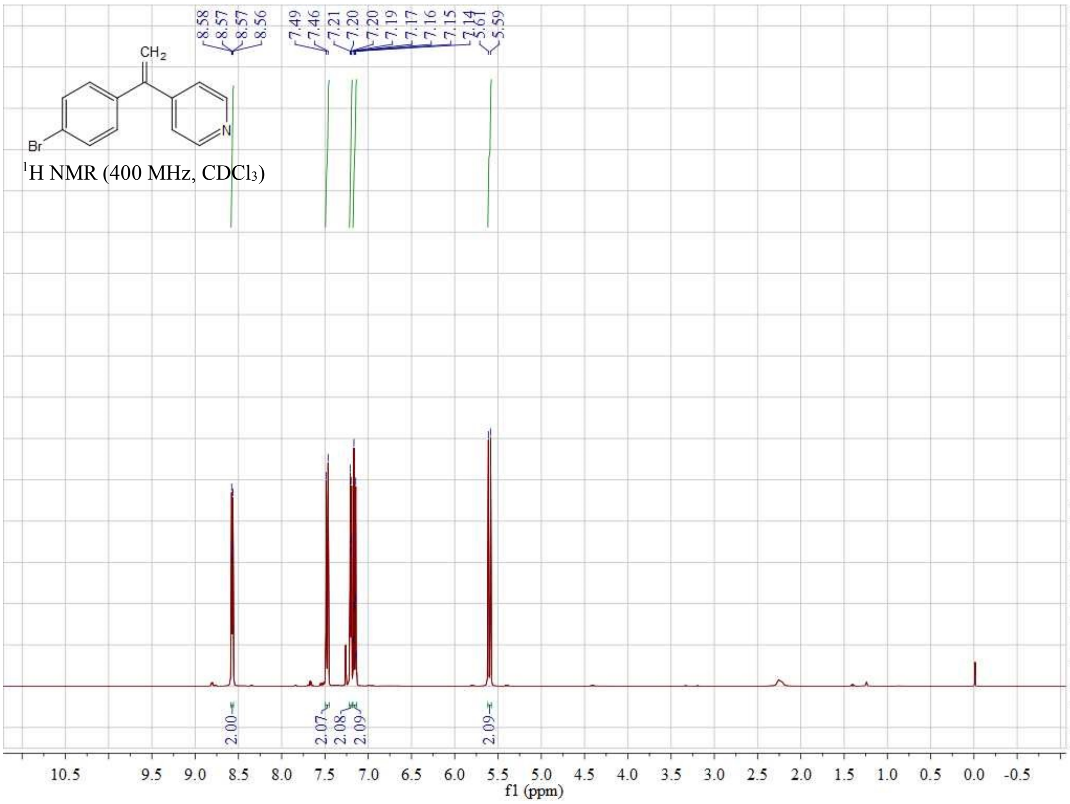

Supplementary Figure 33: ^{1}H NMR Spectra of 4-(1-(4-Bromophenyl)vinyl) pyridine (27)

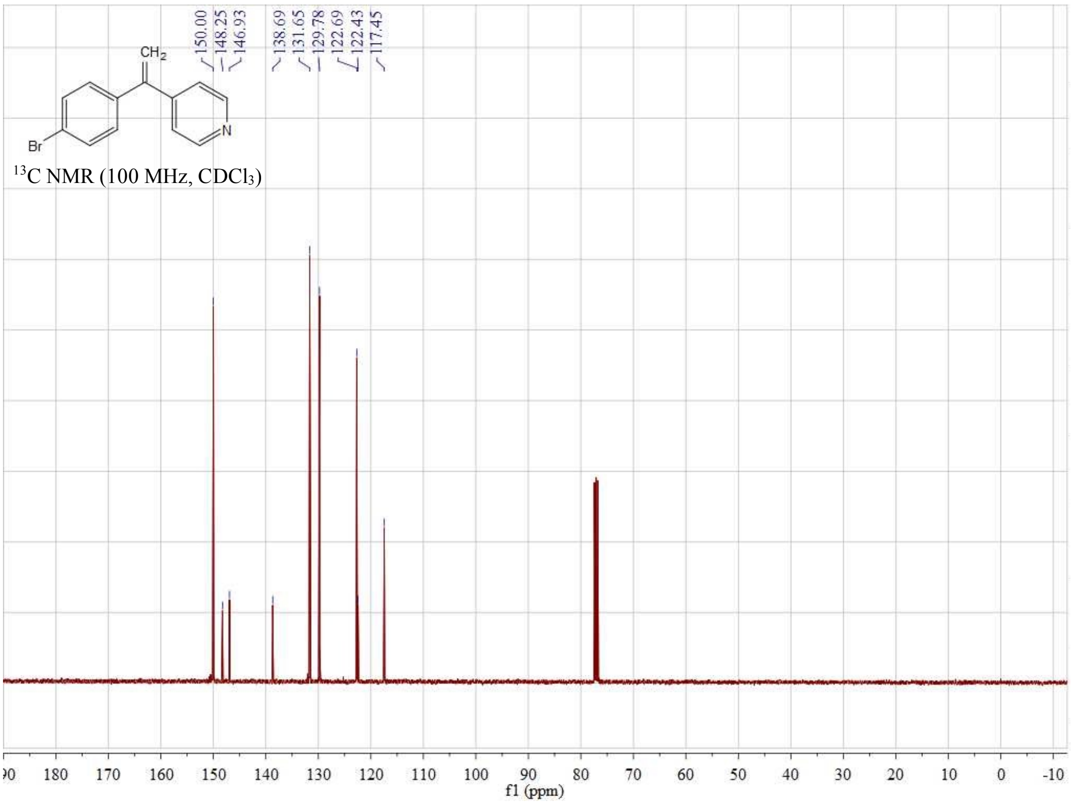

Supplementary Figure 34: ^{13}C NMR Spectra of 4-(1-(4-Bromophenyl)vinyl) pyridine (27)

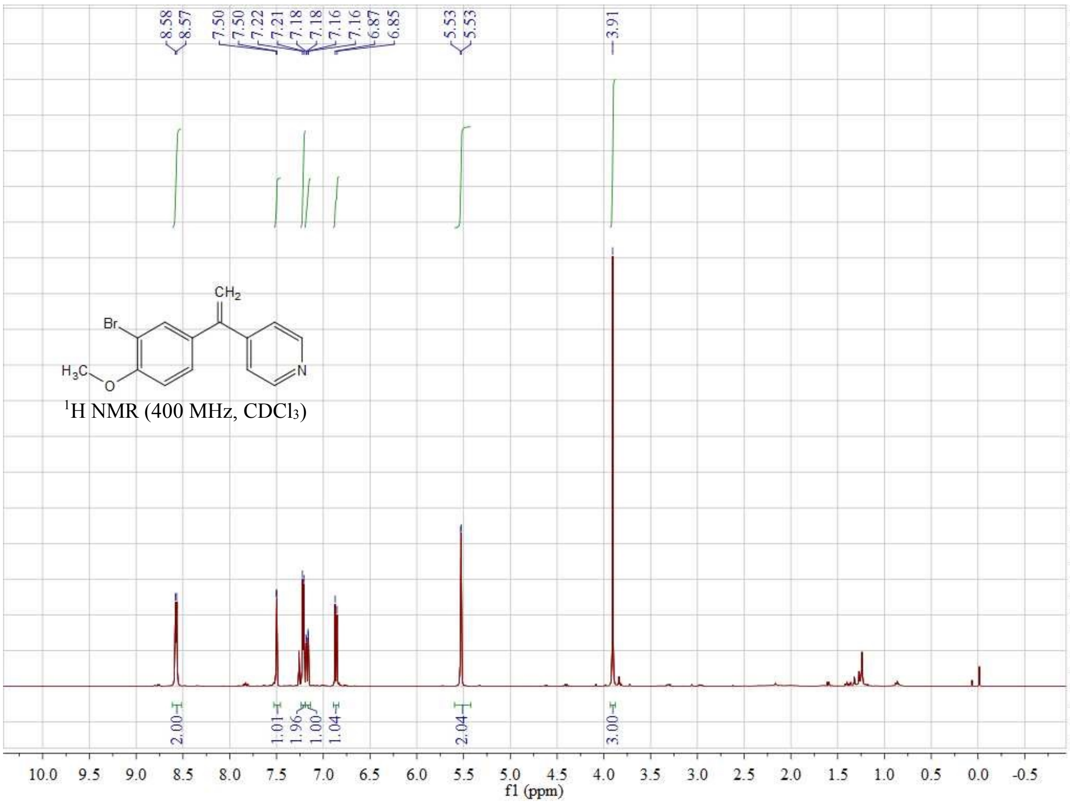

Supplementary Figure 35: ^{1}H NMR Spectra of 4-(1-(3-Bromo-4-methoxyphenyl) vinyl)pyridine (28)

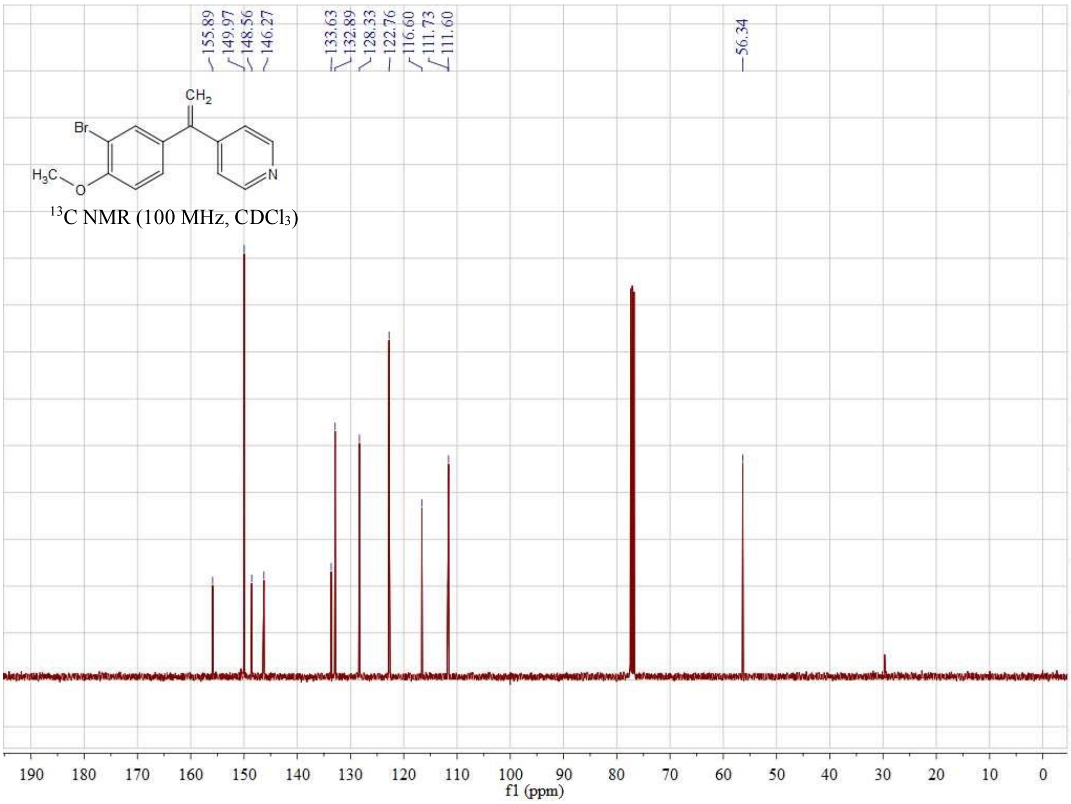

Supplementary Figure 36: ^{13}C NMR Spectra of 4-(1-(3-Bromo-4-methoxyphenyl) vinyl)pyridine (28)

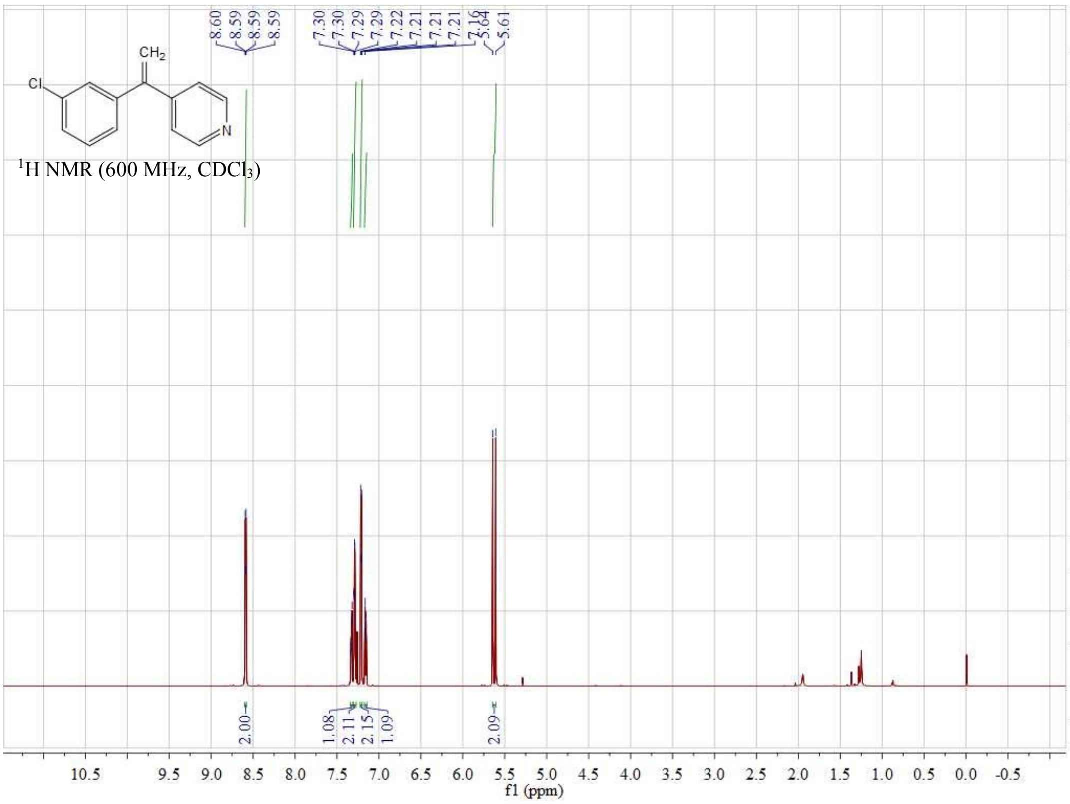

Supplementary Figure 37: ^{1}H NMR Spectra of 4-(1-(3-Chlorophenyl) vinyl) pyridine (29)

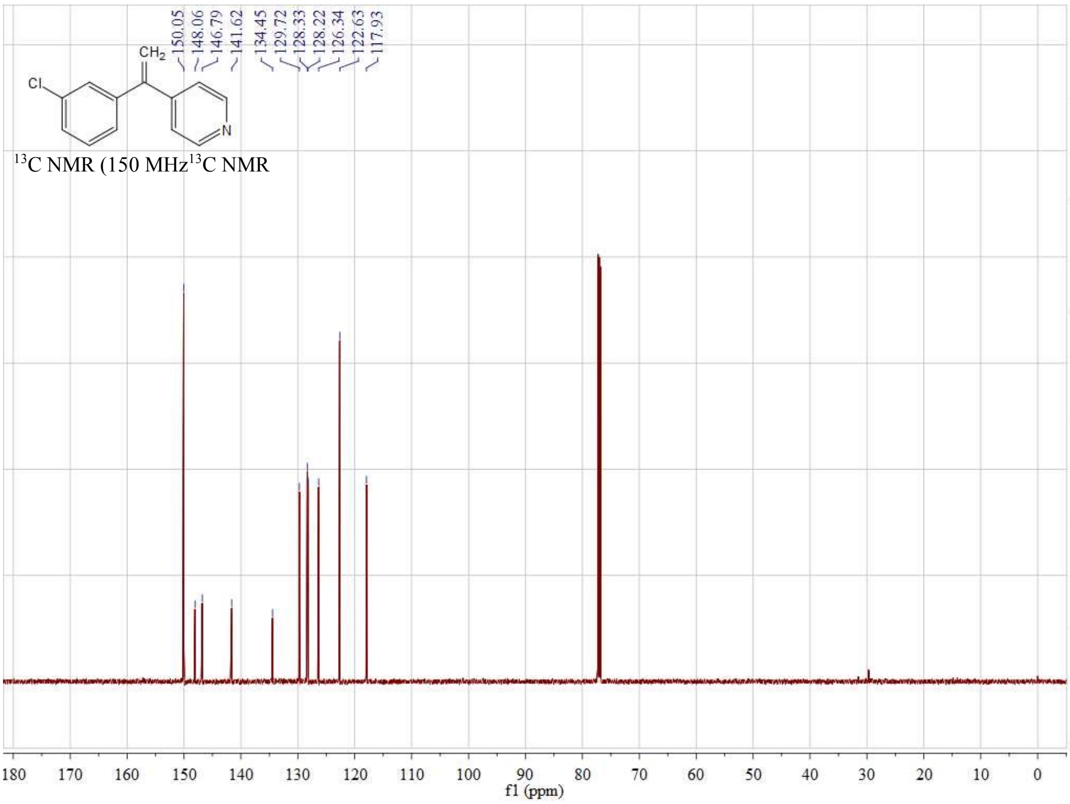

Supplementary Figure 38: ^{13}C NMR Spectra of 4-(1-(3-Chlorophenyl) vinyl) pyridine (29)

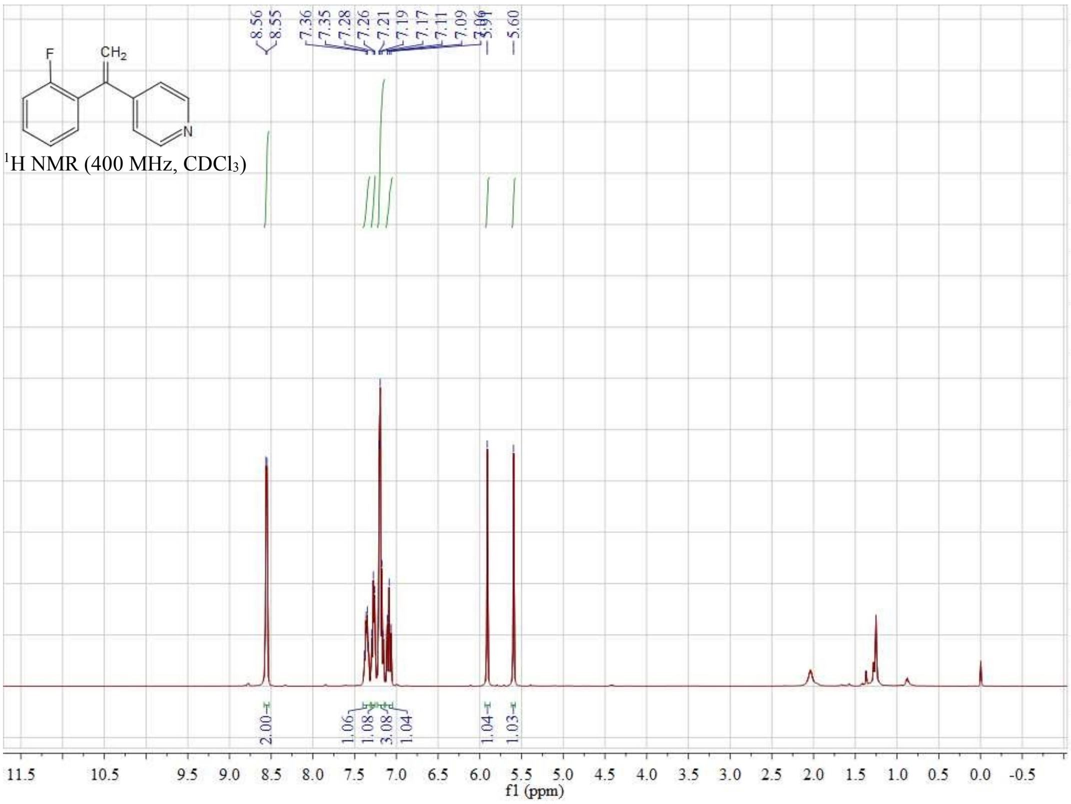

Supplementary Figure 39: ^{1}H NMR Spectra of 4-(1-(2-Fluorophenyl)vinyl) pyridine (30)

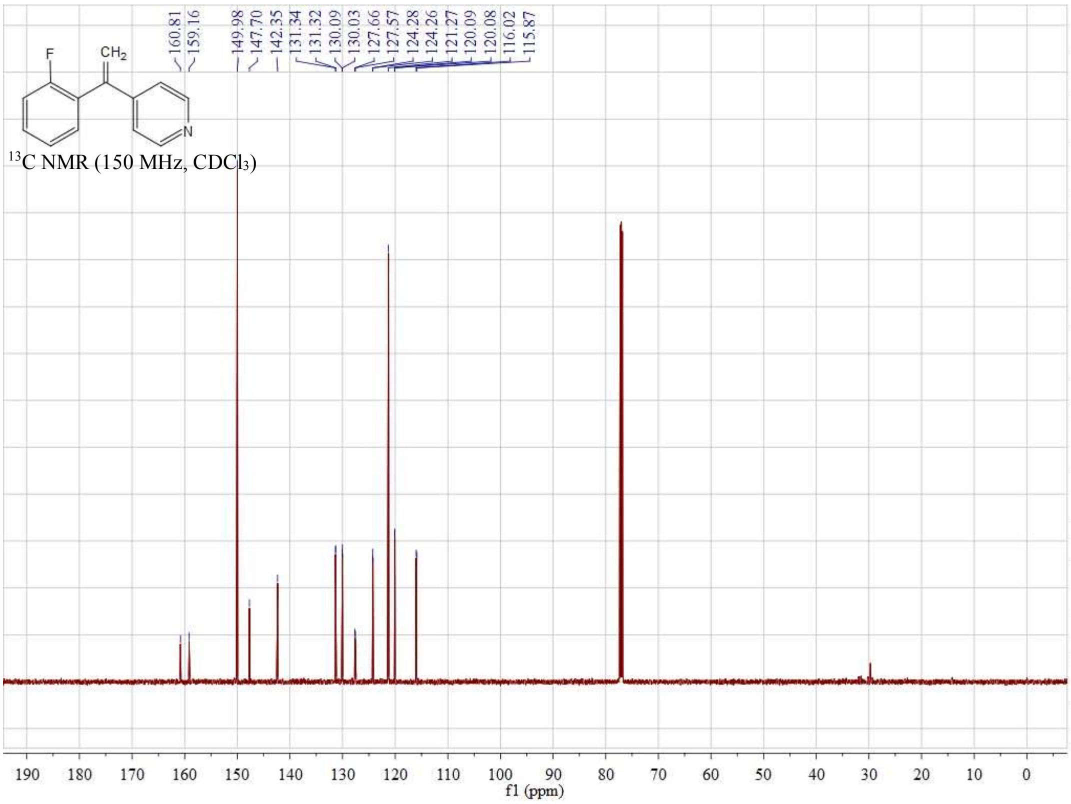

Supplementary Figure 40: ^{13}C NMR Spectra of 4-(1-(2-Fluorophenyl)vinyl) pyridine (30)

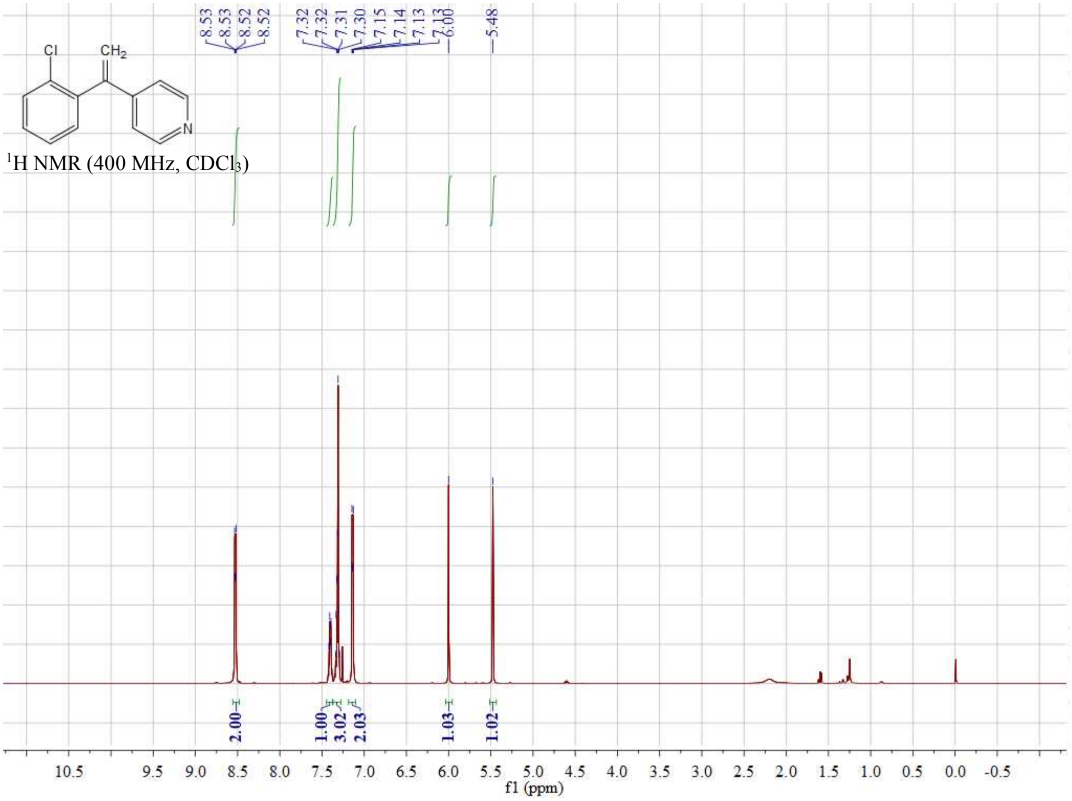

Supplementary Figure 41: ^{1}H NMR Spectra of 4-(1-(2-Chlrophenyl)vinyl)pyridine (31)

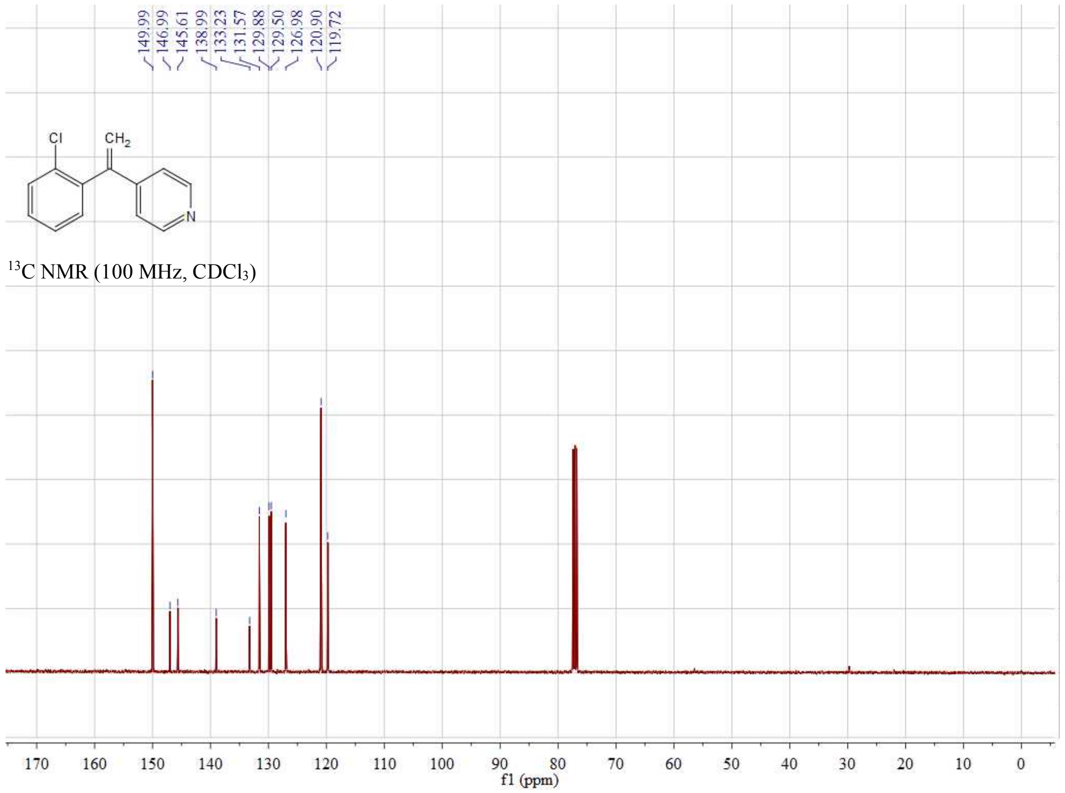

Supplementary Figure 42: ^{13}C NMR Spectra of 4-(1-(2-Chlrophenyl)vinyl) pyridine (31)

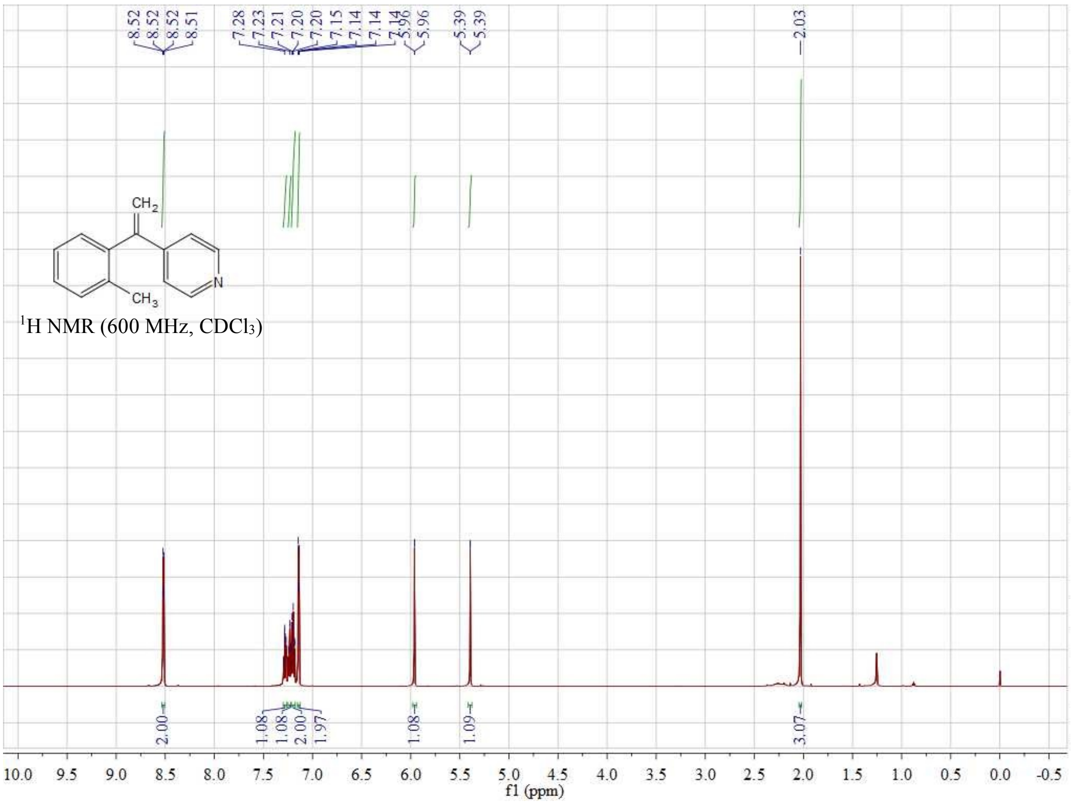

Supplementary Figure 43: ^{1}H NMR Spectra of 4-(1-(o-Tolyl)vinyl)pyridine (32)

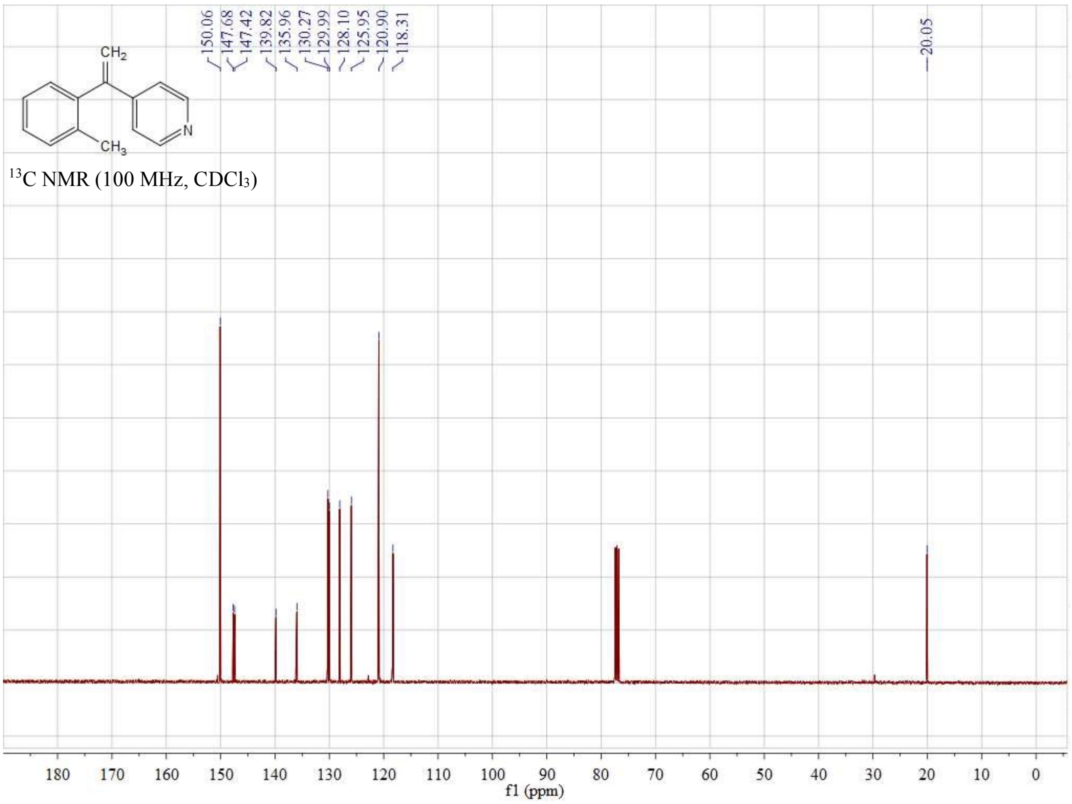

Supplementary Figure 44: ^{13}C NMR Spectra of 4-(1-(o-Tolyl)vinyl)pyridine (32)

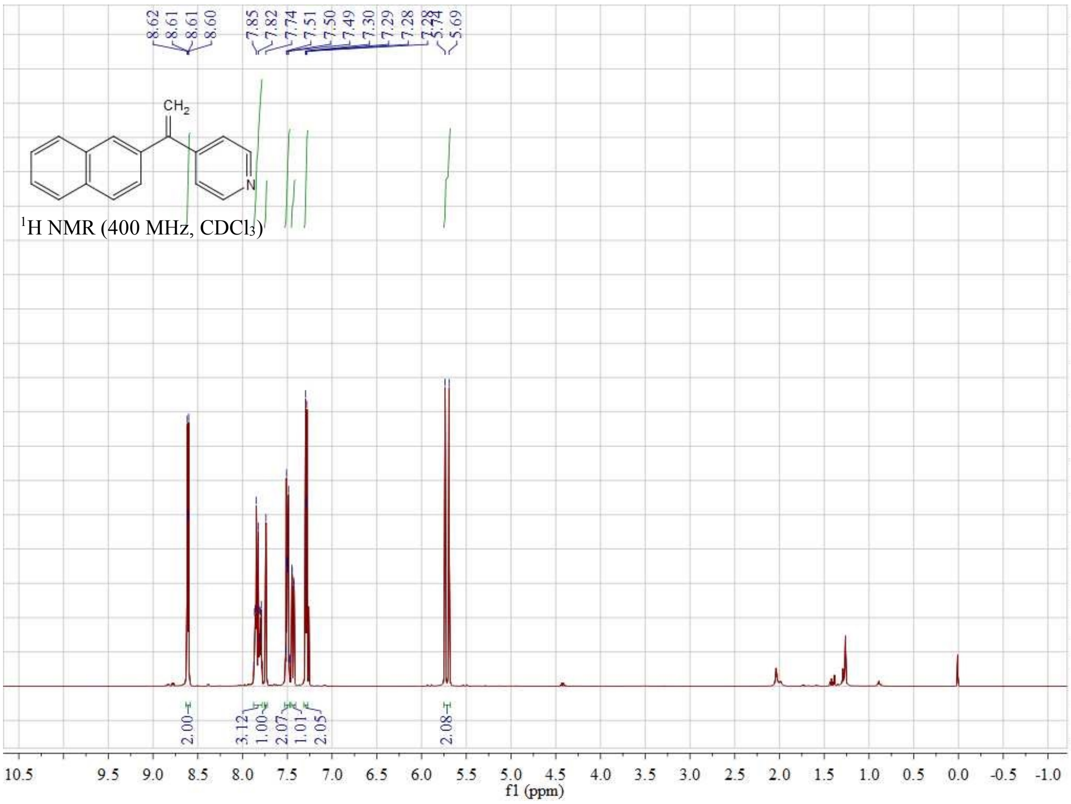

Supplementary Figure 45: ^{1}H NMR Spectra of 4-(1-(Naphthalen-2-yl)vinyl) pyridine (33)

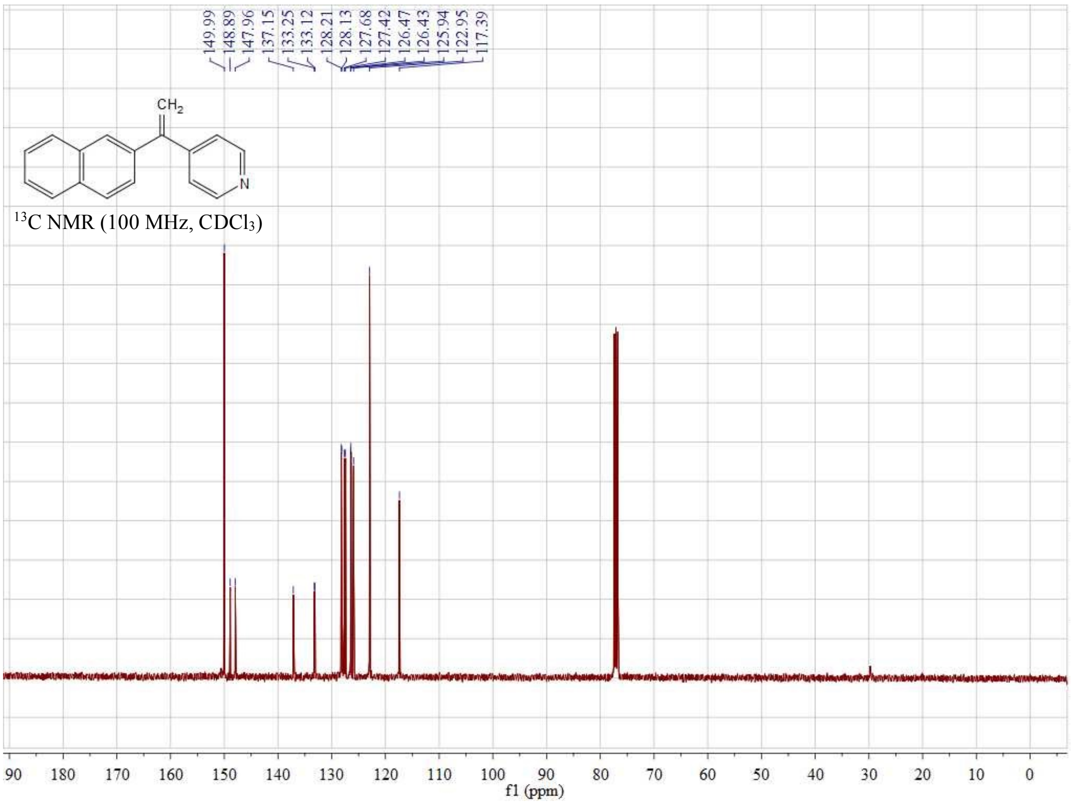

Supplementary Figure 46: ^{13}C NMR Spectra of 4-(1-(Naphthalen-2-yl)vinyl) pyridine (33)

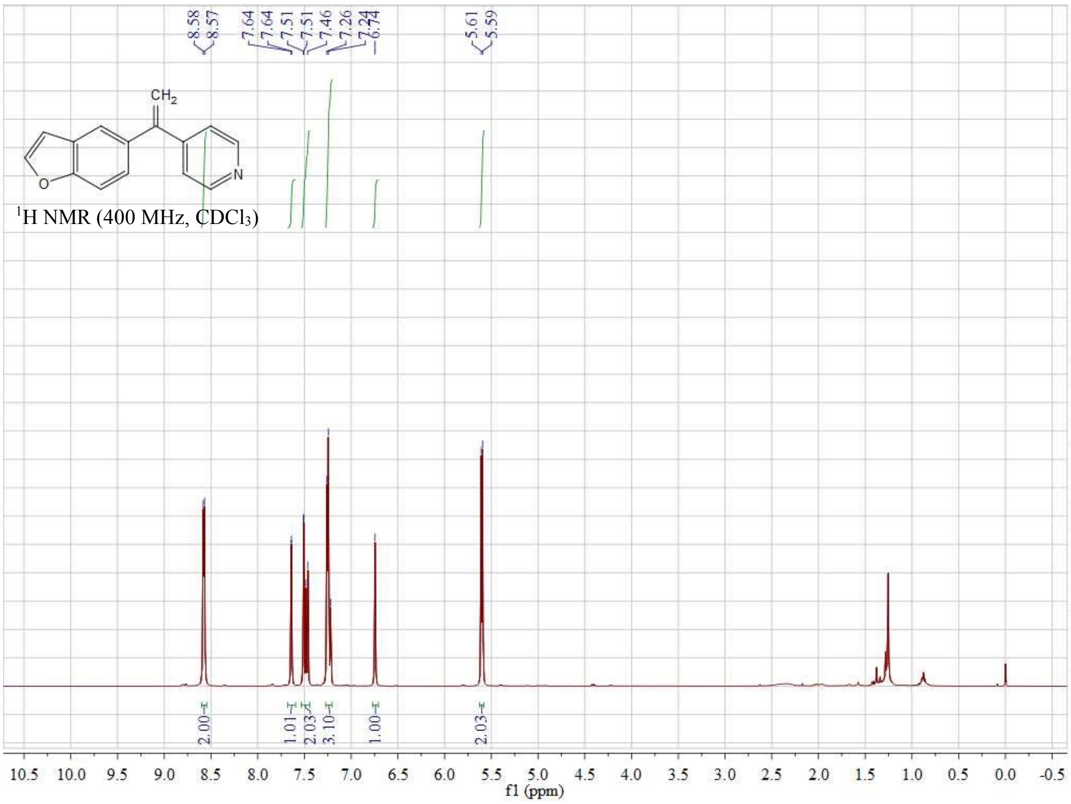

Supplementary Figure 47: ^{1}H NMR Spectra of 4-(1-(Benzofuran-5-yl)vinyl) pyridine (34)

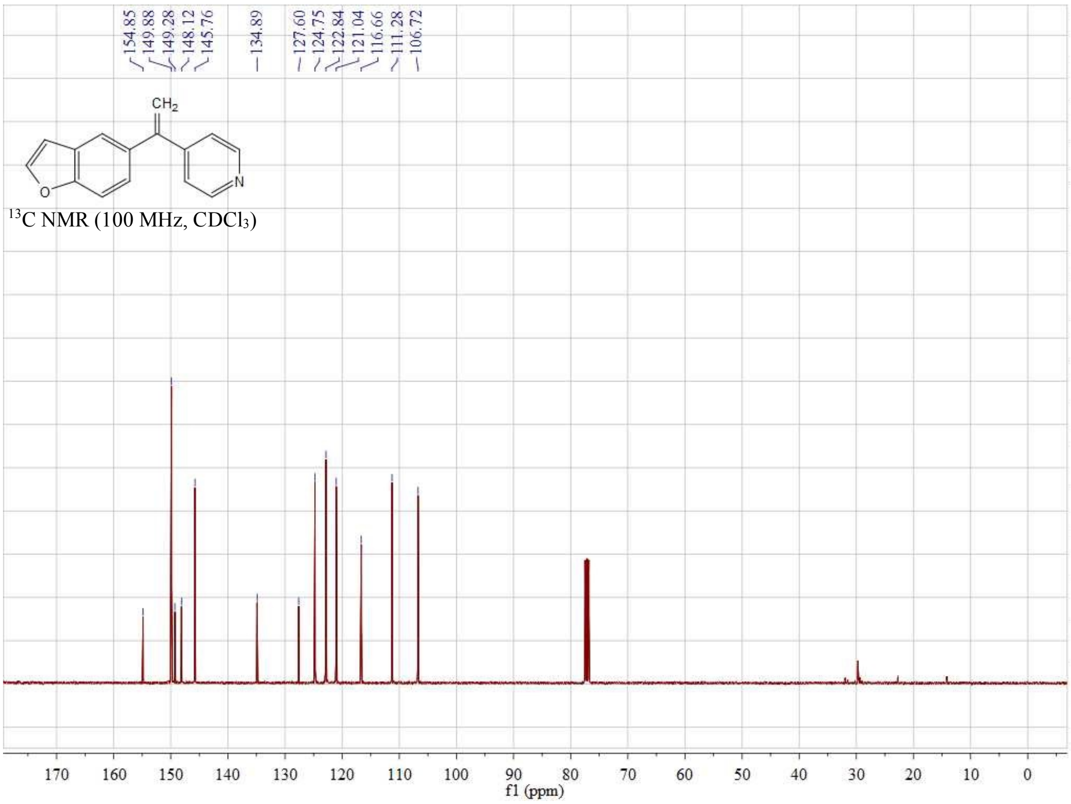

Supplementary Figure 48: ^{13}C NMR Spectra of 4-(1-(Benzofuran-5-yl)vinyl) pyridine (34)

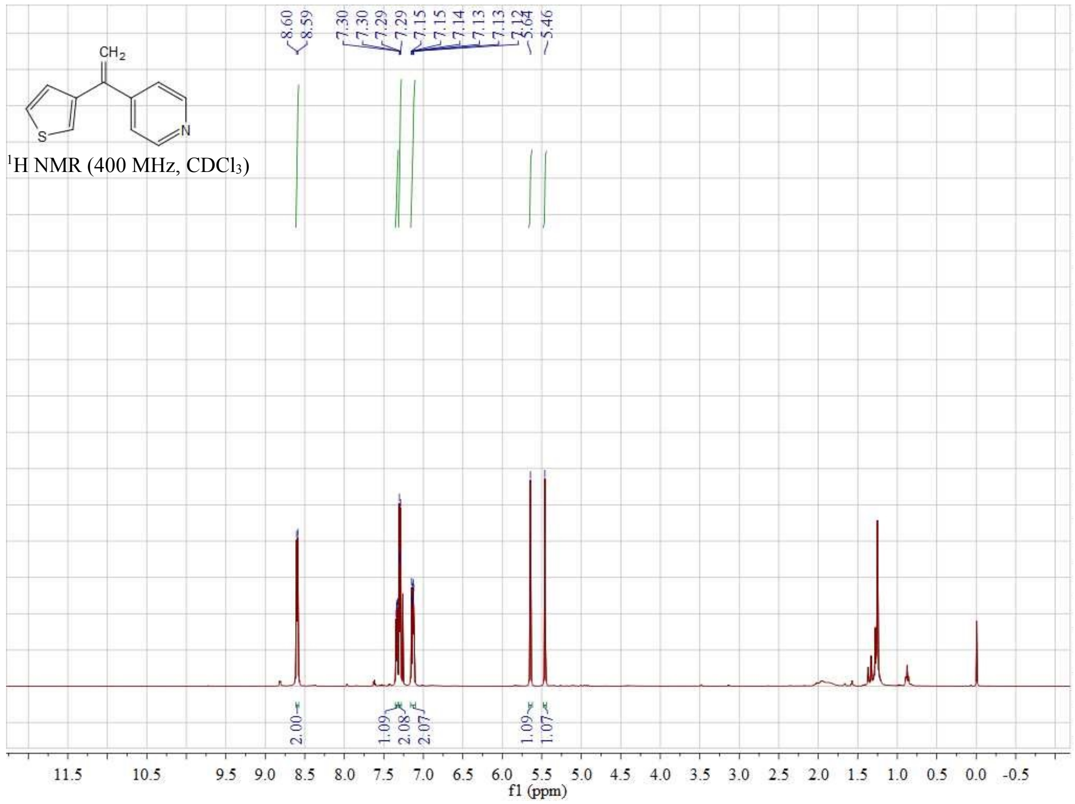

Supplementary Figure 49: ^{1}H NMR Spectra of 4-(1-(Thiophen-3-yl)vinyl)pyridine (35)

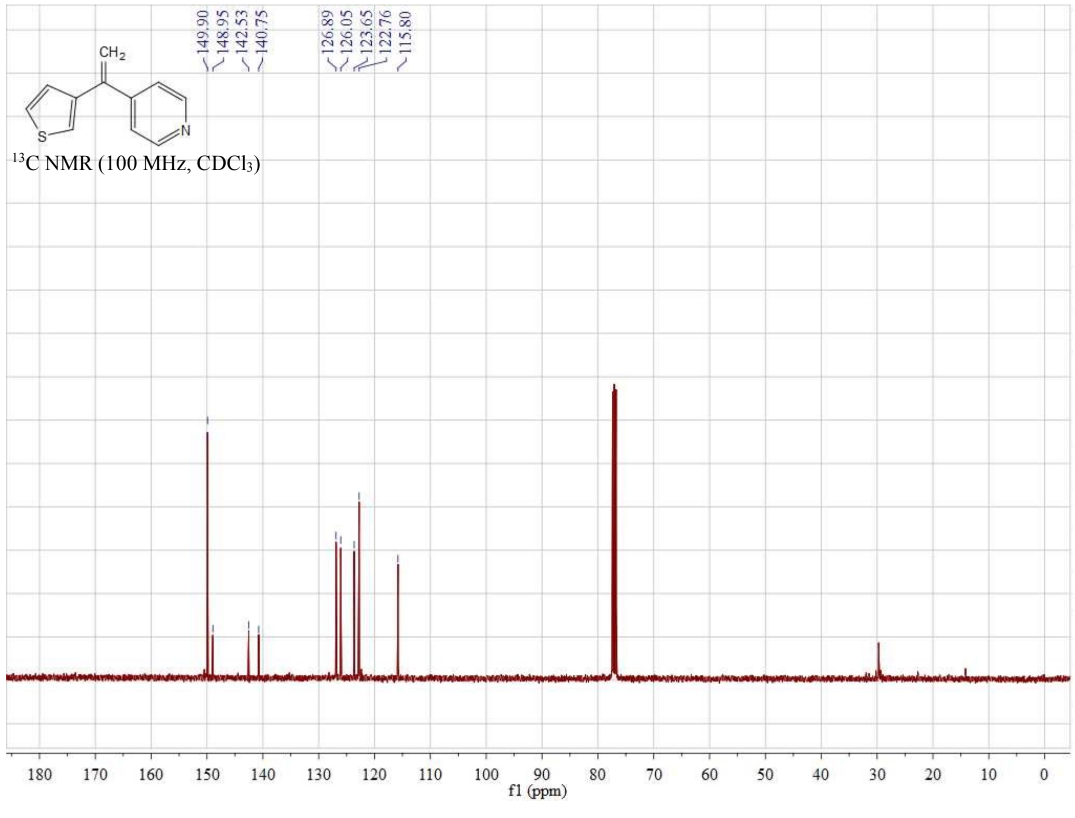

Supplementary Figure 50: ^{13}C NMR Spectra of 4-(1-(Thiophen-3-yl)vinyl) pyridine (35)

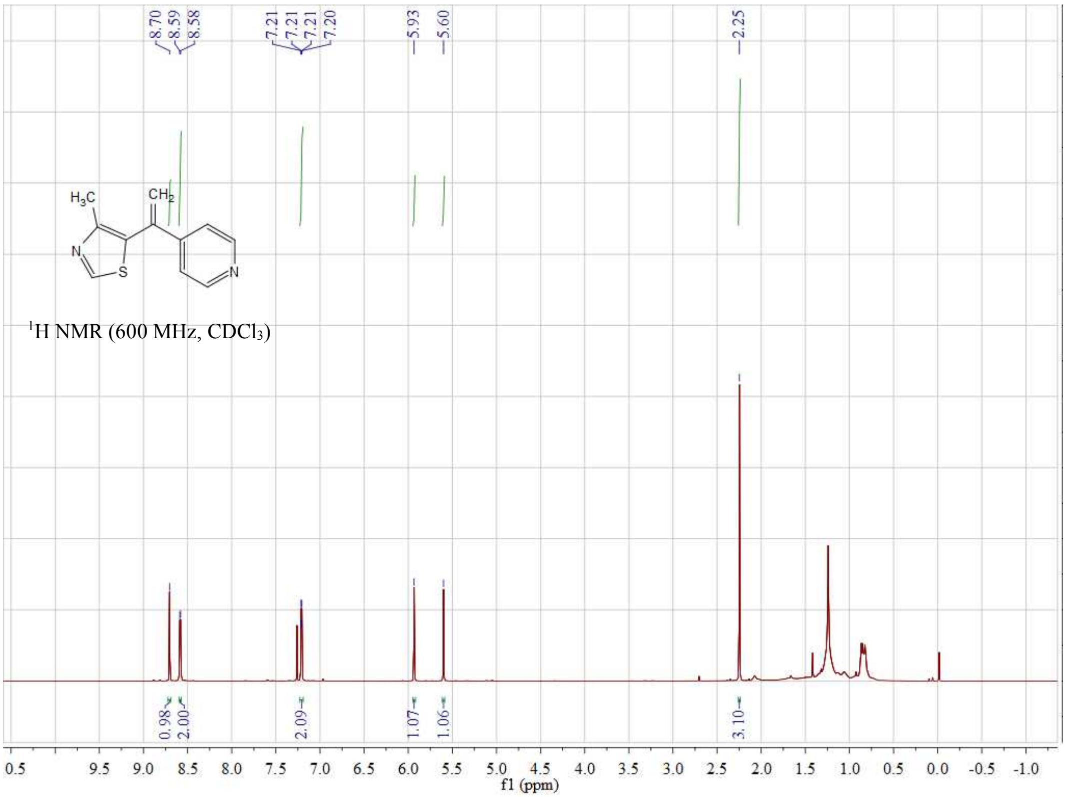

Supplementary Figure 51: ^{1}H NMR Spectra of 4-methyl-5-(1-(pyridin-4-yl)vinyl) thiazole (36)

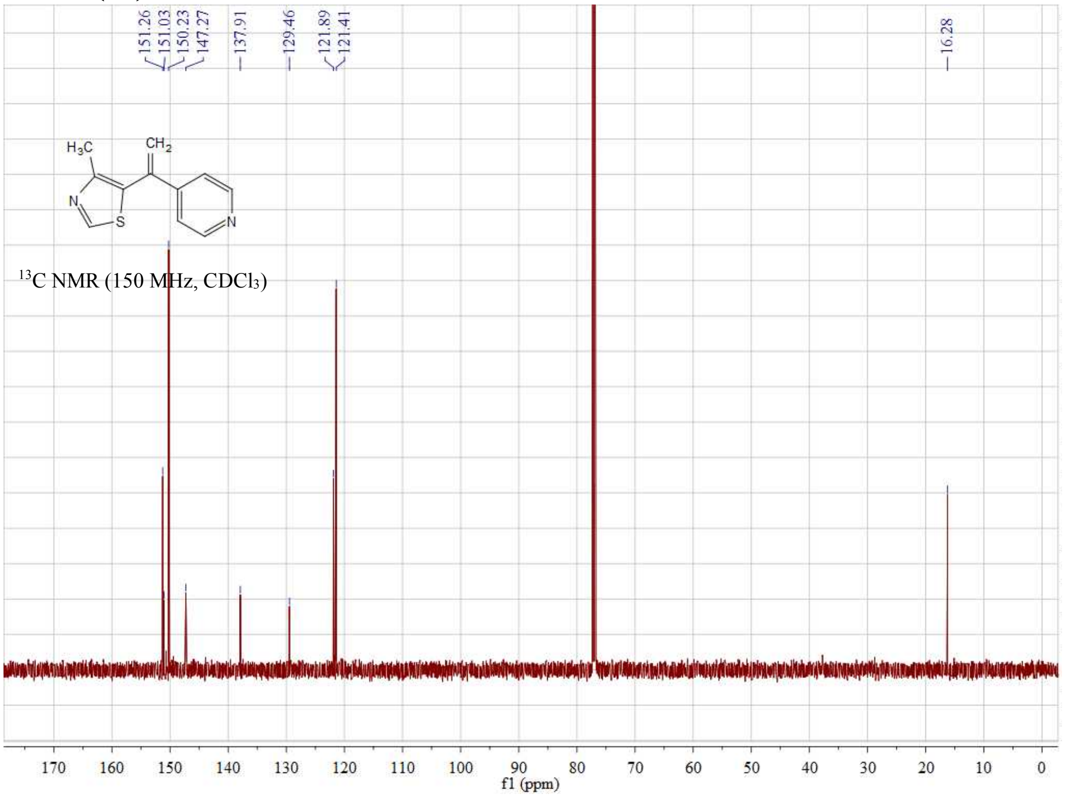

Supplementary Figure 52: ^{13}C NMR Spectra of 4-methyl-5-(1-(pyridin-4-yl)vinyl) thiazole (36)

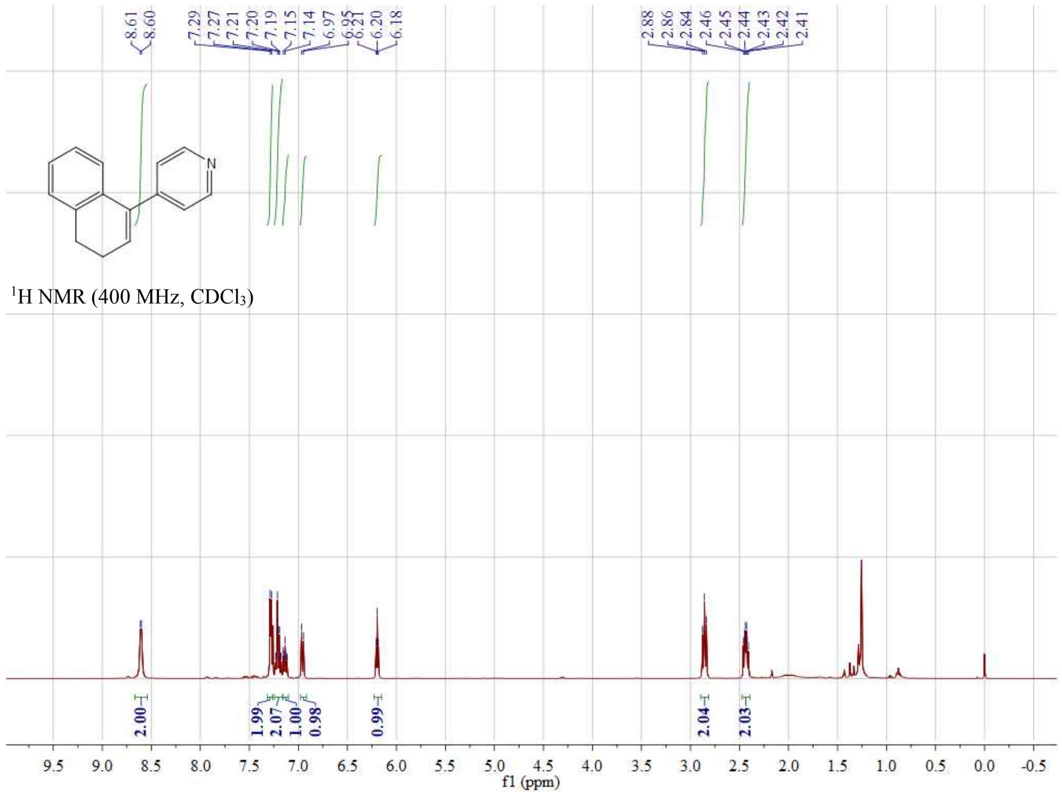

Supplementary Figure 53: ^{1}H NMR Spectra of 4-(3,4-Dihydronaphthalen-1-yl)pyridine (37)

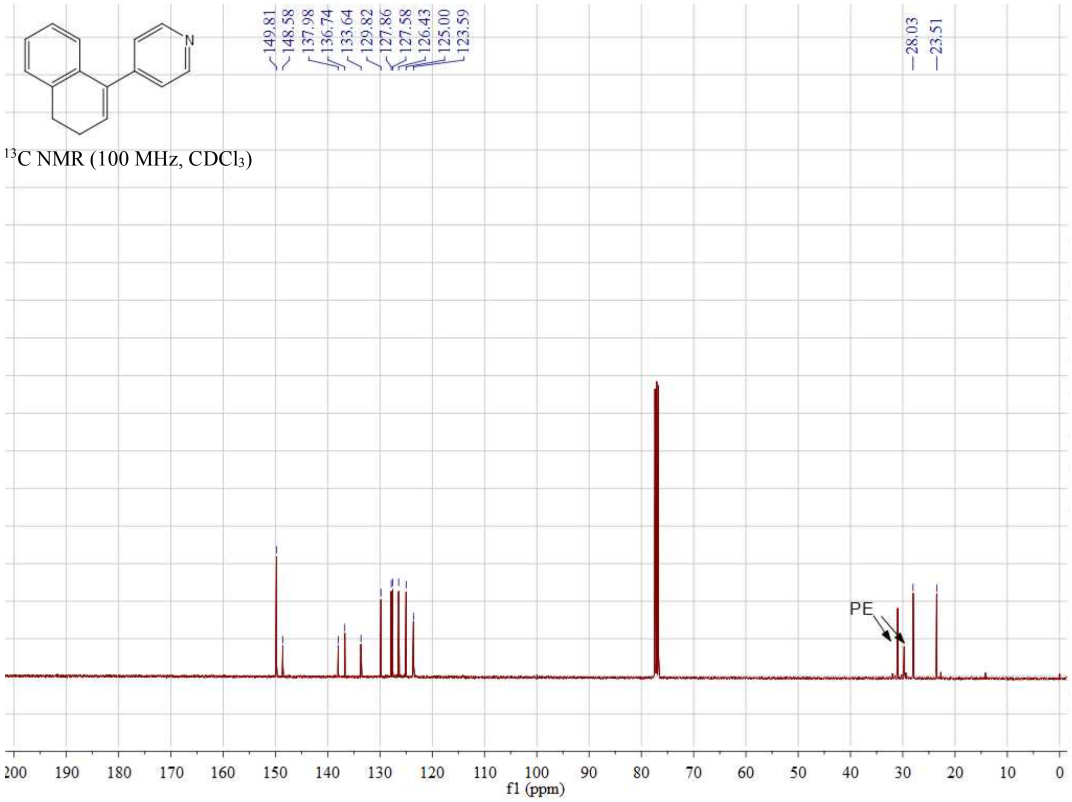

Supplementary Figure 54: ^{13}C NMR Spectra of 4-(3,4-Dihydronaphthalen-1-yl)pyridine (37)

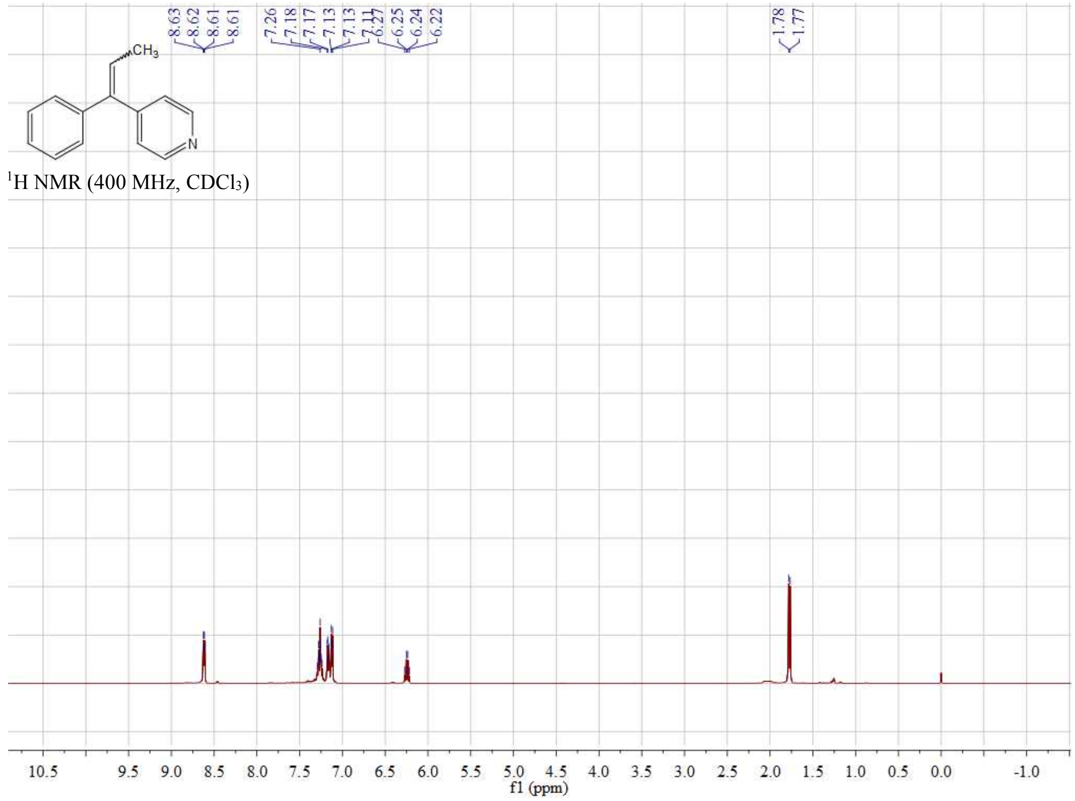

Supplementary Figure 55: ^{1}H NMR Spectra of 4-(1-Phenylprop-1-en-1-yl)pyridine (38)

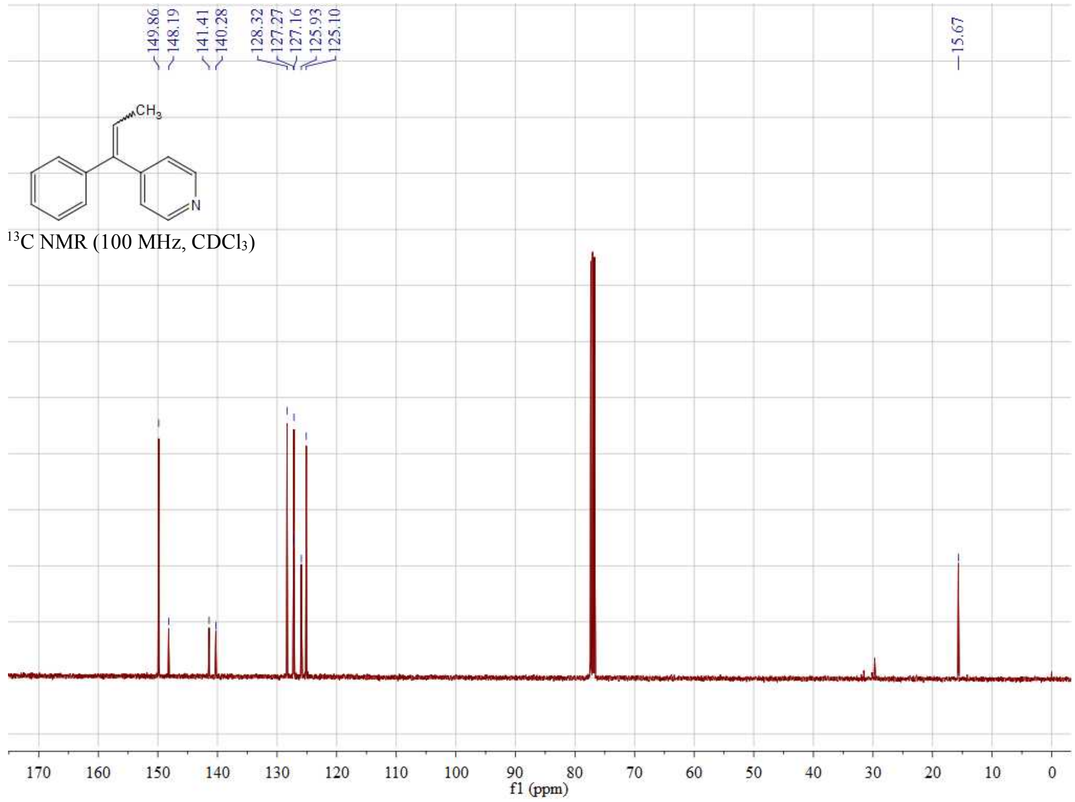

Supplementary Figure 56: ^{13}C NMR Spectra of 4-(1-Phenylprop-1-en-1-yl)pyridine (38)

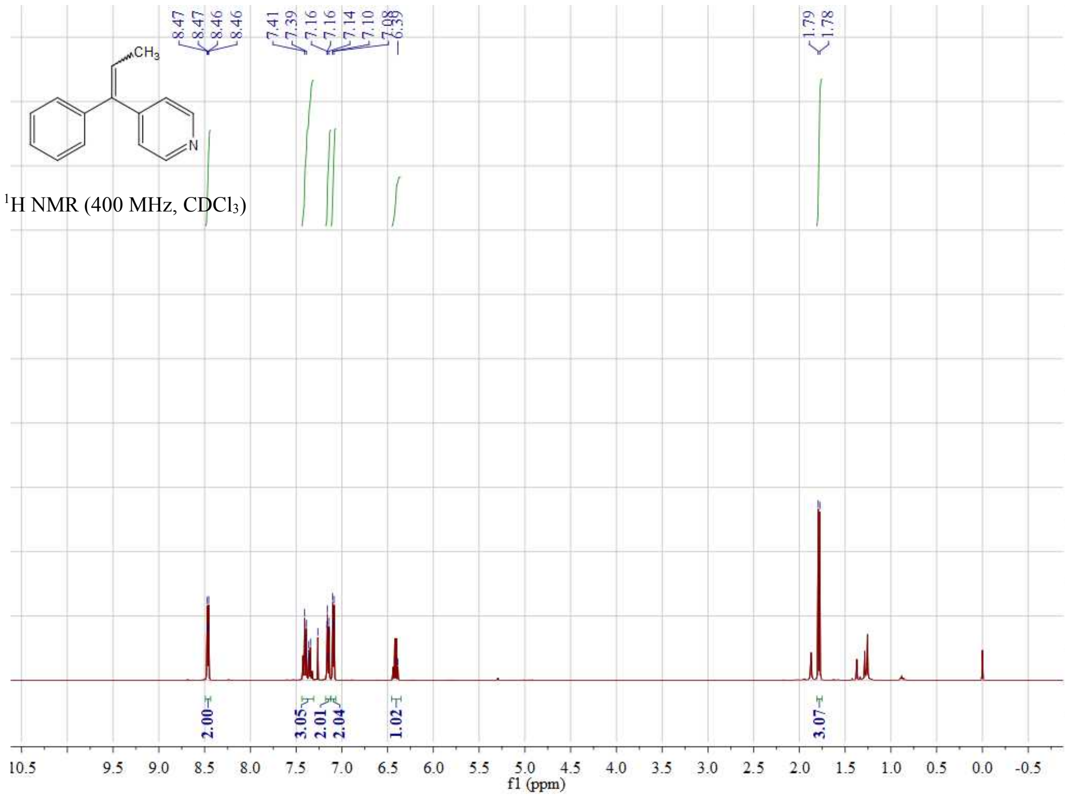

Supplementary Figure 57: ^{1}H NMR Spectra of 4-(1-Phenylprop-1-en-1-yl) pyridine (38)

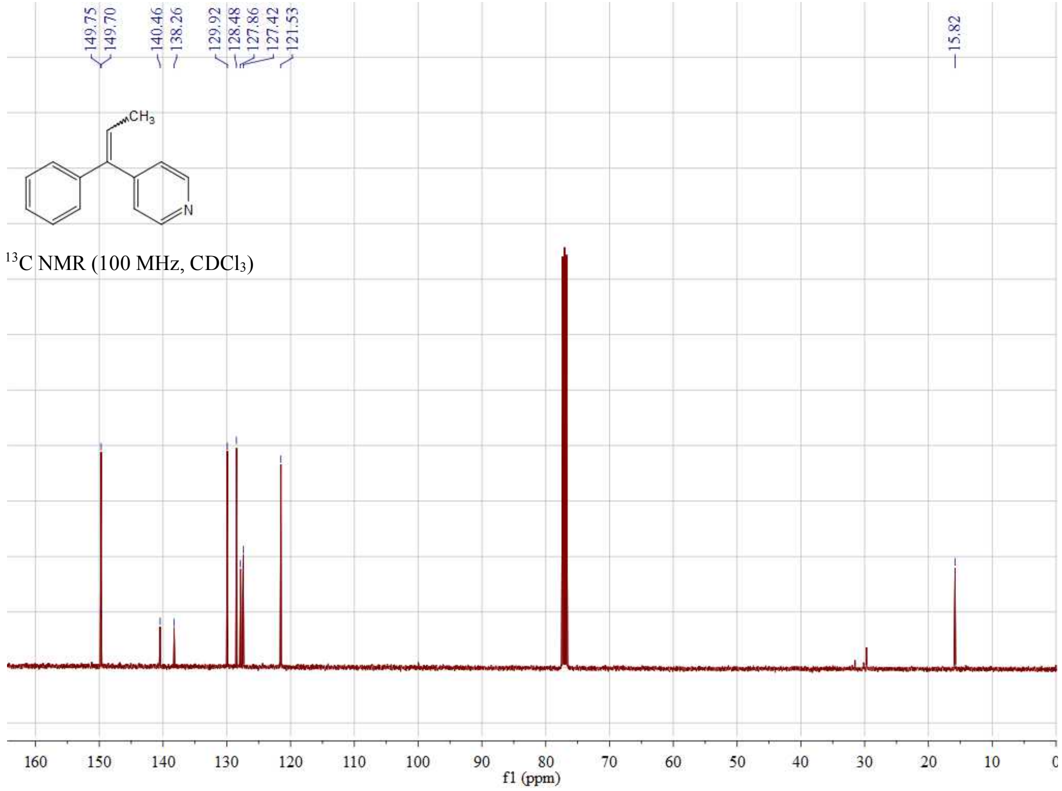

Supplementary Figure 58: ^{13}C NMR Spectra of 4-(1-Phenylprop-1-en-1-yl) pyridine (38)

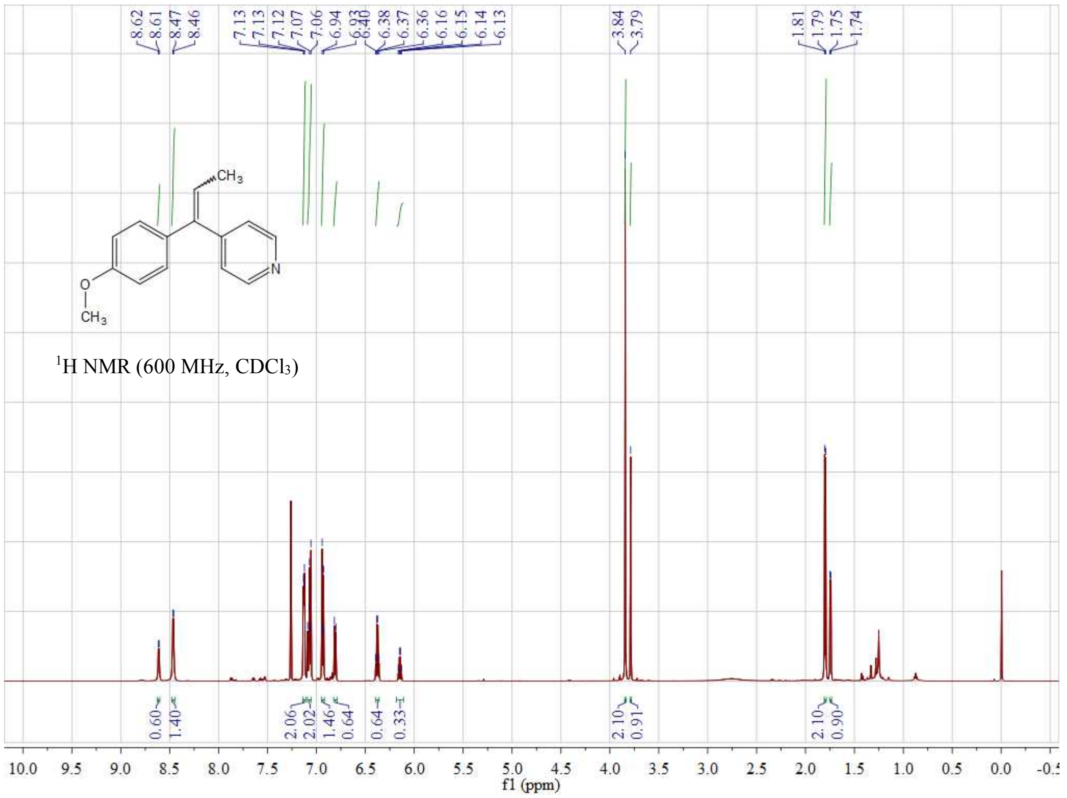

Supplementary Figure 59: ^{1}H NMR Spectra of 4-(1-(4-methoxyphenyl)prop-1-en-1-yl)pyridine (39)

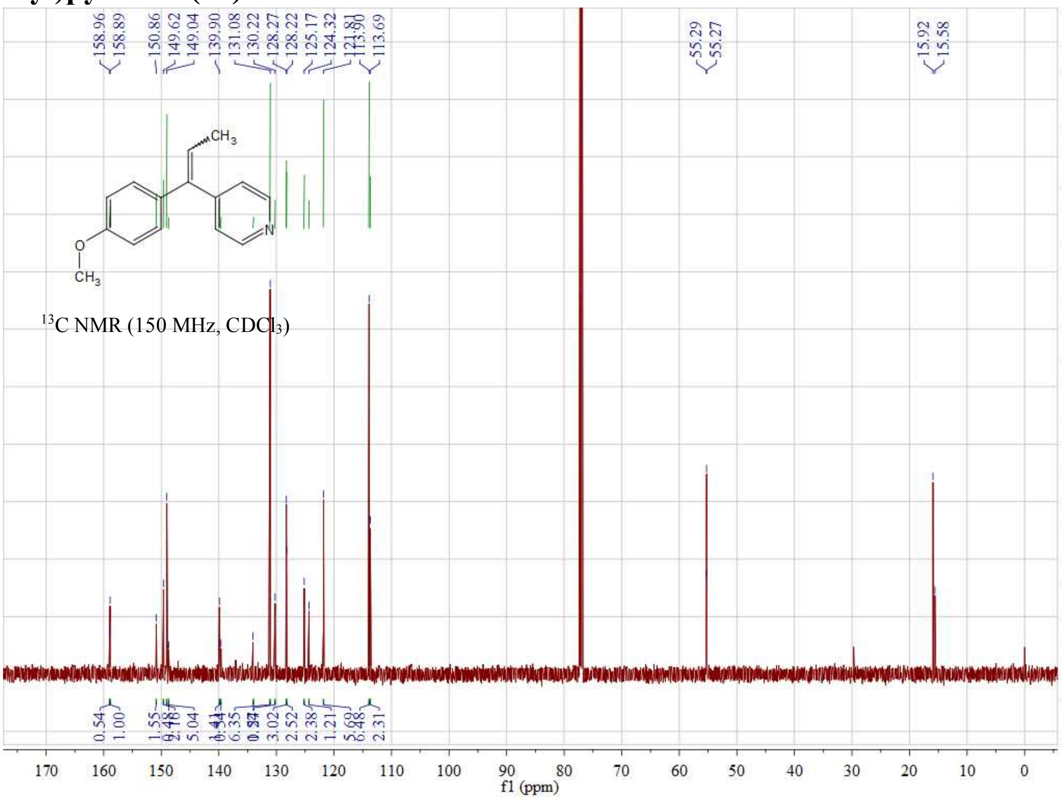

Supplementary Figure 60: ^{13}C NMR Spectra of 4-(1-(4-methoxyphenyl)prop-1-en-1-yl)pyridine (39)

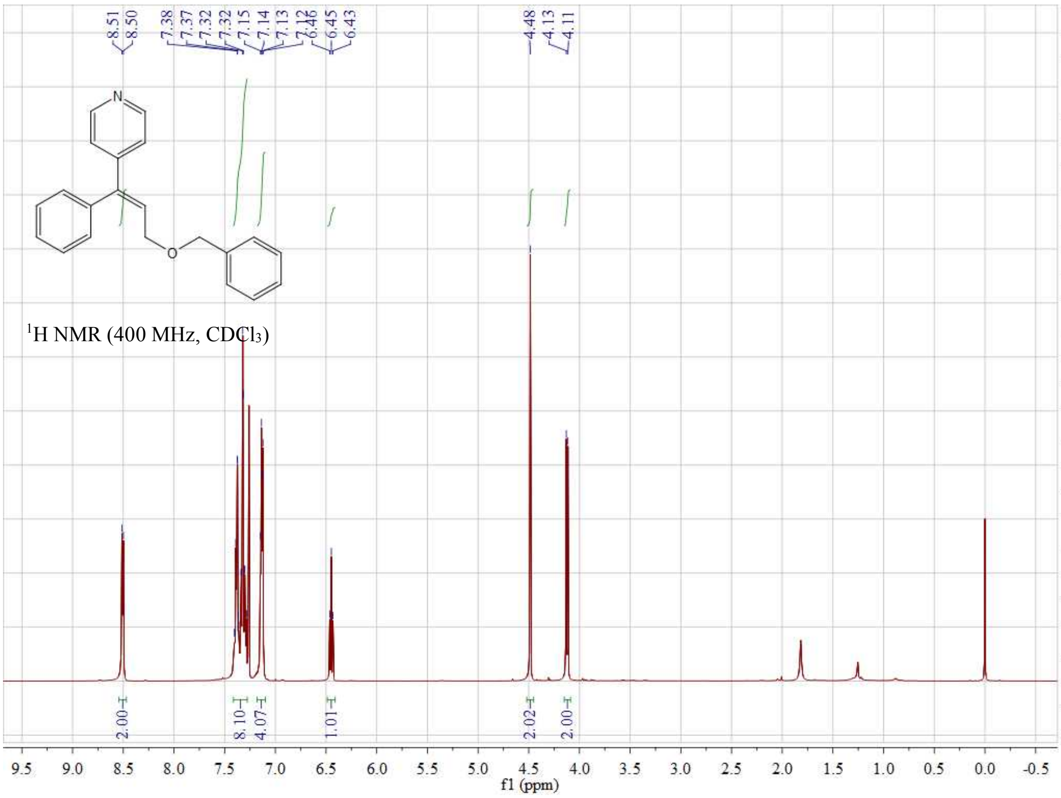

Supplementary Figure 61: ^{1}H NMR Spectra of (E)-4-(3-(benzyloxy)-1-phenylprop -1-en-1-yl)pyridine (40)

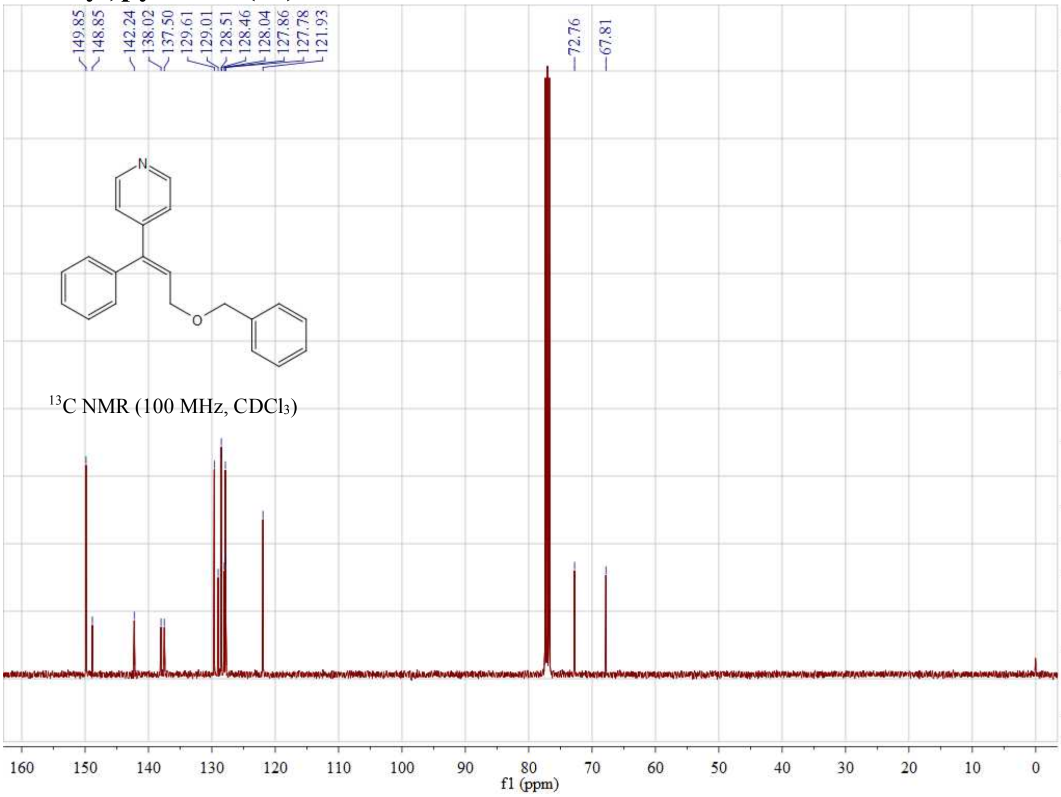

Supplementary Figure 62: ^{13}C NMR Spectra of (E)-4-(3-(benzyloxy)-1-phenylprop-1-en-1-yl)pyridine (40)

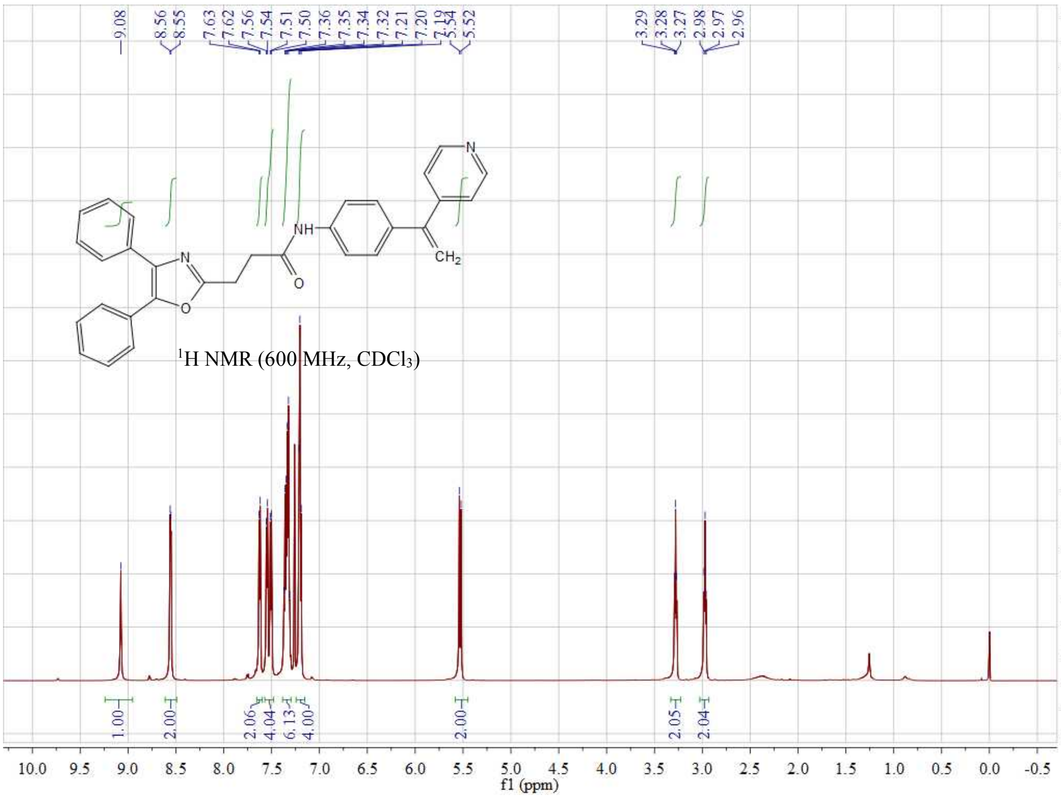

Supplementary Figure 63: ^{1}H NMR Spectra of 3-(4,5-diphenyloxazol-2-yl)-N-(4-(1-(pyridin-4-yl)vinyl)phenyl)propenamide (41)

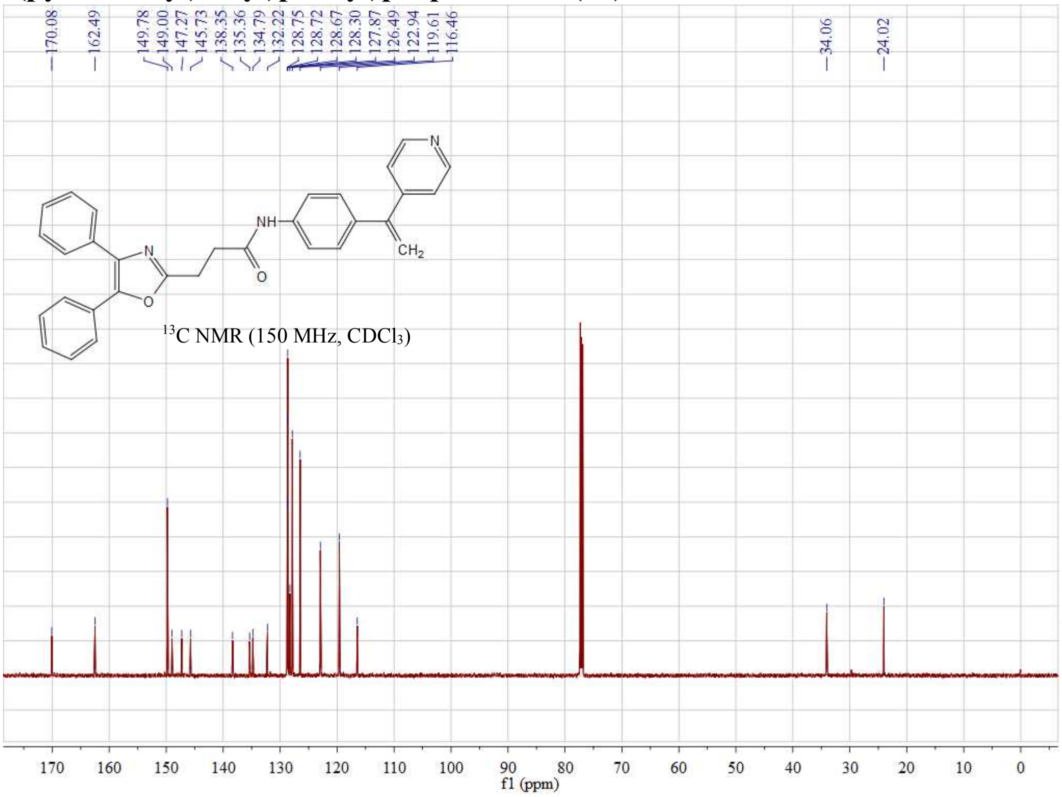

Supplementary Figure 64: ^{13}C NMR Spectra of 3-(4,5-diphenyloxazol-2-yl)-N-(4-(1-(pyridin-4-yl)vinyl)phenyl)propenamide (41)

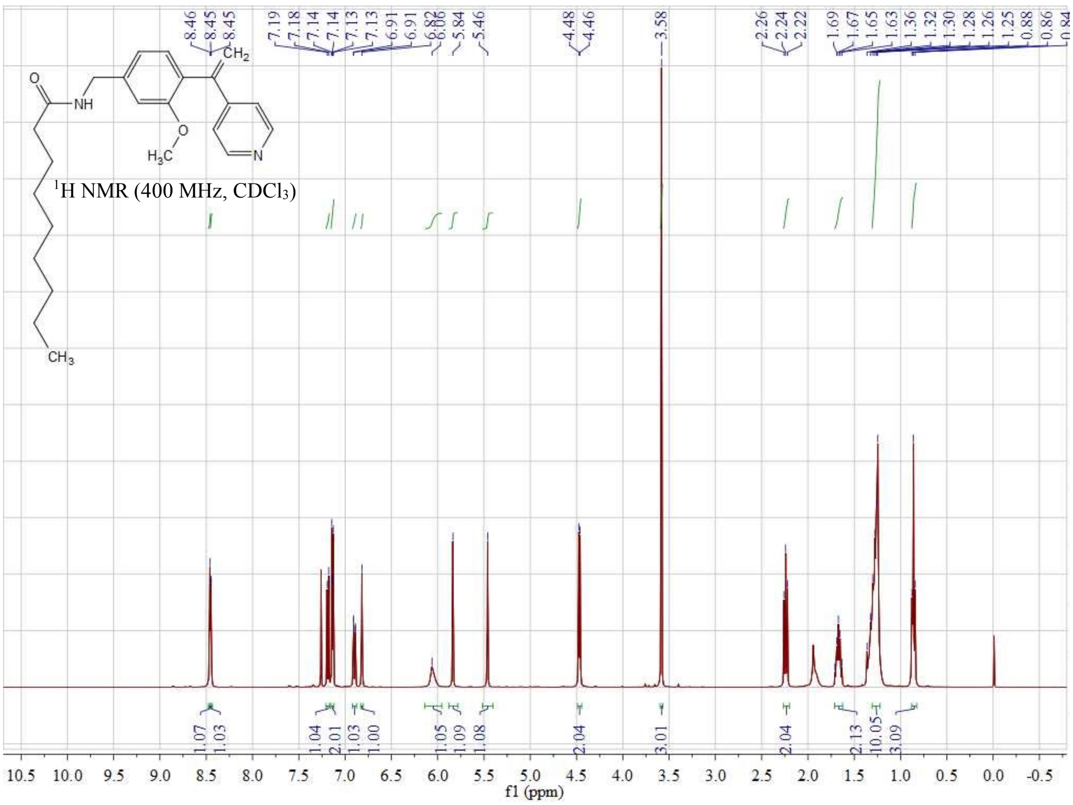

Supplementary Figure 65: ^{1}H NMR Spectra of N-(3-Methoxy-4-(1-(pyridin-4-yl)vinyl)benzyl)nonanamide (42)

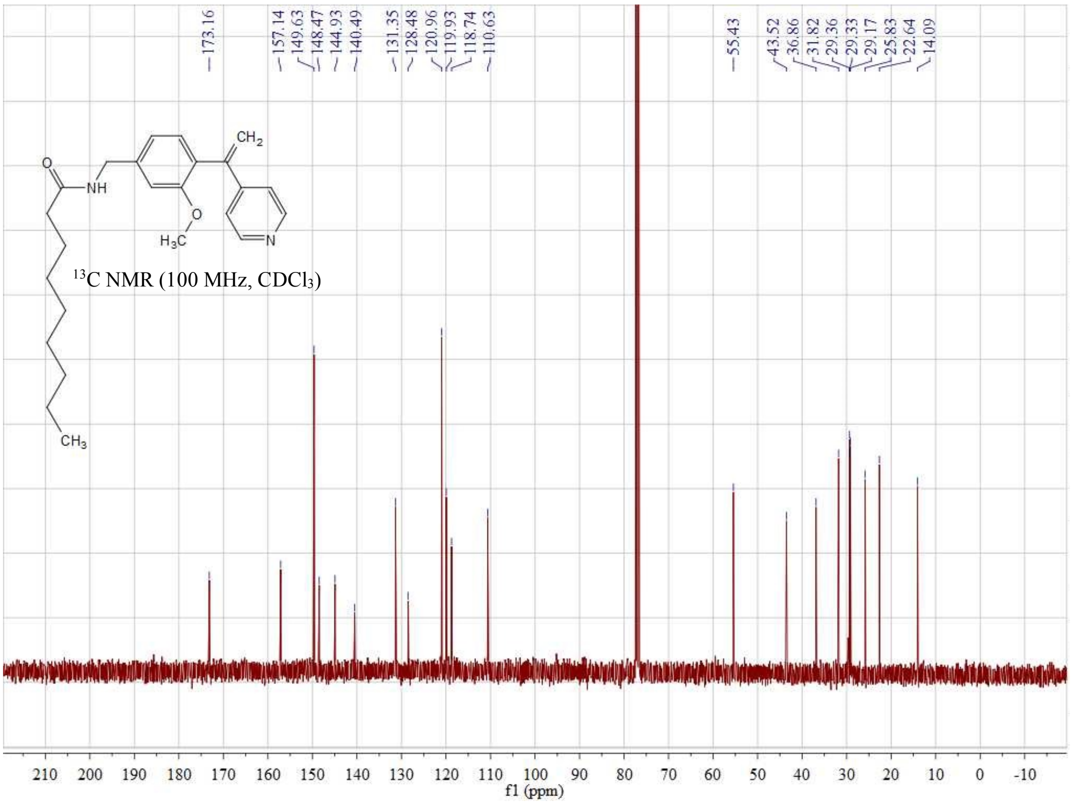

Supplementary Figure 66: ^{13}C NMR Spectra of N-(3-Methoxy-4-(1-(pyridin-4-yl)vinyl)benzyl)nonanamide (42)

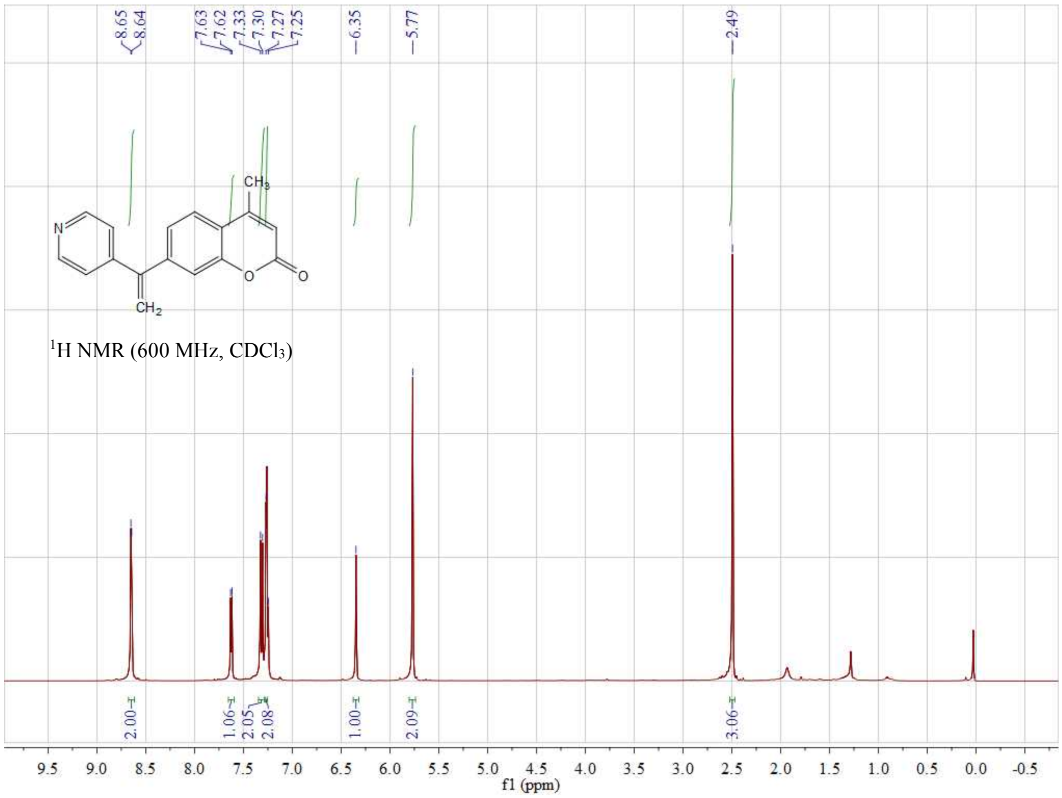

Supplementary Figure 67: ^{1}H NMR Spectra of 4-methyl-7-(1-(pyridin-4-yl)vinyl)-2H-chromen-2-one (43)

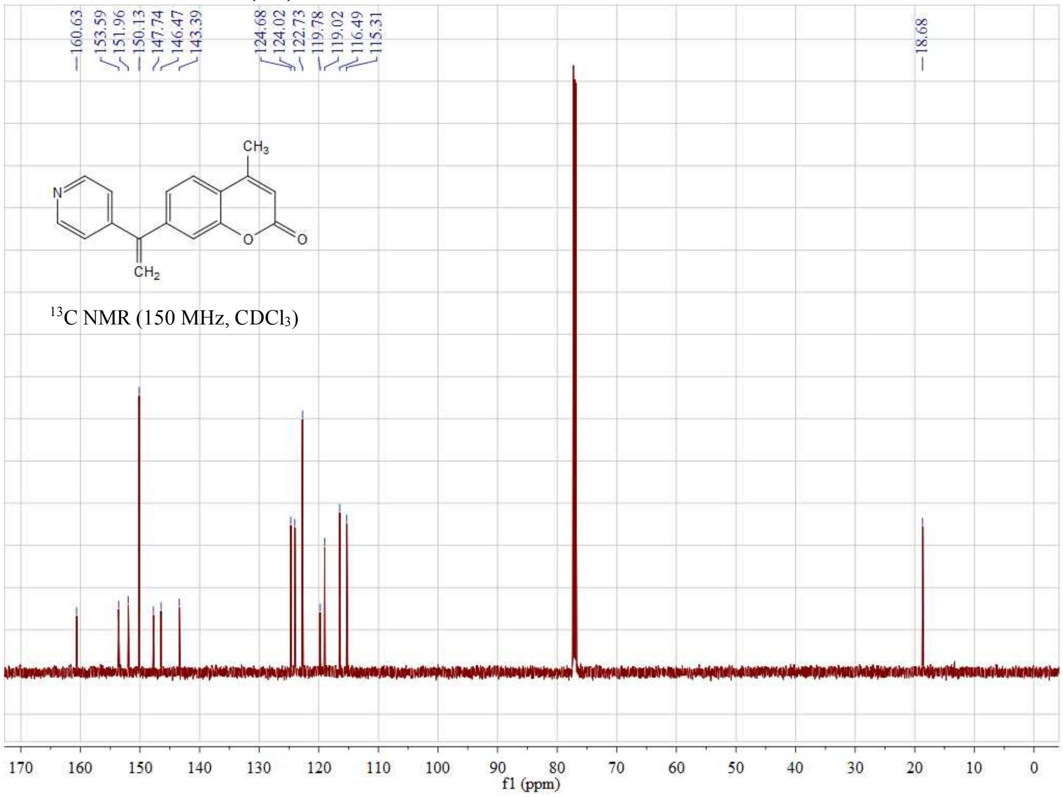

Supplementary Figure 68: ^{13}C NMR Spectra of 4-methyl-7-(1-(pyridin-4-yl)vinyl)-2H-chromen-2-one (43)

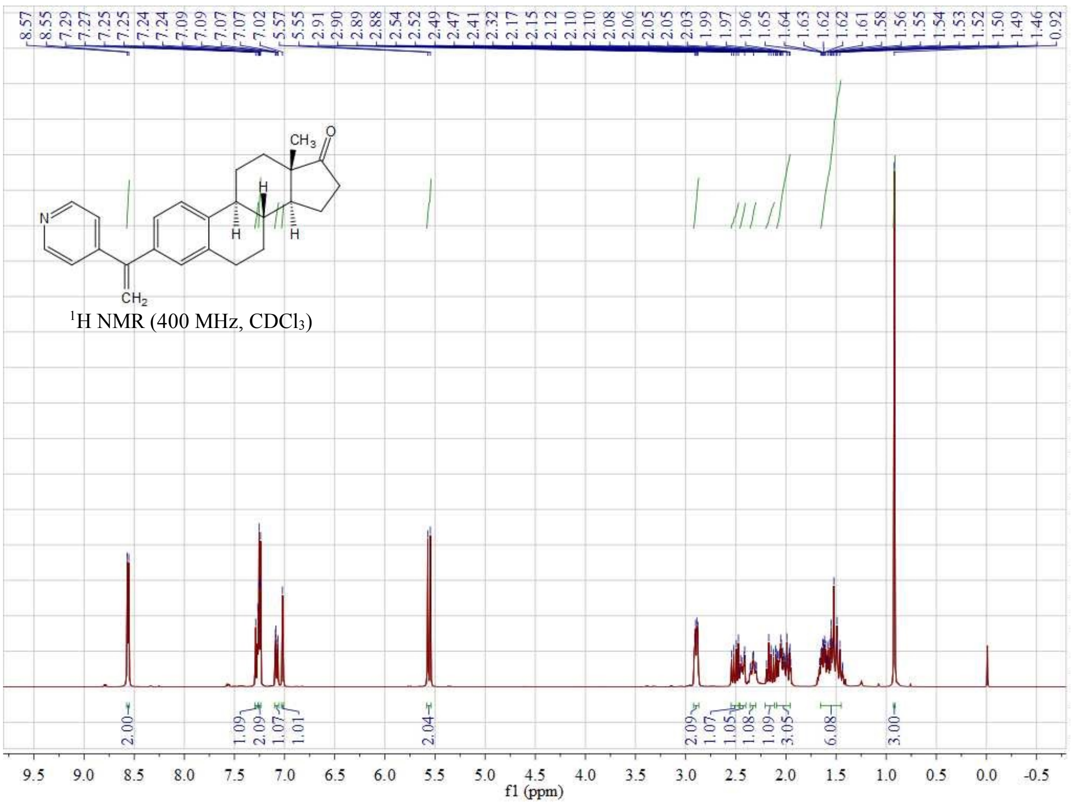

Supplementary Figure 69: ^{1}H NMR Spectra of (8R,9S,13S,14S)-13-Methyl-3-(1-(pyridin-4-yl)vinyl)-6,7,8,9,11,12,13,14,15,16-decahydro-17H-cyclopenta[a] phenanthren-17-one (44)

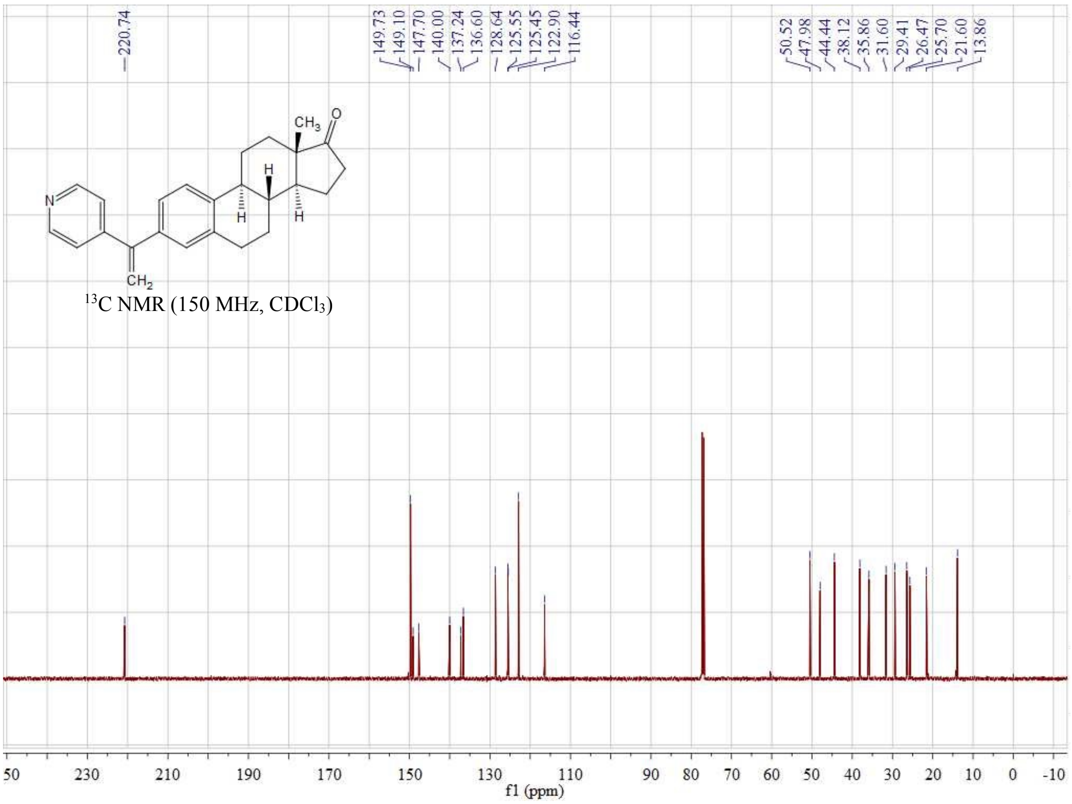

Supplementary Figure 70: ^{13}C NMR Spectra of (8R,9S,13S,14S)-13-Methyl-3-(1-

(pyridin-4-yl)vinyl)-6,7,8,9,11,12,13,14,15,16-decahydro-17H-cyclopenta [a]phenanthren-17-one (44)

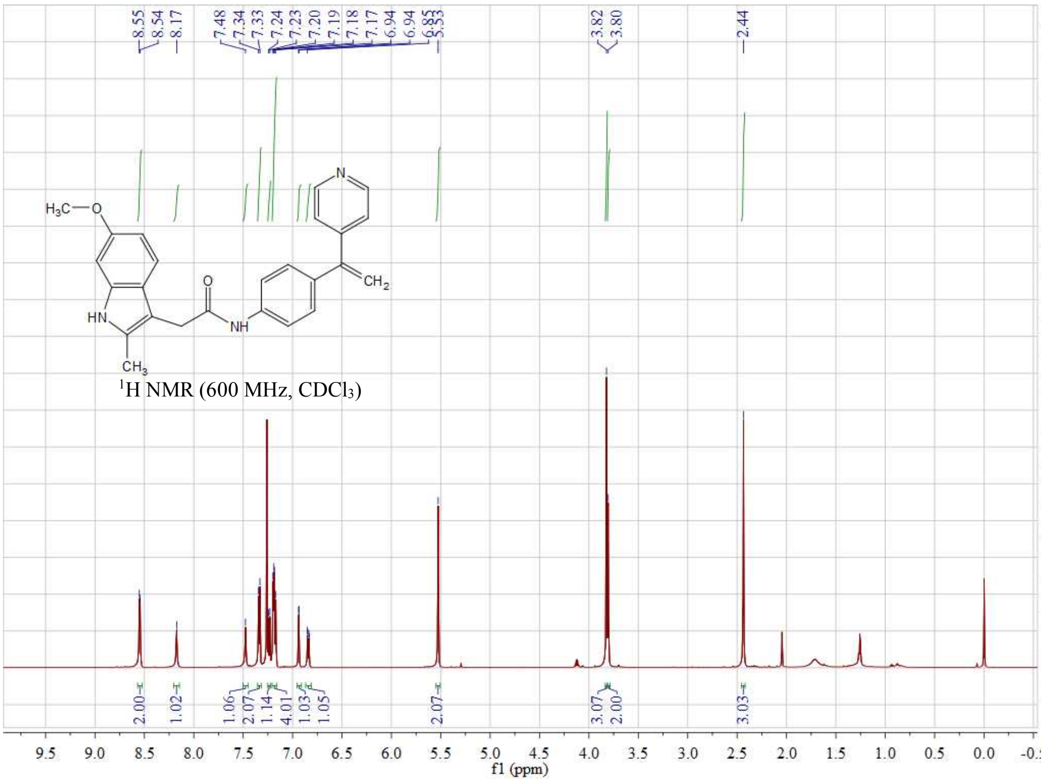

Supplementary Figure 71: ^{1}H NMR Spectra of 2-(6-methoxy-2-methyl-1H-indol-3-yl)-N-(4-(1-(pyridin-4-yl)vinyl)phenyl) acetamide (45)

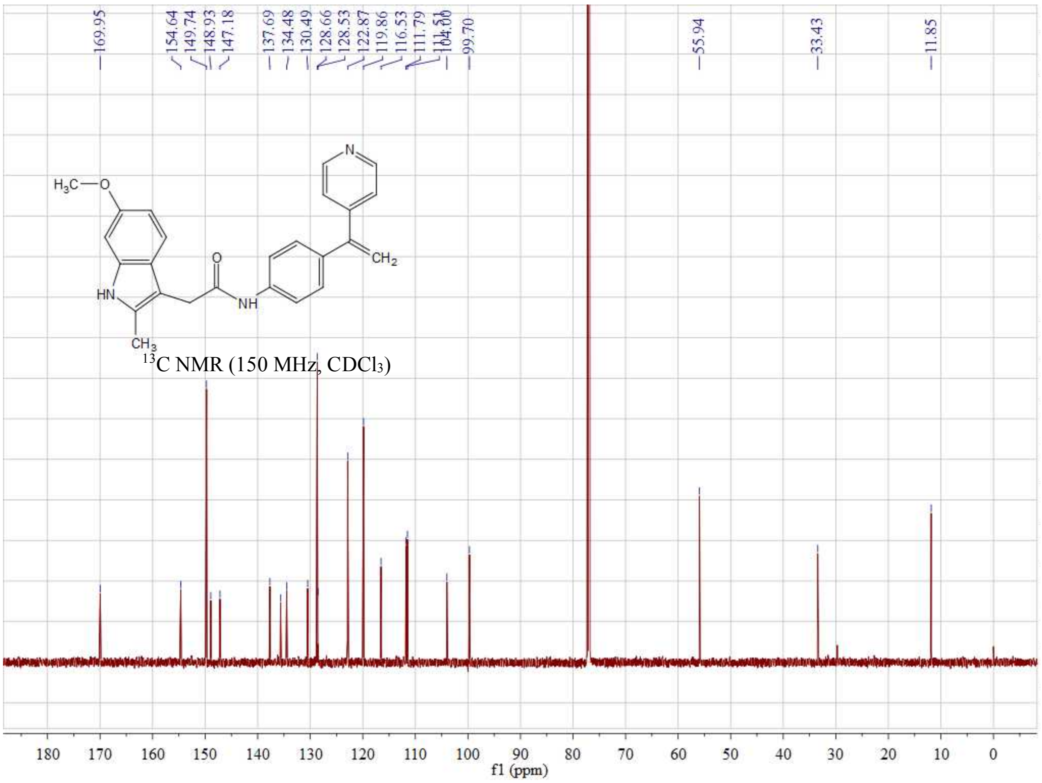

Supplementary Figure 72: ^{13}C NMR Spectra of 2-(6-methoxy-2-methyl-1H-indol-3-yl)-N-(4-(1-(pyridin-4-yl)vinyl)phenyl) acetamide (45)

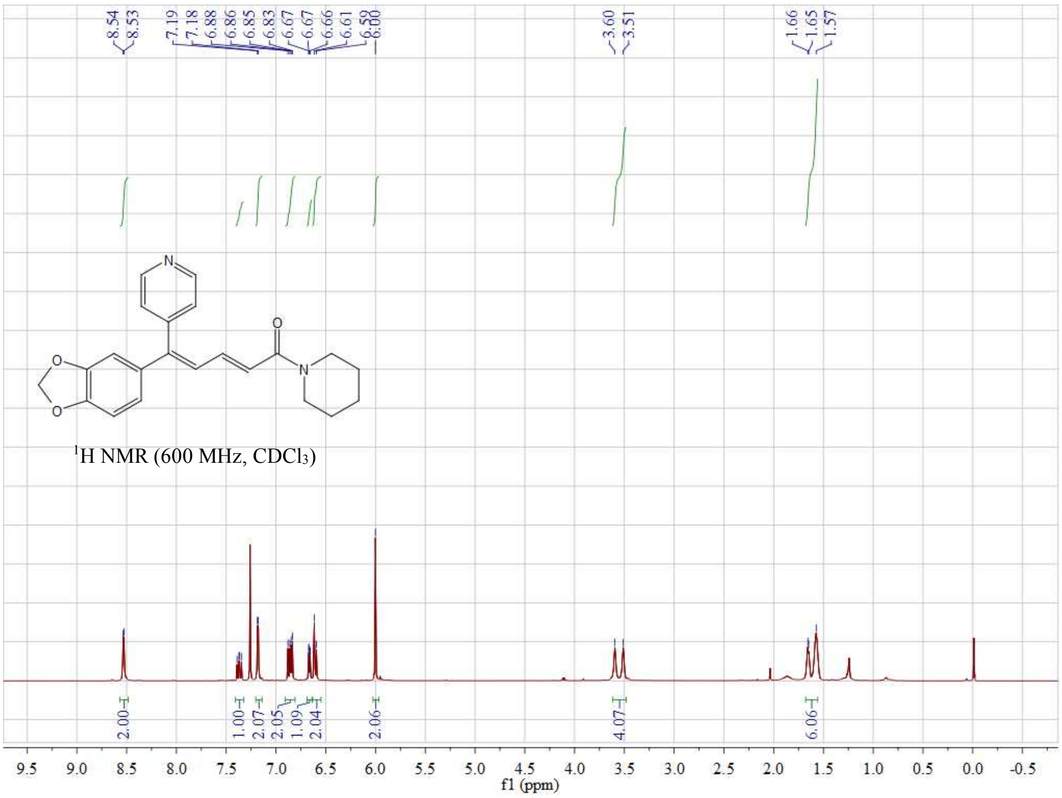

Supplementary Figure 73: ^{1}H NMR Spectra of (2E,4E)-5-(benzo[d][1,3]dioxol-5-yl)-1-(piperidin-1-yl)-5-(pyridin-4-yl)penta-2,4-dien-1-one (46)

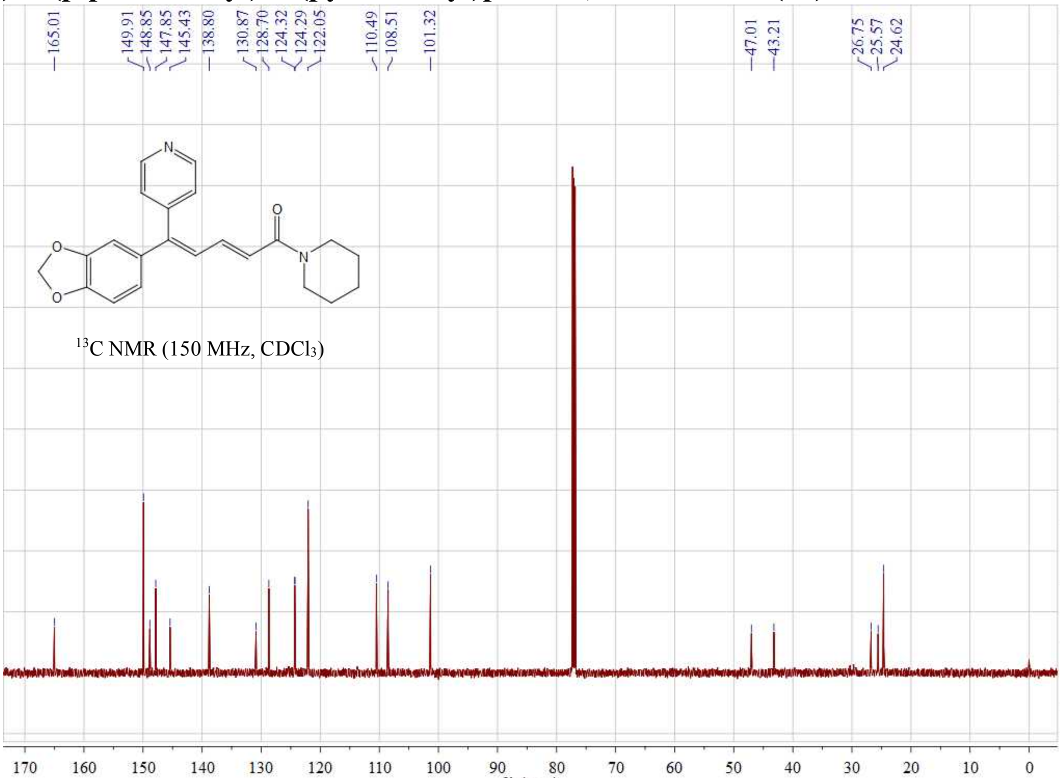

Supplementary Figure 74: ^{13}C NMR Spectra of (2E,4E)-5-(benzo[d][1,3]dioxol-5-yl)-1-(piperidin-1-yl)-5-(pyridin-4-yl)penta-2,4-dien-1-one (46)

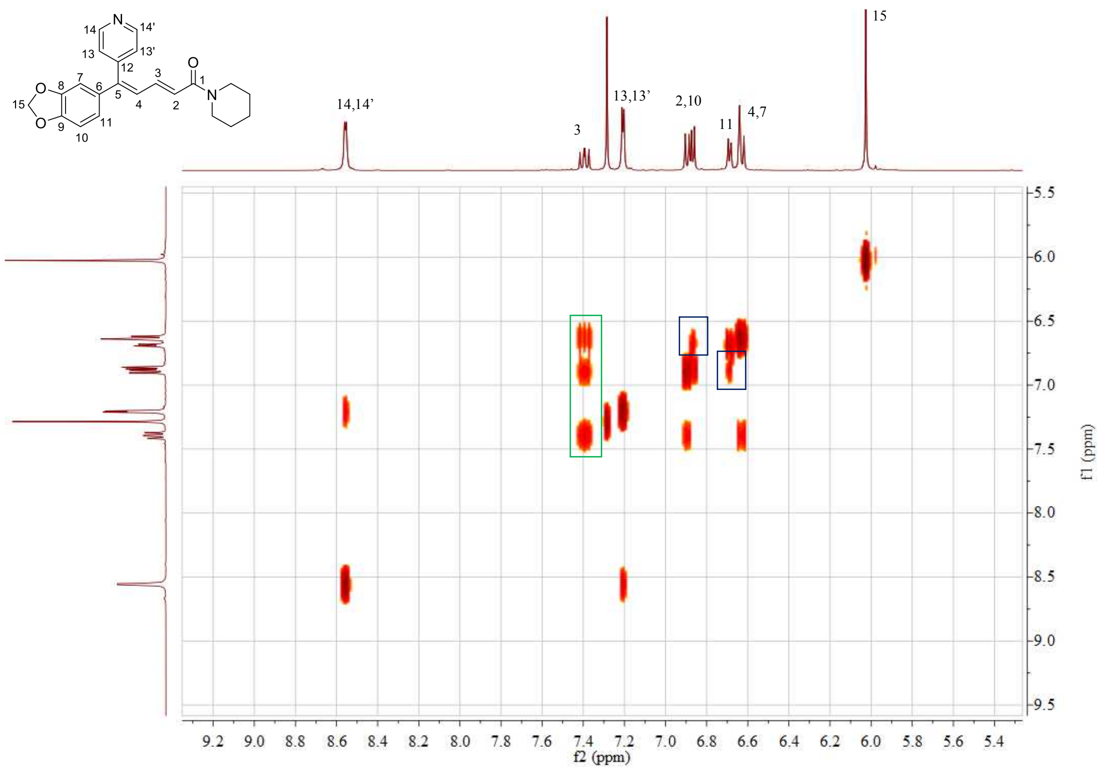

Supplementary Figure 75: H-H COSY Spectra of (2E,4E)-5-(benzo[d][1,3]dioxol-5-yl)-1-(piperidin-1-yl)-5-(pyridin-4-yl)penta-2,4-dien-1-one (46)

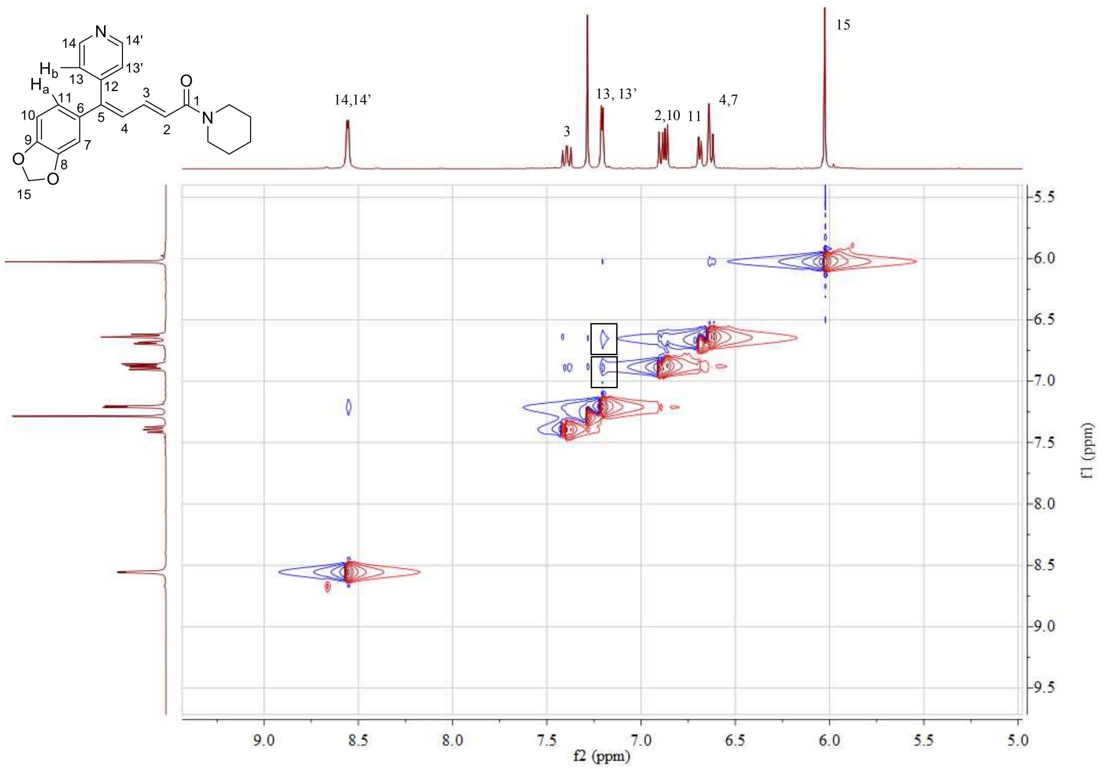

Supplementary Figure 76: NOESY Spectra of (2E,4E)-5-(benzo[d][1,3]dioxol-5-yl)-1-(piperidin-1-yl)-5-(pyridin-4-yl)penta-2,4-dien-1-one (46)

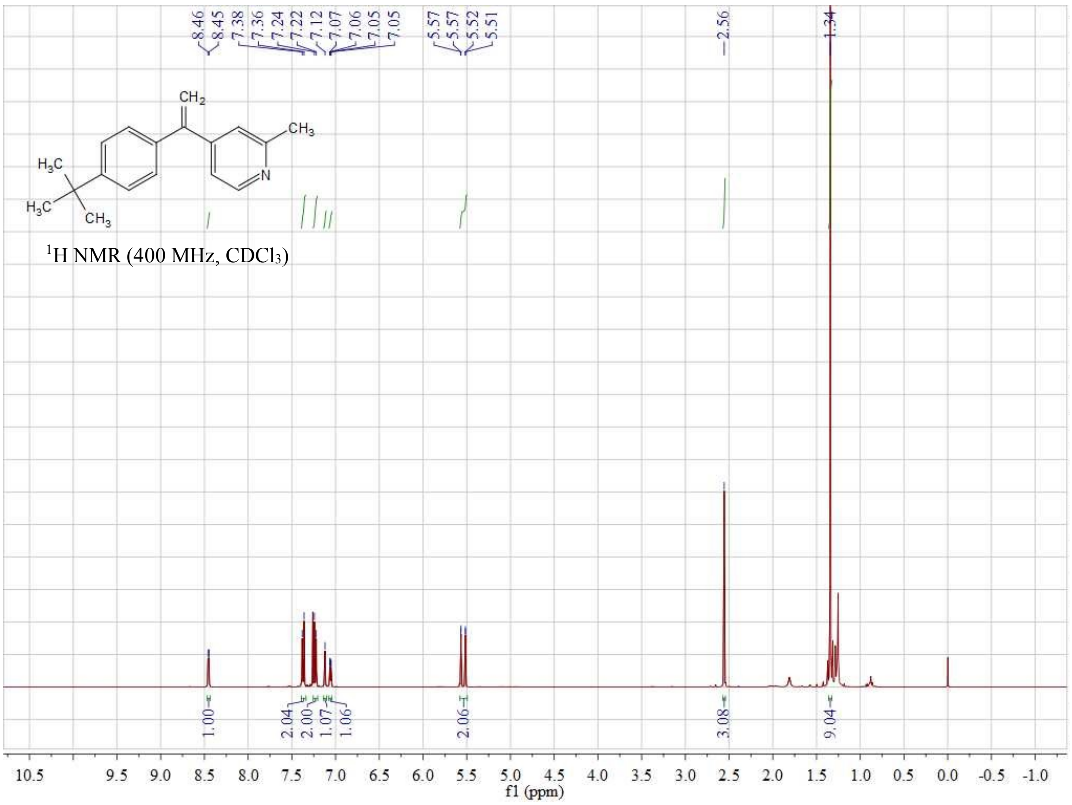

Supplementary Figure 77: ^{1}H NMR Spectra of 4-(1-(4-(*tert*-Butyl)phenyl)vinyl)-2-methylpyridine (47)

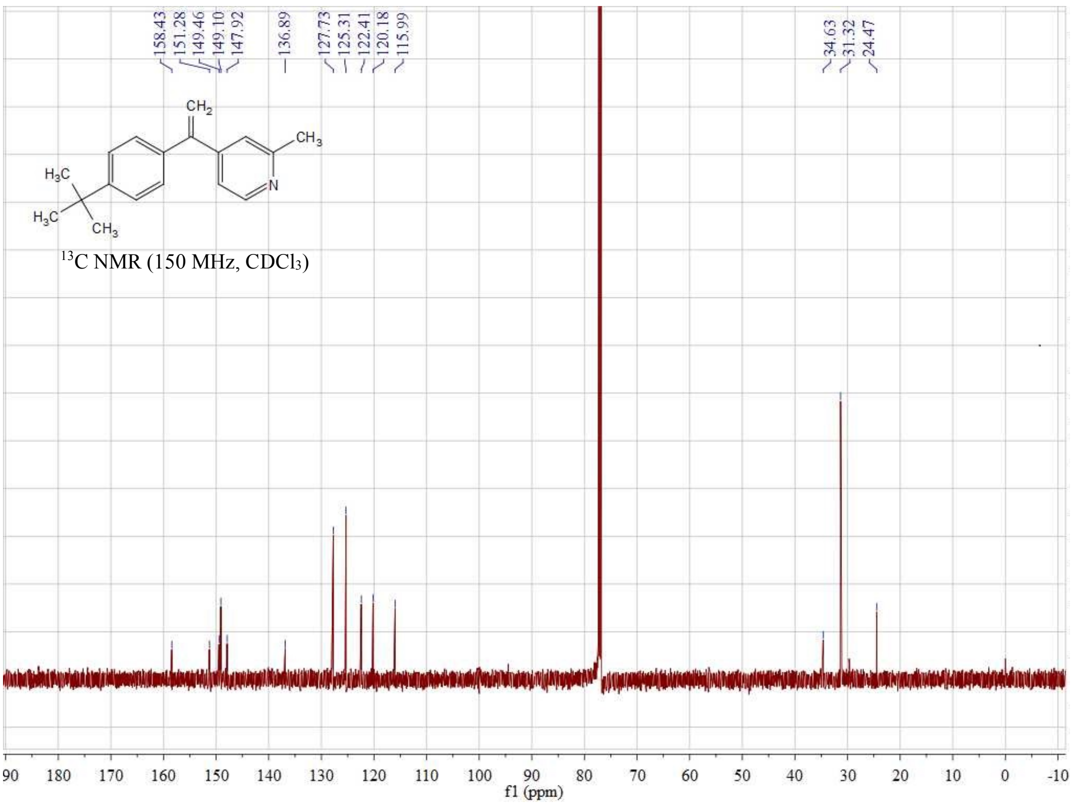

Supplementary Figure 78: ^{13}C NMR Spectra of 4-(1-(4-(*tert*-Butyl)phenyl)vinyl)-2-methylpyridine (47)

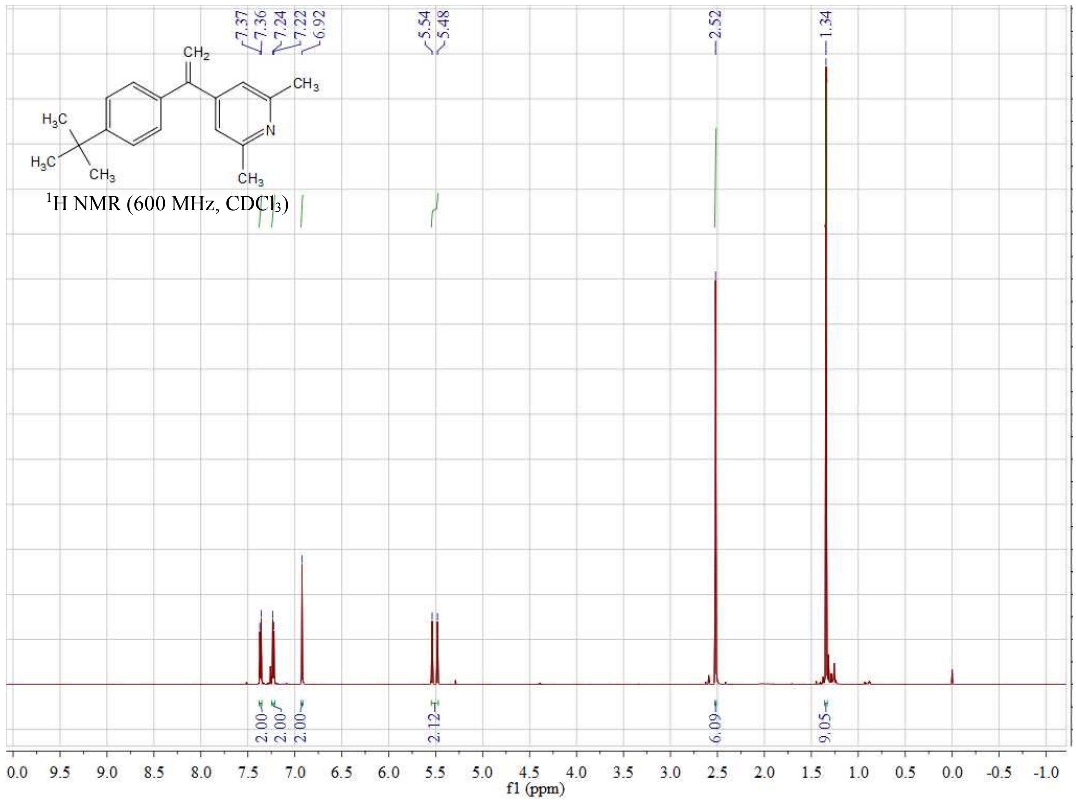

Supplementary Figure 79: ^{1}H NMR Spectra of 4-(1-(4-(*tert*-Butyl)phenyl)vinyl)-2,6-dimethylpyridine (48)

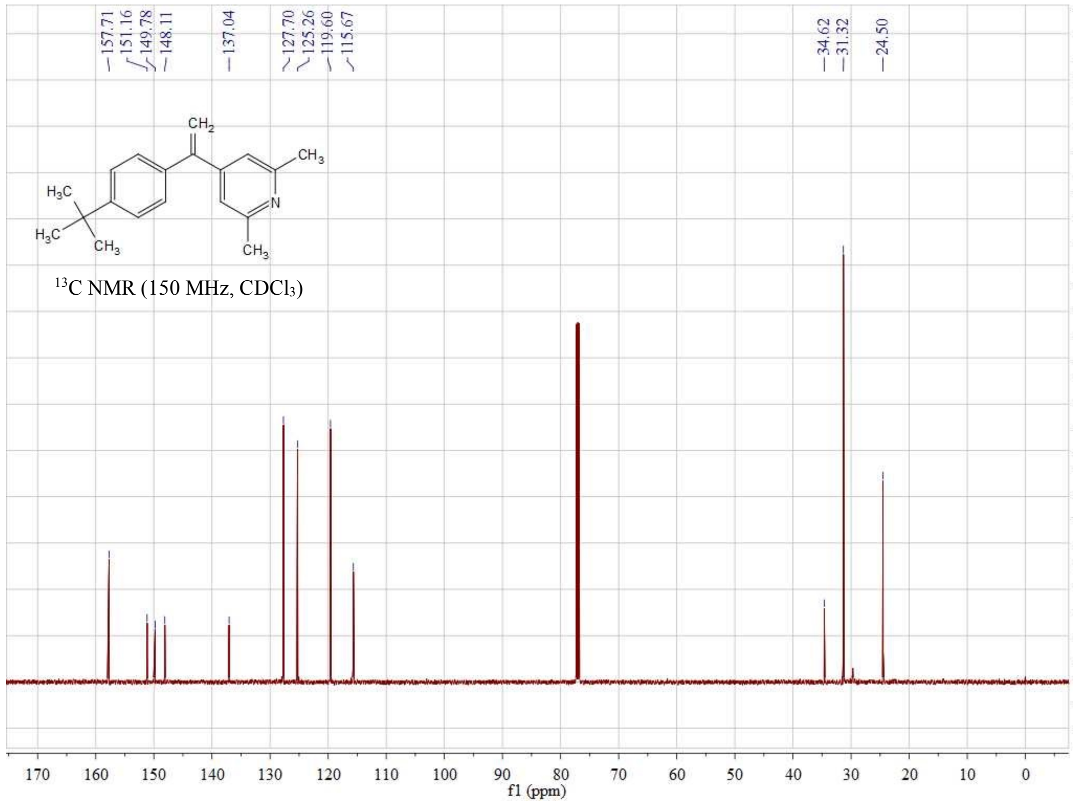

Supplementary Figure 80: ^{13}C NMR Spectra of 4-(1-(4-(*tert*-Butyl)phenyl)vinyl)-2,6-dimethylpyridine (48)

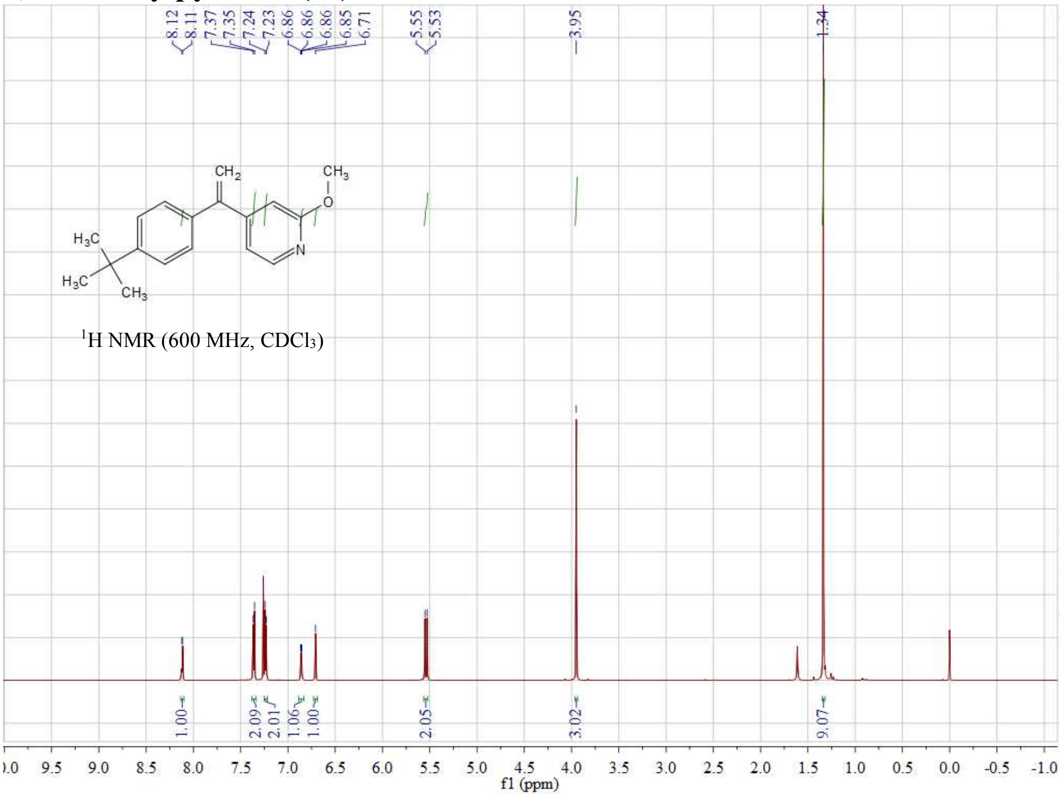

Supplementary Figure 81: ^{1}H NMR Spectra of 4-(1-(4-(tert-butyl)phenyl)vinyl)-2-methoxypyridine (49)

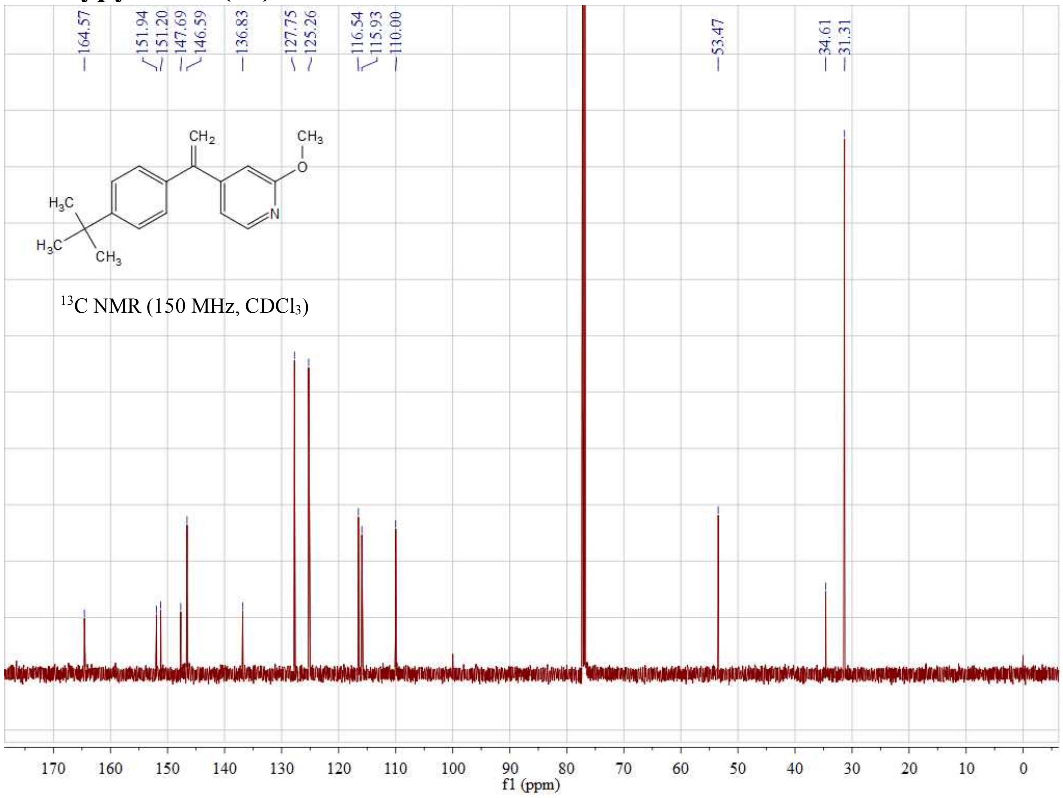

Supplementary Figure 82: ^{13}C NMR Spectra of 4-(1-(4-(tert-butyl)phenyl)vinyl)-2-methoxypyridine (49)

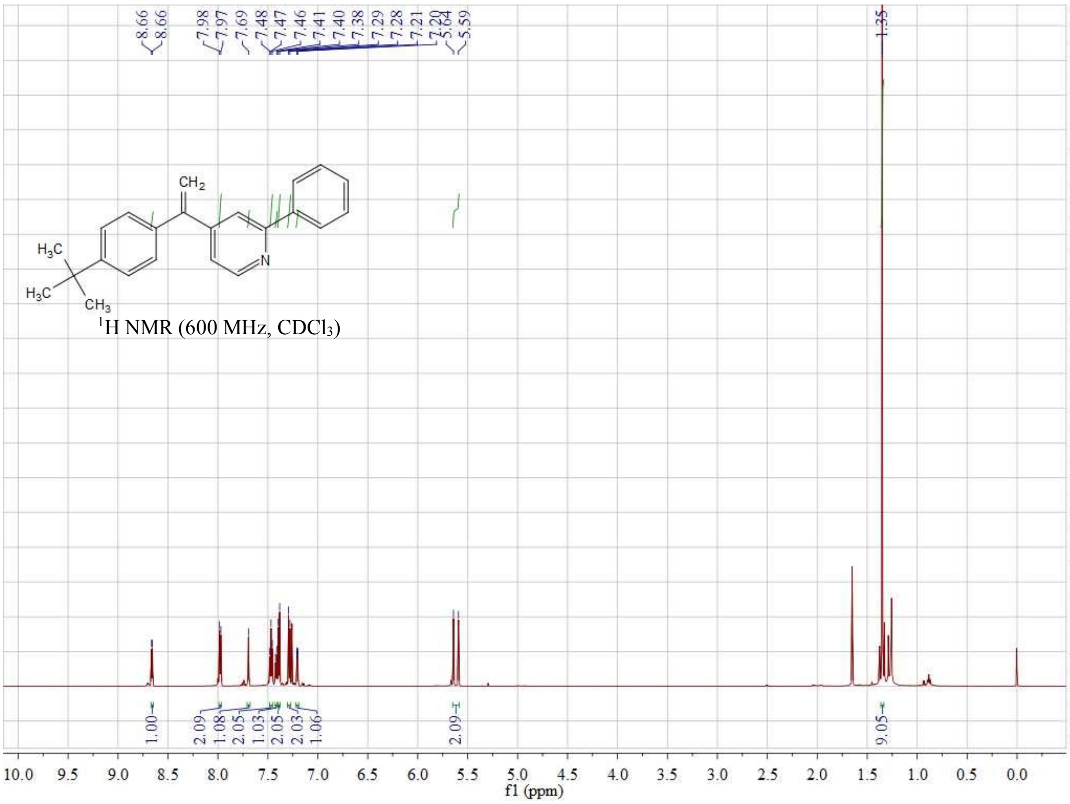

Supplementary Figure 83: ^{1}H NMR Spectra of 4-(1-(4-(*tert*-Butyl)phenyl)vinyl)-2-phenylpyridine (50)

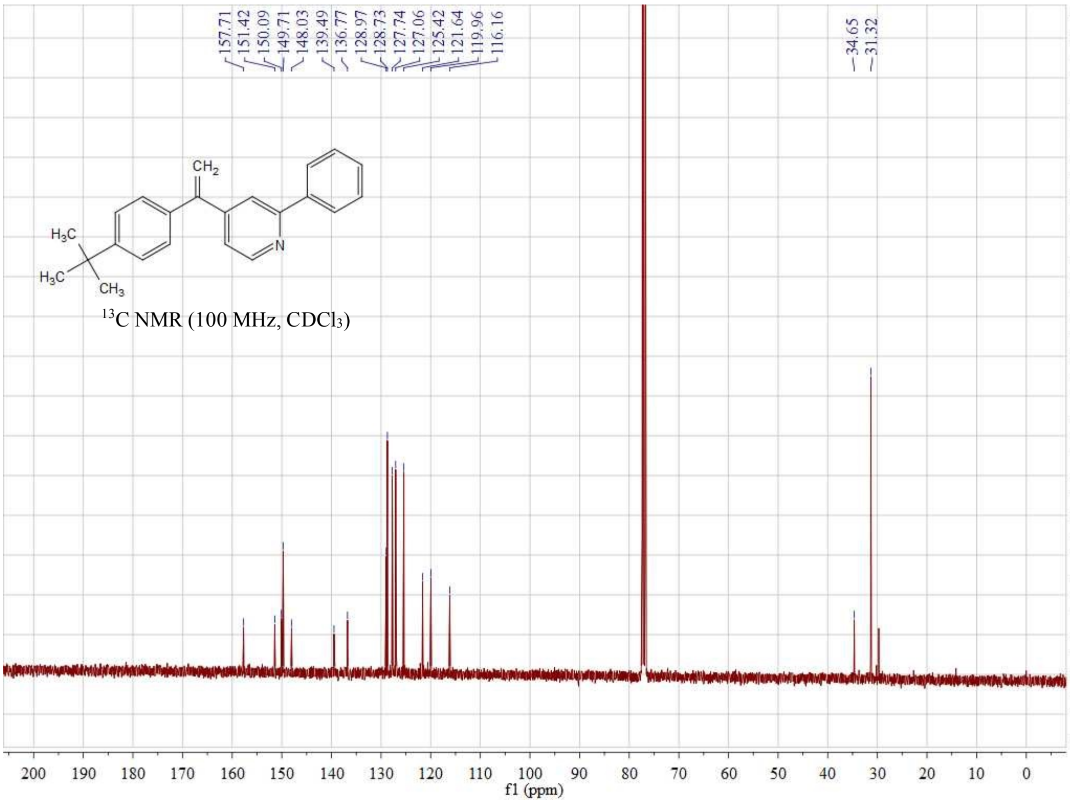

Supplementary Figure 84: ^{13}C NMR Spectra of 4-(1-(4-(*tert*-Butyl)phenyl)vinyl)-2-phenylpyridine (50)

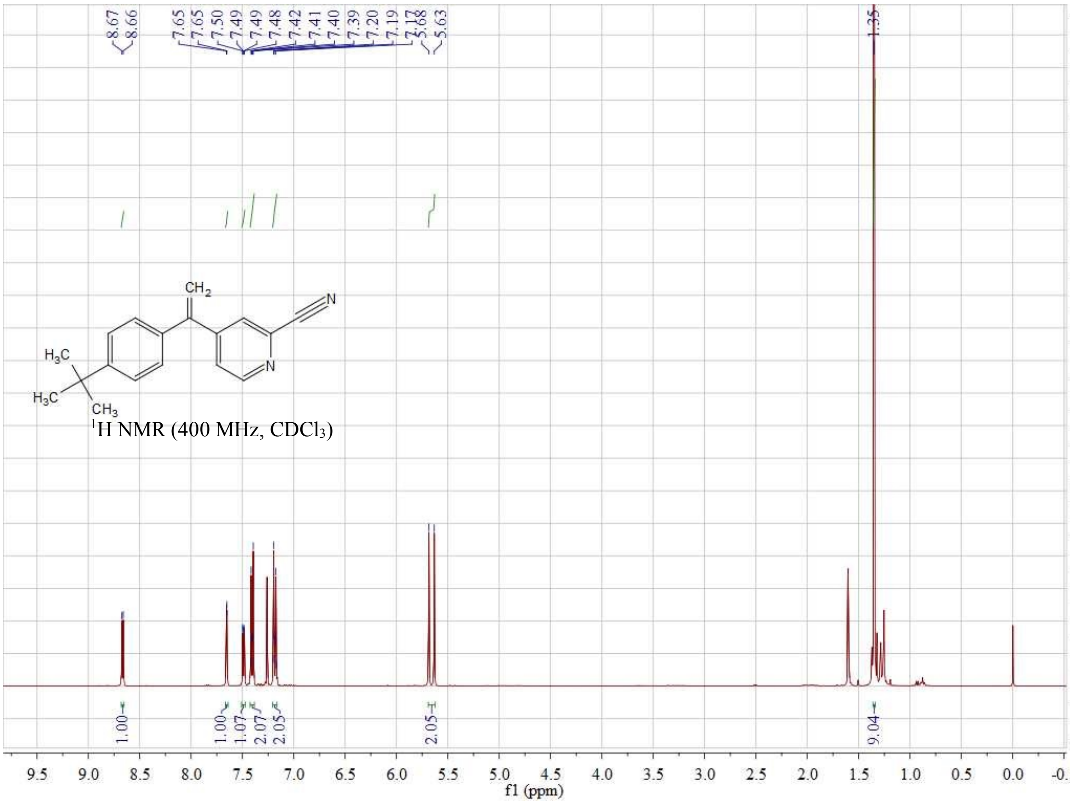

Supplementary Figure 85: ^{1}H NMR Spectra of 2-(1-(4-(*tert*-Butyl)phenyl)vinyl) isonicotinonitrile (51)

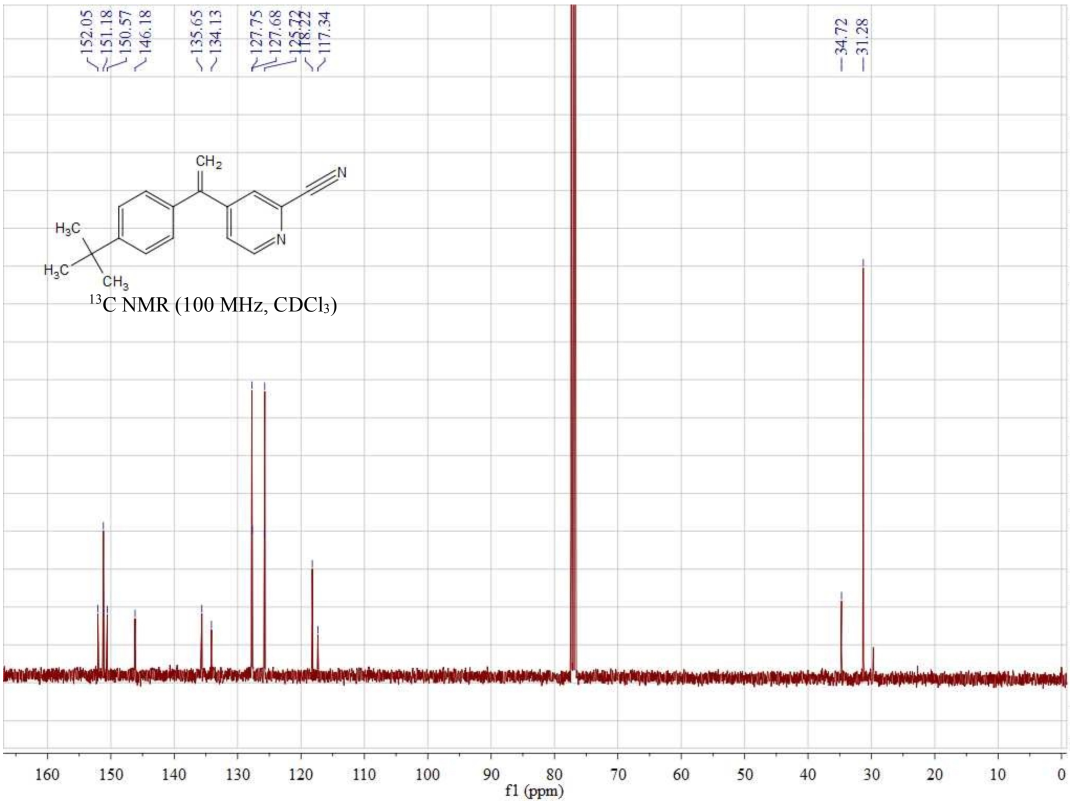

Supplementary Figure 86: ^{13}C NMR Spectra of 2-(1-(4-(*tert*-Butyl)phenyl)vinyl) isonicotinonitrile (51)

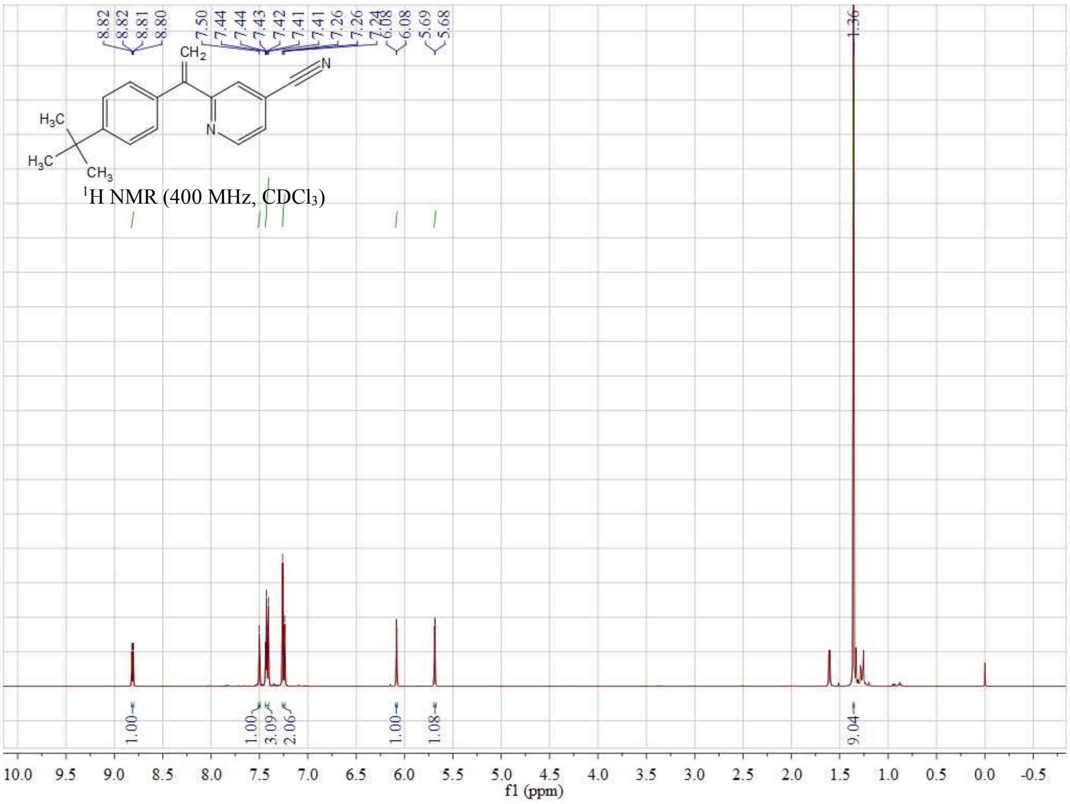

Supplementary Figure 87: ^{1}H NMR Spectra of 4-(1-(4-(*tert*-butyl)phenyl)vinyl) picolinonitrile (51)

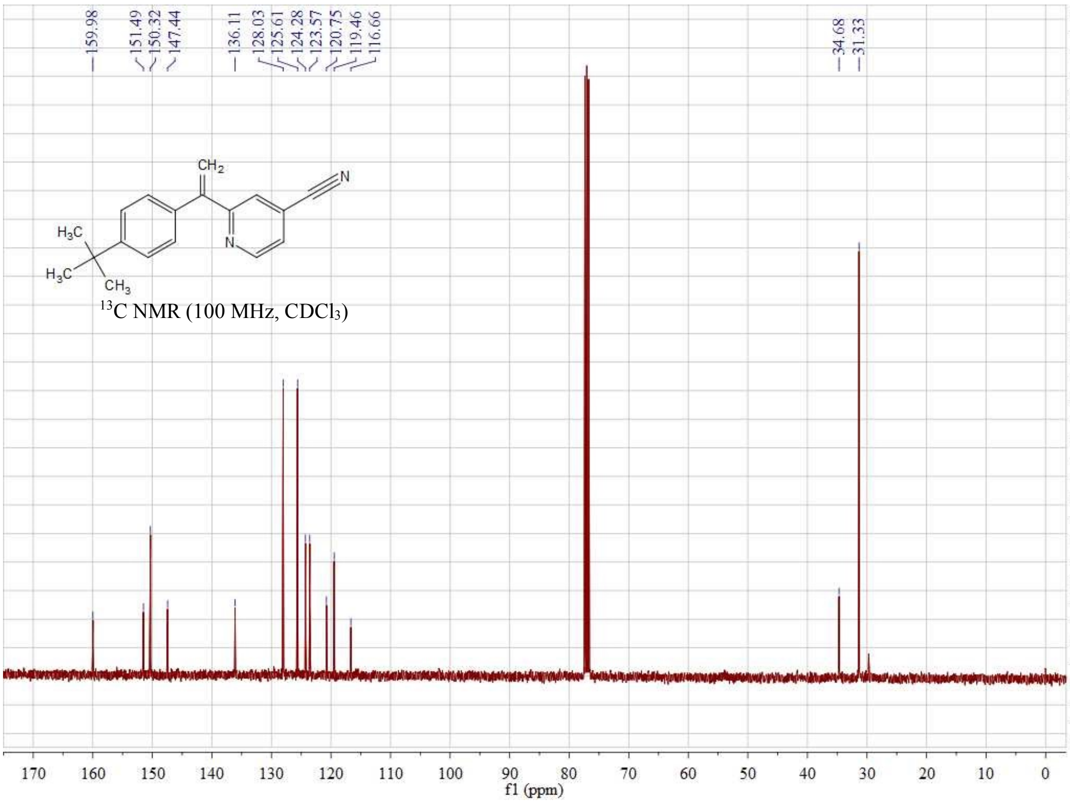

Supplementary Figure 88: ^{13}C NMR Spectra of 4-(1-(4-(*tert*-butyl)phenyl)vinyl) picolinonitrile (51)

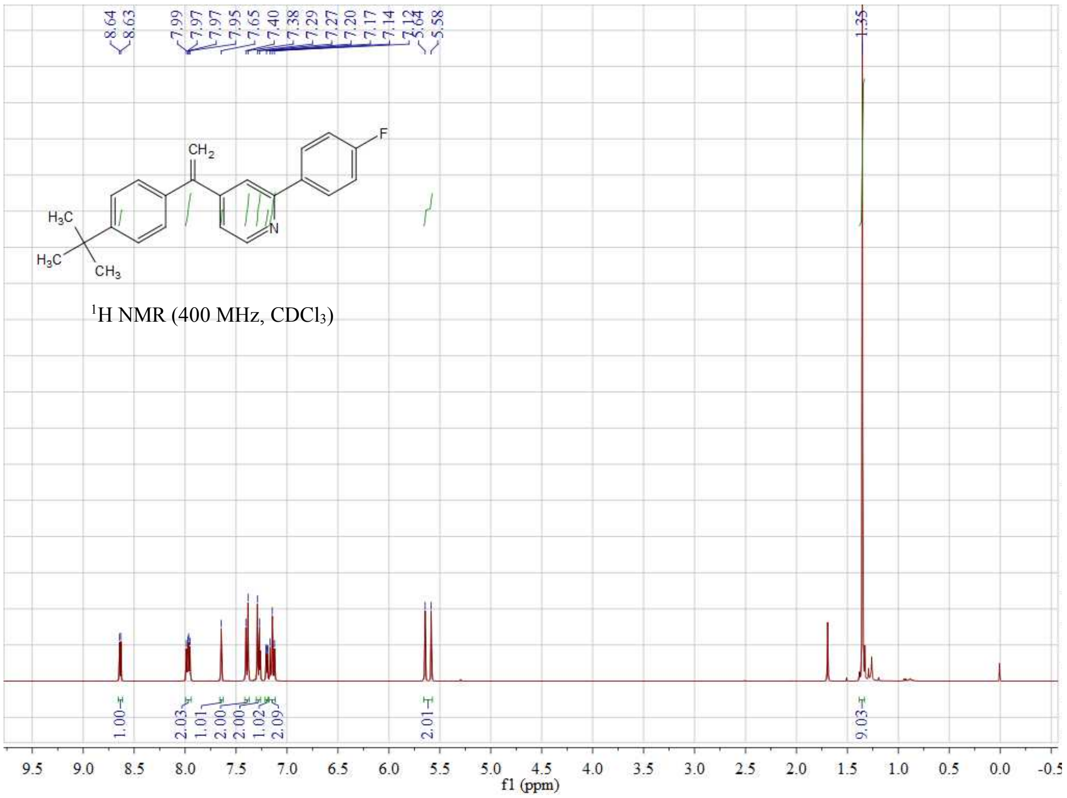

Supplementary Figure 89: ^{1}H NMR Spectra of 4-(1-(4-(tert-butyl)phenyl)vinyl)-2-(4-fluorophenyl)pyridine (52)

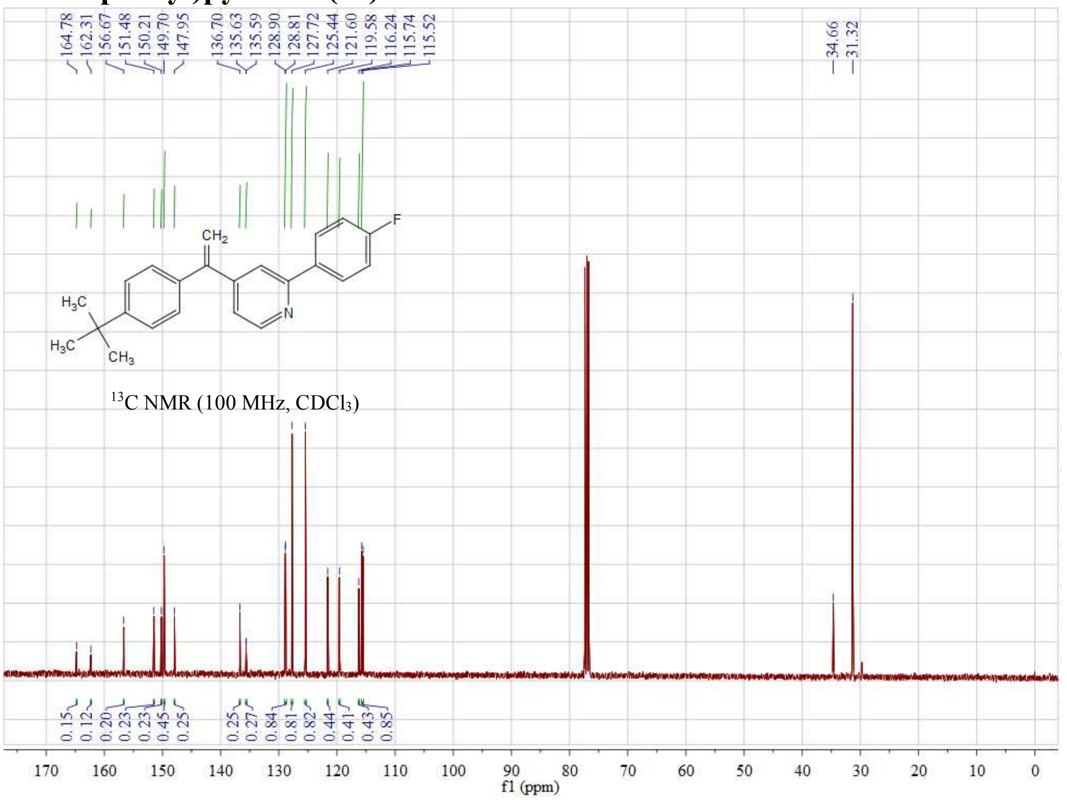

Supplementary Figure 90: ^{13}C NMR Spectra of 4-(1-(4-(tert-butyl)phenyl)vinyl)-2-(4-fluorophenyl)pyridine (52)

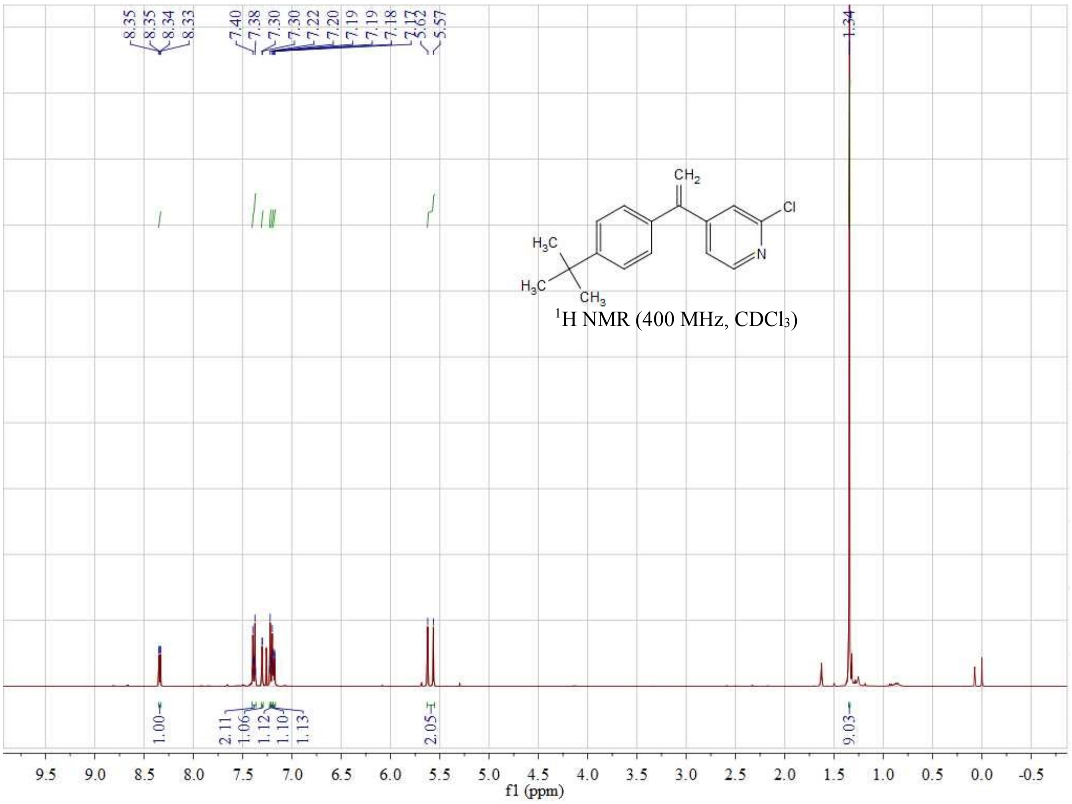

Supplementary Figure 91: ^{1}H NMR Spectra of 4-(1-(4-(*tert*-Butyl)phenyl)vinyl)-2-chloropyridine (53)

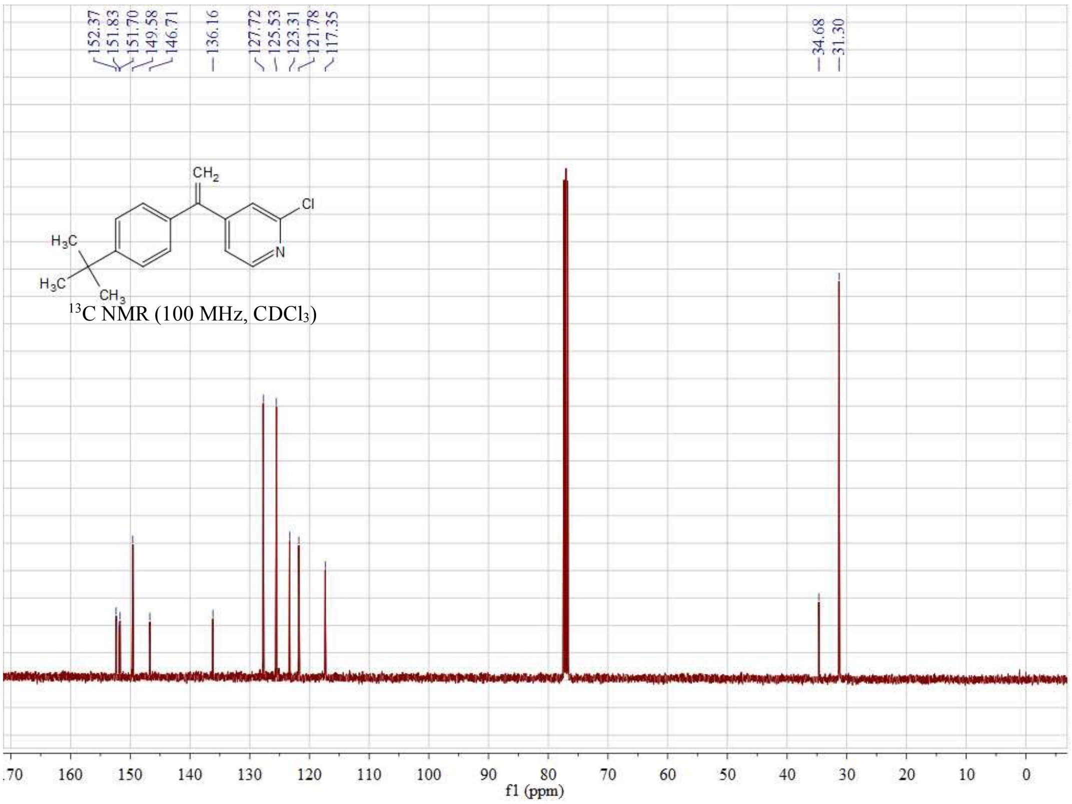

Supplementary Figure 92: ^{13}C NMR Spectra of 4-(1-(4-(*tert*-Butyl)phenyl)vinyl)-2-chloropyridine (53)

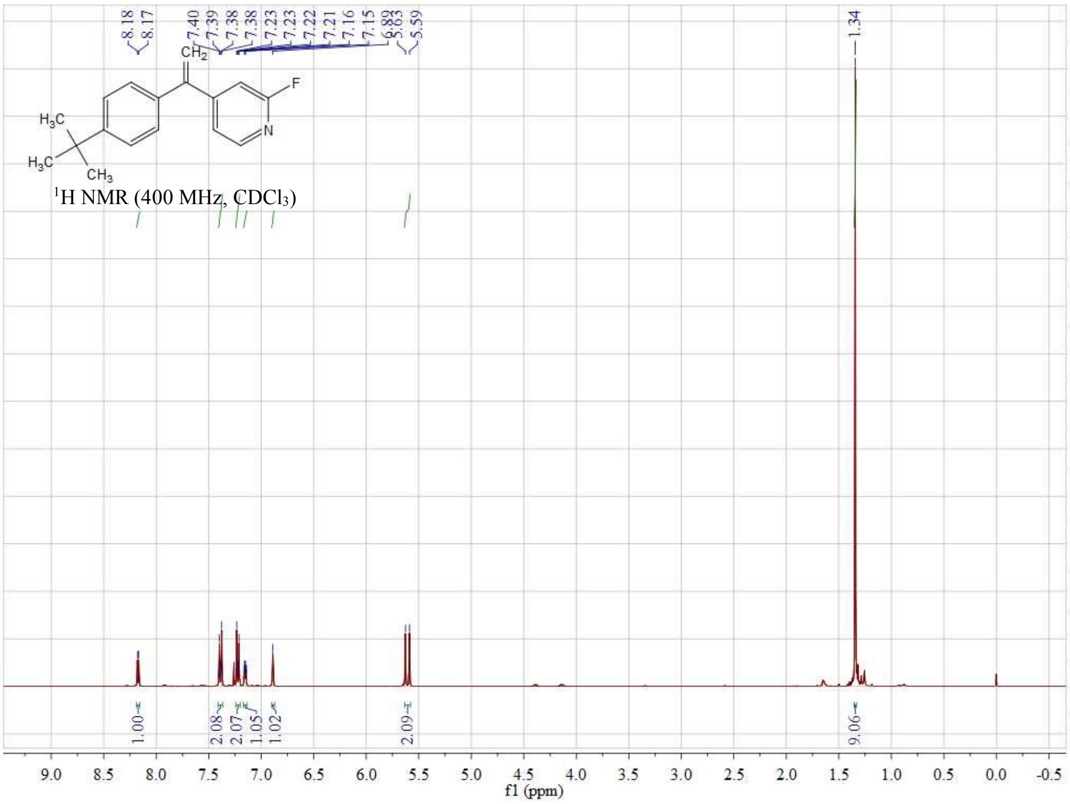

Supplementary Figure 93: ^{1}H NMR Spectra of 4-(1-(4-(*tert*-Butyl)phenyl)vinyl)-2-fluoropyridine (54)

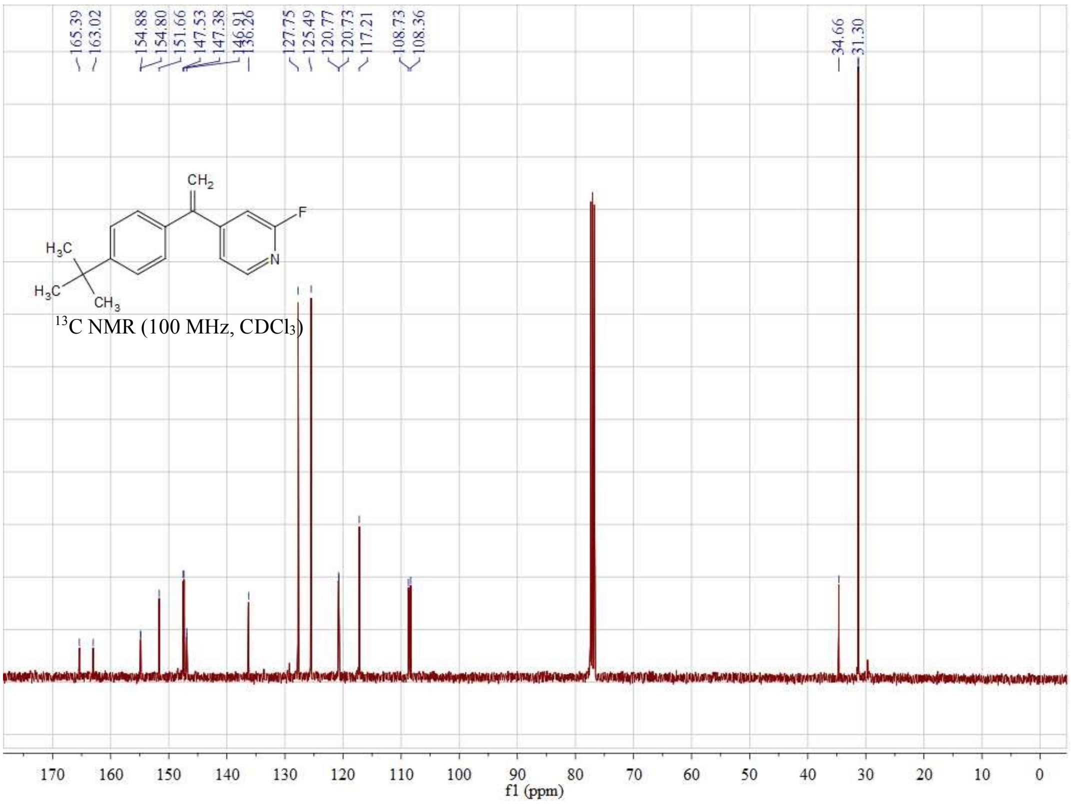

Supplementary Figure 94: ^{13}C NMR Spectra of 4-(1-(4-(*tert*-Butyl)phenyl)vinyl)-2-fluoropyridine (54)

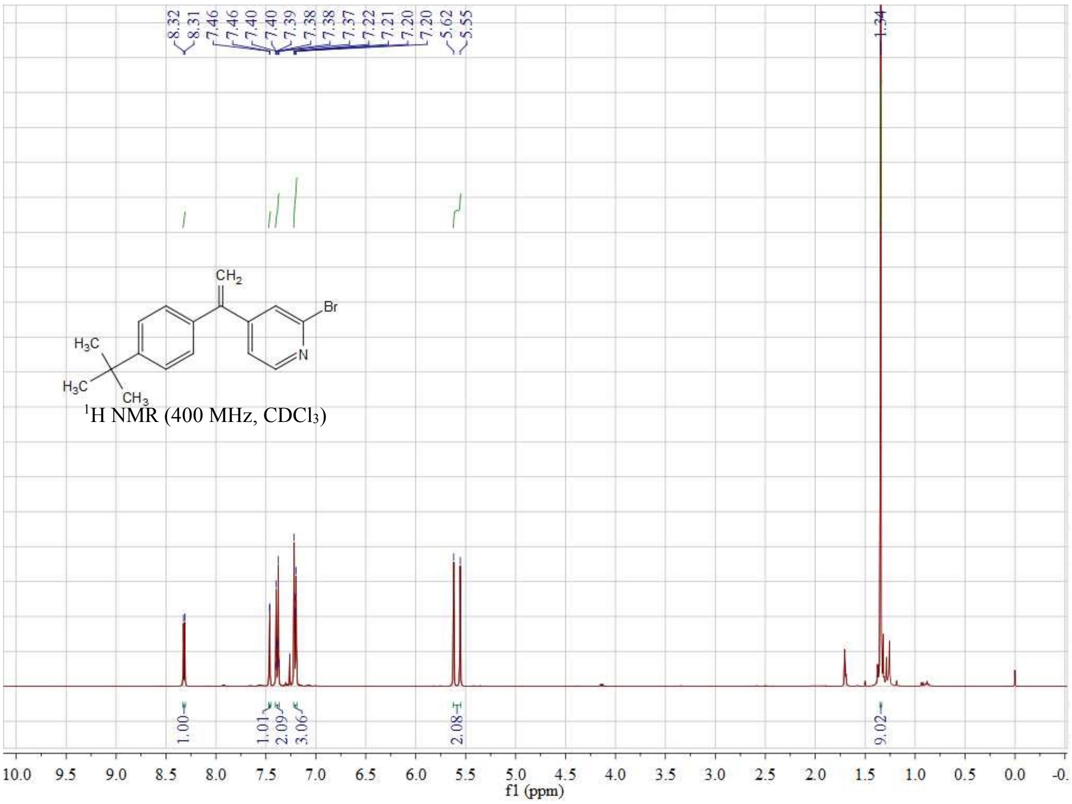

Supplementary Figure 95: ^{1}H NMR Spectra of 2-Bromo-4-(1-(4-(*tert*-butyl)phenyl)vinyl)pyridine (55)

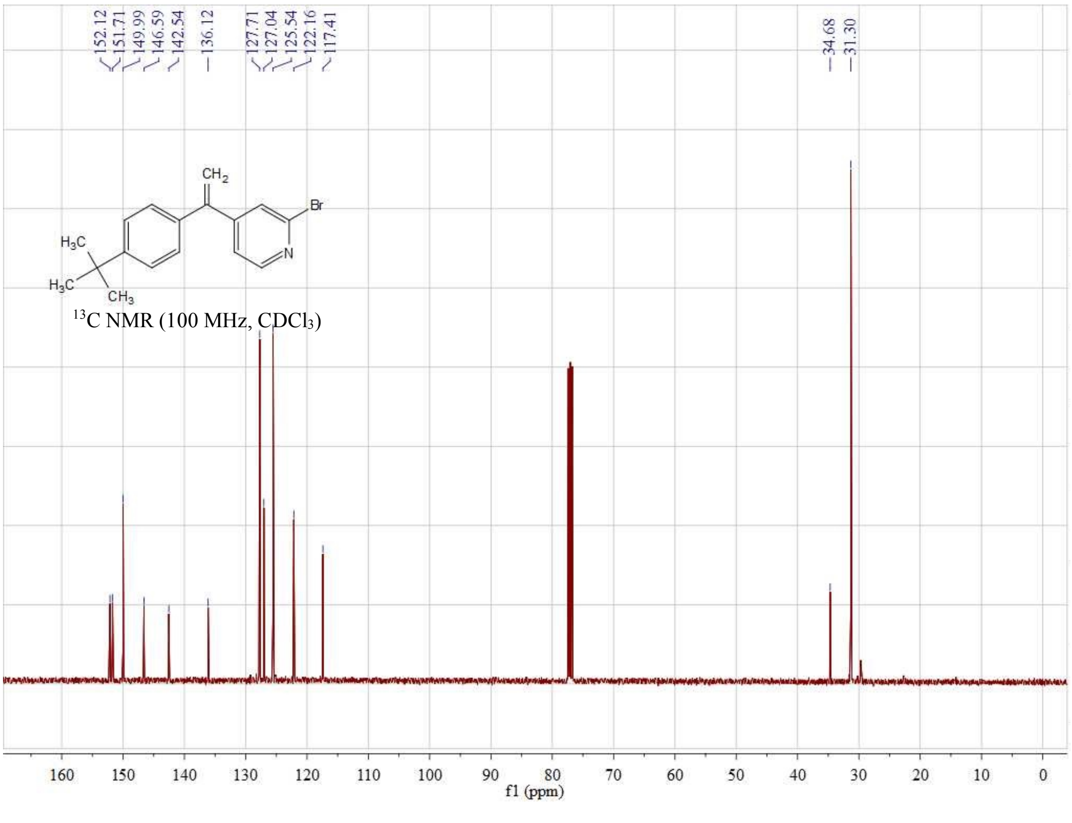

Supplementary Figure 96: ^{13}C NMR Spectra of 2-Bromo-4-(1-(4-(*tert*-butyl)phenyl)vinyl)pyridine (55)

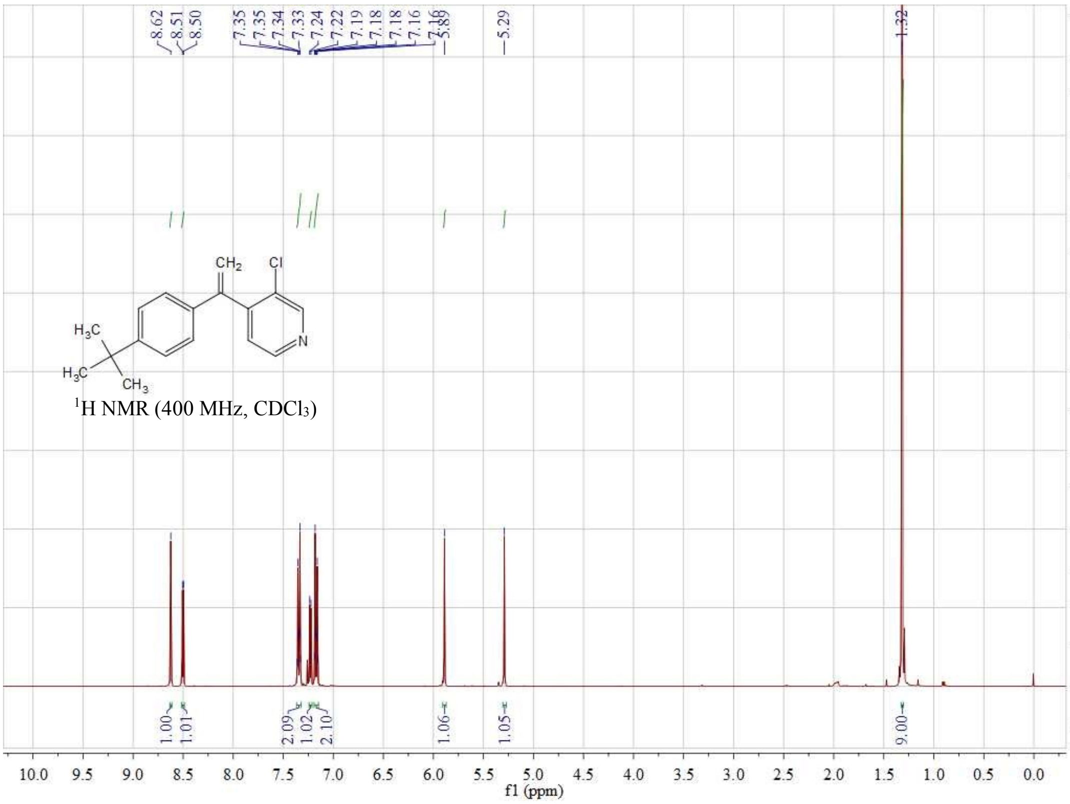

Supplementary Figure 97: ^{1}H NMR Spectra of 4-(1-(4-(*tert*-Butyl)phenyl)vinyl)-3-chloropyridine (56)

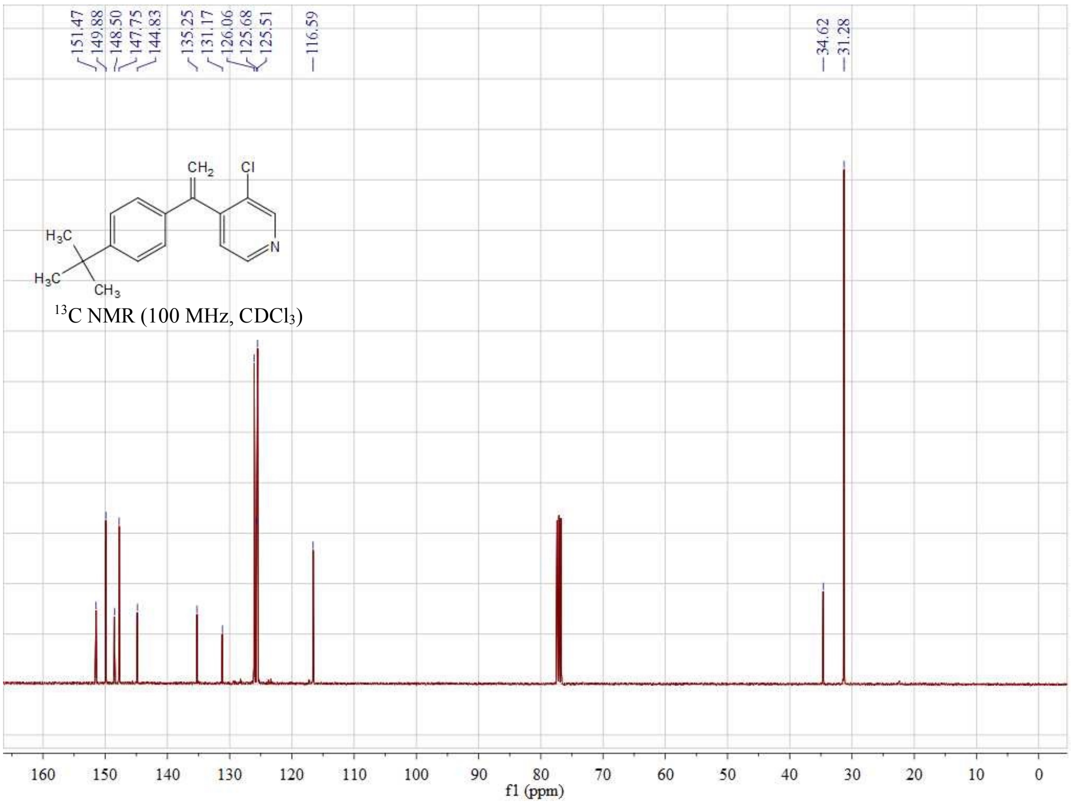

Supplementary Figure 98: ^{13}C NMR Spectra of 4-(1-(4-(*tert*-Butyl)phenyl)vinyl)-3-chloropyridine (56)

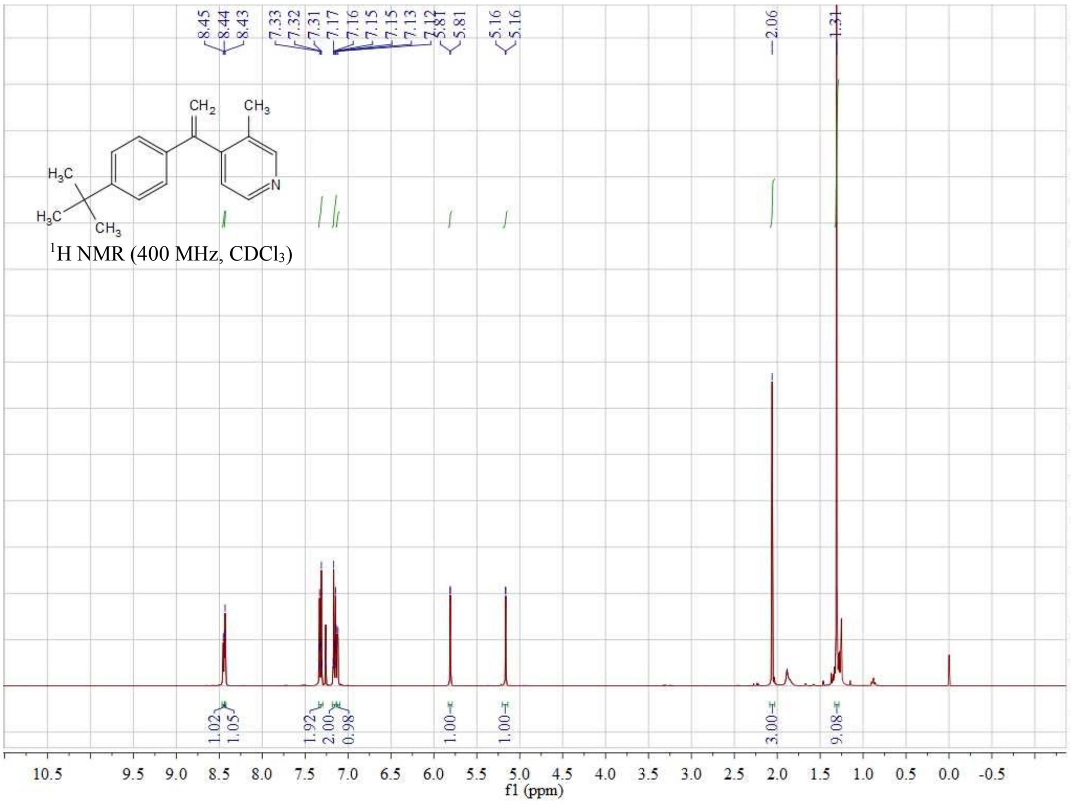

Supplementary Figure 99: ^{1}H NMR Spectra of 4-(1-(4-(*tert*-Butyl)phenyl)vinyl)-3-methylpyridine (57)

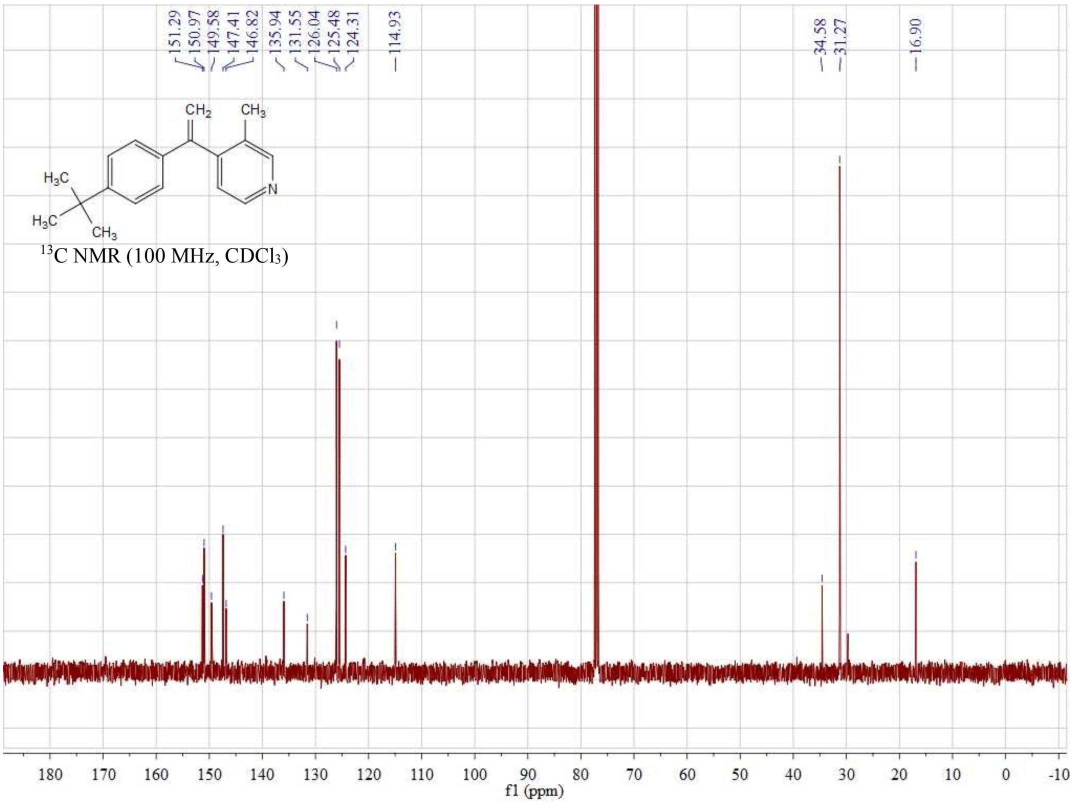

Supplementary Figure 100: ^{13}C NMR Spectra of 4-(1-(4-(*tert*-Butyl)phenyl)vinyl)-3-methylpyridine (57)

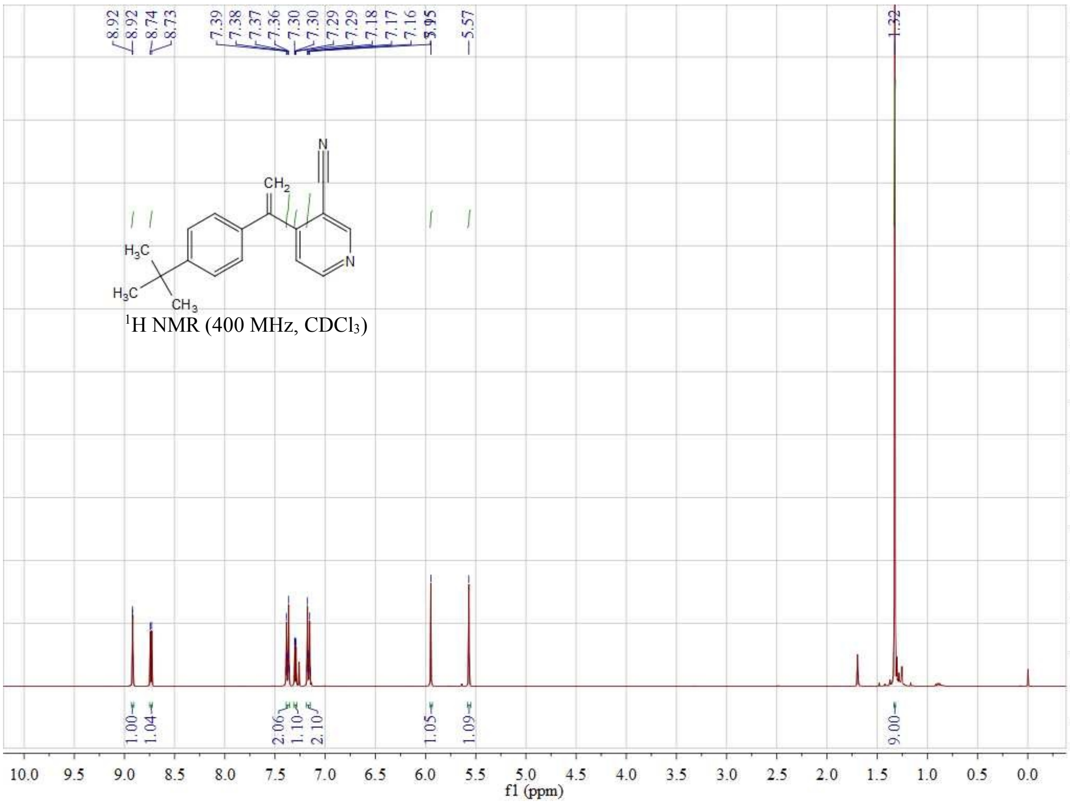

Supplementary Figure 101: ^{1}H NMR Spectra of 4-(1-(4-(*tert*-Butyl)phenyl)vinyl) nicotinonitrile (58)

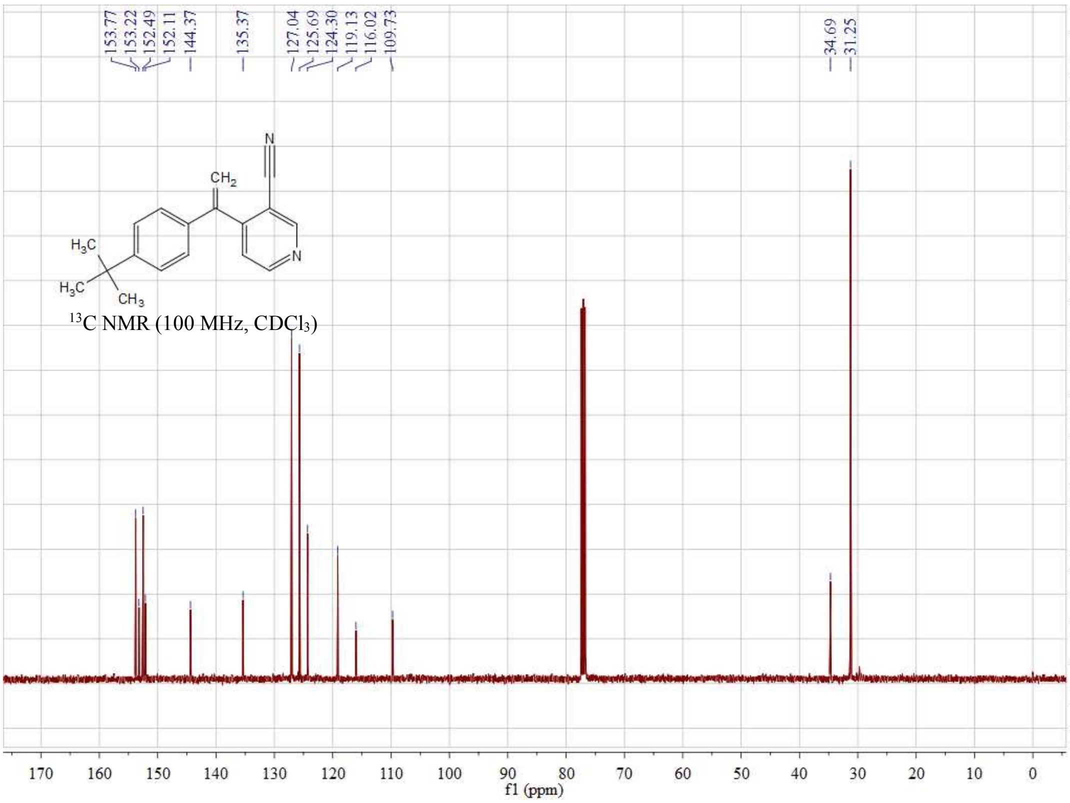

Supplementary Figure 102: ^{13}C NMR Spectra of 4-(1-(4-(*tert*-Butyl)phenyl)vinyl) nicotinonitrile (58)

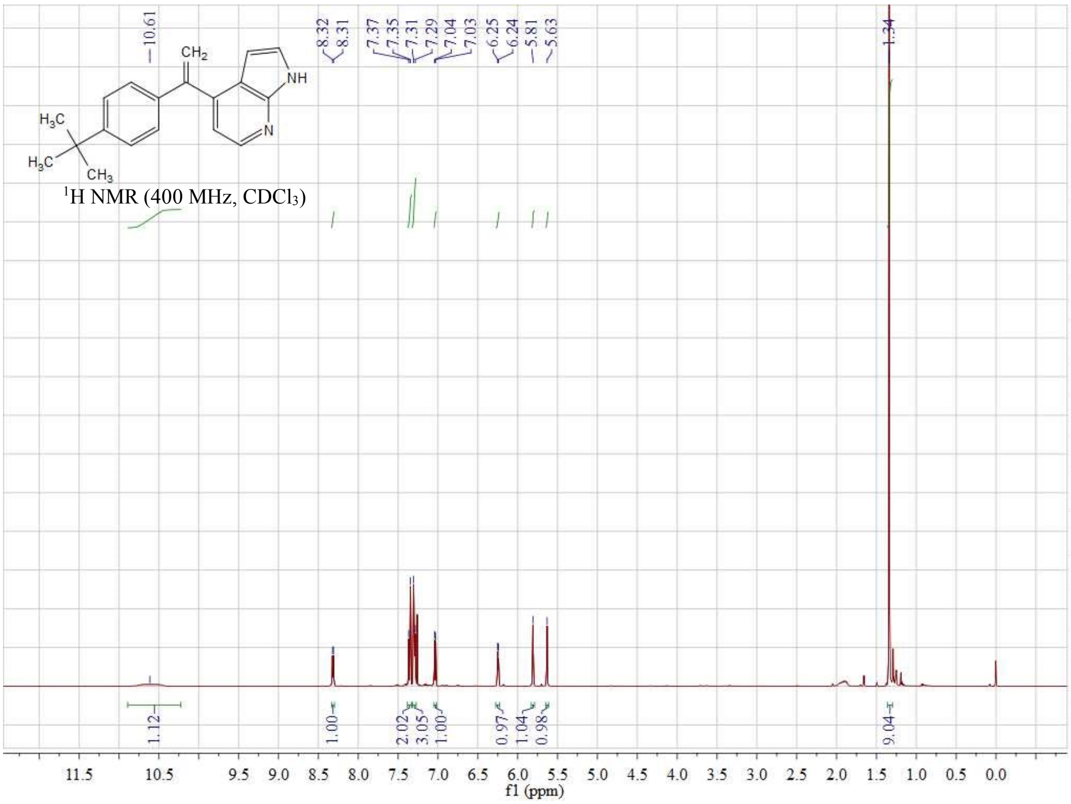

Supplementary Figure 103: ¹H NMR Spectra of 4-(1-(4-(*tert*-Butyl)phenyl)vinyl)-1H-pyrrolo[2,3-b]pyridine (59)

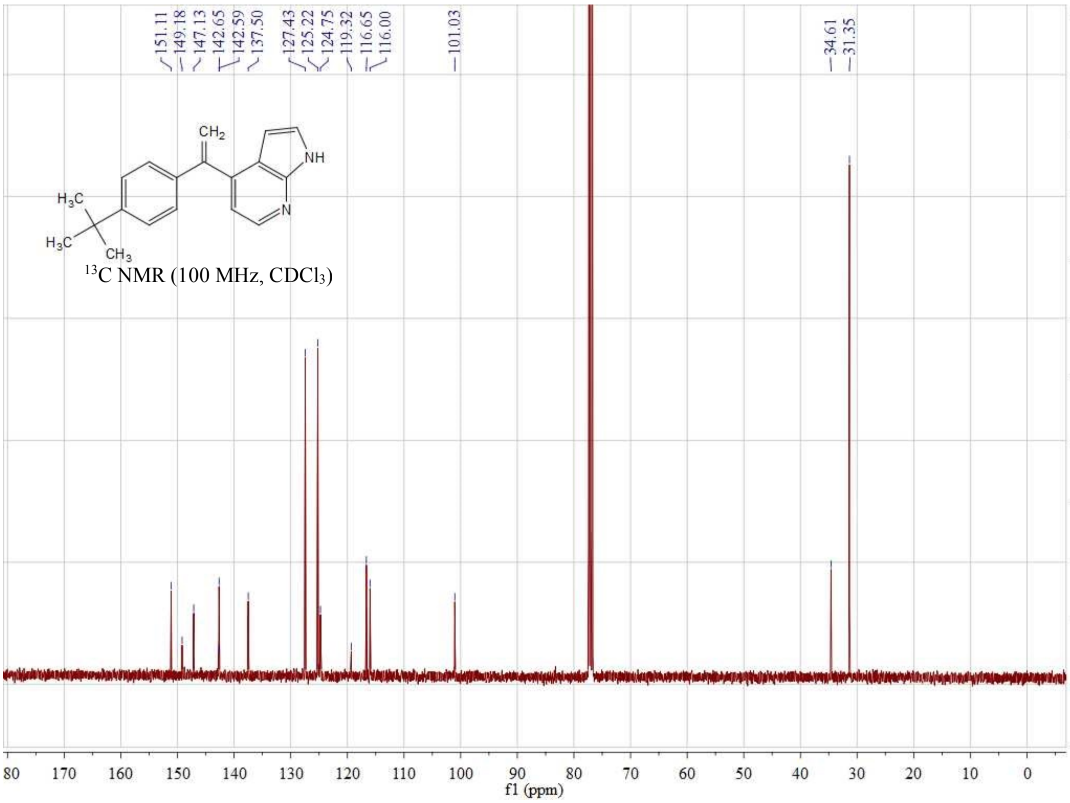

Supplementary Figure 104: ¹³C NMR Spectra of 4-(1-(4-(*tert*-Butyl)phenyl)vinyl)-1H-pyrrolo[2,3-b]pyridine (59)

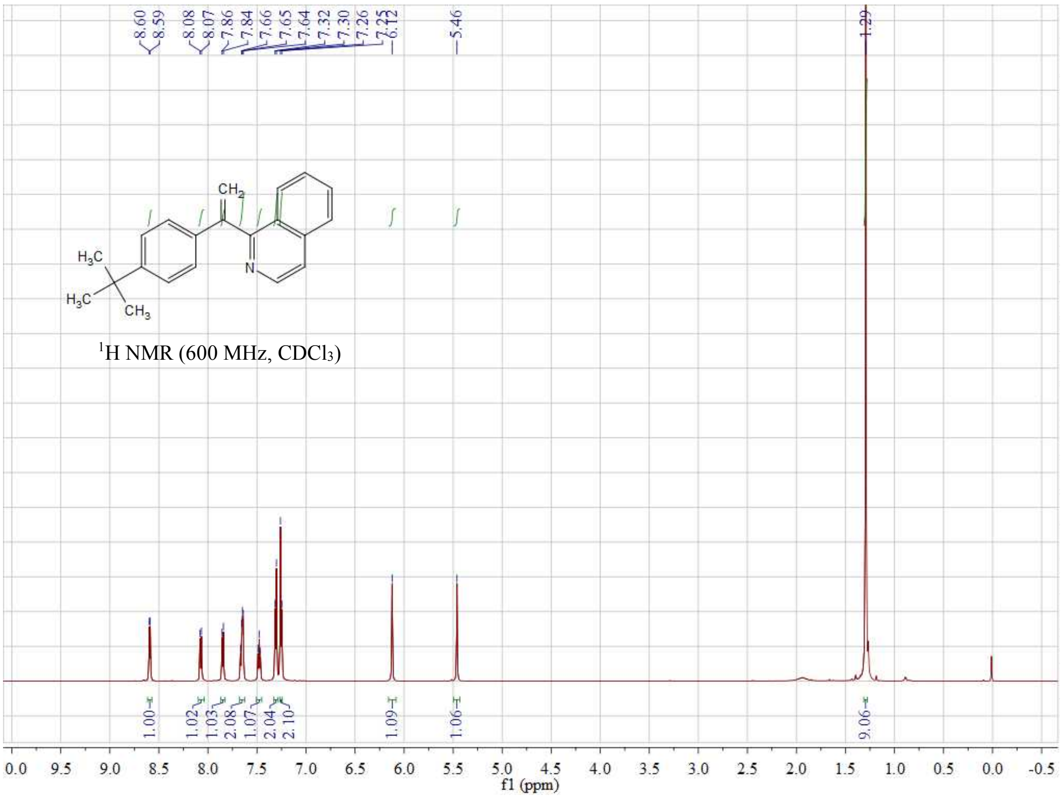

Supplementary Figure 105: ^{1}H NMR Spectra of 1-(1-(4-(tert-butyl)phenyl)vinyl) isoquinoline (60)

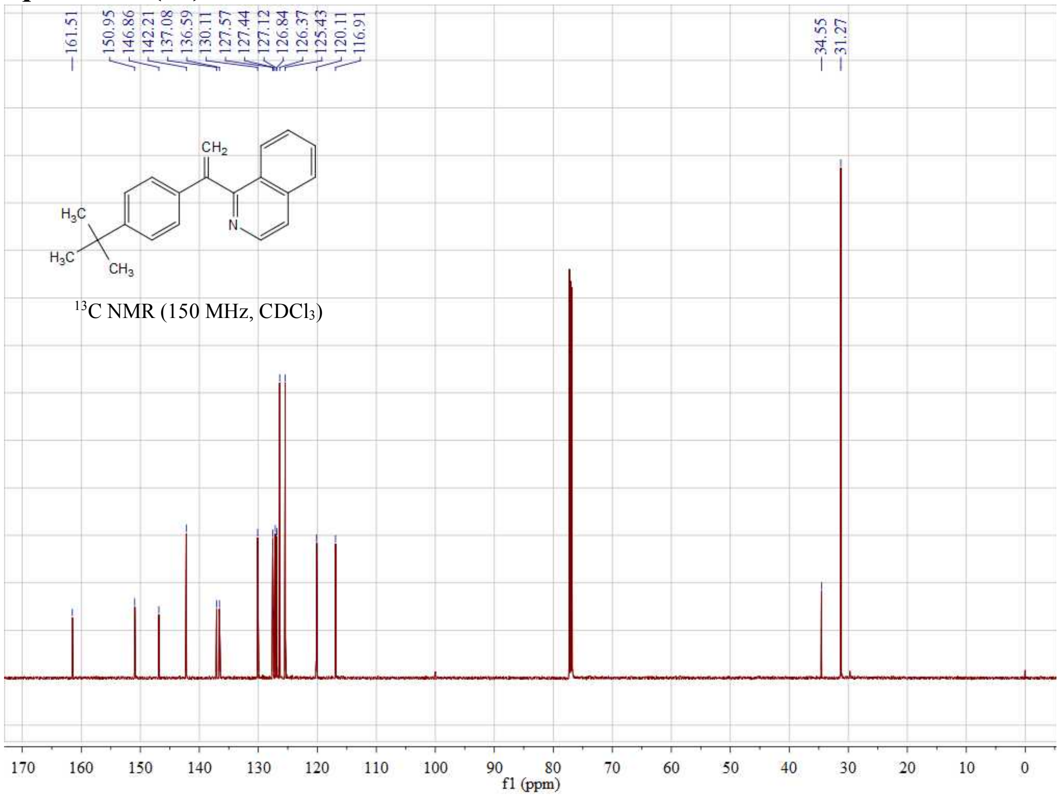

Supplementary Figure 106: ^{13}C NMR Spectra of 1-(1-(4-(tert-butyl)phenyl)vinyl) isoquinoline (60)

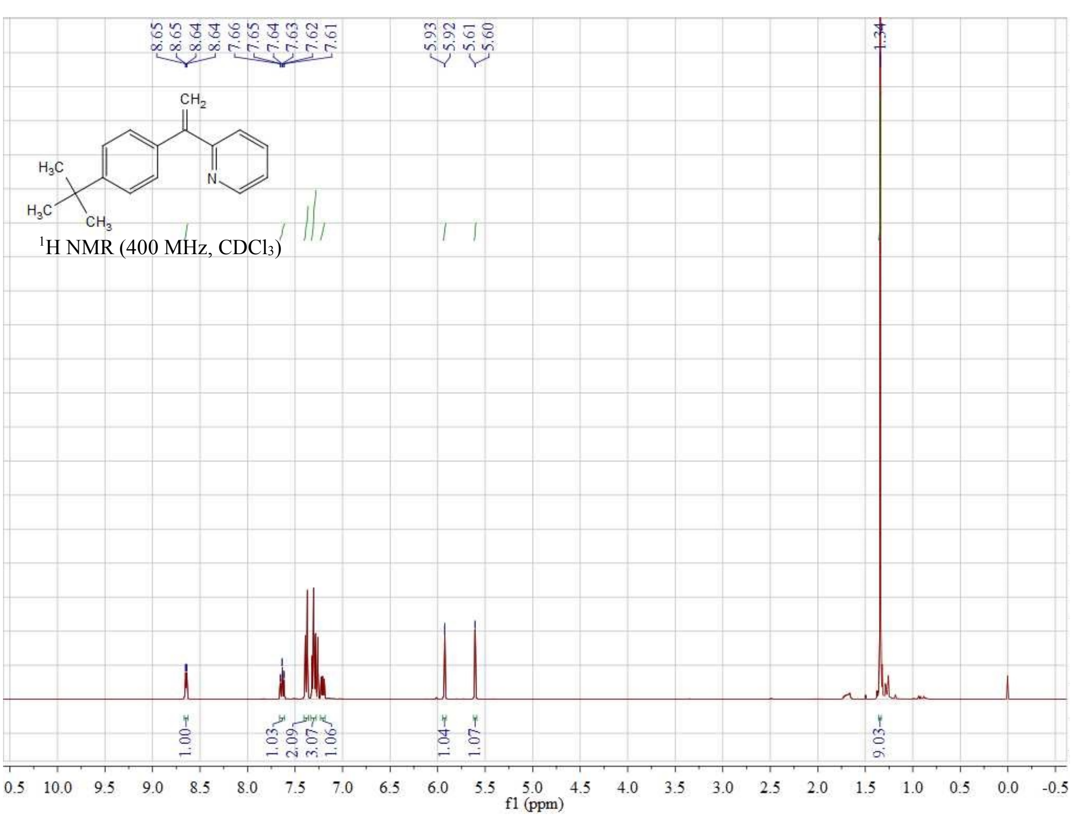

Supplementary Figure 107: ^{1}H NMR Spectra of 2-(1-(4-(*tert*-Butyl)phenyl)vinyl) pyridine (13)

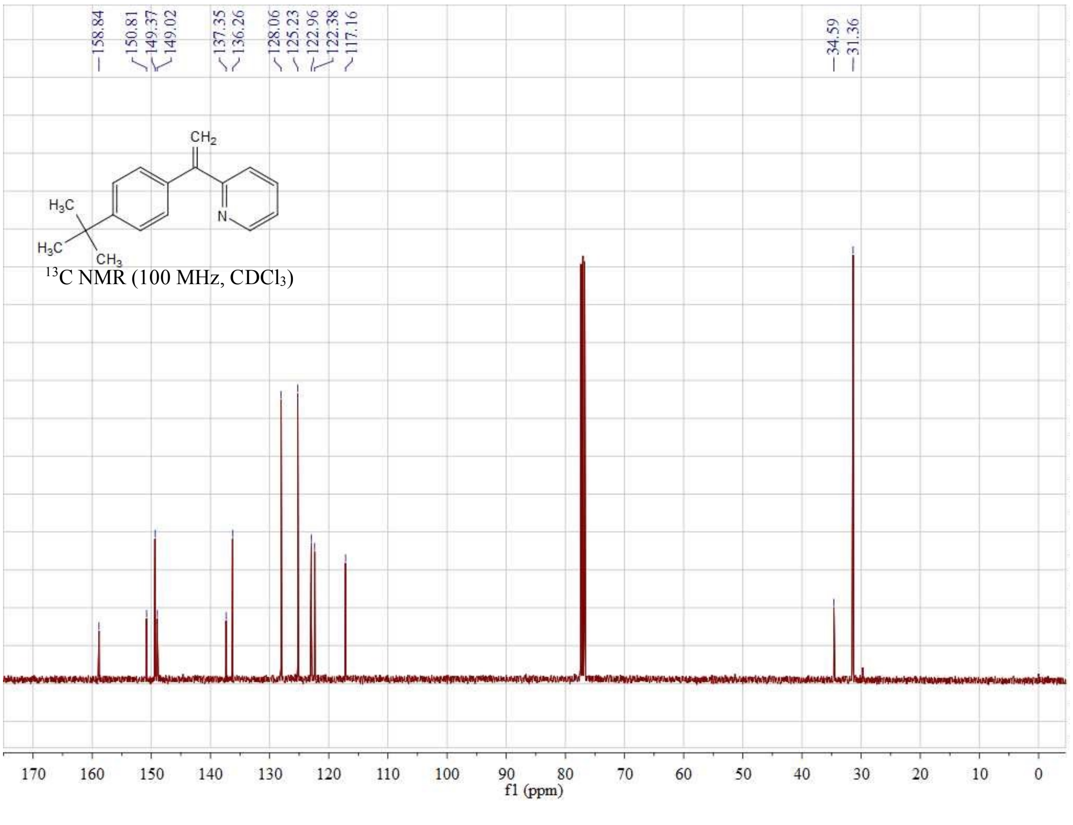

Supplementary Figure 108: ^{13}C NMR Spectra of 2-(1-(4-(*tert*-Butyl)phenyl)vinyl) pyridine (13)

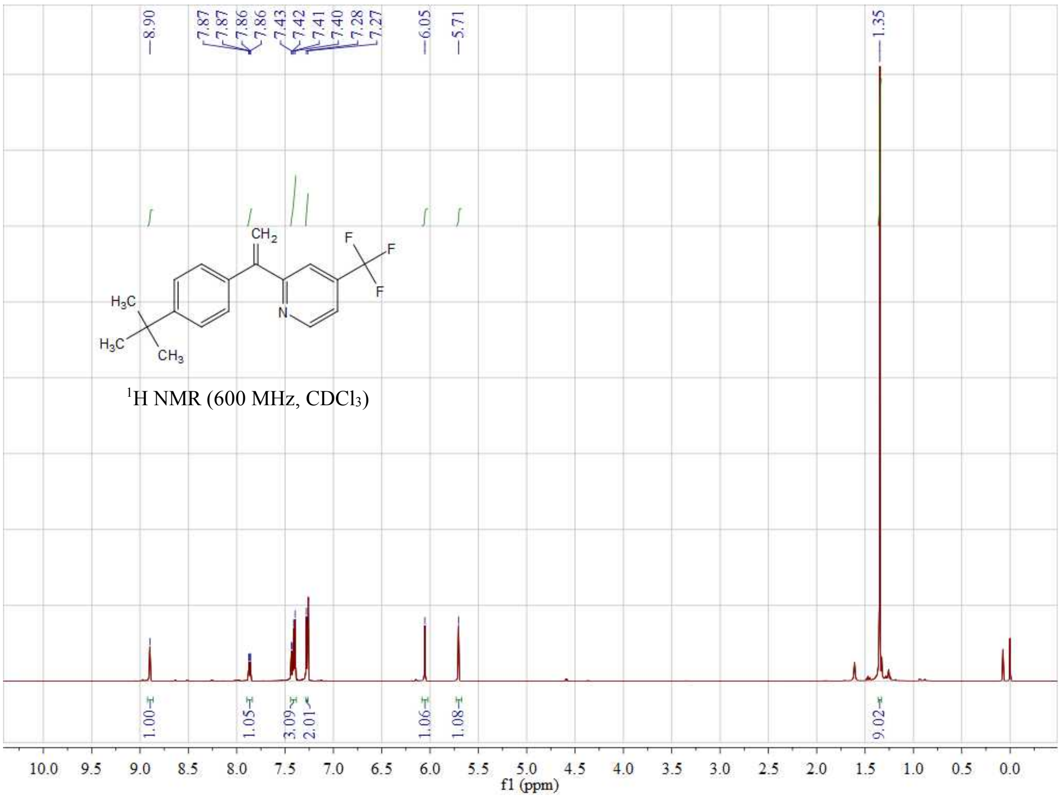

Supplementary Figure 109: ^{1}H NMR Spectra of 2-(1-(4-(tert-butyl)phenyl)vinyl)-4-(trifluoromethyl)pyridine (61)

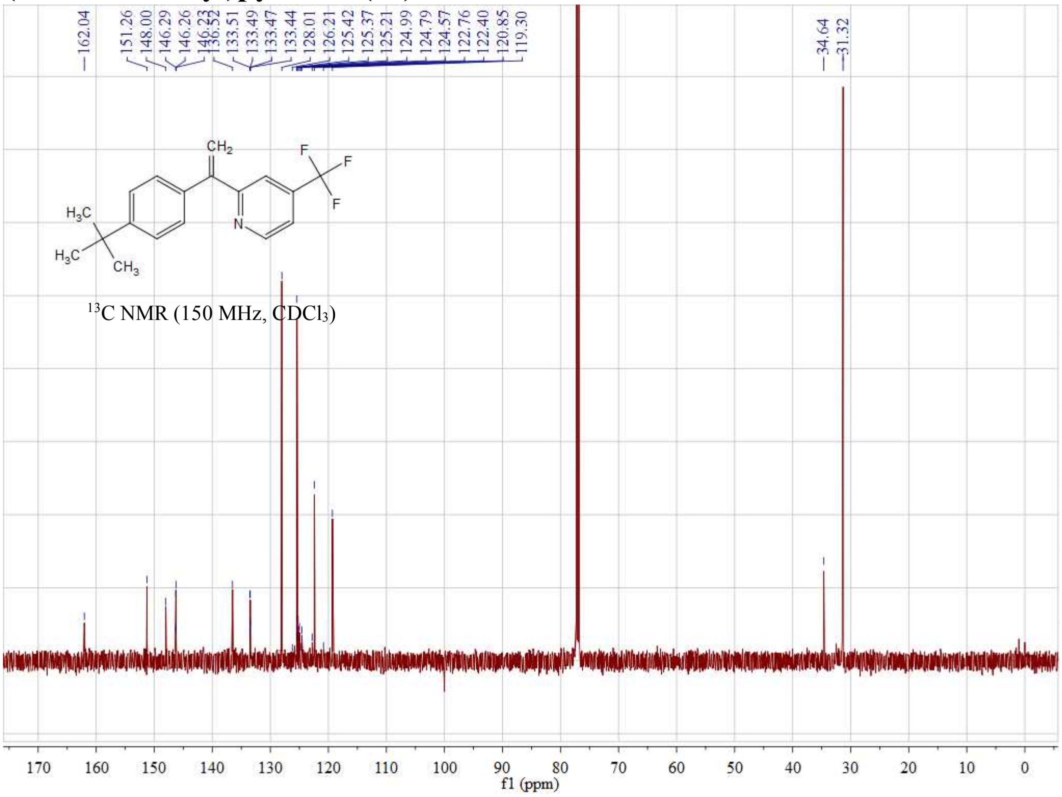

Supplementary Figure 110: ^{13}C NMR Spectra of 2-(1-(4-(tert-butyl)phenyl)vinyl)-4-(trifluoromethyl)pyridine (61)

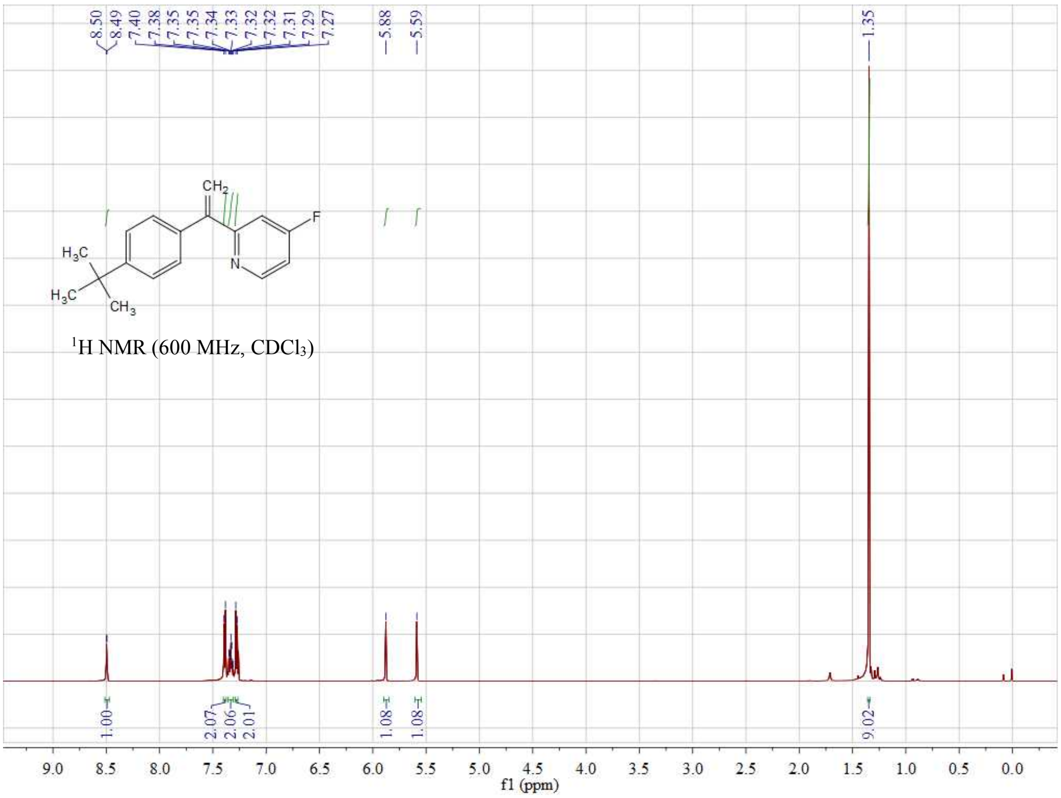

Supplementary Figure 111: ^{1}H NMR Spectra of 2-(1-(4-(tert-butyl)phenyl)vinyl)-4-fluoropyridine (62)

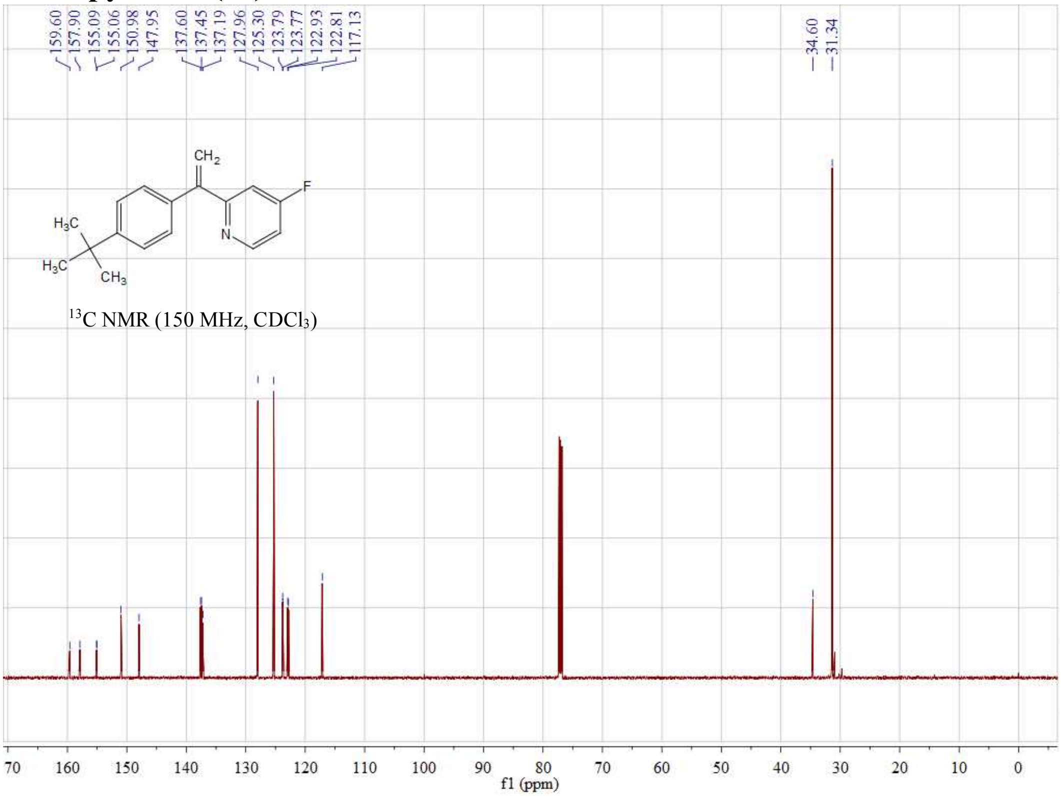

Supplementary Figure 112: ^{13}C NMR Spectra of 2-(1-(4-(tert-butyl)phenyl)vinyl)-4-fluoropyridine (62)

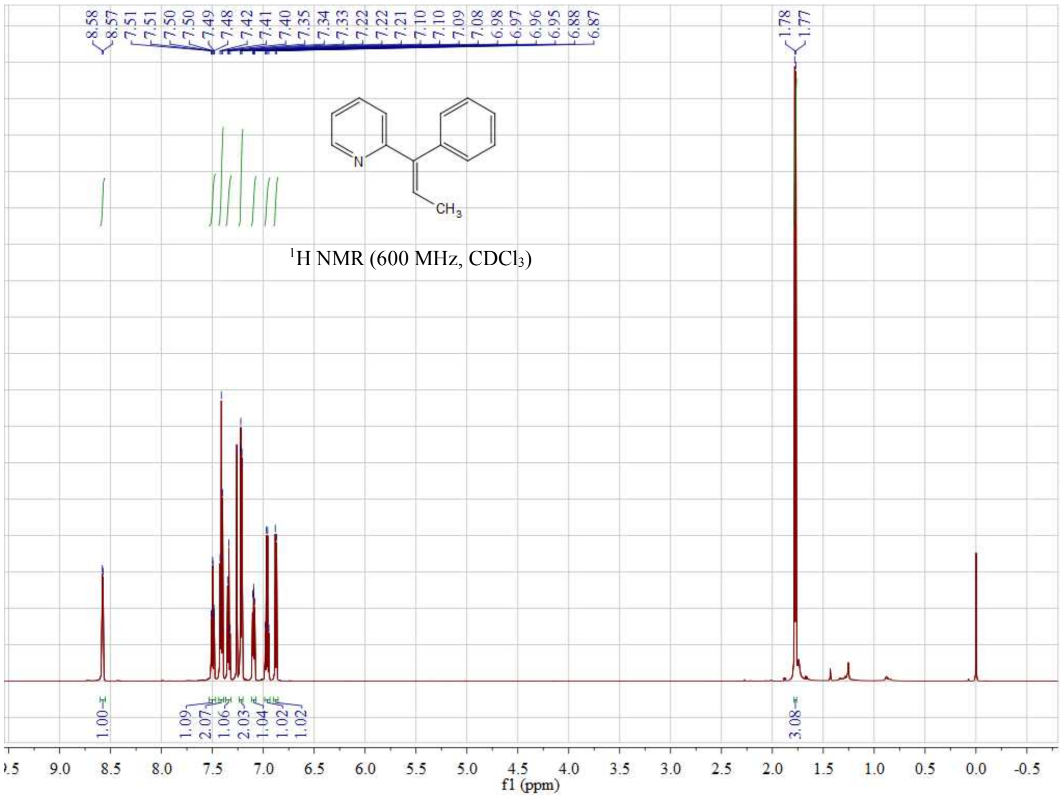

Supplementary Figure 113: ^{1}H NMR Spectra of (E)-2-(1-phenylprop-1-en-1-yl) pyridine (63)

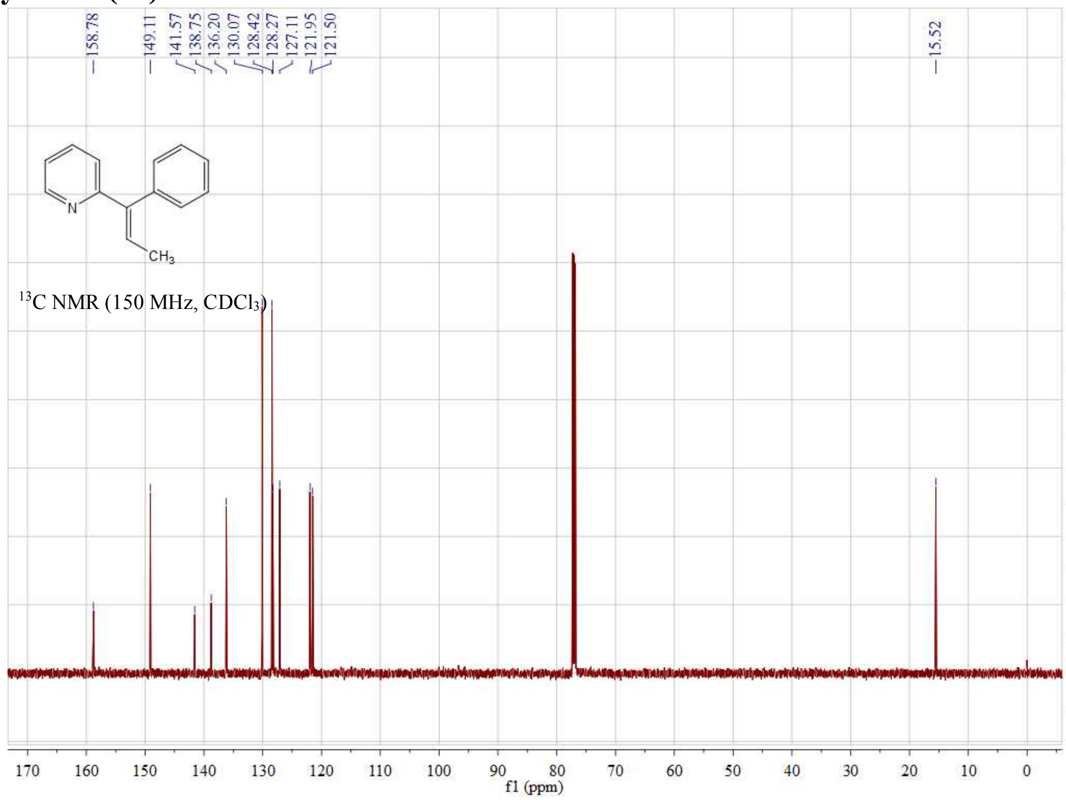

Supplementary Figure 114: ^{13}C NMR Spectra of (E)-2-(1-phenylprop-1-en-1-yl) pyridine (63)

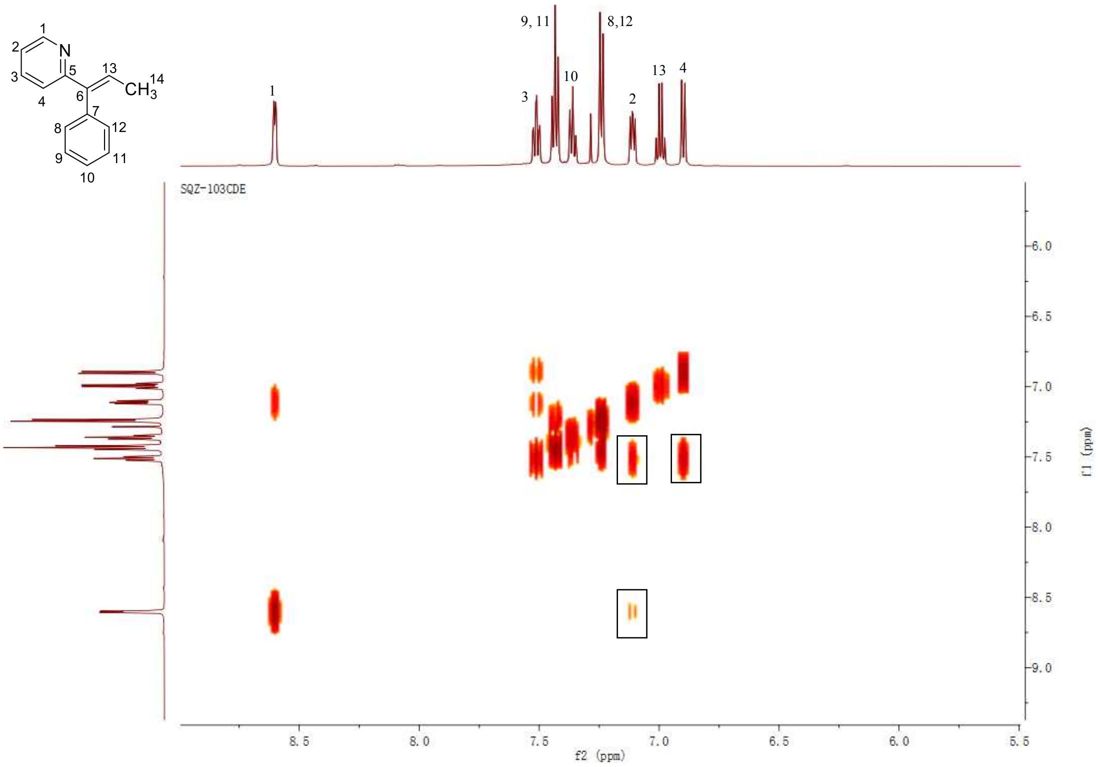

Supplementary Figure 115: H-H COSY Spectra of (E)-2-(1-phenylprop-1-en-1-yl) pyridine (63)

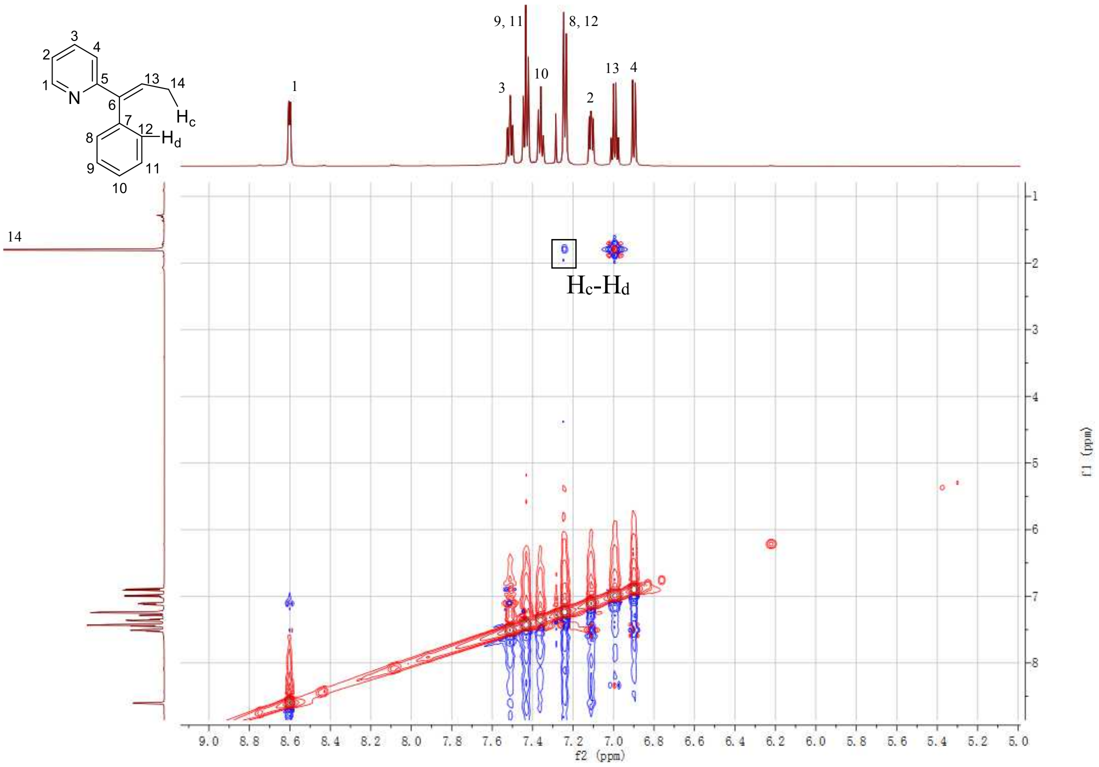

Supplementary Figure 116: NOESY Spectra of (E)-2-(1-phenylprop-1-en-1-yl) pyridine (63)

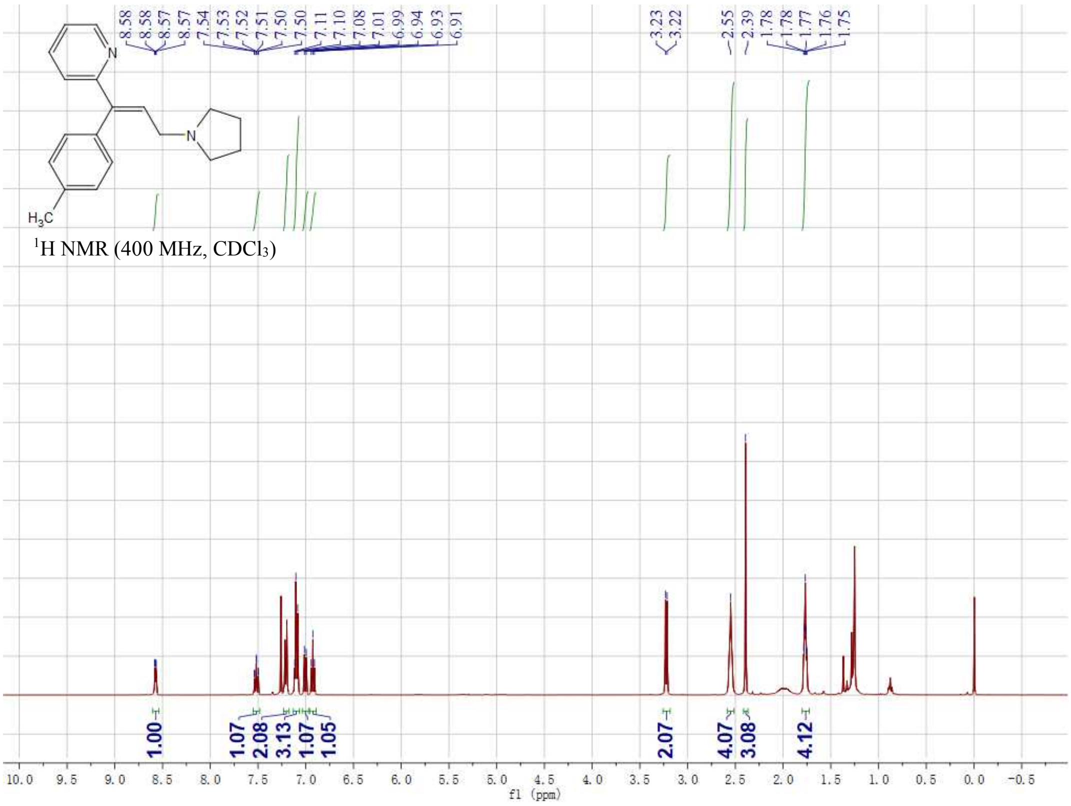

Supplementary Figure 117: ^{1}H NMR Spectra of (E)-2-(3-(pyrrolidin-1-yl)-1-(p-tolyl)prop-1-en-1-yl)pyridine

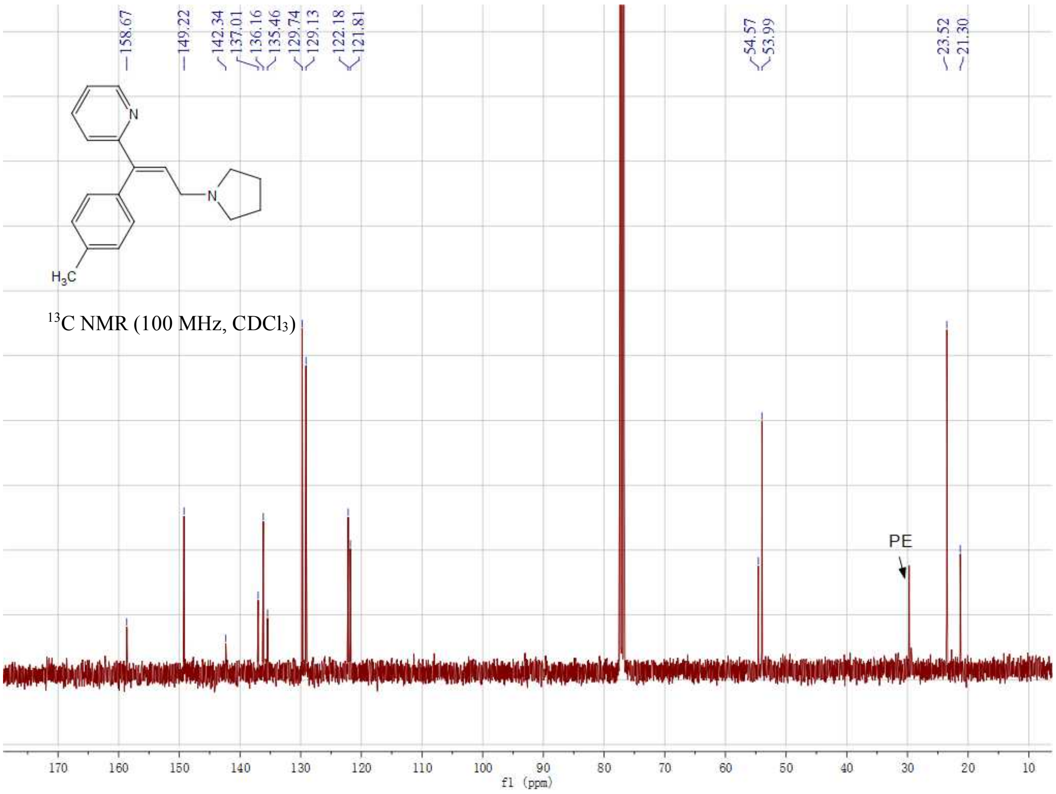

Supplementary Figure 118: ^{13}C NMR Spectra of (E)-2-(3-(pyrrolidin-1-yl)-1-(p-tolyl)prop-1-en-1-yl)pyridine

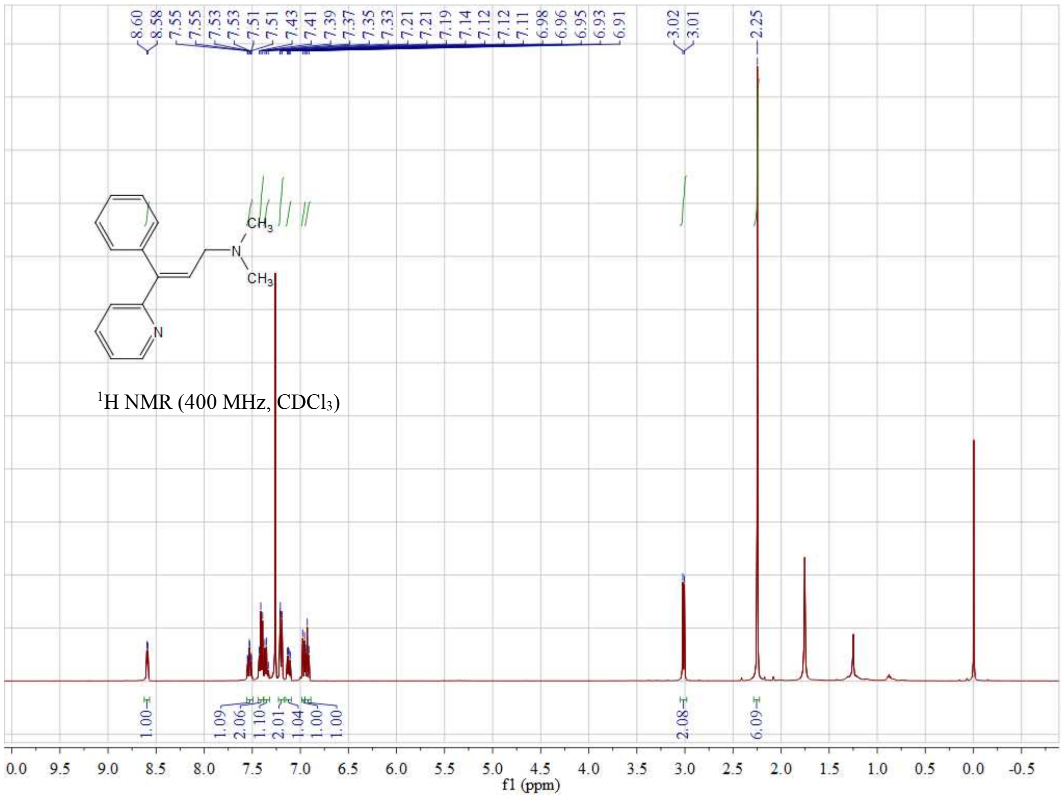

Supplementary Figure 119: ^{1}H NMR Spectra of (E)-N,N-dimethyl-3-phenyl-3-(pyridin-2-yl)prop-2-en-1-amine (67)

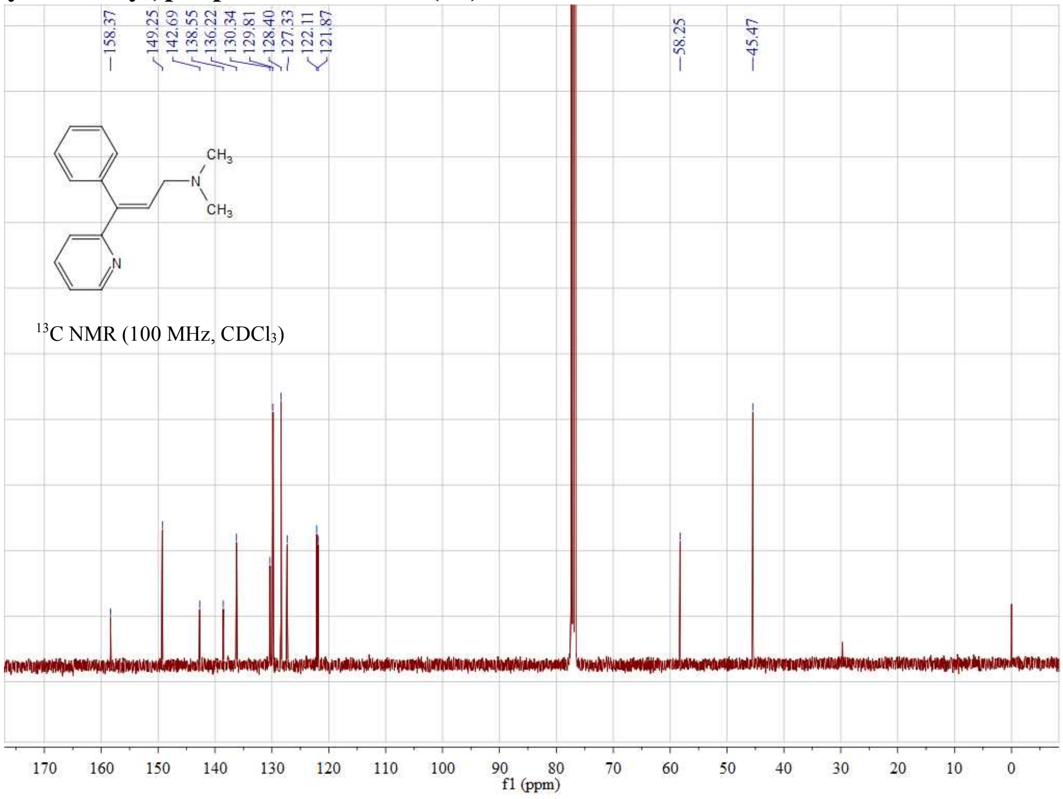

Supplementary Figure 120: ^{13}C NMR Spectra of (E)-N,N-dimethyl-3-phenyl-3-(pyridin-2-yl)prop-2-en-1-amine (67)

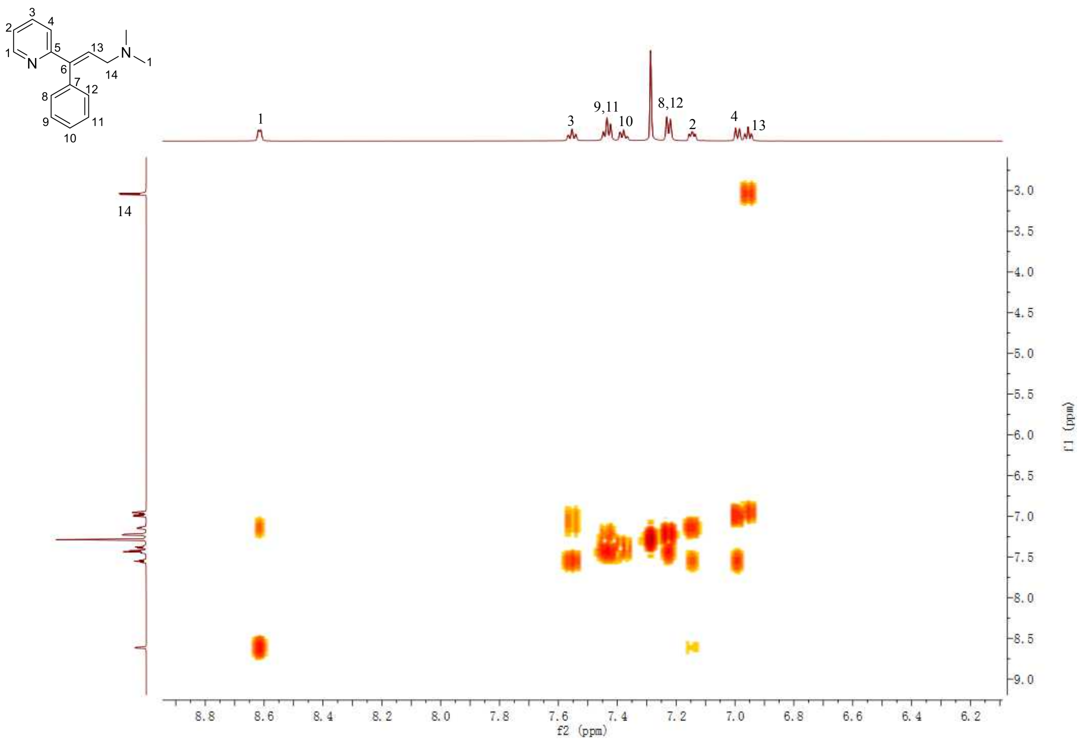

Supplementary Figure 121: H-H COSY Spectra of (E)-N,N-dimethyl-3-phenyl-3-(pyridin-2-yl)prop-2-en-1-amine (67)

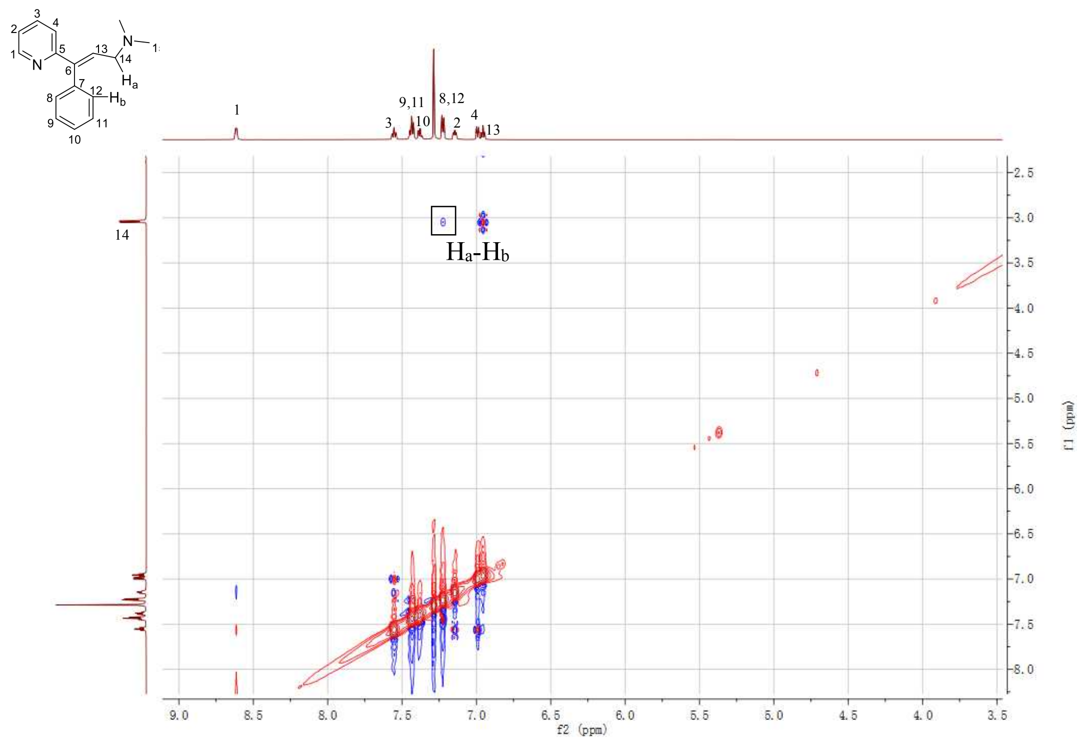

Supplementary Figure 122: NOESY Spectra of (E)-N,N-dimethyl-3-phenyl-3-(pyridin-2-yl)prop-2-en-1-amine (67)

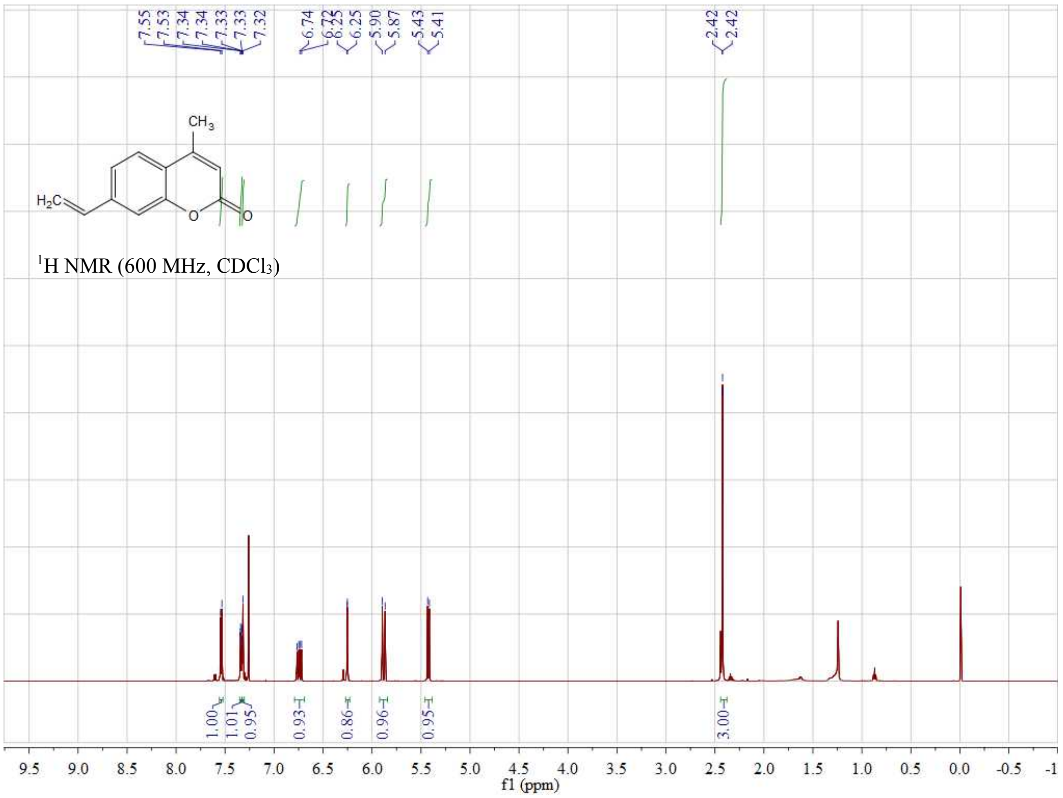

Supplementary Figure 123: ^{1}H NMR Spectra of 4-methyl-7-vinyl-2H-chromen-2-one (S2)

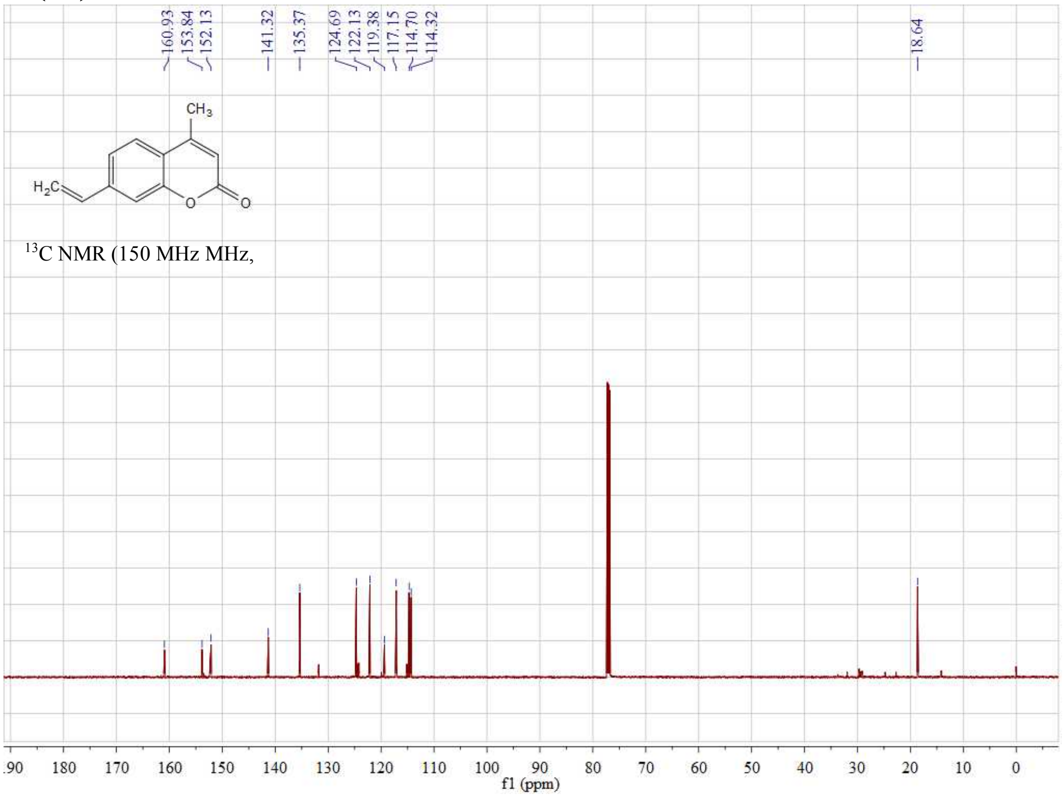

Supplementary Figure 124: ^{13}C NMR Spectra of 4-methyl-7-vinyl-2H-chromen-2-one (S2)

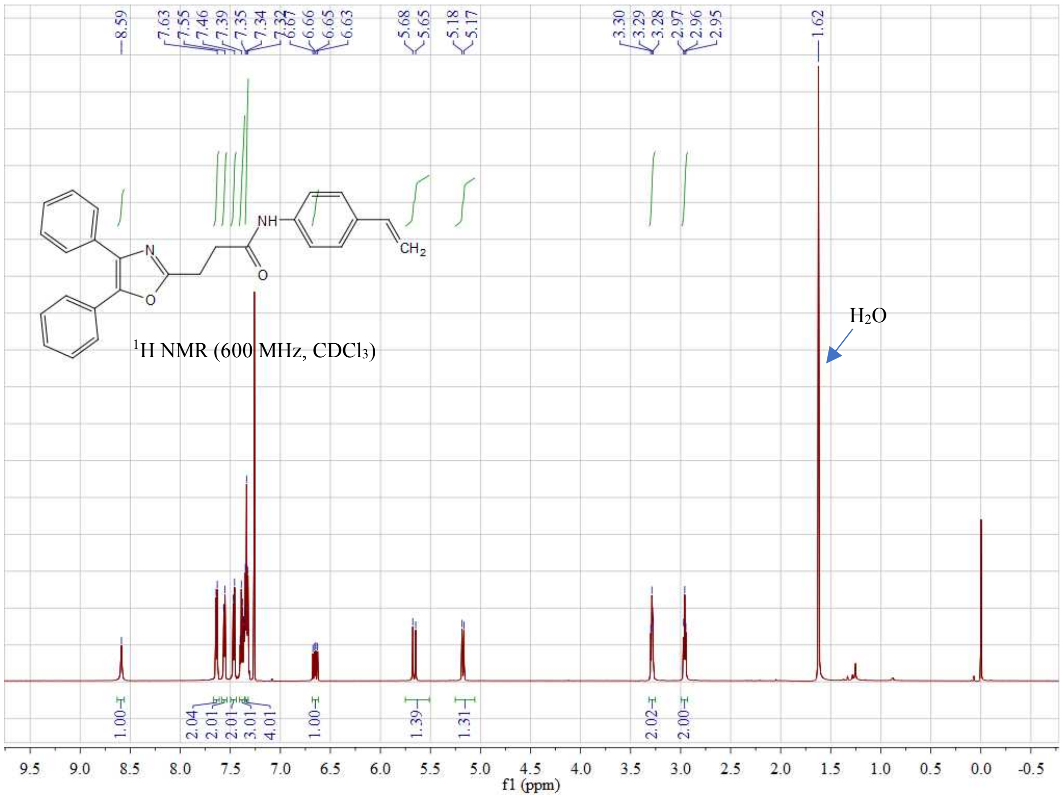

Supplementary Figure 125: ^{1}H NMR Spectra of 3-(4,5-diphenyloxazol-2-yl)-N-(4-vinylphenyl)propenamide (S3)

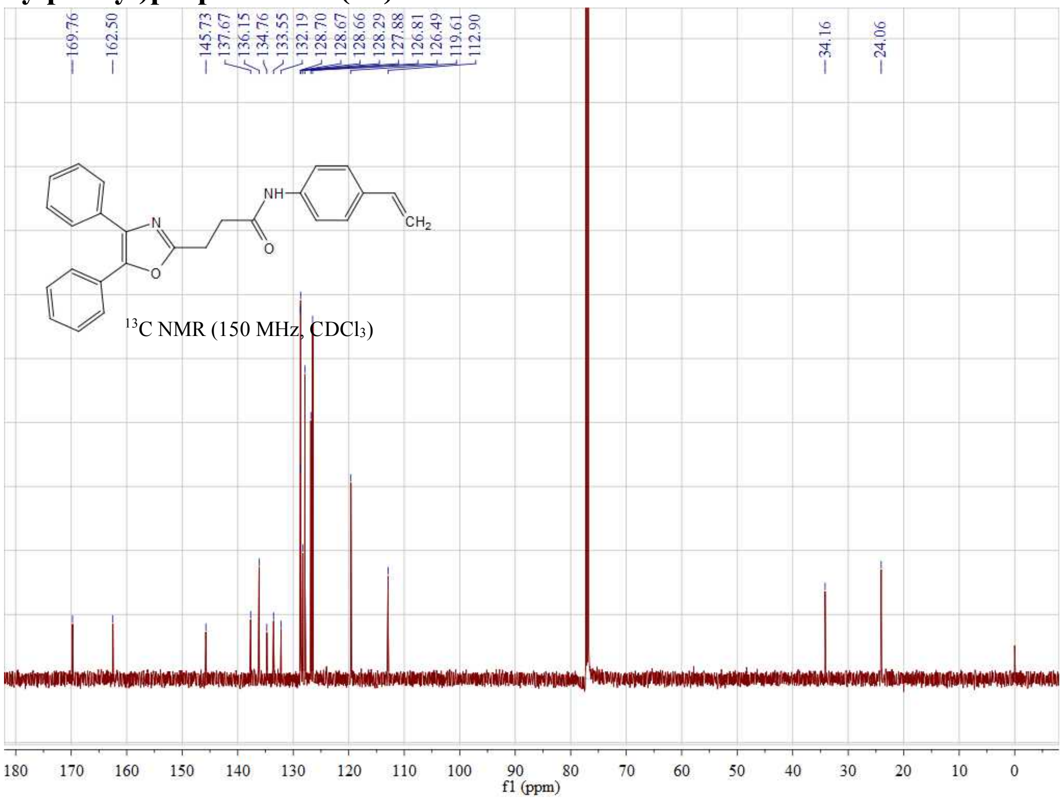

Supplementary Figure 126: ^{13}C NMR Spectra of 3-(4,5-diphenyloxazol-2-yl)-N-(4-vinylphenyl)propenamide (S3)

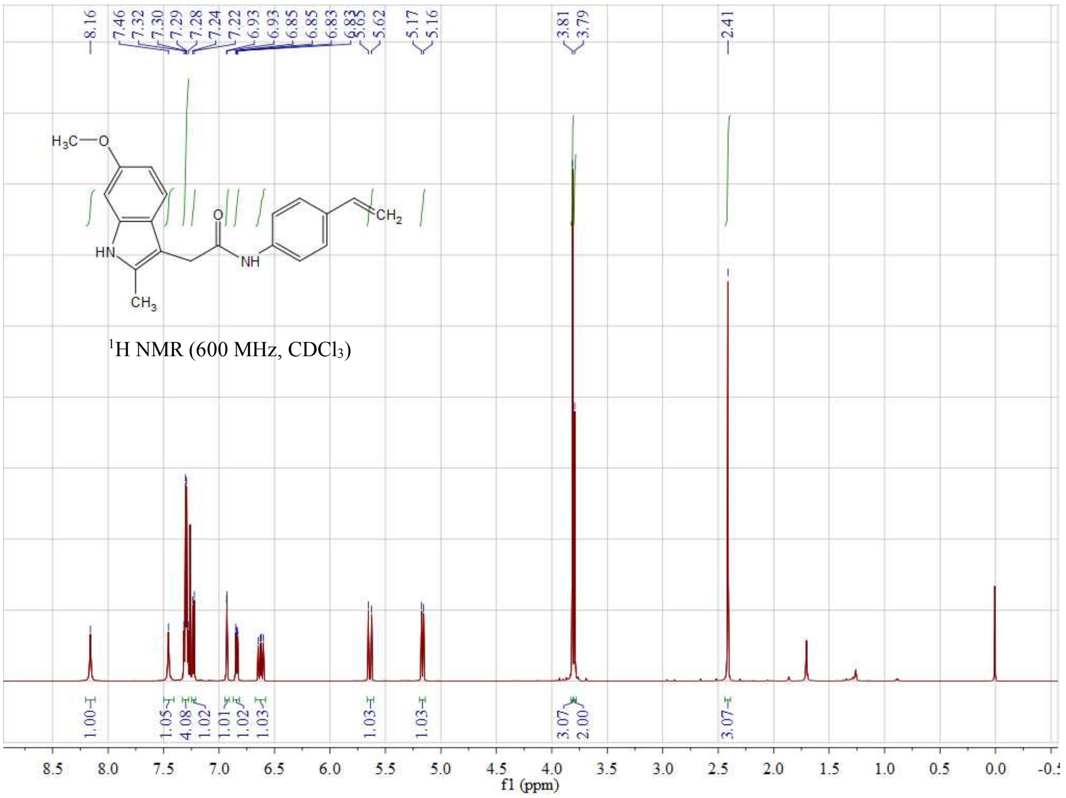

Supplementary Figure 127: ^{1}H NMR Spectra of 2-(6-methoxy-2-methyl-1H-indol-3-yl)-N-(4-vinylphenyl)acetamide (S4)

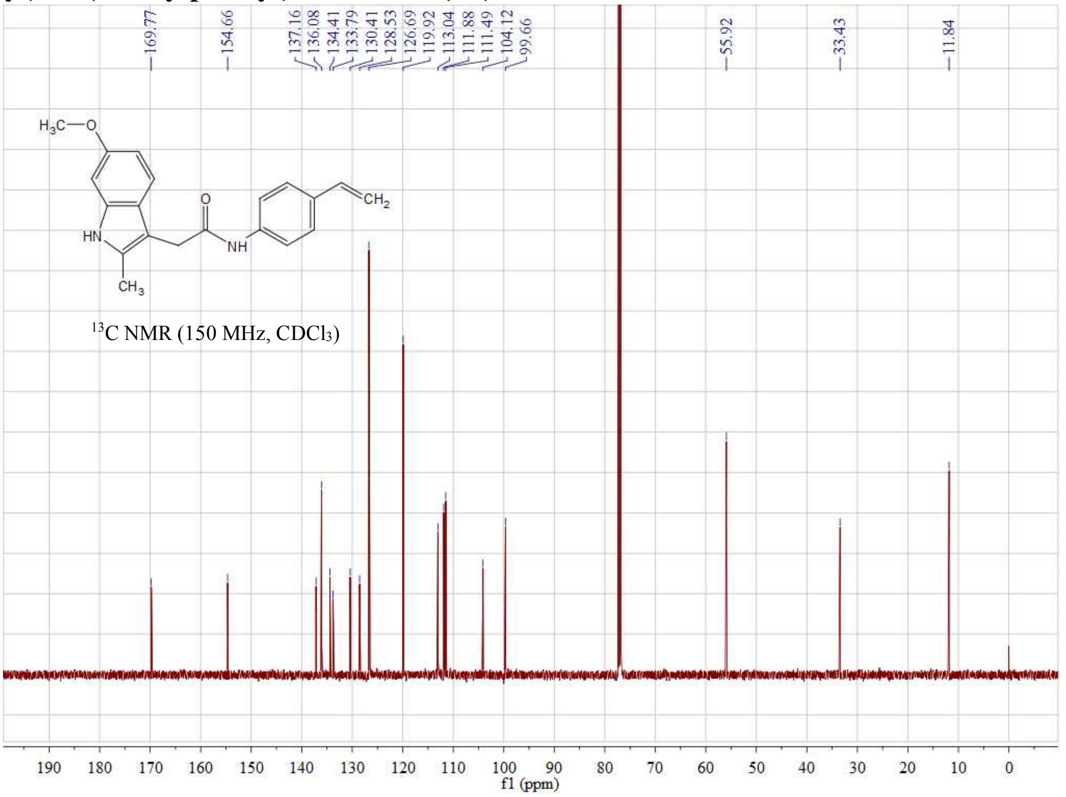

Supplementary Figure 128: ^{13}C NMR Spectra of 2-(6-methoxy-2-methyl-1H-indol-3-yl)-N-(4-vinylphenyl)acetamide (S4)

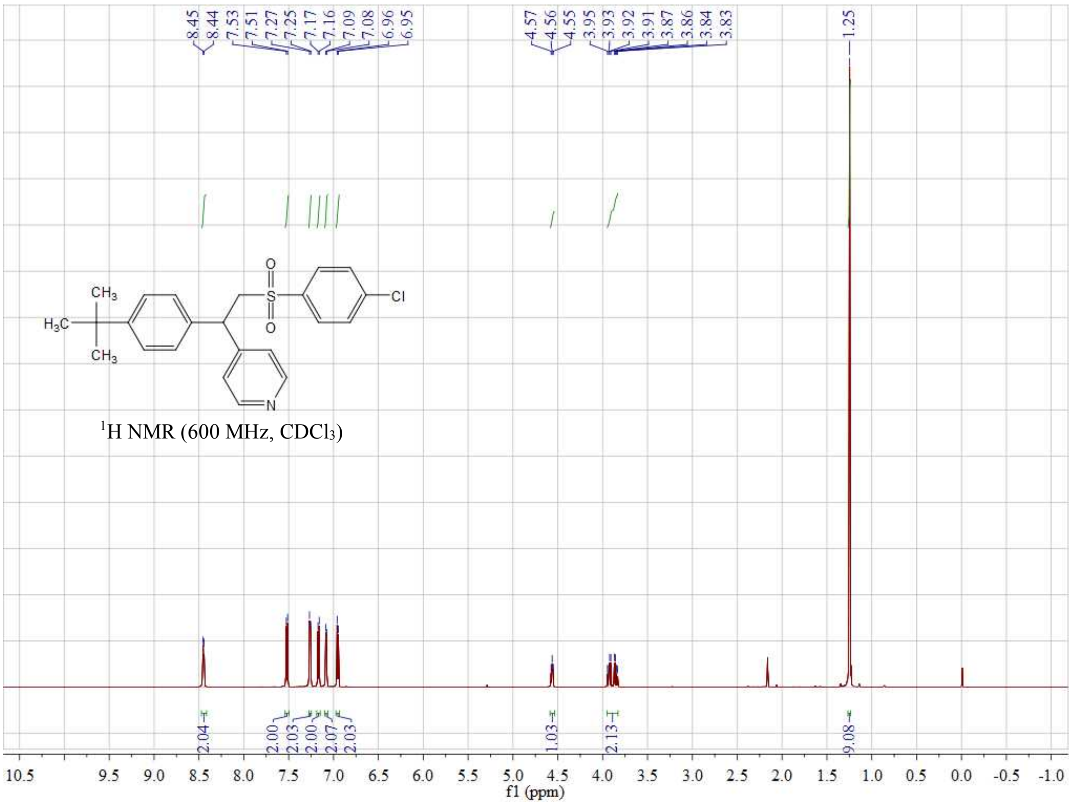

Supplementary Figure 129: ^{1}H NMR Spectra of 4-(1-(4-(tert-butyl)phenyl)-2-((4-chlorophenyl)sulfonyl)ethyl)pyridine (S6)

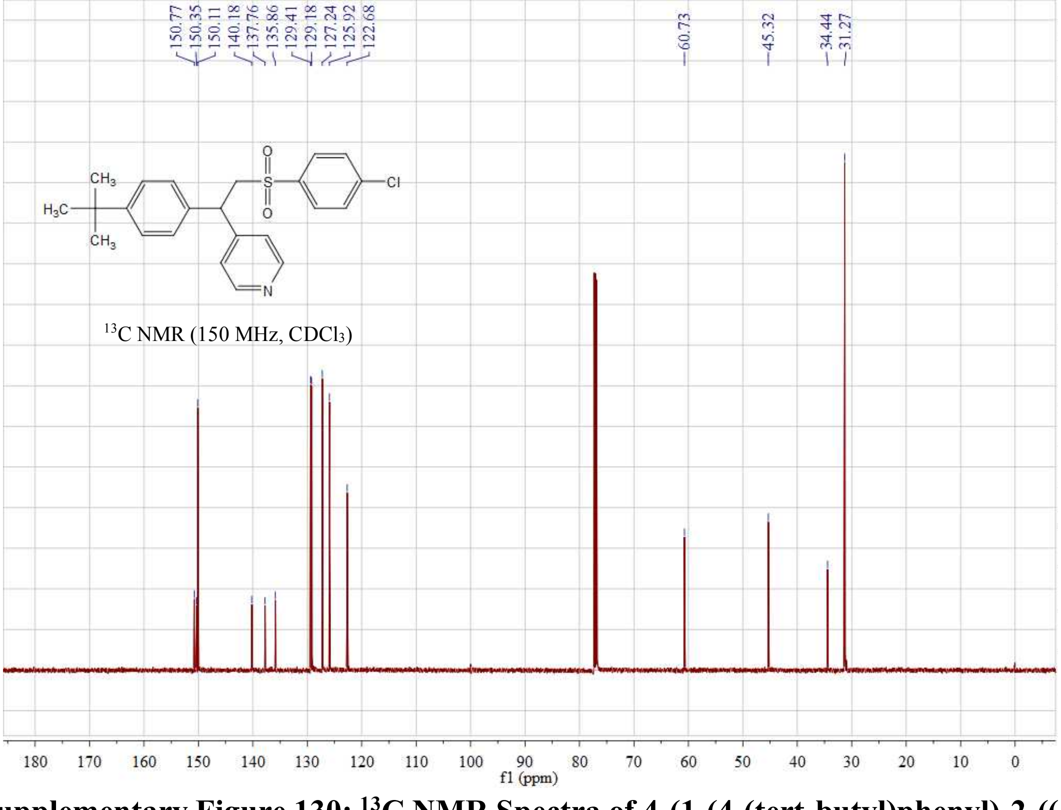

Supplementary Figure 130: ^{13}C NMR Spectra of 4-(1-(4-(tert-butyl)phenyl)-2-((4-chlorophenyl)sulfonyl)ethyl)pyridine (S6)

Supplementary References

(1) Tanaka, F., Mase, N., Barbas, CF. Design and Use of Fluorogenic Aldehydes for Monitoring the Progress of Aldehyde Transformations. *J. Am. Chem. Soc.* **126**, 3692-3693, (2004).

(2) Jing, J., Huo, X., Shen, J., Fu, J., Meng, Q., Zhang, W. Direct use of allylic alcohols and allylic amines in palladium-catalyzed allylic amination. *Chem. Commun.* **53**, 5151-5154, (2017).
